# Supplementary figures and images for: Effects of combination therapy of a CDK4/6 and MEK inhibitor in diffuse midline glioma preclinical models
Source: PLoS One. 2025 Dec 22;20(12):e0323235. doi: 10.1371/journal.pone.0323235 (PMC12721541; doi:10.1371/journal.pone.0323235)

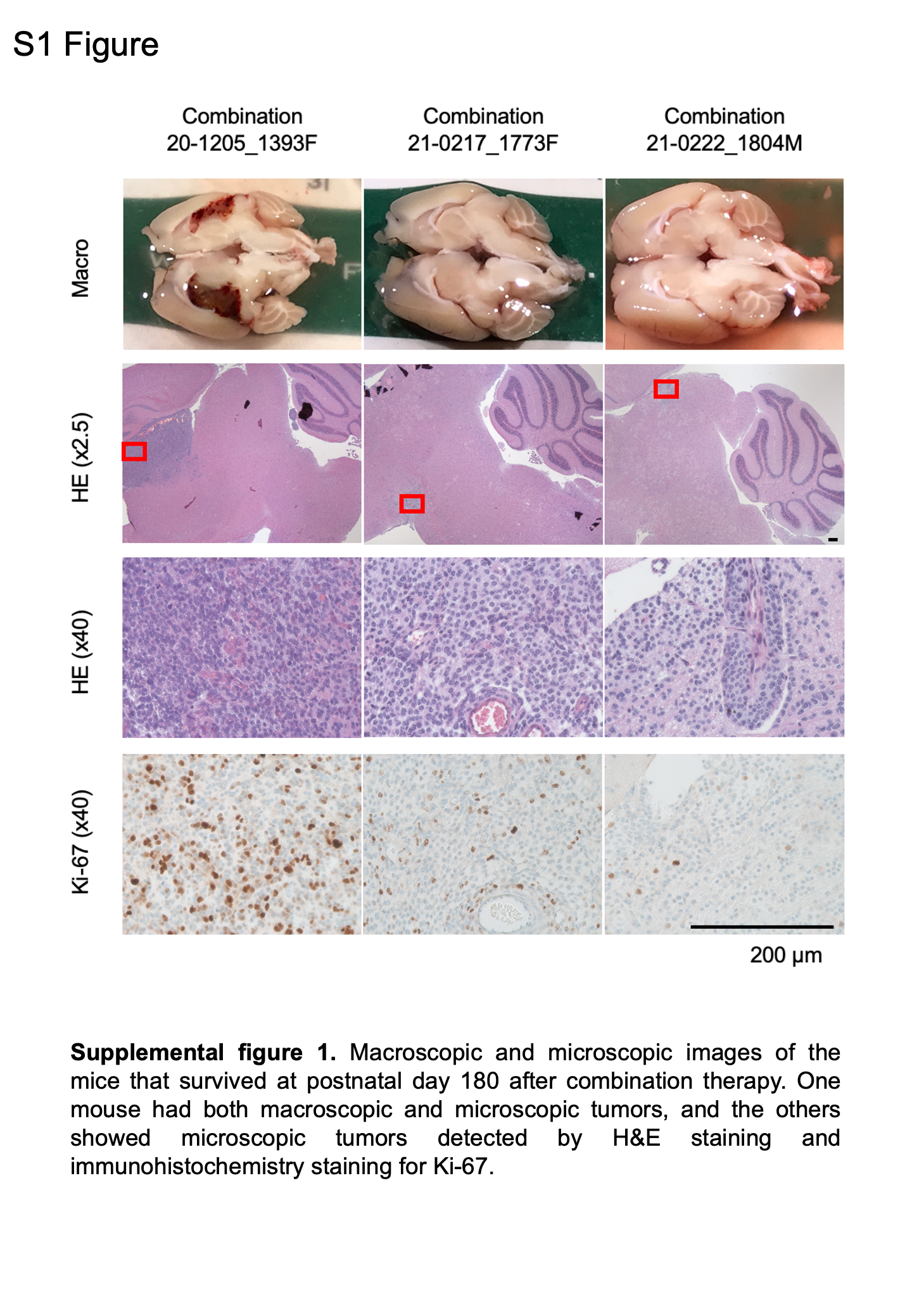

Supplement: S1 Fig — (TIFF) [file pone.0323235.s002.tiff]

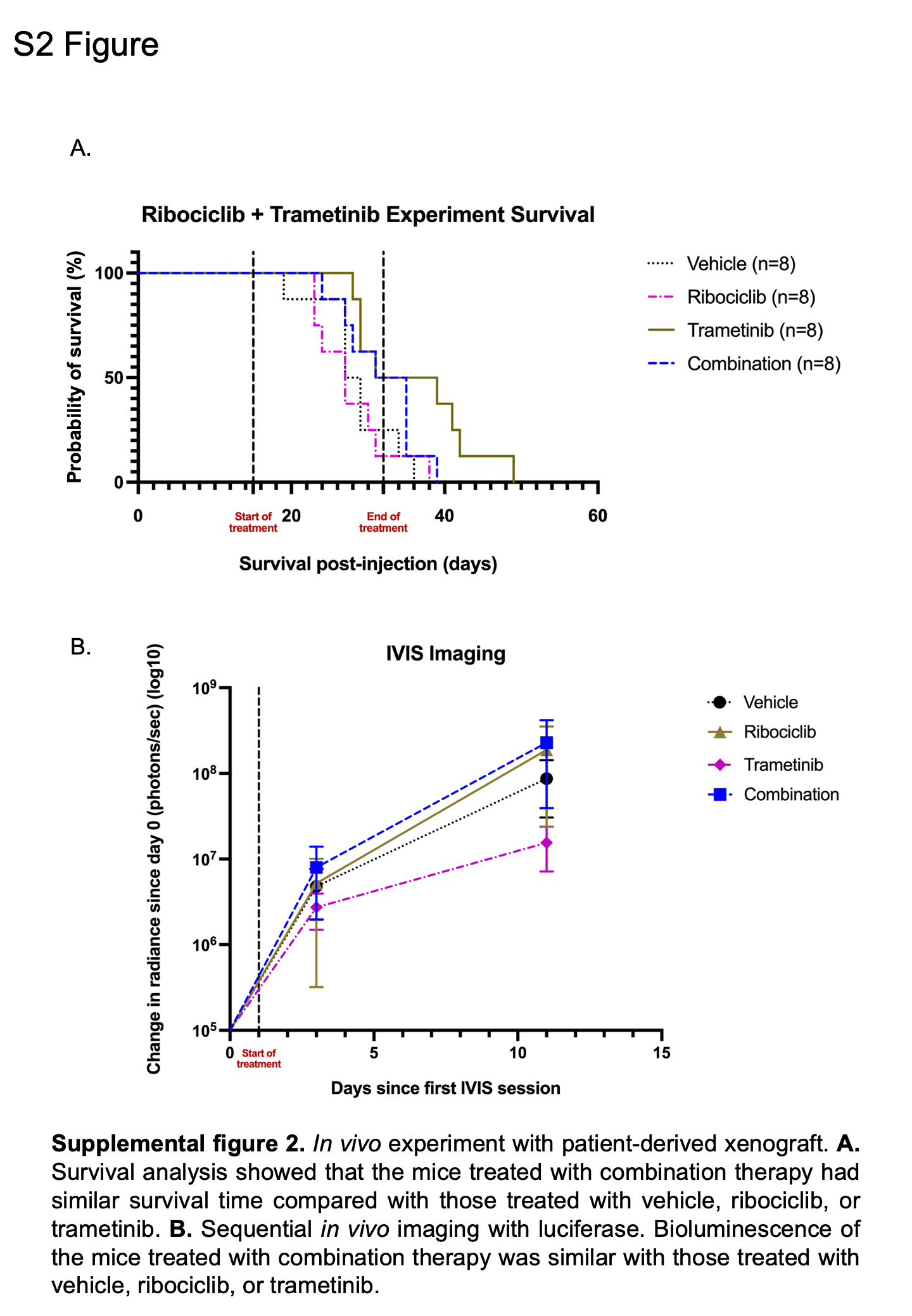

Supplement: S2 Fig — (TIFF) [file pone.0323235.s003.tiff]

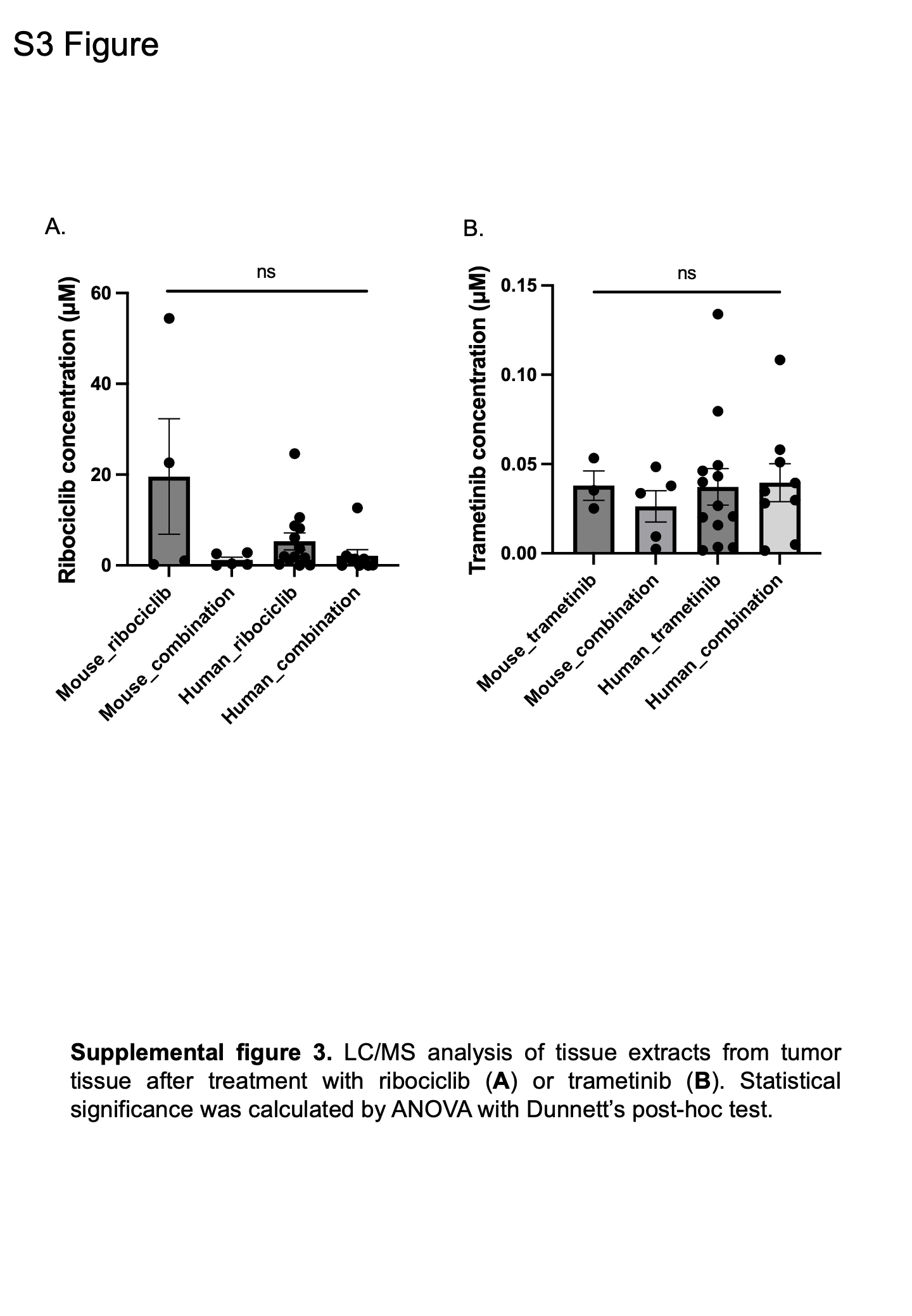

Supplement: S3 Fig — (TIFF) [file pone.0323235.s004.tiff]

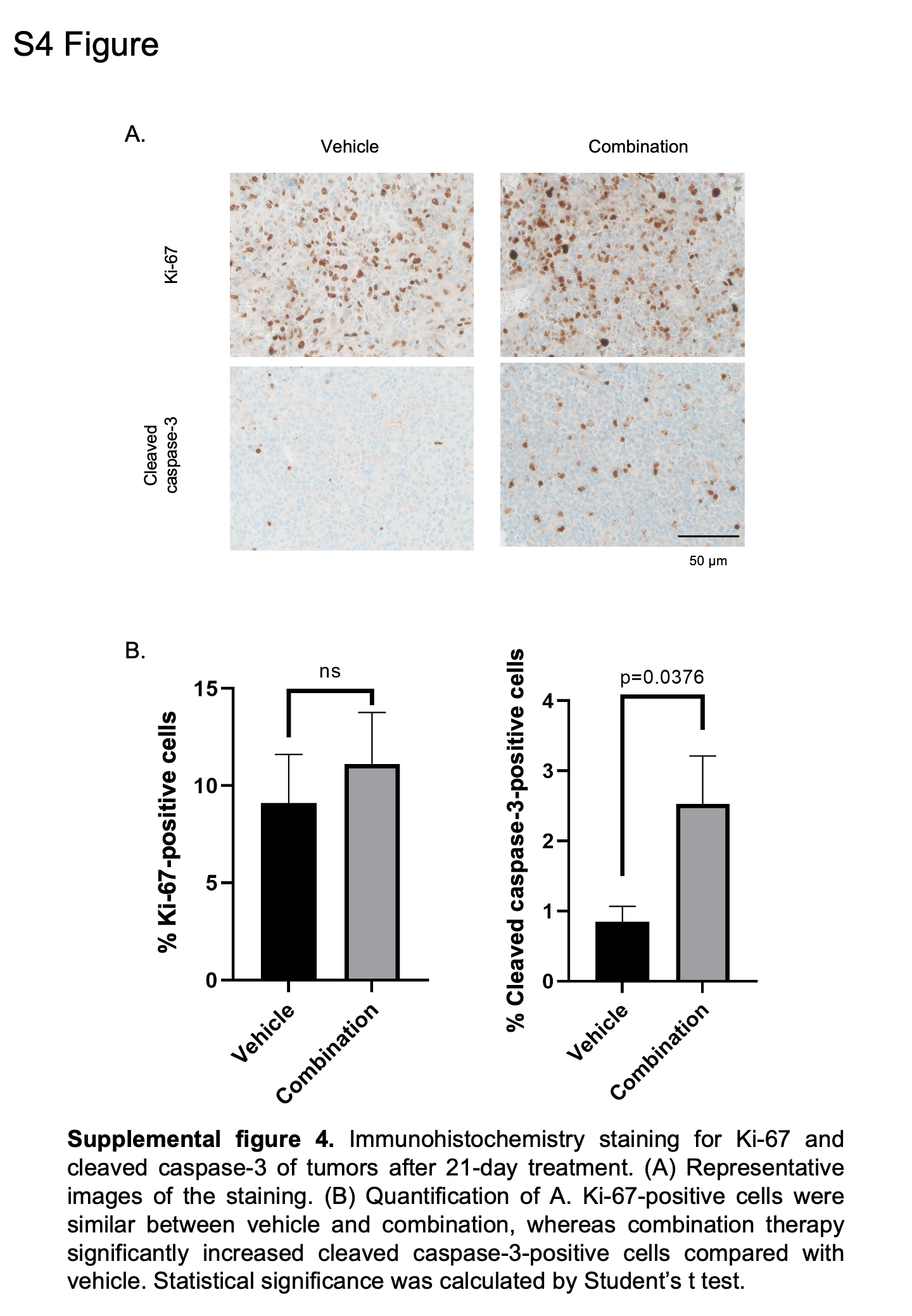

Supplement: S4 Fig — (TIFF) [file pone.0323235.s005.tiff]

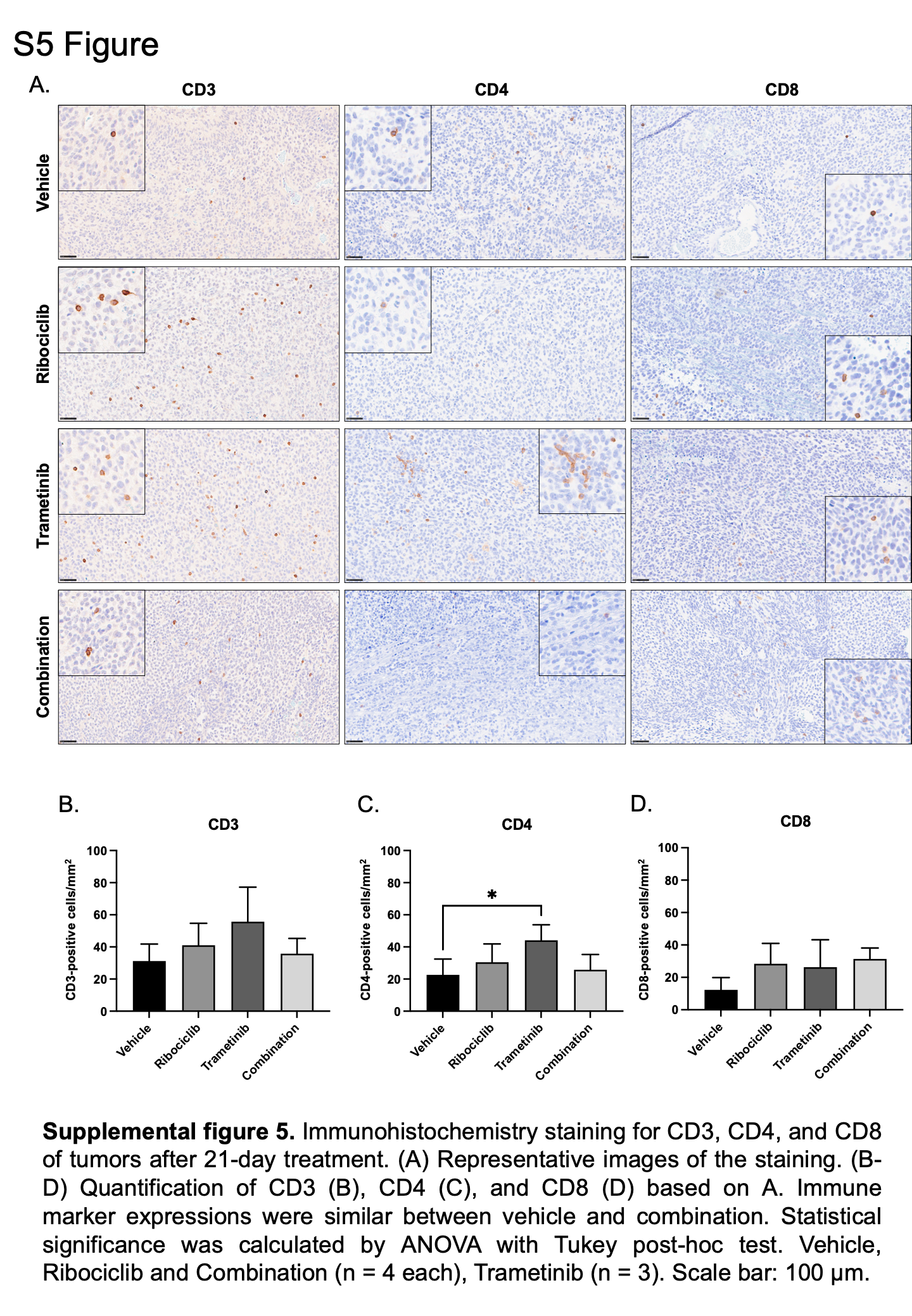

Supplement: S5 Fig — (TIFF) [file pone.0323235.s006.tiff]

23-0104-3  
Phospho-Erk

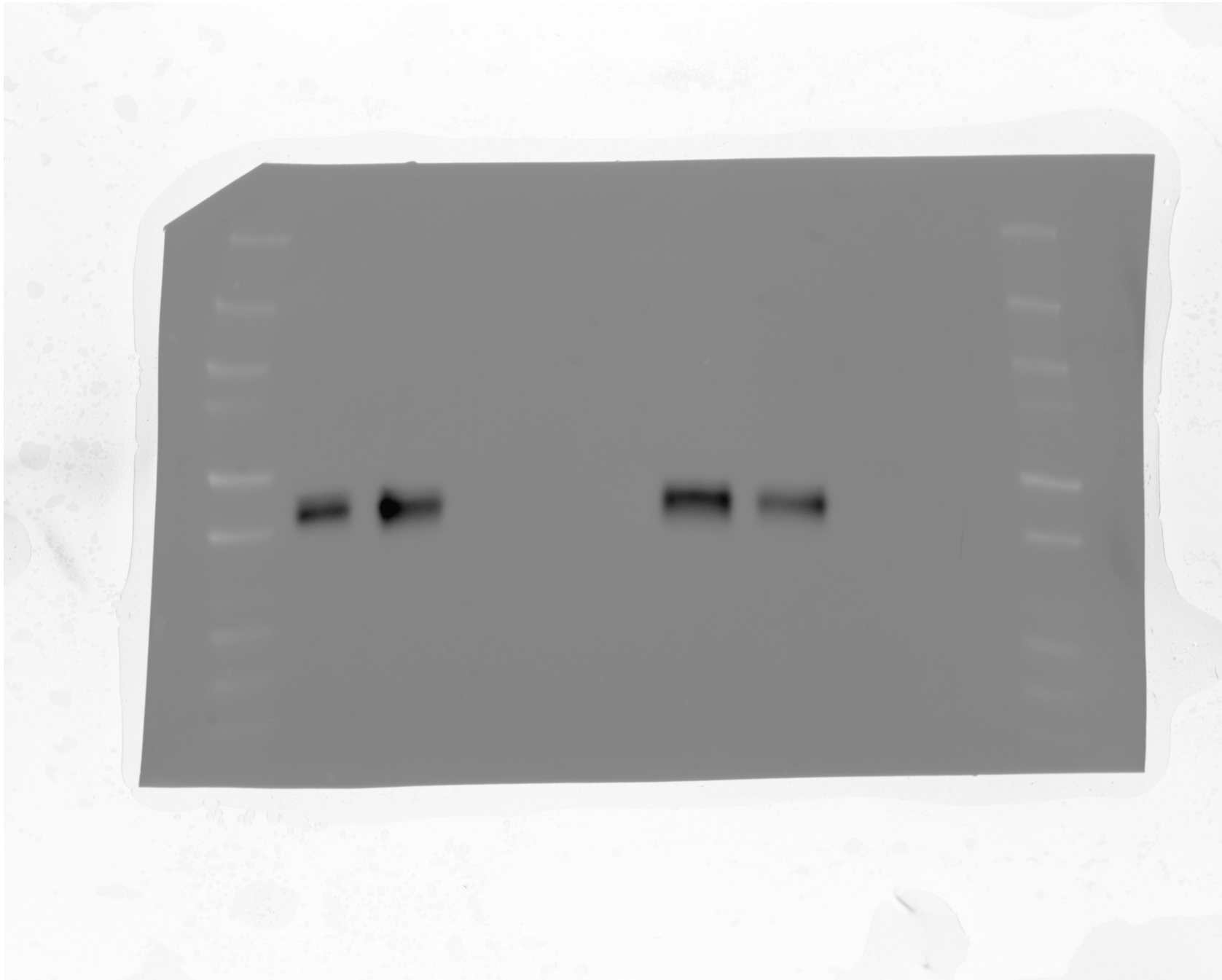

23-0104-3

Total Erk

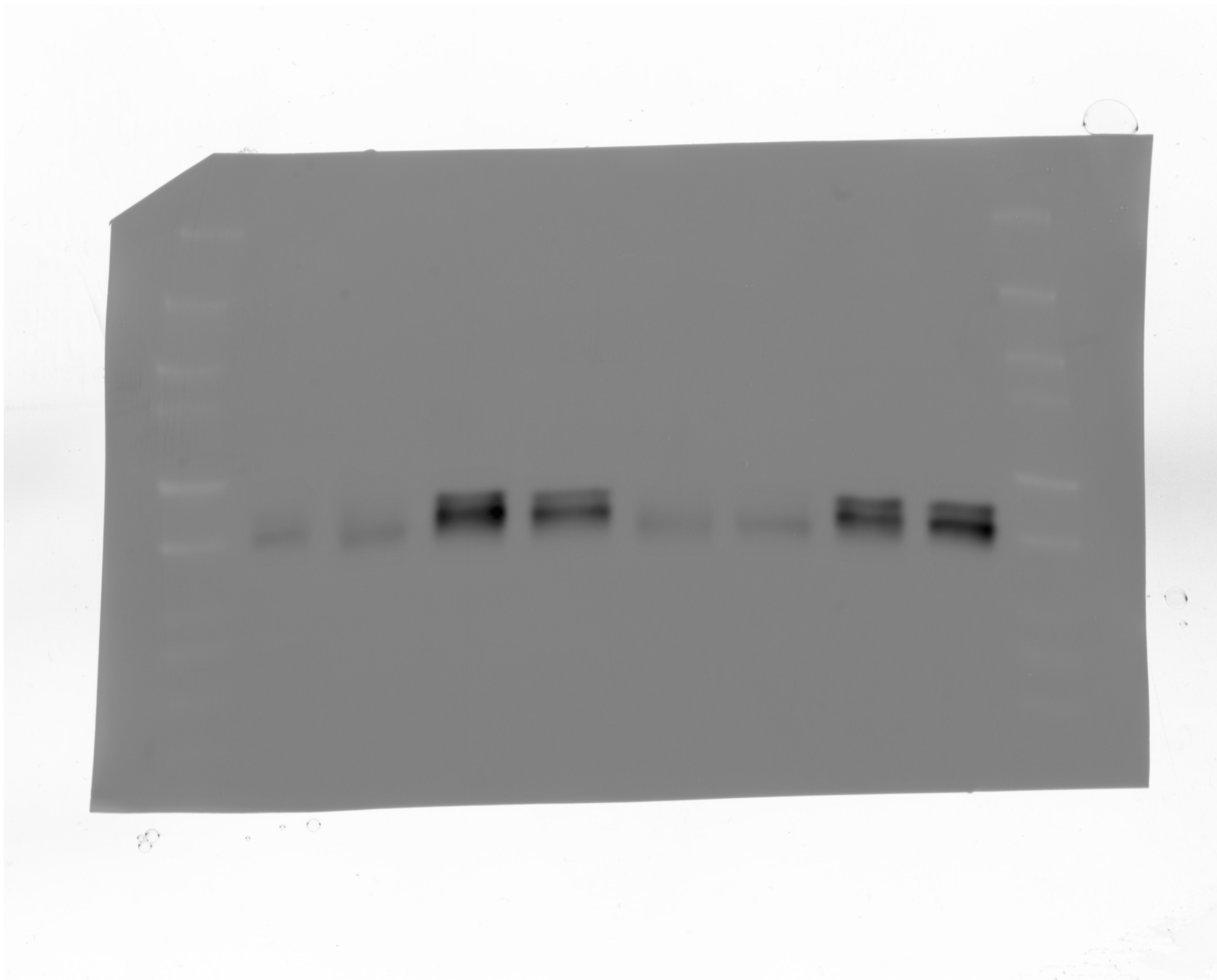

23-0104-3

Actin

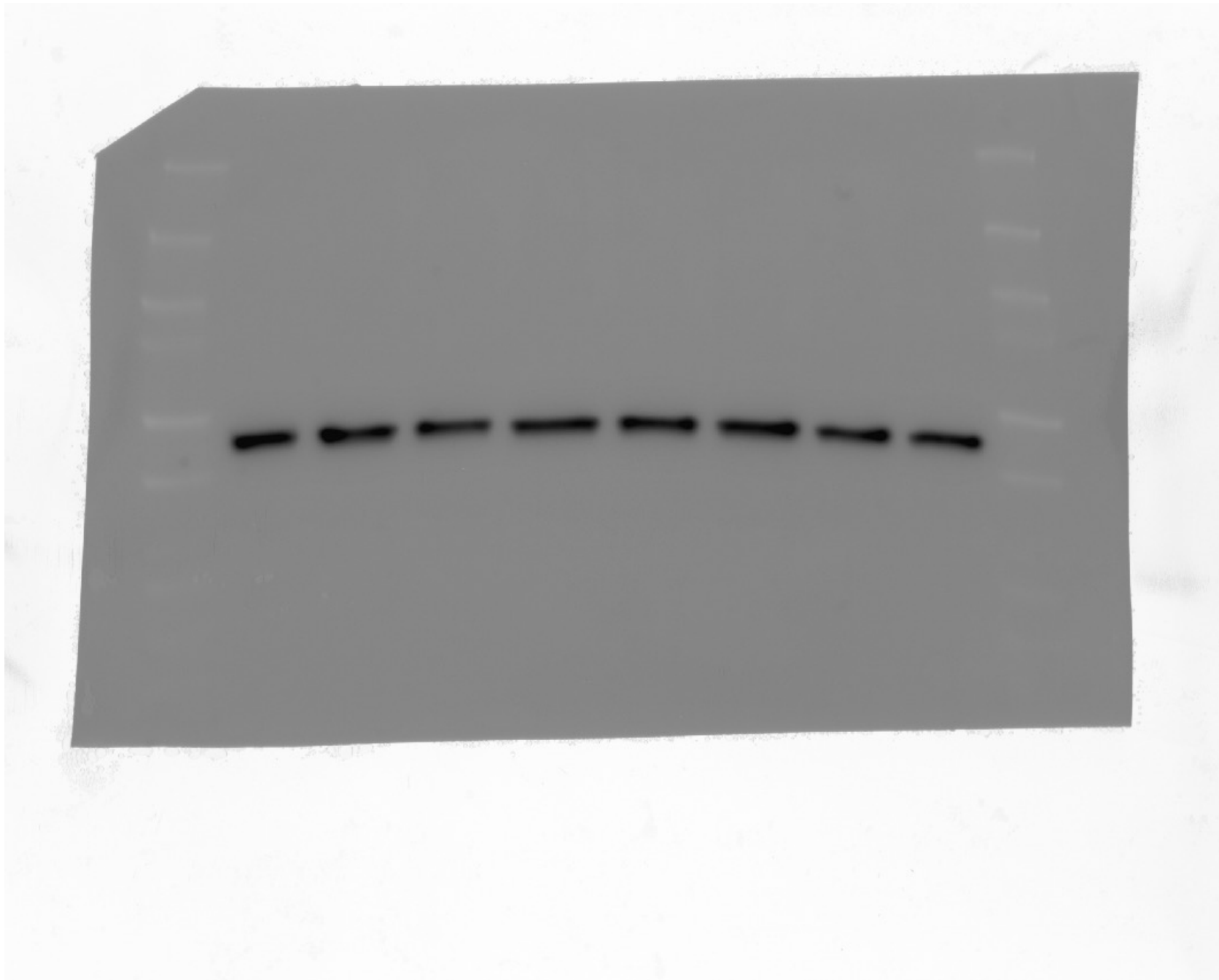

23-0509-2  
Phospho-Erk

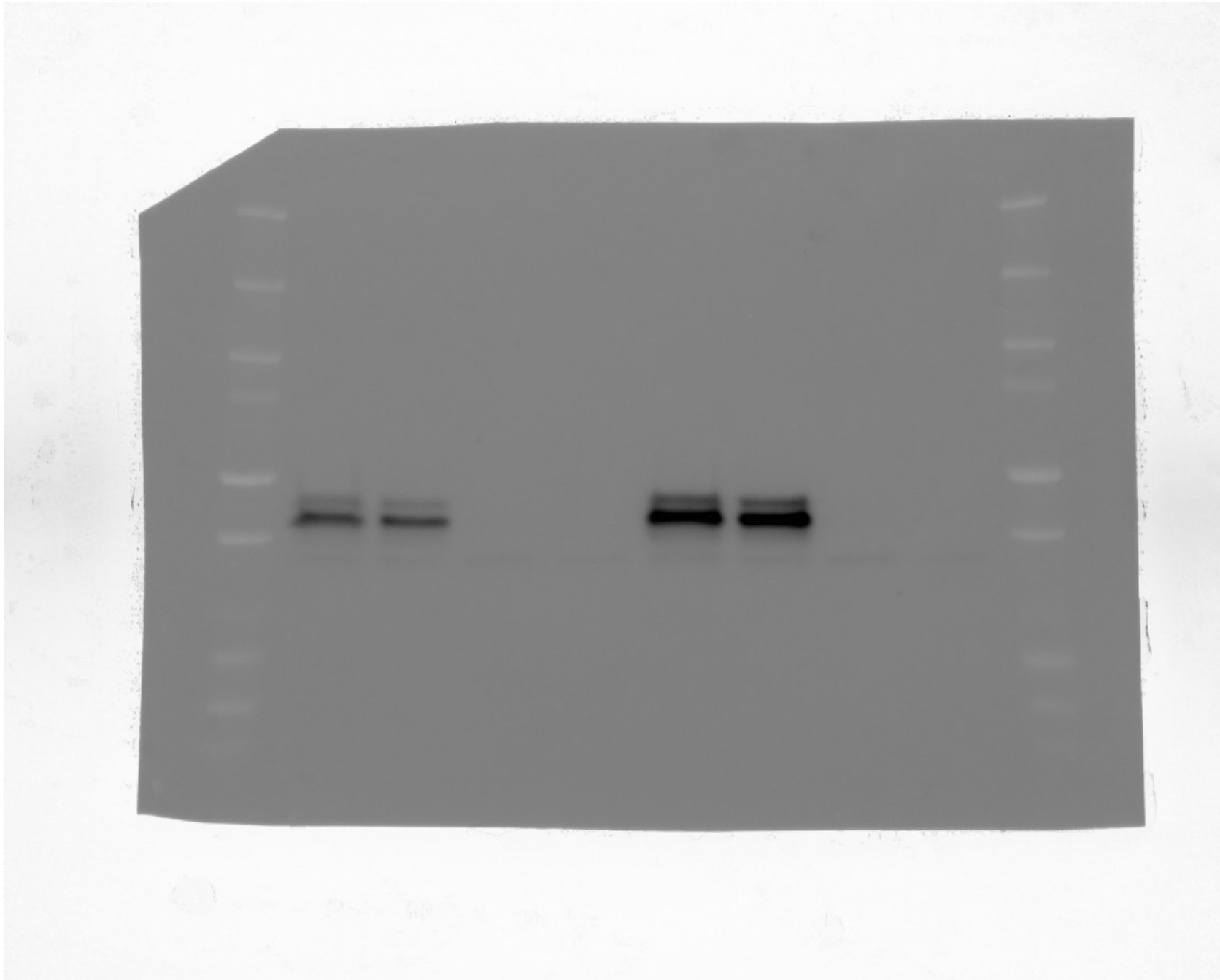

23-0509-2  
Total Erk

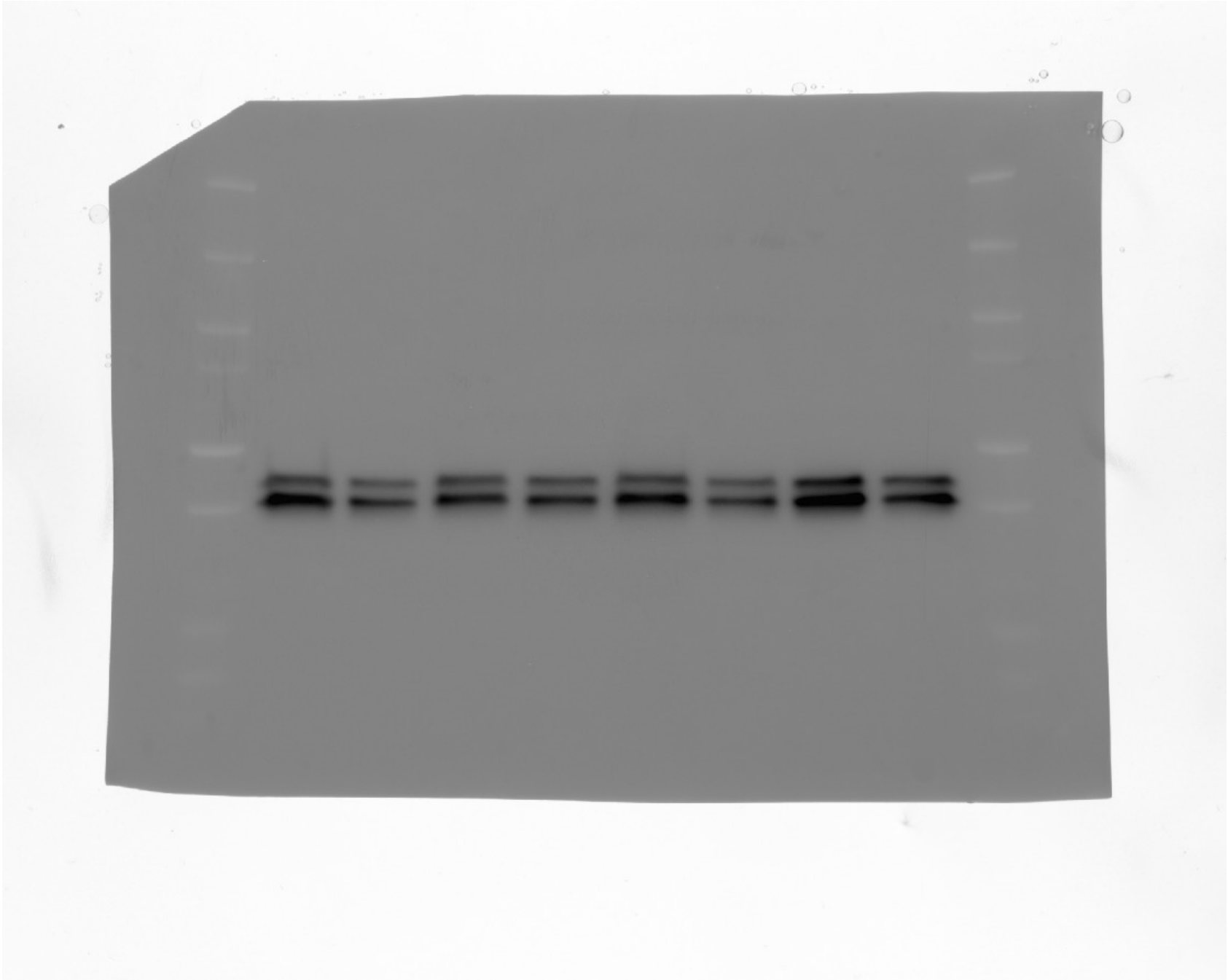

23-0509-2  
Actin

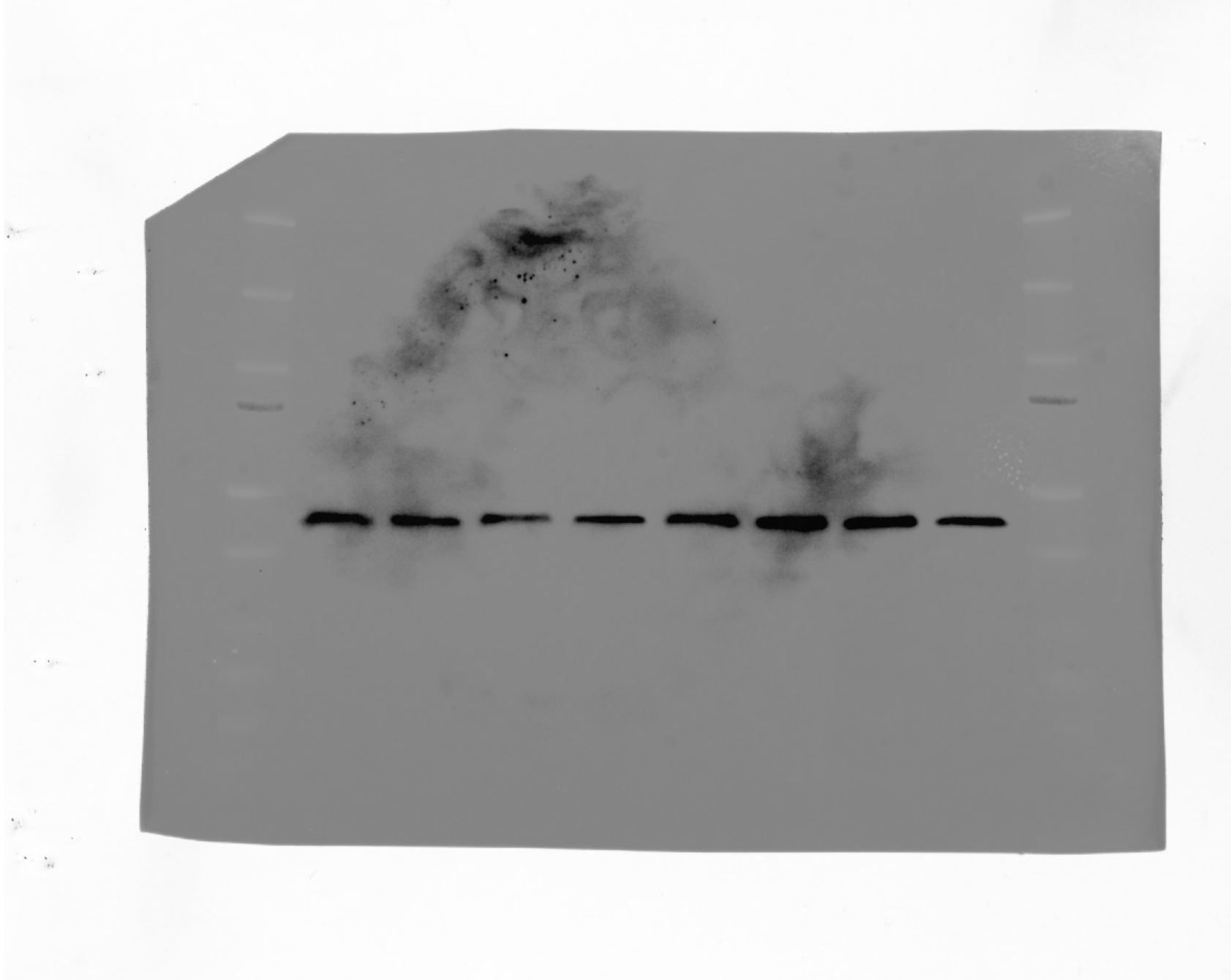

4738  
Phospho-Erk

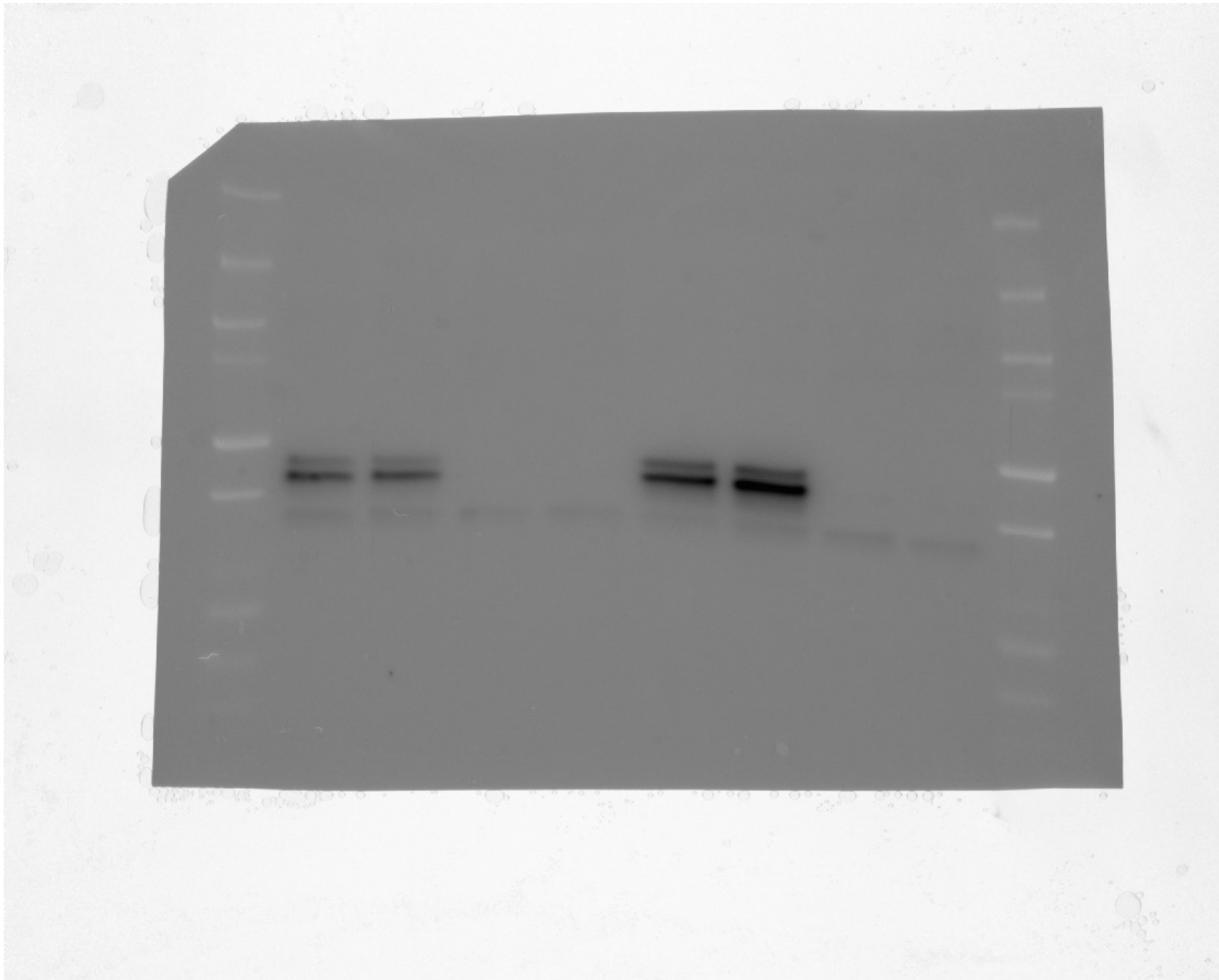

4738  
Total Erk

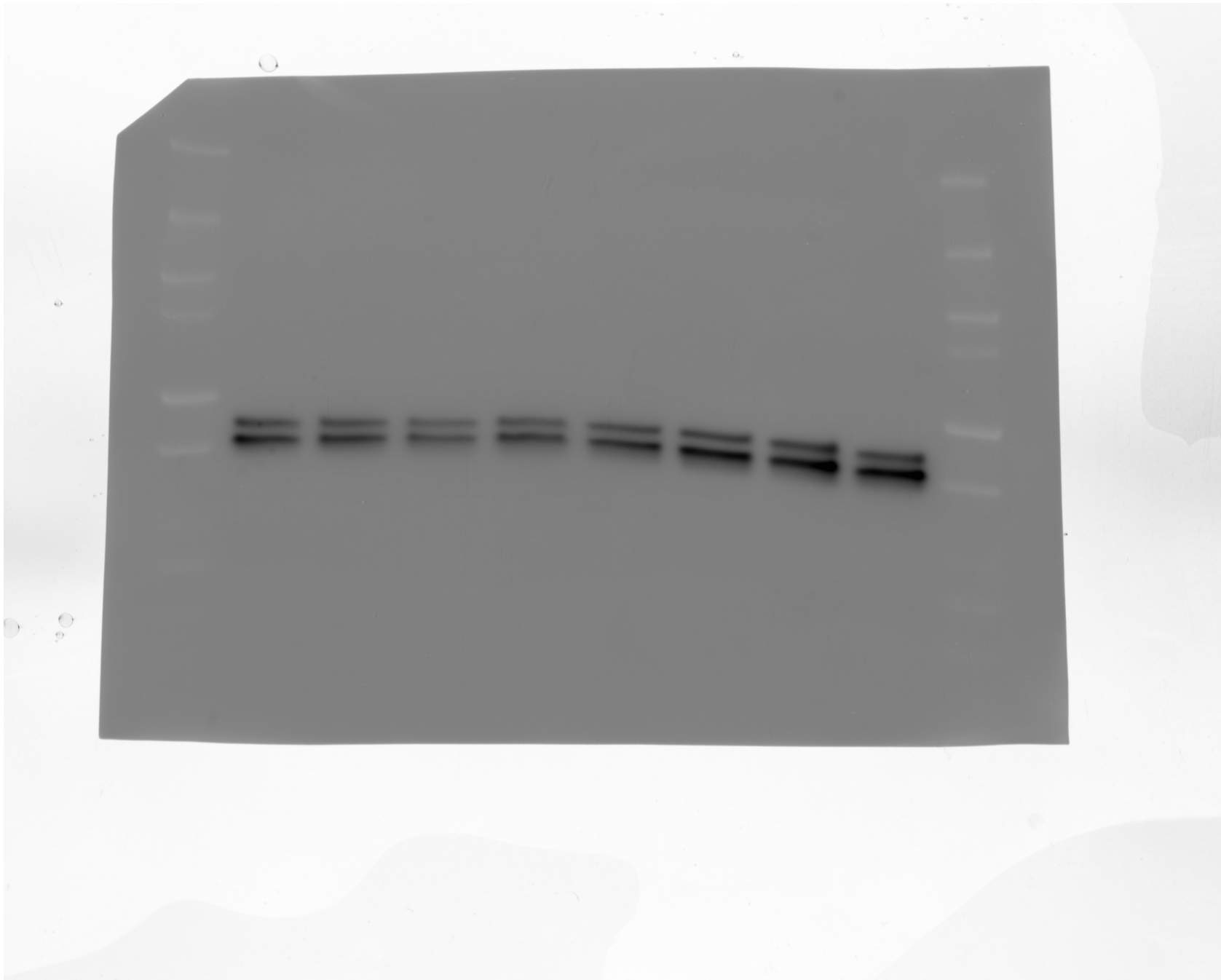

4738  
Actin

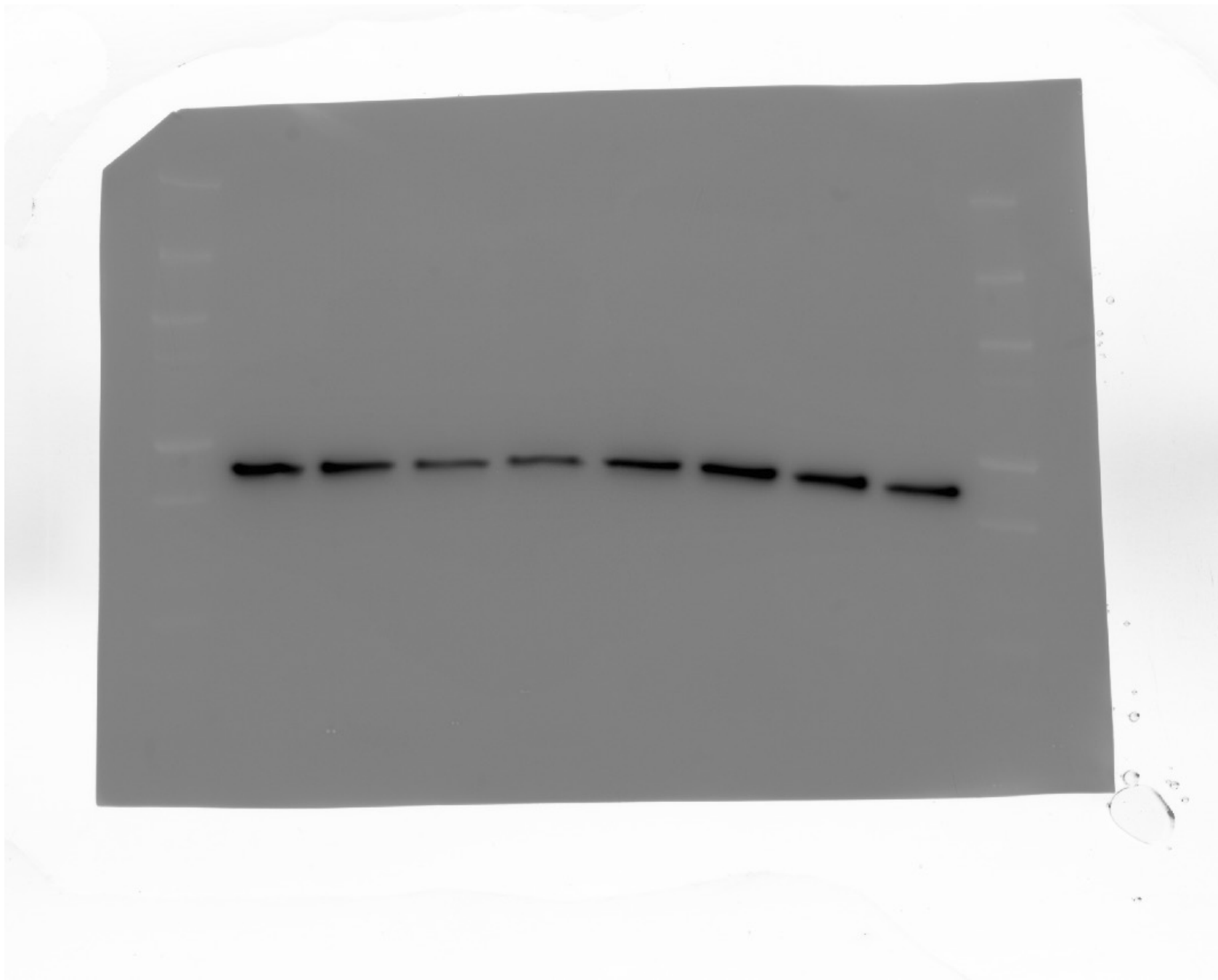

Supplement: S6 Fig — (PDF) [file pone.0323235.s007.pdf]
