# Supplementary material for: Effects of combination therapy of a CDK4/6 and MEK inhibitor in diffuse midline glioma preclinical models
Source: PLoS One. 2025 Dec 22;20(12):e0323235. doi: 10.1371/journal.pone.0323235 (PMC12721541; doi:10.1371/journal.pone.0323235)
Supplement: S1 Table — (DOCX) [file pone.0323235.s008.docx]

**Supplemental table 1. Background features of the mice with 21-day treatment**

|  |  | Vehicle | Ribociclib | Trametinib | Combination | p value |
| --- | --- | --- | --- | --- | --- | --- |
| Number of mice | | 17 | 18 | 18 | 18 | - |
| Sex | Female | 7 | 7 | 8 | 8 | 1.0000 |
|  | Male | 10 | 11 | 10 | 10 |  |
| Age at the time of randomization (days) |  | 50.4 | 50.2 | 50.2 | 50.2 | 0.9608 |
| Body weight at the time of randomization (g) |  | 19.3 | 19.4 | 19.3 | 19.3 | 0.9663 |
| Treatment completion |  | 10 | 12 | 11 | 15 | 0.4046 |
