# Supplementary material for: Effects of combination therapy of a CDK4/6 and MEK inhibitor in diffuse midline glioma preclinical models
Source: PLoS One. 2025 Dec 22;20(12):e0323235. doi: 10.1371/journal.pone.0323235 (PMC12721541; doi:10.1371/journal.pone.0323235)
Supplement: S2 Table — (DOCX) [file pone.0323235.s009.docx]

**Supplemental table 2. LC/MS analysis of ribociclib and trametinib in brain tumor**

| **Sample**  **number** | **Concentration of ribociclib (μM)** | | | | **Trametinib concentration (μM)** | | | |  |
| --- | --- | --- | --- | --- | --- | --- | --- | --- | --- |
|  | **Mouse DMG**  **treat with ribociclib** | **Mouse DMG**  **treat with combination** | **Human DMG treat with ribociclib** | **Human DMG treat with combination** | **Mouse DMG**  **treat with trametinib** | **Mouse DMG**  **treat with combination** | **Human DMG treat with trametinib** | **Human DMG treat with combination** | |
| 1 | 1.007804 | 0.037055 | 24.62374 | 1.43027 | 0.025187 | 0.002424 | 0.049359 | 0.029802 | |
| 2 | 0.246387 | 0.392219 | 3.72808 | 1.424495 | 0.053345 | 0.048479 | 0.020637 | 0.058012 | |
| 3 | 54.44354 | 0.213549 | 10.58937 | 2.149399 | 0.035256 | 0.037781 | 0.13399 | 0.034775 | |
| 4 | 22.63246 | 2.83825 | 1.933079 | 12.69674 |  | 0.03363 | 0.043225 | 0.108286 | |
| 5 |  | 2.588945 | 8.16219 | 1.41529 |  | 0.009435 | 0.026692 | 0.051025 | |
| 6 |  |  | 1.960694 | 0.024624 |  |  | 0.039975 | 0.001516 | |
| 7 |  |  | 0.230128 | 0.075942 |  |  | 0.001706 | 0.028112 | |
| 8 |  |  | 0.052469 | 0.013739 |  |  | 0.079624 | 0.039487 | |
| 9 |  |  | 0.01314 | 0.021034 |  |  | 0.04615 | 0.004924 | |
| 10 |  |  | 8.74488 |  |  |  | 0.003526 |  | |
| 11 |  |  | 1.155245 |  |  |  | 0.003266 |  | |
| 12 |  |  | 6.111607 |  |  |  | 0.015865 |  | |
| 13 |  |  | 1.673034 |  |  |  | 0.02015 |  | |
