# Supplementary material for: Effects of combination therapy of a CDK4/6 and MEK inhibitor in diffuse midline glioma preclinical models
Source: PLoS One. 2025 Dec 22;20(12):e0323235. doi: 10.1371/journal.pone.0323235 (PMC12721541; doi:10.1371/journal.pone.0323235)
Supplement: S3 Table — (DOCX) [file pone.0323235.s010.docx]

**Supplemental table 3. Background information of the DIPG-bearing mice used for RNAseq**

| **Treatment** | **Mouse ID** | **Sex** | **Survival (days)** | **Tumor location** |
| --- | --- | --- | --- | --- |
| Vehicle | Vehicle_1516F_S50 | Female | 71 | Ventral pons |
|  | Vehicle_1612M_S52 | Male | 73 | Ventral pons |
|  | Vehicle_1620M_S51 | Male | 73 | Ventral pons |
|  | Vehicle_1771M_S53 | Male | 72 | Ventral pons |
| Ribociclib | Ribociclib_1618M_ | Male | 74 | Ventral pons |
|  | Ribociclib_1613M | Male | 91 | Ventral pons |
|  | Ribociclib_1616F | Female | 103 | Ventral pons |
|  | Ribociclib_1801M | Male | 88 | Ventral pons |
| Trametinib | Trametinib_1396M | Male | 109 | Ventral pons |
|  | Trametinib_1469F | Female | 80 | Ventral pons |
|  | Trametinib_1811M | Male | 87 | Ventral pons |
| Combination | Combi_1621M_S61 | Male | 119 | Ventral pons |
|  | Combi_1802M_S62 | Male | 71 | Ventral pons |
|  | Combi_430M_S64 | Male | 177 | Ventral pons |
|  | Combi_561F_S63 | Female | 106 | Ventral pons |
