# Supplementary material for: Effects of combination therapy of a CDK4/6 and MEK inhibitor in diffuse midline glioma preclinical models
Source: PLoS One. 2025 Dec 22;20(12):e0323235. doi: 10.1371/journal.pone.0323235 (PMC12721541; doi:10.1371/journal.pone.0323235)
Supplement: S4 Table — (DOCX) [file pone.0323235.s011.docx]

**Supplemental table 4. Differentially expressed genes between tumors treated with ribociclib and those treated with vehicle**

| **Gene name** | **Gene ID** | **Base Mean** | **Vehicle_mean** | **Ribociclib_mean** | **fold change** | **log2 fold change** | **p Value** | **FDR Adj p Value** | **Vehicle_1516F_S50** | **Vehicle_1612M_S52** | **Vehicle_1620M_S51** | **Vehicle_1771M_S53** | **Ribociclib_1618M_** | **Ribociclib_1613M** | **Ribociclib_1616F** | **Ribociclib_1801M** |
| --- | --- | --- | --- | --- | --- | --- | --- | --- | --- | --- | --- | --- | --- | --- | --- | --- |
| Pcdhga7 | ENSMUSG00000103472 | 389.080203 | 676.782592 | 101.377815 | 0.36832391 | -1.440953 | 1.73E-10 | 2.81E-06 | 333.152932 | 1235.6737 | 732.7493 | 405.554438 | 152.211208 | 120.292762 | 85.340074 | 47.6672147 |
| Pde3a | ENSMUSG00000041741 | 785.332686 | 974.669567 | 595.995805 | 0.63175449 | -0.6625641 | 6.99E-09 | 5.32E-05 | 1005.2696 | 908.520505 | 1021.1216 | 963.766556 | 539.236047 | 658.07217 | 569.218293 | 617.456711 |
| Eya1 | ENSMUSG00000025932 | 1027.01387 | 622.463095 | 1431.56465 | 1.98531994 | 0.98937152 | 9.80E-09 | 5.32E-05 | 708.918448 | 678.976954 | 513.397251 | 588.559729 | 1143.44029 | 2059.12905 | 1251.08548 | 1272.60378 |
| Ankrd34c | ENSMUSG00000047606 | 90.7103233 | 163.144789 | 18.2758573 | 0.42720088 | -1.2270135 | 4.34E-08 | 0.00017694 | 253.73857 | 159.822379 | 131.422132 | 107.596075 | 16.7061082 | 7.07604483 | 42.670037 | 6.65123926 |
| En2 | ENSMUSG00000039095 | 305.478203 | 156.43608 | 454.520326 | 2.1077418 | 1.07569815 | 2.08E-07 | 0.00067764 | 170.450337 | 115.844409 | 172.0779 | 167.371673 | 400.018479 | 752.419434 | 331.972888 | 333.670503 |
| Kcnv1 | ENSMUSG00000022342 | 129.458104 | 180.859402 | 78.0568066 | 0.52900896 | -0.9186359 | 2.03E-06 | 0.00550981 | 165.607998 | 187.710848 | 217.461082 | 152.65768 | 98.3804148 | 61.3257219 | 68.2720592 | 84.2490306 |
| Tnfrsf11b | ENSMUSG00000063727 | 150.111766 | 197.939249 | 102.284283 | 0.58441426 | -0.7749367 | 9.65E-06 | 0.01964574 | 198.535904 | 214.526684 | 194.769491 | 183.924915 | 110.445937 | 77.8364932 | 106.675092 | 114.179607 |
| A2m | ENSMUSG00000030111 | 244.266803 | 369.849258 | 118.684349 | 0.5083316 | -0.9761582 | 1.09E-05 | 0.01975121 | 322.499786 | 388.293297 | 160.732104 | 607.871845 | 88.1711264 | 106.140673 | 170.680148 | 109.745448 |
| Cep170b | ENSMUSG00000072825 | 659.824063 | 496.546053 | 823.102072 | 1.56074435 | 0.64223425 | 1.27E-05 | 0.02076507 | 488.107784 | 540.607243 | 576.74461 | 380.724574 | 851.083399 | 727.653277 | 801.343294 | 912.328318 |
| Cyp26b1 | ENSMUSG00000063415 | 396.530908 | 234.509373 | 558.552442 | 1.84067242 | 0.8802329 | 2.07E-05 | 0.03061139 | 395.134873 | 151.241312 | 164.514036 | 227.14727 | 494.686425 | 482.350389 | 648.584562 | 608.588392 |
| Gria1 | ENSMUSG00000020524 | 158.515414 | 263.2949 | 53.7359292 | 0.51984554 | -0.9438451 | 2.59E-05 | 0.03515378 | 226.621471 | 179.129781 | 269.462646 | 377.965701 | 21.3466938 | 20.0487937 | 131.423714 | 42.1245153 |
| Wwp2 | ENSMUSG00000031930 | 2367.84074 | 1664.88857 | 3070.79291 | 1.65196392 | 0.72418218 | 3.17E-05 | 0.03831209 | 2090.92203 | 1741.95667 | 1403.09672 | 1423.57884 | 2801.98559 | 4048.67699 | 3305.22106 | 2127.28802 |
| Ccnd2 | ENSMUSG00000000184 | 10141.2364 | 12327.7217 | 7954.75119 | 0.67454787 | -0.5680073 | 3.29E-05 | 0.03831209 | 11665.195 | 14143.7443 | 11399.6881 | 12102.2594 | 6948.81288 | 9281.41214 | 6954.36263 | 8634.41709 |
