# Supplementary material for: Effects of combination therapy of a CDK4/6 and MEK inhibitor in diffuse midline glioma preclinical models
Source: PLoS One. 2025 Dec 22;20(12):e0323235. doi: 10.1371/journal.pone.0323235 (PMC12721541; doi:10.1371/journal.pone.0323235)
Supplement: S5 Table — (DOCX) [file pone.0323235.s012.docx]

**Supplemental table 5. Differentially expressed genes between tumors treated with trametinib and those treated with vehicle**

| **Gene name** | **Gene ID** | **Base Mean** | **Vehicle_mean** | **Trametinib_mean** | **fold change** | **log2 fold change** | **p Value** | **FDR Adj p Value** | **Vehicle_1516F_S50** | **Vehicle_1612M_S52** | **Vehicle_1620M_S51** | **Vehicle_1771M_S53** | **Trametinib_1396M** | **Trametinib_1469F** | **Trametinib_1811M** |
| --- | --- | --- | --- | --- | --- | --- | --- | --- | --- | --- | --- | --- | --- | --- | --- |
| Col9a1 | ENSMUSG00000026147 | 1066.17994 | 337.238328 | 2038.10209 | 4.75982307 | 2.25090795 | 8.41E-29 | 1.37E-24 | 326.932324 | 276.304514 | 381.272658 | 364.443816 | 2313.31325 | 2473.26291 | 1327.73013 |
| Ankrd34c | ENSMUSG00000047606 | 102.138073 | 166.082914 | 16.878286 | 0.30589022 | -1.7089141 | 6.60E-09 | 5.39E-05 | 258.780269 | 162.724793 | 133.49345 | 109.333145 | 2.92700959 | 17.2553226 | 30.4525259 |
| Cdh15 | ENSMUSG00000031962 | 173.297673 | 49.6198573 | 338.201428 | 3.11652487 | 1.63993823 | 1.90E-08 | 0.00010347 | 55.3118132 | 37.131832 | 16.326537 | 89.7092469 | 382.462586 | 469.728226 | 162.413471 |
| Dbh | ENSMUSG00000000889 | 18.2919423 | 30.9958149 | 1.3534456 | 0.33045366 | -1.5974801 | 7.00E-08 | 0.0002326 | 35.5575942 | 24.0264795 | 31.6926895 | 32.7064963 | 0 | 0 | 4.06033679 |
| Cxxc5 | ENSMUSG00000046668 | 3281.61561 | 4085.90115 | 2209.2349 | 0.56503114 | -0.8235977 | 7.17E-08 | 0.0002326 | 4641.25375 | 4276.71335 | 4133.49502 | 3292.14247 | 2346.48602 | 1937.38928 | 2343.82941 |
| Thbs4 | ENSMUSG00000021702 | 558.615133 | 247.597511 | 973.305295 | 2.66740587 | 1.41543736 | 1.34E-07 | 0.00029761 | 306.190394 | 219.514654 | 147.899218 | 316.785778 | 513.202348 | 1672.80766 | 733.905874 |
| Smpdl3b | ENSMUSG00000028885 | 235.763837 | 86.1225578 | 435.285542 | 2.82695056 | 1.49924665 | 1.46E-07 | 0.00029761 | 79.0168759 | 50.2371845 | 80.6723005 | 134.56387 | 210.74469 | 795.662098 | 299.449838 |
| En2 | ENSMUSG00000039095 | 291.574519 | 159.162266 | 468.124188 | 2.35847833 | 1.23785635 | 2.51E-07 | 0.00045426 | 173.837127 | 117.948172 | 174.789984 | 170.073781 | 338.557443 | 362.361775 | 703.453348 |
| Tbx4 | ENSMUSG00000000094 | 106.689444 | 19.7511174 | 222.607213 | 2.84210531 | 1.50696001 | 3.35E-07 | 0.00054727 | 11.8525314 | 15.2895779 | 17.2869215 | 34.5754389 | 47.8078233 | 520.535565 | 99.4782512 |
| Rab6b | ENSMUSG00000032549 | 2579.15285 | 3143.14315 | 1827.16579 | 0.60179063 | -0.7326665 | 4.36E-07 | 0.0006462 | 3085.60901 | 3338.58854 | 3481.39392 | 2666.98115 | 1878.16449 | 1606.66226 | 1996.67061 |
| Ret | ENSMUSG00000030110 | 301.685581 | 457.691284 | 93.6779771 | 0.38233445 | -1.3870929 | 1.42E-06 | 0.00170466 | 622.257898 | 246.817471 | 800.960698 | 160.729067 | 105.372345 | 68.0626614 | 107.598925 |
| Sft2d2 | ENSMUSG00000040848 | 781.409875 | 555.319529 | 1082.86367 | 1.82256577 | 0.86597087 | 1.46E-06 | 0.00170466 | 552.130421 | 654.175511 | 420.648424 | 594.323761 | 1095.67726 | 1242.38323 | 910.530524 |
| Sytl2 | ENSMUSG00000030616 | 521.321933 | 346.143212 | 754.893562 | 1.96105055 | 0.97162672 | 1.87E-06 | 0.0020343 | 256.804847 | 288.317754 | 357.263045 | 482.187202 | 795.170939 | 781.282662 | 688.227085 |
| Pnlip | ENSMUSG00000046008 | 5511.9902 | 6748.56313 | 3863.22629 | 0.59744809 | -0.7431147 | 2.47E-06 | 0.00251647 | 6342.09201 | 7870.85627 | 5734.45603 | 7046.84824 | 3288.00744 | 3907.37194 | 4394.29949 |
| Cxcl14 | ENSMUSG00000021508 | 2294.37608 | 2705.05662 | 1746.80202 | 0.65974191 | -0.6000263 | 2.66E-06 | 0.00255734 | 2702.37716 | 2998.94149 | 2731.3336 | 2387.57423 | 1684.00618 | 1817.56065 | 1738.83923 |
| Plxna4 | ENSMUSG00000029765 | 376.329023 | 468.756254 | 253.092717 | 0.57294422 | -0.8035334 | 4.18E-06 | 0.00378903 | 454.347037 | 519.845648 | 519.568031 | 381.264299 | 256.601174 | 237.74 | 264.936975 |
| Fbxo17 | ENSMUSG00000030598 | 223.311525 | 157.080512 | 311.619541 | 1.83090228 | 0.87255479 | 5.28E-06 | 0.00453215 | 179.763393 | 160.540568 | 145.978449 | 142.039641 | 268.309212 | 350.858226 | 315.691185 |
| Pcdhb7 | ENSMUSG00000045062 | 568.217867 | 759.132511 | 313.665008 | 0.48681939 | -1.0385414 | 6.26E-06 | 0.00510987 | 775.353095 | 851.84791 | 767.347239 | 641.981798 | 360.997849 | 165.842823 | 414.154352 |
| Dtna | ENSMUSG00000024302 | 1950.86695 | 2379.07901 | 1379.91754 | 0.60576702 | -0.7231651 | 6.93E-06 | 0.0053827 | 2323.09615 | 2423.39809 | 2365.4271 | 2404.39471 | 1223.49001 | 1156.10661 | 1760.156 |
| Fstl5 | ENSMUSG00000034098 | 558.405928 | 787.232288 | 253.304114 | 0.4409457 | -1.1813271 | 7.75E-06 | 0.00572105 | 587.688015 | 1311.62736 | 735.65455 | 513.959227 | 137.569451 | 256.912581 | 365.430311 |
| Hacd3 | ENSMUSG00000033629 | 3770.55561 | 4352.17269 | 2995.06618 | 0.69846913 | -0.5177317 | 8.07E-06 | 0.00572105 | 4433.83445 | 4428.51702 | 4455.22383 | 4091.11545 | 2921.15557 | 2845.21097 | 3218.83199 |
| Cyp26b1 | ENSMUSG00000063415 | 409.4414 | 238.723821 | 637.064839 | 2.14362815 | 1.10005467 | 9.14E-06 | 0.00621096 | 402.986067 | 153.987891 | 167.106908 | 230.814417 | 693.701273 | 682.543872 | 534.949371 |
| Pld5 | ENSMUSG00000055214 | 137.825349 | 82.2522783 | 211.922777 | 2.10036581 | 1.07064062 | 1.19E-05 | 0.0077422 | 86.9185635 | 53.5135226 | 96.9988375 | 91.5781896 | 168.790886 | 275.126533 | 191.850913 |
| Ptprn | ENSMUSG00000026204 | 5567.79375 | 7672.4275 | 2761.61542 | 0.46302384 | -1.1108416 | 1.27E-05 | 0.00796672 | 10923.0954 | 5573.05114 | 7129.89475 | 7063.66872 | 2406.97755 | 1387.13621 | 4490.73248 |
| Epb41 | ENSMUSG00000028906 | 2129.48484 | 1655.42763 | 2761.56112 | 1.60506839 | 0.68263477 | 1.36E-05 | 0.00821609 | 1330.44665 | 1649.09019 | 1820.88907 | 1821.28461 | 3144.58397 | 2711.00291 | 2429.09648 |
| Tagln3 | ENSMUSG00000022658 | 1592.11469 | 1973.4739 | 1083.63575 | 0.58388468 | -0.7762446 | 1.58E-05 | 0.00889052 | 1958.63081 | 2048.80344 | 2354.86287 | 1531.5985 | 1198.12259 | 839.759033 | 1213.02561 |
| Rcan1 | ENSMUSG00000022951 | 1222.23108 | 1458.19663 | 907.610332 | 0.64255959 | -0.6380978 | 1.69E-05 | 0.00889052 | 1564.53414 | 1578.10286 | 1378.1518 | 1311.99774 | 1032.25872 | 785.117179 | 905.455103 |
| Tspan13 | ENSMUSG00000020577 | 3143.88427 | 3867.49611 | 2179.06848 | 0.59514979 | -0.7486753 | 1.73E-05 | 0.00889052 | 4065.41827 | 4420.87223 | 3927.97273 | 3055.72122 | 1780.5975 | 2069.68008 | 2686.92787 |
| Rasgrf1 | ENSMUSG00000032356 | 1225.38369 | 1553.72995 | 787.588675 | 0.55346496 | -0.8534361 | 1.75E-05 | 0.00889052 | 1430.20545 | 1673.11666 | 1343.57796 | 1768.01974 | 544.423784 | 1063.1196 | 755.222642 |
| Diras2 | ENSMUSG00000047842 | 381.647011 | 513.641715 | 205.654072 | 0.48684326 | -1.0384707 | 1.79E-05 | 0.00889052 | 468.17499 | 785.229035 | 358.22343 | 442.939407 | 228.306748 | 139.959839 | 248.695628 |
| Hdhd2 | ENSMUSG00000025421 | 1724.93789 | 2063.461 | 1273.57374 | 0.63830637 | -0.647679 | 1.80E-05 | 0.00889052 | 2127.52938 | 2337.12119 | 1901.56137 | 1887.63207 | 1232.27104 | 1103.38202 | 1485.06818 |
| Fgfr3 | ENSMUSG00000054252 | 1235.91684 | 833.20769 | 1772.86237 | 1.89370604 | 0.9212124 | 1.87E-05 | 0.00891564 | 739.795501 | 1064.80989 | 533.013414 | 995.211958 | 2000.12322 | 1996.82428 | 1321.63962 |
| Rtn1 | ENSMUSG00000021087 | 10550.3995 | 13456.5369 | 6675.54958 | 0.54631285 | -0.8722007 | 1.91E-05 | 0.00891564 | 16117.4673 | 15473.0528 | 12671.3135 | 9564.31398 | 5413.99207 | 5955.00356 | 8657.65311 |
| Trim9 | ENSMUSG00000021071 | 4141.07958 | 5236.75594 | 2680.17776 | 0.55742646 | -0.8431466 | 1.98E-05 | 0.00897945 | 7549.07479 | 4746.32182 | 4928.69341 | 3722.93375 | 2491.86083 | 2685.11992 | 2863.55252 |
| Nrxn3 | ENSMUSG00000066392 | 1289.15817 | 1744.87305 | 681.538335 | 0.48324087 | -1.0491856 | 2.21E-05 | 0.00965201 | 2263.8335 | 1866.42061 | 1490.51679 | 1358.7213 | 546.375123 | 379.617097 | 1118.62278 |
| Daam2 | ENSMUSG00000040260 | 719.946978 | 492.369388 | 1023.38376 | 1.8624937 | 0.89723555 | 2.41E-05 | 0.00993875 | 445.457638 | 444.489871 | 388.955735 | 690.574307 | 1143.48508 | 1188.7 | 737.966211 |
| Gng3 | ENSMUSG00000071658 | 5031.31762 | 6127.51461 | 3569.72162 | 0.61113966 | -0.710426 | 2.48E-05 | 0.00993875 | 6776.68482 | 6396.50412 | 6332.77559 | 5004.09393 | 2862.61538 | 4309.99614 | 3536.55334 |
| Rbp1 | ENSMUSG00000046402 | 1243.54948 | 1632.77707 | 724.579352 | 0.51469536 | -0.9582093 | 2.72E-05 | 0.01058121 | 1411.43895 | 2416.84542 | 1224.49028 | 1478.33363 | 1022.50202 | 601.060404 | 550.175634 |
| Lect1 | ENSMUSG00000022025 | 29.0819768 | 11.2296633 | 52.8850614 | 2.33994804 | 1.22647649 | 3.29E-05 | 0.01250043 | 11.8525314 | 13.1053525 | 12.4849989 | 7.47577058 | 88.7859576 | 37.3865323 | 32.4826943 |
| 4930520O04Rik | ENSMUSG00000074039 | 46.2843601 | 21.0343018 | 79.9511046 | 2.29002568 | 1.19536378 | 3.38E-05 | 0.01252097 | 17.7787971 | 21.8422541 | 22.0888442 | 22.4273117 | 49.759163 | 133.249436 | 56.844715 |
| Igip | ENSMUSG00000110185 | 668.621029 | 863.772888 | 408.41855 | 0.53409015 | -0.9048448 | 3.47E-05 | 0.01256767 | 763.500564 | 1039.6913 | 822.089157 | 829.810534 | 347.338471 | 278.00242 | 599.91476 |
| Wnk4 | ENSMUSG00000035112 | 690.070237 | 364.934274 | 1123.58485 | 2.18765556 | 1.1293856 | 3.74E-05 | 0.01314279 | 514.597405 | 230.435781 | 236.254594 | 478.449317 | 1037.13706 | 1828.10557 | 505.51193 |
| Pla2g3 | ENSMUSG00000034579 | 98.1502892 | 48.4337772 | 164.438972 | 2.2375575 | 1.16192476 | 3.79E-05 | 0.01314279 | 17.7787971 | 55.697748 | 58.5834563 | 61.6751073 | 171.717896 | 209.939758 | 111.659262 |
| Aim1 | ENSMUSG00000019866 | 254.698165 | 117.40365 | 437.757518 | 2.27413258 | 1.18531636 | 3.90E-05 | 0.01324539 | 69.1397664 | 196.580287 | 41.2965348 | 162.59801 | 590.280267 | 461.100565 | 261.891723 |
| Btbd1 | ENSMUSG00000025103 | 1417.18649 | 1620.11047 | 1146.62118 | 0.71797882 | -0.4779868 | 5.15E-05 | 0.01713306 | 1589.22692 | 1737.55131 | 1586.55524 | 1567.10841 | 1178.60919 | 1109.13379 | 1152.12056 |
| Crkl | ENSMUSG00000006134 | 3376.95801 | 3965.79784 | 2591.83825 | 0.67089737 | -0.575836 | 5.40E-05 | 0.01763495 | 4309.38287 | 4387.01674 | 3510.20546 | 3656.58628 | 2608.94121 | 2313.17186 | 2853.40168 |
| Cdk5rap2 | ENSMUSG00000076432 | 1199.7536 | 1431.93264 | 890.181543 | 0.64471105 | -0.6332754 | 6.10E-05 | 0.0195279 | 1301.80303 | 1455.78624 | 1642.25755 | 1327.88375 | 918.105341 | 734.30984 | 1018.12945 |
| Ywhaq | ENSMUSG00000076432 | 1199.7536 | 1431.93264 | 890.181543 | 0.64471105 | -0.6332754 | 6.10E-05 | 0.0195279 | 1301.80303 | 1455.78624 | 1642.25755 | 1327.88375 | 918.105341 | 734.30984 | 1018.12945 |
| Eps8l1 | ENSMUSG00000006154 | 221.738182 | 324.459414 | 84.7765407 | 0.4485534 | -1.1566484 | 6.41E-05 | 0.01974654 | 594.601991 | 199.856625 | 335.174201 | 168.204838 | 132.691101 | 37.3865323 | 84.2519883 |
| Adam23 | ENSMUSG00000025964 | 2330.93156 | 1393.79316 | 3580.44942 | 2.03617538 | 1.02586183 | 6.41E-05 | 0.01974654 | 2063.32817 | 537.319451 | 1146.69913 | 1827.82591 | 3631.44323 | 3925.58589 | 3184.31912 |
| Mro | ENSMUSG00000064036 | 1374.92881 | 1699.27854 | 942.462506 | 0.59345246 | -0.7527956 | 6.76E-05 | 0.02039709 | 1771.95344 | 2084.84315 | 1610.56486 | 1329.75269 | 870.297518 | 758.275566 | 1198.81444 |
| Zfp397 | ENSMUSG00000024276 | 1382.75215 | 1646.72966 | 1030.78214 | 0.64866945 | -0.6244446 | 7.38E-05 | 0.02149125 | 1654.41584 | 1642.53751 | 1616.32716 | 1673.63814 | 830.295054 | 992.18105 | 1269.87033 |
| Eya1 | ENSMUSG00000025932 | 899.973336 | 633.465551 | 1255.31705 | 1.78937681 | 0.83945723 | 7.61E-05 | 0.02176957 | 723.004415 | 691.307343 | 521.4888 | 598.061646 | 1742.54638 | 821.545082 | 1201.85969 |
| Npw | ENSMUSG00000071230 | 27.8992043 | 7.48299603 | 55.1208152 | 2.22730986 | 1.15530228 | 7.85E-05 | 0.02206987 | 11.8525314 | 7.64478894 | 5.76230718 | 4.67235661 | 27.3187562 | 120.787258 | 17.2564313 |
| Adgra1 | ENSMUSG00000025475 | 649.814716 | 884.909395 | 336.355145 | 0.49101529 | -1.0261601 | 8.19E-05 | 0.02266052 | 945.239378 | 1273.40341 | 783.673776 | 537.32101 | 328.800744 | 169.677339 | 510.587351 |
| Tmem255a | ENSMUSG00000036502 | 1947.0299 | 2580.95025 | 1101.80277 | 0.51405354 | -0.9600095 | 8.46E-05 | 0.02298129 | 2939.42778 | 3575.577 | 1796.87946 | 2011.91676 | 761.998163 | 816.751937 | 1726.65822 |
| Reep2 | ENSMUSG00000038555 | 1244.43896 | 1508.78224 | 891.981258 | 0.62171386 | -0.6856774 | 8.59E-05 | 0.02298129 | 1431.19317 | 1602.12934 | 1707.56369 | 1294.24278 | 978.596873 | 670.081694 | 1027.26521 |
| Prrg4 | ENSMUSG00000027171 | 34.2379339 | 8.89894574 | 68.0232515 | 2.20545206 | 1.1410744 | 8.89E-05 | 0.02339741 | 2.96313285 | 17.4738033 | 7.68307624 | 7.47577058 | 35.1241151 | 147.628871 | 21.3167681 |
| Cnih2 | ENSMUSG00000024873 | 733.961802 | 960.130384 | 432.403693 | 0.52803217 | -0.9213023 | 9.72E-05 | 0.02517979 | 788.193338 | 1195.86341 | 957.503376 | 898.961412 | 512.226678 | 218.56742 | 566.416982 |
| Xkr5 | ENSMUSG00000039814 | 177.42723 | 126.817047 | 244.907474 | 1.75698683 | 0.81310338 | 9.98E-05 | 0.02544616 | 122.476158 | 136.514088 | 127.731142 | 120.546801 | 187.328614 | 288.547339 | 258.84647 |
| Crem | ENSMUSG00000063889 | 378.289459 | 458.772337 | 270.978955 | 0.6219831 | -0.6850527 | 0.00010206 | 0.02561802 | 559.044397 | 444.489871 | 445.618422 | 385.936656 | 280.992921 | 257.87121 | 274.072733 |
| Wwp2 | ENSMUSG00000031930 | 2691.22877 | 1694.45781 | 4020.25671 | 1.95132047 | 0.96445073 | 0.00010912 | 0.02697346 | 2132.46794 | 1773.59103 | 1425.21064 | 1446.56161 | 6959.45313 | 2456.00758 | 2645.30942 |
| Nsg2 | ENSMUSG00000020297 | 6560.53676 | 7849.81825 | 4841.49478 | 0.64262638 | -0.6379479 | 0.00011465 | 0.02737932 | 8461.7197 | 8653.90108 | 7905.88545 | 6377.76677 | 3894.87409 | 5469.93727 | 5159.67297 |
| Rxrg | ENSMUSG00000015843 | 332.156455 | 197.874662 | 511.198847 | 2.01371946 | 1.00986271 | 0.00011747 | 0.02737932 | 135.3164 | 304.699445 | 177.671138 | 173.811666 | 790.292589 | 305.802662 | 437.501289 |
| Gabra3 | ENSMUSG00000031343 | 1569.32695 | 2038.63449 | 943.583575 | 0.5369216 | -0.8972166 | 0.00012084 | 0.02737932 | 2120.61541 | 2642.91275 | 1793.9983 | 1597.01149 | 868.346178 | 547.377178 | 1415.02737 |
| Sema3c | ENSMUSG00000028780 | 75.3566782 | 29.4761064 | 136.530774 | 2.20537893 | 1.14102656 | 0.000121 | 0.02737932 | 28.6436175 | 26.2107049 | 16.326537 | 46.7235661 | 98.5426562 | 268.416129 | 42.6335362 |
| Fem1c | ENSMUSG00000033319 | 1061.13326 | 1269.42261 | 783.41413 | 0.64304986 | -0.6369975 | 0.00012135 | 0.02737932 | 1439.09485 | 1313.81158 | 1101.56106 | 1223.22296 | 669.309526 | 724.723549 | 956.209313 |
| Hspa2 | ENSMUSG00000059970 | 643.941261 | 770.210631 | 475.5821 | 0.64333542 | -0.636357 | 0.00012251 | 0.02737932 | 941.288535 | 737.176076 | 648.259558 | 754.118357 | 464.418855 | 447.679759 | 514.647688 |
| Fam49a | ENSMUSG00000020589 | 2054.86359 | 2588.0647 | 1343.92878 | 0.57194867 | -0.8060424 | 0.00012418 | 0.02737932 | 2568.04847 | 3099.41586 | 2157.98404 | 2526.81045 | 968.840174 | 1095.71299 | 1967.23317 |
| Slc12a2 | ENSMUSG00000024597 | 3494.60734 | 4139.67464 | 2634.5176 | 0.65859427 | -0.6025381 | 0.00012613 | 0.02743781 | 4249.1325 | 4241.76575 | 3586.07583 | 4481.72446 | 2280.14047 | 2398.48984 | 3224.92249 |
| Kcnd3 | ENSMUSG00000040896 | 3174.7746 | 3981.33676 | 2099.35839 | 0.57754076 | -0.7920053 | 0.00012931 | 0.02775852 | 3524.15267 | 5286.91761 | 3572.63045 | 3541.64631 | 1674.24949 | 1687.1871 | 2936.63858 |
| Hrk | ENSMUSG00000046607 | 64.7448686 | 36.6038684 | 102.266202 | 2.05739889 | 1.04082153 | 0.00013324 | 0.02797432 | 46.4224146 | 48.052959 | 20.1680751 | 31.7720249 | 118.056053 | 104.490565 | 84.2519883 |
| Sprn | ENSMUSG00000045733 | 1146.01505 | 1398.26835 | 809.677322 | 0.61434709 | -0.7028741 | 0.00013374 | 0.02797432 | 1237.60182 | 1890.44709 | 1277.31142 | 1187.71305 | 734.679407 | 755.399678 | 938.952882 |
| Ccl25 | ENSMUSG00000023235 | 346.436776 | 262.067739 | 458.928826 | 1.64400042 | 0.71721067 | 0.00013742 | 0.02837888 | 233.099784 | 261.014937 | 242.977286 | 311.17895 | 458.564836 | 541.625404 | 376.596237 |
| 4930550C14Rik | ENSMUSG00000005131 | 20.172767 | 7.76864867 | 36.7115915 | 2.18771159 | 1.12942256 | 0.00014261 | 0.02874498 | 7.90168759 | 10.9211271 | 3.84153812 | 8.4102419 | 29.2700959 | 57.517742 | 23.3469365 |
| Fzd6 | ENSMUSG00000022297 | 409.351397 | 318.077331 | 531.050152 | 1.58942275 | 0.6685029 | 0.00014271 | 0.02874498 | 352.612809 | 349.476066 | 318.847664 | 251.372786 | 551.253473 | 475.480001 | 566.416982 |
| Gramd2 | ENSMUSG00000074259 | 43.017224 | 23.2484278 | 69.3756191 | 2.07843174 | 1.05549537 | 0.00016084 | 0.03200095 | 18.766508 | 24.0264795 | 24.9699978 | 25.2307257 | 89.7616274 | 75.7316937 | 42.6335362 |
| Galnt12 | ENSMUSG00000039774 | 76.1052959 | 42.8028646 | 120.508538 | 2.04684953 | 1.03340505 | 0.00016703 | 0.03283163 | 23.7050628 | 40.4081701 | 55.7023027 | 51.3959227 | 164.888207 | 108.325081 | 88.3123251 |
| Wnt7b | ENSMUSG00000022382 | 2054.0905 | 2355.85599 | 1651.73651 | 0.71382219 | -0.4863633 | 0.00017991 | 0.03485825 | 2544.34341 | 2349.13443 | 2410.56517 | 2119.38096 | 1540.58271 | 1811.80887 | 1602.81795 |
| Nap1l5 | ENSMUSG00000055430 | 6677.45155 | 8259.01514 | 4568.7001 | 0.59712641 | -0.7438917 | 0.00018161 | 0.03485825 | 8092.31581 | 9322.27405 | 8620.41154 | 7001.05914 | 2916.27722 | 5490.06848 | 5299.75459 |
| Vsnl1 | ENSMUSG00000054459 | 243.171106 | 320.637801 | 139.882181 | 0.52666239 | -0.9250497 | 0.00018661 | 0.03540198 | 233.099784 | 422.647617 | 407.203041 | 219.600761 | 115.129044 | 120.787258 | 183.73024 |
| Reep5 | ENSMUSG00000005873 | 4762.67216 | 5752.16433 | 3443.34926 | 0.63037261 | -0.6657232 | 0.00019169 | 0.03594719 | 5895.64666 | 6688.09821 | 5574.07181 | 4850.84063 | 3262.64002 | 2682.24404 | 4385.16373 |
| Gfpt2 | ENSMUSG00000020363 | 581.117302 | 426.144944 | 787.747111 | 1.69814663 | 0.76396104 | 0.00020276 | 0.03754894 | 483.978365 | 485.990154 | 281.392667 | 453.218591 | 849.808451 | 897.276775 | 616.156107 |
| Otub2 | ENSMUSG00000021203 | 504.152336 | 590.555661 | 388.947903 | 0.67790464 | -0.5608457 | 0.00020687 | 0.03754894 | 651.889227 | 532.951 | 552.221105 | 625.161314 | 377.584237 | 387.28613 | 401.973342 |
| Cd200 | ENSMUSG00000022661 | 2919.24403 | 3713.96344 | 1859.61815 | 0.56359777 | -0.8272622 | 0.00020713 | 0.03754894 | 4701.50412 | 3710.99897 | 3608.16468 | 2835.18599 | 1751.3274 | 1143.64444 | 2683.88262 |
| Gpr150 | ENSMUSG00000045509 | 45.0928448 | 27.5099647 | 68.536685 | 1.95859411 | 0.96981845 | 0.00021333 | 0.03824768 | 26.6681956 | 28.3949303 | 28.8115359 | 26.165197 | 58.5401918 | 70.9385485 | 76.1313147 |
| Txndc15 | ENSMUSG00000021497 | 1715.16943 | 1961.09277 | 1387.27164 | 0.71959987 | -0.4747332 | 0.00021631 | 0.0383592 | 2033.69684 | 2130.71189 | 1939.97675 | 1739.9856 | 1403.01326 | 1313.32178 | 1445.4799 |
| Car4 | ENSMUSG00000000805 | 61.7334877 | 39.780787 | 91.0037552 | 1.89526038 | 0.92239606 | 0.00022068 | 0.0383618 | 42.4715708 | 44.7766209 | 31.6926895 | 40.1822668 | 81.9562685 | 99.6974195 | 91.3575777 |
| Tubb4a | ENSMUSG00000062591 | 6047.27183 | 7445.15512 | 4183.42744 | 0.60424929 | -0.7267842 | 0.00022102 | 0.0383618 | 7072.0104 | 7390.32668 | 7565.90932 | 7752.37409 | 4514.42446 | 2629.51944 | 5406.33843 |
| Galc | ENSMUSG00000021003 | 1424.1806 | 1721.63643 | 1027.57281 | 0.62973208 | -0.6671899 | 0.00022644 | 0.03848702 | 1884.55249 | 2000.75048 | 1548.13986 | 1453.10291 | 979.572543 | 811.958791 | 1291.1871 |
| Ddc | ENSMUSG00000020182 | 56.497054 | 22.2353831 | 102.179282 | 2.13534984 | 1.09447245 | 0.00022838 | 0.03848702 | 3.9508438 | 33.8554939 | 24.9699978 | 26.165197 | 44.8808137 | 141.877097 | 119.779935 |
| Spred3 | ENSMUSG00000037239 | 2668.0353 | 3032.54155 | 2182.02698 | 0.73029504 | -0.4534487 | 0.00023113 | 0.03848702 | 2832.755 | 3192.24544 | 3265.3074 | 2839.85835 | 2099.64155 | 2131.03234 | 2315.40705 |
| Mrpl2 | ENSMUSG00000002767 | 984.208049 | 733.625138 | 1318.3186 | 1.66532237 | 0.73580148 | 0.00023248 | 0.03848702 | 721.028993 | 614.859453 | 751.981087 | 846.631018 | 1167.87683 | 1815.64339 | 971.435576 |
| Tfg | ENSMUSG00000022757 | 2116.02545 | 2384.46093 | 1758.11147 | 0.74609256 | -0.4225735 | 0.0002346 | 0.03848702 | 2410.01472 | 2331.66063 | 2372.14979 | 2424.01861 | 1624.49032 | 1816.60202 | 1833.24206 |
| Aldh2 | ENSMUSG00000029455 | 1753.79556 | 1339.01196 | 2306.84037 | 1.61923456 | 0.69531199 | 0.0002359 | 0.03848702 | 1099.32229 | 1473.26004 | 1407.92372 | 1375.54179 | 3125.07057 | 1843.44363 | 1952.00691 |
| Wrb | ENSMUSG00000023147 | 921.194239 | 1091.15434 | 694.580777 | 0.66067464 | -0.5979881 | 0.00024485 | 0.03915854 | 1114.13795 | 1003.65158 | 1225.45066 | 1021.37715 | 844.930102 | 648.033227 | 590.779002 |
| Rab31 | ENSMUSG00000056515 | 13838.3476 | 17103.6103 | 9484.66406 | 0.59975848 | -0.7375465 | 0.0002464 | 0.03915854 | 15044.8132 | 24612.944 | 12579.1166 | 16177.5675 | 9255.20432 | 9764.59534 | 9434.19252 |
| Pcyt1b | ENSMUSG00000035246 | 2349.15771 | 2941.67819 | 1559.13041 | 0.58335645 | -0.7775504 | 0.00024722 | 0.03915854 | 3452.04977 | 3416.12854 | 2598.80054 | 2299.73392 | 1293.73824 | 1148.43758 | 2235.2154 |
| Kcnk10 | ENSMUSG00000033854 | 783.398789 | 985.123501 | 514.432507 | 0.57873921 | -0.7890147 | 0.00025962 | 0.04072749 | 1099.32229 | 1289.78511 | 941.176839 | 610.209773 | 554.180482 | 406.45871 | 582.658329 |
| Acan | ENSMUSG00000030607 | 2332.65673 | 810.309782 | 4362.45266 | 2.10993104 | 1.07719585 | 0.00026285 | 0.04084163 | 577.810905 | 779.768472 | 580.072256 | 1303.58749 | 8261.9724 | 3297.68388 | 1527.70172 |
| Dusp5 | ENSMUSG00000034765 | 212.117425 | 270.624779 | 134.107619 | 0.56308396 | -0.828578 | 0.00028177 | 0.04278257 | 392.121247 | 251.185922 | 207.443058 | 231.748888 | 140.49646 | 139.00121 | 122.825188 |
| Cxadr | ENSMUSG00000022865 | 2099.3043 | 2520.46519 | 1537.75644 | 0.64064304 | -0.6424074 | 0.00028321 | 0.04278257 | 2171.97638 | 2978.19135 | 2927.25205 | 2004.44099 | 1459.60212 | 1347.83242 | 1805.83479 |
| Alcam | ENSMUSG00000022636 | 4143.42746 | 4782.81767 | 3290.90718 | 0.70344402 | -0.5074925 | 0.0002889 | 0.04324181 | 4493.09711 | 5250.87789 | 4799.04149 | 4588.25419 | 3284.10476 | 2831.79017 | 3756.82661 |
| Ube2d2a | ENSMUSG00000091896 | 1055.23717 | 1256.88333 | 786.375625 | 0.65261564 | -0.6156945 | 0.00029336 | 0.04351086 | 1207.97049 | 1181.66595 | 1381.03295 | 1256.86393 | 600.036966 | 793.74484 | 965.345071 |
| Ptpn14 | ENSMUSG00000026604 | 1455.78557 | 1116.43264 | 1908.25614 | 1.60783037 | 0.68511521 | 0.00029667 | 0.04360444 | 1112.16253 | 1273.40341 | 1005.5226 | 1074.64202 | 2236.23533 | 2211.55718 | 1276.97592 |
| Il17rc | ENSMUSG00000030281 | 262.300655 | 149.272336 | 413.005081 | 2.00197396 | 1.00142321 | 0.00030398 | 0.04408007 | 173.837127 | 145.25099 | 86.4346077 | 191.566621 | 332.703423 | 709.385485 | 196.926334 |
| Gap43 | ENSMUSG00000047261 | 5279.36858 | 6511.61536 | 3636.37287 | 0.60370785 | -0.7280775 | 0.00030743 | 0.04408007 | 7984.65531 | 4951.63901 | 8398.56271 | 4711.60441 | 3887.06874 | 3831.64025 | 3190.40963 |
| Trappc12 | ENSMUSG00000020628 | 938.250741 | 1121.69989 | 693.651873 | 0.64734116 | -0.6274018 | 0.00031282 | 0.04408007 | 1210.93362 | 1253.74539 | 1070.82875 | 951.291806 | 720.044359 | 539.708146 | 821.203115 |
| B4galt6 | ENSMUSG00000056124 | 1794.29028 | 2278.99944 | 1148.0114 | 0.56871018 | -0.8142345 | 0.00031407 | 0.04408007 | 3215.98685 | 2302.17358 | 1840.09676 | 1757.74056 | 905.421633 | 880.980082 | 1657.63249 |
| Metap1d | ENSMUSG00000041921 | 588.338986 | 418.673222 | 814.560006 | 1.74071085 | 0.79967658 | 0.00031666 | 0.04408007 | 237.050628 | 467.424238 | 493.637648 | 476.580374 | 691.749933 | 760.192824 | 991.73726 |
| Padi2 | ENSMUSG00000028927 | 1060.39774 | 685.811009 | 1559.84673 | 1.87626839 | 0.90786621 | 0.00031826 | 0.04408007 | 1160.56037 | 417.187053 | 492.677264 | 672.819352 | 2009.87992 | 1051.61605 | 1618.04421 |
| Cbll1 | ENSMUSG00000020659 | 432.606473 | 532.548379 | 299.350599 | 0.60642375 | -0.7216018 | 0.00031881 | 0.04408007 | 562.995241 | 602.846213 | 484.033803 | 480.31826 | 220.501389 | 284.712823 | 392.837584 |
| Rps2 | ENSMUSG00000044533 | 1413.49806 | 1039.51912 | 1912.13664 | 1.68411724 | 0.75199258 | 0.0003268 | 0.04480472 | 795.107314 | 825.637205 | 1498.19987 | 1039.13211 | 2272.33511 | 1954.6446 | 1509.4302 |
| Osbpl1a | ENSMUSG00000044252 | 1087.33307 | 1326.51776 | 768.420159 | 0.61864598 | -0.692814 | 0.00033312 | 0.04529076 | 1234.63869 | 1465.61525 | 1300.36065 | 1305.45644 | 715.16601 | 543.542662 | 1046.55181 |
| Stmn3 | ENSMUSG00000027581 | 2581.28309 | 3272.84342 | 1659.20266 | 0.57127172 | -0.807751 | 0.0003385 | 0.04564207 | 3803.67487 | 3677.14348 | 3601.44199 | 2009.11334 | 1894.75087 | 1033.4021 | 2049.45499 |
| Paip2 | ENSMUSG00000037058 | 2190.6717 | 2535.17963 | 1731.32781 | 0.69946073 | -0.515685 | 0.00034752 | 0.04647388 | 2696.45089 | 2187.50175 | 2784.15475 | 2472.61112 | 1951.33973 | 1543.39274 | 1699.25094 |
| Tmem28 | ENSMUSG00000071719 | 90.1481953 | 49.9684005 | 143.721255 | 2.01044921 | 1.00751789 | 0.00035059 | 0.04650292 | 60.2503679 | 74.263664 | 32.653074 | 32.7064963 | 190.255623 | 80.5248388 | 160.383303 |
| Mmp12 | ENSMUSG00000049723 | 152.507609 | 100.160496 | 222.303761 | 1.85198623 | 0.88907337 | 0.00036337 | 0.04754943 | 128.402423 | 104.84282 | 73.9496088 | 93.4471322 | 185.377274 | 165.842823 | 315.691185 |
| Cldnd1 | ENSMUSG00000022744 | 2299.30655 | 2681.65346 | 1789.51068 | 0.68667939 | -0.5422914 | 0.00036431 | 0.04754943 | 2923.62441 | 2838.40092 | 2206.96365 | 2757.62487 | 1554.24209 | 1782.09137 | 2032.19856 |
| Ust | ENSMUSG00000047712 | 1535.37605 | 1802.49651 | 1179.21544 | 0.67610117 | -0.5646889 | 0.00037045 | 0.04778024 | 2112.71372 | 1962.52653 | 1474.19025 | 1660.55554 | 1120.069 | 1109.13379 | 1308.44353 |
| Hexim1 | ENSMUSG00000048878 | 1890.26268 | 2229.56072 | 1437.8653 | 0.66871473 | -0.5805372 | 0.0003731 | 0.04778024 | 2138.39421 | 2764.13726 | 2192.55788 | 1823.15355 | 1491.79922 | 1260.59718 | 1561.19949 |
| Brinp1 | ENSMUSG00000028351 | 2399.04477 | 3015.91535 | 1576.55066 | 0.58172722 | -0.7815853 | 0.00037486 | 0.04778024 | 2655.95474 | 4157.67307 | 2984.87512 | 2265.15848 | 1496.67757 | 1069.83 | 2163.14442 |
| Zc3h18 | ENSMUSG00000017478 | 2029.94973 | 1656.20351 | 2528.27802 | 1.47765335 | 0.56330786 | 0.00038124 | 0.04821351 | 1861.83514 | 1589.02399 | 1749.82061 | 1424.13429 | 2238.18667 | 3047.4817 | 2299.1657 |
| Adamtsl3 | ENSMUSG00000070469 | 764.83729 | 979.122409 | 479.123798 | 0.56215578 | -0.8309581 | 0.00038417 | 0.04821351 | 1482.55413 | 1051.70454 | 732.773396 | 649.457569 | 359.04651 | 511.907904 | 566.416982 |
