# Supplementary material for: Effects of combination therapy of a CDK4/6 and MEK inhibitor in diffuse midline glioma preclinical models
Source: PLoS One. 2025 Dec 22;20(12):e0323235. doi: 10.1371/journal.pone.0323235 (PMC12721541; doi:10.1371/journal.pone.0323235)
Supplement: S6 Table — (DOCX) [file pone.0323235.s013.docx]

**Supplemental table 6. Differentially expressed genes between tumors treated with combination therapy and those treated with vehicle**

| **Gene name** | **Gene ID** | **Base Mean** | **Vehicle_mean** | **Combination_mean** | **fold change** | **log2 fold change** | **p Value** | **FDR Adj p Value** | **Vehicle_1516F_S50** | **Vehicle_1612M_S52** | **Vehicle_1620M_S51** | **Vehicle_1771M_S53** | **Combi_1621M_S61** | **Combi_1802M_S62** | **Combi_430M_S64** | **Combi_561F_S63** |
| --- | --- | --- | --- | --- | --- | --- | --- | --- | --- | --- | --- | --- | --- | --- | --- | --- |
| Tns2 | ENSMUSG00000037003 | 2650.23752 | 1538.0369 | 3762.43815 | 2.33182795 | 1.22146134 | 1.75E-15 | 1.62E-11 | 1716.05839 | 1416.3422 | 1431.04178 | 1588.70523 | 3324.88187 | 3144.87958 | 4923.47401 | 3656.51713 |
| Ankrd34c | ENSMUSG00000047606 | 83.9464978 | 158.357774 | 9.53522125 | 0.1685216 | -2.5689946 | 1.98E-15 | 1.62E-11 | 247.308745 | 154.944925 | 127.102114 | 104.075315 | 14.4685895 | 12.2567571 | 10.5286801 | 0.88685839 |
| Lect1 | ENSMUSG00000022025 | 113.336363 | 10.7023532 | 215.970373 | 5.810537 | 2.5386715 | 1.96E-14 | 1.07E-10 | 11.3271181 | 12.4787859 | 11.887248 | 7.11626084 | 393.545634 | 114.396399 | 300.067382 | 55.8720784 |
| Eya1 | ENSMUSG00000025932 | 1173.3327 | 603.758058 | 1742.90733 | 2.63970826 | 1.40037849 | 1.54E-13 | 6.27E-10 | 690.954203 | 658.255955 | 496.521206 | 569.300867 | 1677.39181 | 1420.76242 | 2495.29717 | 1378.17793 |
| Adam19 | ENSMUSG00000011256 | 838.282507 | 1235.83158 | 440.73343 | 0.38778827 | -1.3666589 | 2.25E-13 | 7.35E-10 | 1437.60007 | 932.789244 | 1192.38242 | 1380.5546 | 532.444094 | 527.040554 | 354.026867 | 349.422204 |
| Itih3 | ENSMUSG00000006522 | 173.141198 | 59.0811122 | 287.201284 | 3.63974724 | 1.86383827 | 1.29E-12 | 3.01E-09 | 46.2523988 | 59.2742329 | 66.7514697 | 64.0463476 | 358.82102 | 207.343474 | 413.250692 | 169.389952 |
| Pstpip2 | ENSMUSG00000025429 | 379.427874 | 542.953278 | 215.902471 | 0.42653327 | -1.2292698 | 5.08E-12 | 8.31E-09 | 597.505478 | 527.228703 | 559.615061 | 487.463868 | 269.115765 | 236.96397 | 152.665861 | 204.864287 |
| Lhfp | ENSMUSG00000048332 | 617.643306 | 835.834361 | 399.45225 | 0.49671418 | -1.0095122 | 3.84E-11 | 4.68E-08 | 942.98258 | 761.205938 | 906.174061 | 732.974867 | 393.545634 | 485.1633 | 382.980737 | 336.119329 |
| Pde3a | ENSMUSG00000041741 | 655.755384 | 945.094044 | 366.416723 | 0.42026523 | -1.250628 | 4.01E-11 | 4.68E-08 | 979.795713 | 880.794303 | 987.55599 | 932.23017 | 395.47478 | 502.527039 | 239.527471 | 328.137603 |
| Col9a3 | ENSMUSG00000027570 | 6506.48661 | 2759.34895 | 10253.6243 | 3.03699146 | 1.60264285 | 7.73E-11 | 8.41E-08 | 2894.07867 | 3286.08028 | 2336.30144 | 2520.9354 | 9994.90163 | 6827.01368 | 17794.7854 | 6397.7964 |
| Aldh2 | ENSMUSG00000029455 | 2220.9746 | 1275.83038 | 3166.11882 | 2.30440122 | 1.20439192 | 1.20E-10 | 1.22E-07 | 1050.5902 | 1402.82351 | 1340.51582 | 1309.39199 | 4167.91835 | 2154.12505 | 3610.02117 | 2732.41069 |
| Gap43 | ENSMUSG00000047261 | 4111.02111 | 6206.77171 | 2015.2705 | 0.37312528 | -1.422268 | 2.59E-10 | 2.49E-07 | 7630.70188 | 4714.90126 | 7996.46031 | 4485.02339 | 1575.14711 | 2956.94264 | 1996.50096 | 1532.49129 |
| Hcar1 | ENSMUSG00000049241 | 24.9909346 | 1.64866751 | 48.3332018 | 4.42687073 | 2.14628724 | 3.29E-10 | 2.98E-07 | 3.77570602 | 1.03989882 | 0 | 1.77906521 | 46.2994864 | 26.556307 | 94.7581205 | 25.7188932 |
| Fam46a | ENSMUSG00000032265 | 824.065801 | 1184.44224 | 463.689362 | 0.42673443 | -1.2285896 | 4.41E-10 | 3.79E-07 | 1550.87125 | 830.879159 | 935.43498 | 1420.58357 | 490.002898 | 464.735372 | 382.980737 | 517.03844 |
| Adra2c | ENSMUSG00000045318 | 61.6196987 | 14.9463437 | 108.293054 | 3.85496263 | 1.94671688 | 6.60E-10 | 5.39E-07 | 19.8224566 | 14.5585835 | 18.2880739 | 7.11626084 | 129.252733 | 48.0056318 | 159.246286 | 96.6675642 |
| Clic5 | ENSMUSG00000023959 | 1547.05648 | 2467.67507 | 626.437905 | 0.32595706 | -1.6172462 | 8.30E-10 | 6.45E-07 | 3522.73372 | 2094.35623 | 1891.90124 | 2361.70907 | 1153.62887 | 545.425689 | 360.607292 | 446.089769 |
| Ccl11 | ENSMUSG00000020676 | 38.0023059 | 67.7416805 | 8.26293123 | 0.25751828 | -1.9572533 | 1.15E-09 | 8.56E-07 | 50.0281048 | 47.8353458 | 85.0395436 | 88.0637279 | 10.610299 | 12.2567571 | 1.31608501 | 8.86858387 |
| Hey2 | ENSMUSG00000019789 | 818.062549 | 518.971946 | 1117.15315 | 2.04557124 | 1.03250378 | 1.24E-09 | 8.81E-07 | 427.598707 | 533.468096 | 499.264417 | 615.556563 | 1337.86224 | 836.523669 | 1239.75208 | 1054.47462 |
| Klf15 | ENSMUSG00000030087 | 421.000497 | 267.903924 | 574.097069 | 2.0376803 | 1.02692772 | 1.84E-09 | 1.22E-06 | 319.047159 | 308.84995 | 223.114502 | 220.604086 | 488.073753 | 567.89641 | 602.766933 | 637.65118 |
| Col9a1 | ENSMUSG00000026147 | 2607.44161 | 321.367515 | 4893.51571 | 4.12254409 | 2.04353492 | 1.86E-09 | 1.22E-06 | 312.439674 | 263.094402 | 363.018267 | 346.917716 | 5663.00593 | 1983.55185 | 10423.3933 | 1504.11182 |
| Mmrn2 | ENSMUSG00000041445 | 355.742884 | 510.234643 | 201.251125 | 0.43059006 | -1.2156131 | 2.00E-09 | 1.26E-06 | 459.692208 | 463.794875 | 563.272676 | 554.178813 | 192.914527 | 230.835591 | 255.320491 | 125.933891 |
| Rasal2 | ENSMUSG00000070565 | 1317.31855 | 1694.93862 | 939.698481 | 0.56861614 | -0.814473 | 3.02E-09 | 1.77E-06 | 1817.05852 | 1540.09016 | 1427.38417 | 1995.22163 | 968.430924 | 1005.05408 | 815.972704 | 969.336217 |
| Slx4 | ENSMUSG00000039738 | 1007.26599 | 1225.2299 | 789.302082 | 0.6508011 | -0.6197114 | 3.04E-09 | 1.77E-06 | 1348.87098 | 1106.45235 | 1252.73306 | 1192.86322 | 795.772423 | 757.876145 | 810.708364 | 792.851398 |
| Acan | ENSMUSG00000030607 | 4111.57339 | 771.970645 | 7451.17613 | 3.89674113 | 1.96226809 | 3.67E-09 | 2.02E-06 | 552.197006 | 742.487759 | 552.299832 | 1240.89798 | 11006.7383 | 3161.22193 | 11865.8224 | 3770.92186 |
| Pld2 | ENSMUSG00000020828 | 1194.21987 | 689.332594 | 1699.10714 | 2.26351755 | 1.1785665 | 3.77E-09 | 2.02E-06 | 521.991358 | 837.118552 | 551.385428 | 846.83504 | 1180.6369 | 1659.76919 | 1913.5876 | 2042.43486 |
| Il17d | ENSMUSG00000050222 | 786.853835 | 571.203173 | 1002.5045 | 1.71585934 | 0.77893129 | 3.92E-09 | 2.02E-06 | 519.159578 | 542.827185 | 643.740201 | 579.085726 | 1013.76584 | 843.673444 | 1134.46528 | 1018.11343 |
| Akap12 | ENSMUSG00000038587 | 4942.32718 | 7156.42983 | 2728.22452 | 0.42261359 | -1.2425889 | 3.95E-09 | 2.02E-06 | 7895.0013 | 6996.43928 | 6667.83174 | 7066.44701 | 3242.89319 | 3909.9055 | 1742.49655 | 2017.60283 |
| B3gnt5 | ENSMUSG00000022686 | 715.065233 | 990.256168 | 439.874297 | 0.4739036 | -1.0773345 | 4.10E-09 | 2.02E-06 | 945.814359 | 915.110964 | 1043.33462 | 1056.76473 | 476.498881 | 545.425689 | 273.745681 | 463.826936 |
| Hs3st1 | ENSMUSG00000051022 | 291.899177 | 105.198616 | 478.599738 | 3.21315424 | 1.68399023 | 4.20E-09 | 2.02E-06 | 43.4206193 | 158.064621 | 83.2107362 | 136.098489 | 589.353879 | 302.333341 | 642.249483 | 380.462248 |
| Kif7 | ENSMUSG00000050382 | 808.04396 | 552.67872 | 1063.4092 | 1.85774508 | 0.89355255 | 5.22E-09 | 2.44E-06 | 614.496156 | 558.425668 | 447.143407 | 590.64965 | 1115.04596 | 874.315337 | 1262.12552 | 1002.14998 |
| Reck | ENSMUSG00000028476 | 395.152804 | 273.581784 | 516.723823 | 1.82645262 | 0.86904433 | 6.41E-09 | 2.91E-06 | 251.084451 | 289.091873 | 260.605053 | 293.54576 | 451.419992 | 529.083346 | 617.243868 | 469.148087 |
| Chst7 | ENSMUSG00000037347 | 170.369468 | 269.73973 | 70.9992052 | 0.34073682 | -1.5532702 | 7.58E-09 | 3.35E-06 | 465.355768 | 237.096932 | 204.826428 | 171.679793 | 68.484657 | 85.7972994 | 60.5399103 | 69.1749542 |
| Wbscr17 | ENSMUSG00000034040 | 658.977225 | 851.917945 | 466.036506 | 0.56305446 | -0.8286536 | 1.23E-08 | 5.29E-06 | 852.365635 | 789.283206 | 861.36828 | 904.654659 | 415.730805 | 594.452717 | 418.515032 | 435.447468 |
| Ednra | ENSMUSG00000031616 | 372.208636 | 523.148438 | 221.268833 | 0.45778256 | -1.1272656 | 1.50E-08 | 6.27E-06 | 709.832733 | 444.036797 | 533.097354 | 405.626868 | 225.709996 | 235.942573 | 251.372236 | 172.050527 |
| Ifngr2 | ENSMUSG00000022965 | 765.640295 | 1004.29719 | 526.983399 | 0.54395719 | -0.878435 | 1.63E-08 | 6.64E-06 | 1081.73978 | 804.881689 | 1051.56425 | 1079.00305 | 649.157382 | 473.92794 | 452.733242 | 532.115032 |
| Glul | ENSMUSG00000026473 | 4251.46093 | 3062.31335 | 5440.60851 | 1.73199459 | 0.79243442 | 1.74E-08 | 6.95E-06 | 3339.61198 | 3347.43431 | 2843.79549 | 2718.41164 | 6069.09101 | 4436.94606 | 6168.49043 | 5087.90657 |
| Als2cl | ENSMUSG00000044037 | 556.455177 | 257.272057 | 855.638298 | 2.7097433 | 1.43815619 | 2.08E-08 | 8.07E-06 | 170.850698 | 340.046915 | 213.970465 | 304.220151 | 865.221652 | 740.512406 | 1347.67105 | 469.148087 |
| Gfpt2 | ENSMUSG00000020363 | 774.163284 | 406.15564 | 1142.17093 | 2.45215598 | 1.29405075 | 2.27E-08 | 8.42E-06 | 462.523988 | 462.754976 | 267.920283 | 431.423313 | 1431.42579 | 971.347997 | 1501.65299 | 664.256932 |
| Fa2h | ENSMUSG00000033579 | 852.14426 | 491.427631 | 1212.86089 | 2.24632425 | 1.16756619 | 2.41E-08 | 8.74E-06 | 458.748282 | 638.497877 | 468.174692 | 400.289672 | 743.6855 | 1296.15206 | 1208.16604 | 1603.43996 |
| Adam11 | ENSMUSG00000020926 | 1691.9462 | 946.606884 | 2437.28552 | 2.31093758 | 1.20847829 | 2.74E-08 | 9.73E-06 | 637.150392 | 1174.04577 | 834.850573 | 1140.3808 | 1473.86698 | 2895.65886 | 2841.42753 | 2538.1887 |
| Matn1 | ENSMUSG00000040533 | 132.87987 | 27.3005646 | 238.459174 | 3.6061233 | 1.85044873 | 2.94E-08 | 1.02E-05 | 25.4860157 | 48.8752447 | 5.48642217 | 29.354576 | 73.3075201 | 275.777034 | 377.716397 | 227.035747 |
| P4ha3 | ENSMUSG00000051048 | 202.728822 | 45.871123 | 359.586521 | 3.54204449 | 1.82458233 | 3.42E-08 | 1.16E-05 | 79.2898265 | 43.6757505 | 33.8329367 | 26.6859781 | 722.464903 | 112.353606 | 450.101072 | 153.426501 |
| Kif3c | ENSMUSG00000020668 | 1952.15825 | 2608.83014 | 1295.48635 | 0.52065579 | -0.9415982 | 3.67E-08 | 1.22E-05 | 3246.16325 | 2672.53997 | 2600.56411 | 1916.05323 | 1215.36152 | 1496.34576 | 1037.07499 | 1433.16315 |
| Serpinh1 | ENSMUSG00000070436 | 2010.29894 | 2964.40991 | 1056.18798 | 0.41011931 | -1.2858844 | 4.25E-08 | 1.39E-05 | 4117.40742 | 2338.73245 | 3410.72578 | 1990.77397 | 1331.11023 | 1162.34913 | 1137.09745 | 594.195119 |
| Rxrg | ENSMUSG00000015843 | 421.794075 | 188.516863 | 655.071287 | 2.74373965 | 1.45614359 | 5.23E-08 | 1.67E-05 | 129.317931 | 290.131771 | 169.164684 | 165.453065 | 496.754906 | 406.515776 | 1104.19532 | 612.819145 |
| Pros1 | ENSMUSG00000022912 | 1409.68635 | 2016.21789 | 803.154805 | 0.44207779 | -1.1776278 | 5.33E-08 | 1.67E-05 | 3208.40619 | 1372.66645 | 1780.34399 | 1703.45494 | 865.221652 | 944.79169 | 694.892884 | 707.712993 |
| Nek6 | ENSMUSG00000026749 | 1419.51792 | 1874.77196 | 964.263882 | 0.53645082 | -0.8984822 | 5.52E-08 | 1.68E-05 | 2180.47023 | 1547.36945 | 1817.83455 | 1953.4136 | 1143.01857 | 1062.25228 | 697.525054 | 954.259624 |
| Unc5b | ENSMUSG00000020099 | 620.756321 | 900.44282 | 341.069823 | 0.42714249 | -1.2272107 | 5.57E-08 | 1.68E-05 | 1089.29119 | 784.083712 | 753.468644 | 974.927735 | 407.049651 | 378.938072 | 405.354182 | 172.937385 |
| Atp8b1 | ENSMUSG00000039529 | 95.7663519 | 142.507253 | 49.0254505 | 0.40292759 | -1.3114075 | 6.30E-08 | 1.87E-05 | 195.392787 | 121.668162 | 110.642847 | 142.325217 | 48.2286317 | 59.2409925 | 46.0629752 | 42.5692026 |
| Slc16a8 | ENSMUSG00000032988 | 92.501664 | 39.4116009 | 145.591727 | 2.80836663 | 1.48973129 | 7.98E-08 | 2.29E-05 | 53.8038109 | 28.0772682 | 38.4049552 | 37.3603694 | 186.162518 | 82.7331102 | 126.344161 | 187.12712 |
| Abcc10 | ENSMUSG00000032842 | 595.693954 | 430.885061 | 760.502847 | 1.71781674 | 0.78057613 | 7.99E-08 | 2.29E-05 | 468.187547 | 412.839833 | 429.769737 | 412.743129 | 658.803109 | 654.715106 | 821.237044 | 907.25613 |
| 2610035D17Rik | ENSMUSG00000087259 | 686.22878 | 477.767987 | 894.689573 | 1.80573997 | 0.85259016 | 8.59E-08 | 2.38E-05 | 551.25308 | 477.31356 | 363.018267 | 519.487041 | 741.756355 | 885.550698 | 993.64418 | 957.807058 |
| Il17rb | ENSMUSG00000015966 | 83.3876225 | 44.2797341 | 122.495511 | 2.405176 | 1.26614247 | 9.12E-08 | 2.48E-05 | 40.5888398 | 50.9550423 | 39.3193589 | 46.2556955 | 152.402476 | 97.0326601 | 103.970716 | 136.576192 |
| Camk2b | ENSMUSG00000057897 | 6647.16821 | 8559.74112 | 4734.5953 | 0.57043626 | -0.8098624 | 9.63E-08 | 2.58E-05 | 8552.91807 | 6974.6014 | 10208.4028 | 8503.04217 | 4690.71672 | 5718.79857 | 4154.88037 | 4373.98556 |
| Pick1 | ENSMUSG00000068206 | 802.97578 | 595.298387 | 1010.65317 | 1.6603421 | 0.73148053 | 1.02E-07 | 2.68E-05 | 617.327935 | 523.069108 | 683.05956 | 557.736943 | 938.529172 | 917.213987 | 997.592435 | 1189.2771 |
| Kcnk2 | ENSMUSG00000037624 | 2584.82799 | 3374.45939 | 1795.1966 | 0.55243076 | -0.8561344 | 1.09E-07 | 2.80E-05 | 4073.9868 | 2981.38992 | 3189.44009 | 3253.02074 | 2079.6186 | 1906.94712 | 1296.34373 | 1897.87695 |
| Fbxo32 | ENSMUSG00000022358 | 513.009889 | 321.181607 | 704.838172 | 2.04583437 | 1.03268935 | 1.10E-07 | 2.80E-05 | 371.907043 | 349.406004 | 241.402575 | 322.010803 | 862.327934 | 621.009024 | 825.185299 | 510.830431 |
| Cdh8 | ENSMUSG00000036510 | 289.94798 | 116.423177 | 463.472783 | 2.88242369 | 1.52728242 | 1.21E-07 | 3.04E-05 | 147.252535 | 169.503508 | 79.5531214 | 69.3835432 | 560.4167 | 225.728609 | 701.473309 | 366.272514 |
| Cdk17 | ENSMUSG00000020015 | 1876.28313 | 2262.62849 | 1489.93777 | 0.66608563 | -0.5862204 | 1.32E-07 | 3.26E-05 | 2352.26485 | 2205.6254 | 2176.28079 | 2316.3429 | 1313.74793 | 1712.8818 | 1412.15921 | 1520.96213 |
| Sema3c | ENSMUSG00000028780 | 71.5215787 | 28.0882334 | 114.954924 | 2.88298498 | 1.52756332 | 1.86E-07 | 4.54E-05 | 27.3738687 | 24.9575717 | 15.5448628 | 44.4766302 | 98.3864086 | 105.203831 | 171.091051 | 85.1384051 |
| Id3 | ENSMUSG00000007872 | 1547.38597 | 908.064742 | 2186.70719 | 2.17965925 | 1.12410261 | 2.09E-07 | 5.02E-05 | 1065.69303 | 904.711976 | 890.629199 | 771.224769 | 2697.90966 | 1492.26017 | 3083.58717 | 1473.07178 |
| Epha2 | ENSMUSG00000006445 | 149.89416 | 206.243865 | 93.5444542 | 0.48893563 | -1.0322836 | 2.12E-07 | 5.03E-05 | 237.86948 | 209.019663 | 197.511198 | 180.575119 | 112.854998 | 102.139642 | 82.9133554 | 76.2698213 |
| Pi4ka | ENSMUSG00000041720 | 2467.0904 | 2981.48685 | 1952.69394 | 0.66309225 | -0.5927185 | 3.00E-07 | 6.82E-05 | 3151.7706 | 3011.54699 | 3017.53219 | 2745.09762 | 1897.31437 | 2271.58564 | 1932.01279 | 1709.86297 |
| Plcd4 | ENSMUSG00000026173 | 217.955692 | 70.9568296 | 364.954555 | 3.07118803 | 1.61879684 | 3.01E-07 | 6.82E-05 | 64.1870024 | 91.5110964 | 66.7514697 | 61.3777497 | 453.349138 | 171.594599 | 708.053734 | 126.820749 |
| Pcyt1a | ENSMUSG00000005615 | 1751.65568 | 2261.96933 | 1241.34204 | 0.56855011 | -0.8146406 | 3.69E-07 | 8.26E-05 | 2562.76046 | 2373.04911 | 1986.08482 | 2125.98293 | 1355.22455 | 1558.65094 | 968.638565 | 1082.85409 |
| Creb5 | ENSMUSG00000053007 | 1597.69595 | 2212.78735 | 982.604562 | 0.48254154 | -1.0512749 | 3.74E-07 | 8.26E-05 | 2274.86288 | 2656.94149 | 1899.21647 | 2020.12855 | 1036.91558 | 1456.5113 | 672.519438 | 764.471929 |
| Khk | ENSMUSG00000029162 | 282.163553 | 150.24926 | 414.077847 | 2.36548521 | 1.24213614 | 3.79E-07 | 8.26E-05 | 105.719769 | 183.022193 | 164.592665 | 147.662412 | 635.653365 | 364.638523 | 393.509417 | 262.510082 |
| Myrip | ENSMUSG00000041794 | 470.390591 | 314.24098 | 626.540202 | 1.89021396 | 0.91854955 | 4.31E-07 | 9.27E-05 | 330.374277 | 372.283778 | 299.010008 | 255.295858 | 645.299092 | 536.233121 | 812.024449 | 512.604148 |
| Galnt12 | ENSMUSG00000039774 | 86.5502054 | 40.77255 | 132.327861 | 2.56978342 | 1.36164677 | 5.09E-07 | 0.00010795 | 22.6542361 | 38.4762564 | 53.0354143 | 48.9242933 | 165.906493 | 94.9898672 | 151.349776 | 117.065307 |
| Prox1 | ENSMUSG00000010175 | 734.098473 | 886.420145 | 581.7768 | 0.66511007 | -0.588335 | 5.24E-07 | 0.00010977 | 952.421845 | 790.323105 | 844.909014 | 958.026616 | 617.326485 | 585.26015 | 535.646598 | 588.873969 |
| Ier5l | ENSMUSG00000089762 | 862.366373 | 1255.547 | 469.185744 | 0.43186847 | -1.2113361 | 5.38E-07 | 0.00011133 | 2067.19905 | 843.357945 | 1140.26141 | 971.369605 | 640.476229 | 479.034922 | 342.182102 | 415.049725 |
| Myo16 | ENSMUSG00000039057 | 1250.45433 | 1725.55938 | 775.349284 | 0.48800873 | -1.0350211 | 5.84E-07 | 0.00011826 | 1793.46036 | 1658.63862 | 1883.67161 | 1566.46692 | 1010.87212 | 938.663312 | 426.411542 | 725.45016 |
| Fn1 | ENSMUSG00000026193 | 5936.47316 | 8821.82822 | 3051.11811 | 0.41323806 | -1.274955 | 5.86E-07 | 0.00011826 | 12697.6994 | 6680.31004 | 9844.47018 | 6064.8333 | 4628.98407 | 3575.90887 | 2109.68427 | 1889.89522 |
| Scml4 | ENSMUSG00000044770 | 661.288535 | 1125.84544 | 196.731631 | 0.32435107 | -1.6243719 | 6.03E-07 | 0.00012009 | 984.515346 | 1526.57147 | 775.414333 | 1216.8806 | 112.854998 | 316.632891 | 78.9651004 | 278.473533 |
| Hrk | ENSMUSG00000046607 | 123.43088 | 34.89167 | 211.970091 | 3.10971124 | 1.63678062 | 6.21E-07 | 0.00012233 | 44.3645458 | 45.7555482 | 19.2024776 | 30.2441086 | 141.792177 | 65.369371 | 455.365412 | 185.353403 |
| Plxnd1 | ENSMUSG00000030123 | 2412.29884 | 3323.37899 | 1501.2187 | 0.48985955 | -1.0295599 | 6.61E-07 | 0.0001285 | 2895.96652 | 3371.35198 | 3360.43358 | 3665.76387 | 2259.02911 | 1184.81985 | 1581.93418 | 979.091659 |
| Gabrb1 | ENSMUSG00000029212 | 331.117102 | 144.770574 | 517.46363 | 2.67667967 | 1.42044449 | 7.00E-07 | 0.00013461 | 87.7851651 | 273.49339 | 93.2691769 | 124.534565 | 457.207428 | 488.22749 | 339.549932 | 784.869672 |
| Trpv4 | ENSMUSG00000014158 | 512.078885 | 255.968534 | 768.189237 | 2.45602766 | 1.29632681 | 8.76E-07 | 0.00016635 | 449.309017 | 207.979765 | 134.417343 | 232.16801 | 913.450284 | 671.057449 | 935.73644 | 552.512775 |
| Elk3 | ENSMUSG00000008398 | 1107.87564 | 1442.26631 | 773.484974 | 0.55936038 | -0.83815 | 8.94E-07 | 0.00016787 | 1728.32943 | 1579.60631 | 1202.44086 | 1258.68864 | 969.395497 | 823.245516 | 631.720803 | 669.578082 |
| Pde1a | ENSMUSG00000059173 | 523.763372 | 785.591369 | 261.935375 | 0.40789638 | -1.2937254 | 1.02E-06 | 0.00018699 | 1494.23566 | 500.191334 | 539.49818 | 608.440302 | 244.036876 | 269.648655 | 313.228232 | 220.827738 |
| Pdgfrb | ENSMUSG00000024620 | 1962.24259 | 2678.04789 | 1246.4373 | 0.50162446 | -0.9953204 | 1.04E-06 | 0.00018967 | 3121.56496 | 2465.60011 | 3122.68862 | 2002.33789 | 1159.41631 | 1390.12053 | 1642.47409 | 793.738256 |
| Col11a2 | ENSMUSG00000024330 | 15388.5213 | 7177.91761 | 23599.125 | 2.55591909 | 1.35384217 | 1.21E-06 | 0.00021579 | 4472.32379 | 11899.5622 | 4552.816 | 7786.96842 | 18218.8479 | 21850.7336 | 38237.5338 | 16089.3849 |
| Gbx1 | ENSMUSG00000067724 | 32.9111067 | 7.46478953 | 58.3574239 | 3.11494964 | 1.63920884 | 1.22E-06 | 0.00021579 | 9.43926506 | 4.15959529 | 9.14403695 | 7.11626084 | 25.0788885 | 25.5349105 | 111.867226 | 70.9486709 |
| Rbp1 | ENSMUSG00000046402 | 1067.33389 | 1555.81809 | 578.849685 | 0.43440697 | -1.2028808 | 1.25E-06 | 0.00021958 | 1348.87098 | 2301.29609 | 1165.86471 | 1407.24058 | 878.725669 | 678.207224 | 392.193332 | 366.272514 |
| Cfap100 | ENSMUSG00000048794 | 198.590384 | 129.72281 | 267.457957 | 1.93496453 | 0.95230712 | 1.35E-06 | 0.00023443 | 105.719769 | 163.264115 | 128.930921 | 120.976434 | 299.982089 | 266.584466 | 207.941431 | 295.323843 |
| Myo5a | ENSMUSG00000034593 | 9493.54003 | 12155.1792 | 6831.90089 | 0.58175182 | -0.7815243 | 1.39E-06 | 0.00023989 | 14122.0845 | 12663.8879 | 10639.087 | 11195.6574 | 7006.65561 | 8431.62746 | 5332.77645 | 6556.54405 |
| Fem1c | ENSMUSG00000033319 | 989.388327 | 1209.87961 | 768.897049 | 0.64598886 | -0.6304188 | 1.46E-06 | 0.00024859 | 1375.30092 | 1250.99828 | 1048.82104 | 1164.39818 | 739.82721 | 777.282677 | 879.144785 | 679.333524 |
| Cdh18 | ENSMUSG00000040420 | 171.569444 | 78.8929428 | 264.245944 | 2.56374606 | 1.35825337 | 1.61E-06 | 0.00027071 | 89.6730181 | 97.7504893 | 34.7473404 | 93.4009235 | 373.289609 | 176.701581 | 314.544317 | 192.44827 |
| Guf1 | ENSMUSG00000029208 | 1744.09239 | 1333.68118 | 2154.5036 | 1.58278284 | 0.66246333 | 1.72E-06 | 0.00028607 | 1159.14175 | 1320.6715 | 1290.22361 | 1564.68785 | 2588.91295 | 1924.31086 | 1975.4436 | 2129.34699 |
| Etnppl | ENSMUSG00000019232 | 31.8532795 | 6.46690632 | 57.2396527 | 3.08788417 | 1.62661864 | 1.78E-06 | 0.000294 | 4.71963253 | 10.3989882 | 2.74321108 | 8.00579344 | 113.819571 | 53.1126139 | 46.0629752 | 15.963451 |
| Mex3c | ENSMUSG00000037253 | 1446.76323 | 1725.94592 | 1167.58055 | 0.68473757 | -0.5463769 | 1.86E-06 | 0.00030343 | 1823.66601 | 1816.70324 | 1624.89537 | 1638.51906 | 1259.73186 | 1254.27481 | 993.64418 | 1162.67135 |
| Cd276 | ENSMUSG00000035914 | 3359.6604 | 4470.16219 | 2249.15862 | 0.53336824 | -0.9067962 | 1.88E-06 | 0.00030343 | 5598.42811 | 4008.80996 | 4279.40929 | 3994.0014 | 3107.85303 | 2205.19487 | 1559.56073 | 2124.02584 |
| Lmo4 | ENSMUSG00000028266 | 3812.88161 | 5025.07406 | 2600.68917 | 0.54504577 | -0.8755507 | 1.91E-06 | 0.0003061 | 5871.22287 | 4601.55229 | 3830.43708 | 5797.08399 | 2908.18649 | 3270.51134 | 1976.75968 | 2247.29915 |
| Ets1 | ENSMUSG00000032035 | 1852.7376 | 2431.94529 | 1273.52992 | 0.55004441 | -0.86238 | 2.08E-06 | 0.00032738 | 2750.60184 | 2045.48098 | 2686.51806 | 2245.18029 | 1474.83156 | 1422.80522 | 1380.57317 | 815.909716 |
| March4 | ENSMUSG00000039372 | 423.587824 | 740.032928 | 107.14272 | 0.33047545 | -1.597385 | 2.10E-06 | 0.00032738 | 1576.35727 | 264.134301 | 643.740201 | 475.899944 | 65.5909391 | 121.546174 | 103.970716 | 137.46305 |
| P2rx4 | ENSMUSG00000029470 | 805.035163 | 534.42822 | 1075.64211 | 1.89273998 | 0.92047623 | 2.12E-06 | 0.00032738 | 453.084723 | 640.577675 | 480.976343 | 563.074139 | 1525.95391 | 788.518038 | 967.32248 | 1020.774 |
| Fam185a | ENSMUSG00000047221 | 245.054444 | 168.923048 | 321.185841 | 1.80978262 | 0.85581642 | 2.12E-06 | 0.00032738 | 191.617081 | 153.905026 | 169.164684 | 161.005402 | 378.112472 | 250.242123 | 359.291207 | 297.09756 |
| Lmx1b | ENSMUSG00000038765 | 425.664928 | 311.349544 | 539.980311 | 1.68022053 | 0.7486506 | 2.36E-06 | 0.00035941 | 274.682613 | 333.807522 | 294.43799 | 342.470053 | 463.959437 | 465.756768 | 604.083018 | 626.122021 |
| 2900005J15Rik | ENSMUSG00000043833 | 170.93819 | 99.9241994 | 241.952181 | 2.14402921 | 1.10032456 | 2.38E-06 | 0.00035941 | 101.944063 | 81.1121082 | 83.2107362 | 133.429891 | 211.241407 | 182.829959 | 334.285592 | 239.451764 |
| Il1rap | ENSMUSG00000022514 | 5105.94975 | 7390.99933 | 2820.90017 | 0.44387655 | -1.1717696 | 2.40E-06 | 0.00035941 | 10748.4911 | 5902.46572 | 5679.36135 | 7233.67914 | 4526.73937 | 2982.47755 | 1856.99594 | 1917.38783 |
| Col9a2 | ENSMUSG00000028626 | 2764.01306 | 1008.91236 | 4519.11376 | 2.80281842 | 1.48687828 | 2.53E-06 | 0.00037174 | 769.300103 | 1548.40935 | 728.779745 | 989.160257 | 8231.66285 | 3773.03838 | 3791.6409 | 2280.11291 |
| Hmgcr | ENSMUSG00000021670 | 1900.75136 | 2279.45821 | 1522.04452 | 0.67618789 | -0.5645039 | 2.53E-06 | 0.00037174 | 2118.17108 | 2074.59815 | 2475.2908 | 2449.77279 | 1607.94258 | 1666.91896 | 1501.65299 | 1311.66355 |
| Dgki | ENSMUSG00000038665 | 162.011125 | 225.139036 | 98.8832133 | 0.48570783 | -1.0418393 | 2.56E-06 | 0.00037282 | 282.234025 | 235.017134 | 225.857713 | 157.447271 | 124.42987 | 88.8614887 | 82.9133554 | 99.3281393 |
| Ctsw | ENSMUSG00000024910 | 51.5603586 | 86.5212381 | 16.5994791 | 0.34640206 | -1.5294806 | 2.65E-06 | 0.00038323 | 67.0187819 | 87.3515011 | 115.214866 | 76.499804 | 11.5748716 | 36.7702712 | 11.8447651 | 6.20800871 |
| Amotl1 | ENSMUSG00000013076 | 1793.34345 | 2305.88993 | 1280.79697 | 0.57728288 | -0.7926496 | 2.68E-06 | 0.00038371 | 2946.93855 | 1931.09211 | 2033.63382 | 2311.89524 | 1643.63177 | 1208.31197 | 1109.45966 | 1161.78449 |
| Cdh5 | ENSMUSG00000031871 | 800.69158 | 1108.4941 | 492.889056 | 0.48901706 | -1.0320433 | 2.76E-06 | 0.00038912 | 1262.02974 | 965.026107 | 1340.51582 | 866.404757 | 666.51969 | 559.725239 | 460.629752 | 284.681542 |
| D930016D06Rik | ENSMUSG00000097392 | 975.674606 | 742.493675 | 1208.85554 | 1.59233443 | 0.67114337 | 2.93E-06 | 0.00040626 | 606.000817 | 773.684724 | 712.320478 | 877.968681 | 1369.69314 | 1095.95836 | 1101.56315 | 1268.20749 |
| Ctif | ENSMUSG00000052928 | 887.666044 | 1056.463 | 718.869086 | 0.68871684 | -0.5380171 | 3.20E-06 | 0.00043984 | 1137.43144 | 958.786714 | 1082.65397 | 1046.97988 | 770.693534 | 764.004523 | 630.404718 | 710.373568 |
| Bcr | ENSMUSG00000009681 | 1520.9466 | 1866.13959 | 1175.75361 | 0.6426275 | -0.6379454 | 3.26E-06 | 0.00044383 | 2070.03083 | 1849.98001 | 1855.3251 | 1689.22242 | 983.864086 | 1372.75679 | 1048.91975 | 1297.47382 |
| Apobec2 | ENSMUSG00000040694 | 25.4064362 | 4.76792679 | 46.0449457 | 2.99471753 | 1.58241993 | 3.63E-06 | 0.00048827 | 0.94392651 | 7.27929176 | 6.40082586 | 4.44766302 | 31.8308969 | 20.4279284 | 102.654631 | 29.2663268 |
| Mink1 | ENSMUSG00000020827 | 4480.40719 | 3066.48599 | 5894.32839 | 1.82240195 | 0.8658412 | 3.65E-06 | 0.00048827 | 2412.67615 | 2672.53997 | 2795.33209 | 4385.39574 | 5349.51983 | 5003.82107 | 7023.94568 | 6200.02698 |
| Glt8d1 | ENSMUSG00000021916 | 1227.44159 | 1052.02437 | 1402.8588 | 1.32675959 | 0.40790697 | 3.90E-06 | 0.00051562 | 1007.16958 | 1022.22054 | 1088.1404 | 1090.56697 | 1410.20519 | 1451.40432 | 1385.83751 | 1363.9882 |
| Magi3 | ENSMUSG00000052539 | 702.029781 | 848.439764 | 555.619798 | 0.66522655 | -0.5880823 | 3.91E-06 | 0.00051562 | 767.412249 | 786.16351 | 906.174061 | 934.009235 | 519.904649 | 618.966232 | 505.376643 | 578.231668 |
| Ccl2 | ENSMUSG00000035385 | 139.174469 | 214.129375 | 64.2195639 | 0.39811132 | -1.3287562 | 4.08E-06 | 0.00053351 | 285.065805 | 157.024722 | 160.020647 | 254.406325 | 108.996708 | 68.4335603 | 34.2182102 | 45.2297777 |
| Rad9b | ENSMUSG00000038569 | 227.725264 | 150.627283 | 304.823244 | 1.89126033 | 0.91934796 | 4.23E-06 | 0.00054794 | 148.196461 | 162.224216 | 142.646976 | 149.441478 | 370.395891 | 247.177934 | 371.135972 | 230.583181 |
| Cyp26b1 | ENSMUSG00000063415 | 452.161837 | 227.642136 | 676.681539 | 2.3887848 | 1.25627689 | 4.27E-06 | 0.00054947 | 385.122015 | 146.625734 | 159.106243 | 219.714553 | 368.466746 | 618.966232 | 975.21899 | 744.074187 |
| Mogat2 | ENSMUSG00000052396 | 26.8739415 | 48.1959475 | 5.55193541 | 0.33745087 | -1.5672506 | 4.32E-06 | 0.00055176 | 50.0281048 | 49.9151435 | 44.805781 | 48.0347607 | 14.4685895 | 5.10698211 | 2.63217001 | 0 |
| Itih5 | ENSMUSG00000025780 | 2563.22153 | 3418.7326 | 1707.71047 | 0.53272288 | -0.9085428 | 4.66E-06 | 0.00058624 | 3255.60252 | 2999.0682 | 2379.27841 | 5040.98127 | 1956.1533 | 1383.99215 | 1764.86999 | 1725.82642 |
| Bcl7a | ENSMUSG00000029438 | 2070.88804 | 1681.59097 | 2460.18512 | 1.44630147 | 0.5323683 | 4.66E-06 | 0.00058624 | 1576.35727 | 1716.87296 | 1716.33574 | 1716.79793 | 2611.09812 | 2031.55748 | 2692.70992 | 2505.37494 |
| Pak2 | ENSMUSG00000022781 | 2563.47574 | 3074.22385 | 2052.72763 | 0.67682602 | -0.5631431 | 4.86E-06 | 0.00060583 | 3354.7148 | 3107.21768 | 2929.74944 | 2905.21349 | 1926.25155 | 2470.75794 | 1975.4436 | 1838.45744 |
| Sertm1 | ENSMUSG00000056306 | 759.777897 | 513.297223 | 1006.25857 | 1.84633911 | 0.88466755 | 5.03E-06 | 0.00062302 | 508.776387 | 557.385769 | 510.237262 | 476.789476 | 662.661399 | 898.828851 | 1410.84313 | 1052.70091 |
| Mmp25 | ENSMUSG00000023903 | 55.2929217 | 79.6283015 | 30.957542 | 0.45317013 | -1.1418753 | 5.20E-06 | 0.00063922 | 96.2805036 | 77.9924117 | 70.4090845 | 73.8312062 | 34.7246148 | 35.7488748 | 27.6377851 | 25.7188932 |
| Msn | ENSMUSG00000031207 | 5411.68776 | 7480.12268 | 3343.25283 | 0.49336678 | -1.0192675 | 5.48E-06 | 0.00066795 | 10359.5934 | 5970.05914 | 7346.31928 | 6244.51889 | 5009.02569 | 3431.89198 | 2440.0216 | 2492.07207 |
| Papss2 | ENSMUSG00000024899 | 1255.61035 | 99.2991751 | 2411.92152 | 2.87283247 | 1.52247386 | 5.57E-06 | 0.00067408 | 127.430078 | 74.8727152 | 68.5802771 | 126.31363 | 2299.54116 | 785.453848 | 6251.40378 | 311.287294 |
| Chrne | ENSMUSG00000014609 | 27.5999575 | 10.6627584 | 44.5371566 | 2.67432607 | 1.41917538 | 5.88E-06 | 0.00070653 | 12.2710446 | 5.19949411 | 10.0584406 | 15.1220543 | 35.6891874 | 32.6846855 | 59.2238253 | 50.550928 |
| Rev3l | ENSMUSG00000019841 | 5472.29545 | 6983.70948 | 3960.88143 | 0.58867649 | -0.7644531 | 6.08E-06 | 0.0007254 | 7354.13141 | 7710.84977 | 6282.86779 | 6586.98894 | 3250.60977 | 4686.16678 | 3044.10462 | 4862.64453 |
| Slpi | ENSMUSG00000017002 | 285.708087 | 190.811947 | 380.604228 | 1.87010009 | 0.90311549 | 6.27E-06 | 0.00073769 | 204.832052 | 231.897437 | 198.425602 | 128.092695 | 460.101146 | 350.338973 | 385.612907 | 326.363886 |
| Abcc9 | ENSMUSG00000030249 | 963.972129 | 1329.92416 | 598.020098 | 0.49549954 | -1.0130444 | 6.28E-06 | 0.00073769 | 1381.90841 | 1199.00334 | 1468.53233 | 1270.25256 | 723.429475 | 494.355868 | 854.139169 | 320.155878 |
| Ablim2 | ENSMUSG00000029095 | 377.915779 | 217.341703 | 538.489855 | 2.15558133 | 1.108077 | 6.36E-06 | 0.00073783 | 286.009731 | 192.381282 | 148.133399 | 242.842401 | 528.585803 | 379.959469 | 838.346149 | 407.068 |
| Clic4 | ENSMUSG00000037242 | 3415.44994 | 4252.01151 | 2578.88837 | 0.62249977 | -0.6838548 | 6.40E-06 | 0.00073783 | 5043.39932 | 3792.51101 | 4186.14011 | 3985.9956 | 3135.82563 | 2785.34804 | 2178.12069 | 2216.25911 |
| 3110079O15Rik | ENSMUSG00000026258 | 24.2538554 | 4.57899606 | 43.9287147 | 2.9131549 | 1.54258241 | 6.41E-06 | 0.00073783 | 5.66355904 | 0 | 7.31522956 | 5.33719563 | 45.3349138 | 33.7060819 | 81.5972704 | 15.0765926 |
| Fam167a | ENSMUSG00000035095 | 50.3694297 | 71.3368828 | 29.4019767 | 0.47029684 | -1.0883565 | 6.85E-06 | 0.00077719 | 67.9627084 | 75.912614 | 66.7514697 | 74.7207388 | 29.9017516 | 28.5990998 | 28.9538701 | 30.1531852 |
| AW146154 | ENSMUSG00000074166 | 100.718437 | 38.8562958 | 162.580577 | 2.67473433 | 1.4193956 | 6.85E-06 | 0.00077719 | 14.1588976 | 59.2742329 | 38.4049552 | 43.5870976 | 125.394442 | 85.7972994 | 275.061766 | 164.068802 |
| Fam167b | ENSMUSG00000050493 | 42.0506254 | 69.8602937 | 14.240957 | 0.36115643 | -1.4693042 | 7.07E-06 | 0.00079695 | 71.7384145 | 37.4363576 | 111.557251 | 58.7091519 | 8.6811537 | 18.3851356 | 23.6895301 | 6.20800871 |
| Lrrc58 | ENSMUSG00000034158 | 2053.33165 | 2470.80854 | 1635.85476 | 0.67183727 | -0.5738163 | 7.45E-06 | 0.00083205 | 2677.9195 | 2596.62736 | 2061.06593 | 2547.62138 | 1587.68655 | 1755.78045 | 1792.50778 | 1407.44426 |
| Trank1 | ENSMUSG00000062296 | 861.318649 | 505.614021 | 1217.02328 | 2.11672944 | 1.08183688 | 7.50E-06 | 0.00083205 | 488.95393 | 652.016562 | 325.527715 | 555.957878 | 1129.51455 | 642.458349 | 1634.57758 | 1461.54262 |
| Mapk1ip1 | ENSMUSG00000041775 | 570.965848 | 398.068528 | 743.863168 | 1.77639545 | 0.82895278 | 7.57E-06 | 0.00083205 | 404.944471 | 399.321148 | 453.544233 | 334.464259 | 528.585803 | 703.742135 | 985.74767 | 757.377062 |
| Xlr3a | ENSMUSG00000057836 | 27.8063797 | 9.07218673 | 46.5405727 | 2.77137872 | 1.47060387 | 7.59E-06 | 0.00083205 | 4.71963253 | 6.23939294 | 15.5448628 | 9.78485865 | 37.6183327 | 71.4977495 | 51.3273153 | 25.7188932 |
| Crkl | ENSMUSG00000006134 | 3085.26235 | 3779.62788 | 2390.89683 | 0.64573148 | -0.6309937 | 7.66E-06 | 0.0008347 | 4118.35135 | 4177.27357 | 3342.1455 | 3480.74108 | 2170.28843 | 2934.47192 | 2082.04648 | 2376.78048 |
| Apc | ENSMUSG00000005871 | 3910.24596 | 4716.40163 | 3104.09028 | 0.66880763 | -0.5803368 | 8.34E-06 | 0.00089633 | 4691.31474 | 5075.74615 | 4528.1271 | 4570.41852 | 2906.25734 | 3596.3368 | 2529.51538 | 3384.2516 |
| Col27a1 | ENSMUSG00000045672 | 2257.4683 | 1482.15034 | 3032.78626 | 1.90040591 | 0.9263076 | 8.65E-06 | 0.00092328 | 1546.15162 | 2061.07947 | 1460.3027 | 861.067562 | 2613.02726 | 3713.79739 | 3015.15075 | 2789.16963 |
| Slc6a19 | ENSMUSG00000021565 | 91.0257861 | 41.7827651 | 140.268807 | 2.47534673 | 1.30763062 | 9.03E-06 | 0.00095765 | 50.9720313 | 21.8378753 | 33.8329367 | 60.4882171 | 136.004741 | 104.182435 | 219.786196 | 101.101856 |
| Akna | ENSMUSG00000039158 | 681.293454 | 850.924899 | 511.662009 | 0.61877761 | -0.6925071 | 9.51E-06 | 0.00100195 | 1061.91732 | 754.966545 | 911.660484 | 675.155247 | 534.373239 | 501.505643 | 475.106687 | 535.662466 |
| Esrrg | ENSMUSG00000026610 | 160.577766 | 226.841672 | 94.3138603 | 0.47449768 | -1.0755271 | 9.67E-06 | 0.0010122 | 230.318068 | 240.216628 | 264.262668 | 172.569325 | 129.252733 | 114.396399 | 69.7525054 | 63.8538038 |
| Fras1 | ENSMUSG00000034687 | 95.8204356 | 32.6523353 | 158.988536 | 2.72580993 | 1.44668497 | 1.02E-05 | 0.00106597 | 16.9906771 | 37.4363576 | 21.031285 | 55.1510215 | 267.186619 | 56.1768032 | 207.941431 | 104.64929 |
| Tesk2 | ENSMUSG00000033985 | 225.382788 | 158.395967 | 292.369609 | 1.76012309 | 0.81567632 | 1.04E-05 | 0.00107068 | 145.364682 | 202.78027 | 127.102114 | 158.336804 | 332.777559 | 292.119377 | 282.958276 | 261.623224 |
| Nid1 | ENSMUSG00000005397 | 2454.58343 | 3378.4576 | 1530.70926 | 0.50038175 | -0.9988989 | 1.04E-05 | 0.00107068 | 3767.21069 | 3346.39441 | 3566.17441 | 2834.05088 | 2178.00501 | 1452.42571 | 1735.91612 | 756.490204 |
| Wnk4 | ENSMUSG00000035112 | 624.298853 | 347.897091 | 900.700615 | 2.19815344 | 1.1362921 | 1.05E-05 | 0.00107531 | 491.78571 | 219.418652 | 224.943309 | 455.440694 | 1357.1537 | 829.373894 | 619.876038 | 796.398831 |
| Jakmip1 | ENSMUSG00000063646 | 408.934091 | 245.008171 | 572.860011 | 2.07076203 | 1.05016177 | 1.09E-05 | 0.00110561 | 219.934876 | 311.969647 | 287.12276 | 161.005402 | 508.329778 | 433.072083 | 881.776955 | 468.261228 |
| Rnd2 | ENSMUSG00000001313 | 3712.76234 | 2661.94107 | 4763.58361 | 1.71533292 | 0.77848861 | 1.14E-05 | 0.00115135 | 2810.06921 | 3111.37728 | 2003.4585 | 2722.8593 | 3813.92019 | 5010.97084 | 6165.85826 | 4063.58513 |
| Neurl1a | ENSMUSG00000006435 | 723.776654 | 1001.99511 | 445.558203 | 0.49478625 | -1.0151227 | 1.16E-05 | 0.00116558 | 844.814223 | 1079.41498 | 1096.37003 | 987.381192 | 232.462005 | 681.271413 | 468.526262 | 399.973132 |
| Hipk2 | ENSMUSG00000061436 | 1267.94431 | 1632.06716 | 903.821446 | 0.57901793 | -0.7883201 | 1.17E-05 | 0.00117008 | 2041.71303 | 1792.78557 | 1219.81453 | 1473.95553 | 1086.10879 | 698.635152 | 856.771339 | 973.770509 |
| Grm5 | ENSMUSG00000049583 | 2577.52908 | 3750.81372 | 1404.24444 | 0.44829578 | -1.1574772 | 1.19E-05 | 0.00117437 | 4378.87506 | 4375.89425 | 3285.45248 | 2963.03311 | 961.678916 | 2121.44037 | 606.715188 | 1927.14327 |
| Sirt2 | ENSMUSG00000015149 | 12099.2937 | 8601.02028 | 15597.5671 | 1.73319552 | 0.79343442 | 1.20E-05 | 0.00117996 | 7780.78619 | 10870.0624 | 6591.93624 | 9161.2963 | 13882.1293 | 14206.6028 | 19634.6722 | 14666.864 |
| Cdr2l | ENSMUSG00000050910 | 1935.77162 | 2330.00017 | 1541.54306 | 0.6722395 | -0.5729528 | 1.21E-05 | 0.00118478 | 2486.30242 | 2157.79006 | 2406.71052 | 2269.19768 | 1741.0536 | 1756.80185 | 1337.14237 | 1331.17444 |
| Trim16 | ENSMUSG00000047821 | 148.686061 | 202.431772 | 94.94035 | 0.51115952 | -0.9681545 | 1.25E-05 | 0.00121356 | 204.832052 | 195.500979 | 203.912024 | 205.482032 | 99.3509813 | 105.203831 | 118.447651 | 56.7589368 |
| Crem | ENSMUSG00000063889 | 345.667587 | 437.290376 | 254.044799 | 0.6022868 | -0.7314774 | 1.34E-05 | 0.00129523 | 534.262402 | 423.238821 | 424.283314 | 367.376966 | 275.867773 | 289.055187 | 219.786196 | 231.470039 |
| Il6ra | ENSMUSG00000027947 | 250.45654 | 160.036653 | 340.876428 | 1.94646058 | 0.96085313 | 1.35E-05 | 0.00129918 | 216.15917 | 173.663103 | 111.557251 | 138.767086 | 339.529567 | 275.777034 | 439.572392 | 308.626719 |
| Trp53i11 | ENSMUSG00000068735 | 1623.02607 | 2190.82625 | 1055.22589 | 0.52245464 | -0.9366223 | 1.42E-05 | 0.00135317 | 2550.48942 | 1709.59366 | 2304.29731 | 2198.9246 | 1105.40024 | 1075.53043 | 1460.85436 | 579.118527 |
| Id4 | ENSMUSG00000021379 | 297.877633 | 181.599267 | 414.156 | 2.03531762 | 1.02525395 | 1.42E-05 | 0.00135317 | 173.682477 | 240.216628 | 140.818169 | 171.679793 | 465.888582 | 227.771402 | 467.210177 | 495.753838 |
| Cd5l | ENSMUSG00000015854 | 32.8366165 | 2.51809895 | 63.1551341 | 2.7238441 | 1.44564413 | 1.46E-05 | 0.00137878 | 2.83177952 | 0 | 4.57201847 | 2.66859781 | 14.4685895 | 128.695949 | 6.58042503 | 102.875573 |
| Plat | ENSMUSG00000031538 | 2521.88827 | 3205.98572 | 1837.79082 | 0.59562269 | -0.7475294 | 1.49E-05 | 0.00140111 | 3245.21933 | 3603.24942 | 2732.23824 | 3243.23588 | 2446.1562 | 1976.40208 | 1460.85436 | 1467.75063 |
| Srebf1 | ENSMUSG00000020538 | 4298.22892 | 2531.65544 | 6064.80241 | 2.09482987 | 1.06683308 | 1.50E-05 | 0.00140111 | 2144.60102 | 3529.4166 | 1391.72242 | 3060.88169 | 9097.84908 | 5261.21297 | 5615.73472 | 4284.41287 |
| Ddx55 | ENSMUSG00000029389 | 833.50673 | 642.455728 | 1024.55773 | 1.5579116 | 0.63961337 | 1.51E-05 | 0.00140203 | 652.253216 | 571.944352 | 612.650475 | 732.974867 | 1202.82207 | 819.15993 | 1098.93098 | 977.317942 |
| Arhgap24 | ENSMUSG00000057315 | 652.648246 | 372.065966 | 933.230527 | 2.1490863 | 1.10372342 | 1.53E-05 | 0.00140655 | 368.131337 | 472.114065 | 245.06019 | 402.95827 | 960.714343 | 591.388528 | 1558.24465 | 622.574588 |
| Akap6 | ENSMUSG00000061603 | 1000.22665 | 1236.78041 | 763.672888 | 0.63354806 | -0.658474 | 1.54E-05 | 0.00140655 | 1441.37577 | 1367.46695 | 1204.26967 | 934.009235 | 711.854604 | 856.951598 | 702.789394 | 783.095956 |
| Tmem28 | ENSMUSG00000071719 | 109.190958 | 47.6290009 | 170.752915 | 2.50677356 | 1.32583168 | 1.54E-05 | 0.00140655 | 57.5795169 | 70.7131199 | 31.0897256 | 31.1336412 | 123.465297 | 108.268021 | 135.556756 | 315.721586 |
| P2rx2 | ENSMUSG00000029503 | 134.519898 | 35.077705 | 233.962092 | 2.76911718 | 1.4694261 | 1.55E-05 | 0.00140655 | 20.7663831 | 59.2742329 | 24.6888998 | 35.5813042 | 619.255631 | 167.509013 | 73.7007604 | 75.3829629 |
| 1500009L16Rik | ENSMUSG00000087651 | 322.546479 | 429.77262 | 215.320338 | 0.53884144 | -0.8920673 | 1.59E-05 | 0.00143666 | 427.598707 | 393.081755 | 457.201847 | 441.208172 | 219.92256 | 280.884016 | 126.344161 | 234.130614 |
| Stc1 | ENSMUSG00000014813 | 382.226085 | 571.128511 | 193.323659 | 0.42926044 | -1.2200749 | 1.60E-05 | 0.00143666 | 707.000953 | 444.036797 | 498.350014 | 635.12628 | 297.088371 | 202.236491 | 211.889686 | 62.0800871 |
| C2cd2l | ENSMUSG00000032120 | 783.570175 | 924.943652 | 642.196699 | 0.70251103 | -0.5094072 | 1.62E-05 | 0.00144548 | 954.309698 | 800.722093 | 989.384798 | 955.358018 | 684.84657 | 658.800692 | 580.393488 | 644.746047 |
| Col8a1 | ENSMUSG00000068196 | 505.395422 | 695.545728 | 315.245115 | 0.50255662 | -0.9926419 | 1.63E-05 | 0.00144548 | 516.327799 | 715.45039 | 1110.08609 | 440.318639 | 338.564994 | 312.547305 | 314.544317 | 295.323843 |
| Lrrc32 | ENSMUSG00000090958 | 199.754887 | 279.822543 | 119.687231 | 0.48434192 | -1.0459022 | 1.64E-05 | 0.00144798 | 387.009868 | 216.298955 | 296.266797 | 219.714553 | 132.146451 | 144.016895 | 128.976331 | 73.6092461 |
| 5031425E22Rik | ENSMUSG00000073147 | 463.377785 | 345.354767 | 581.400803 | 1.63017919 | 0.70503055 | 1.67E-05 | 0.00146615 | 327.542498 | 394.121654 | 382.220744 | 277.534173 | 634.688793 | 543.382896 | 663.306843 | 484.224679 |
| Dscam | ENSMUSG00000050272 | 4798.29567 | 5960.80804 | 3635.78331 | 0.62691777 | -0.6736519 | 1.70E-05 | 0.00148069 | 5858.95182 | 6929.88575 | 5304.45583 | 5749.93876 | 3052.87239 | 4718.85147 | 3552.11343 | 3219.29594 |
| Dcc | ENSMUSG00000060534 | 124.638494 | 182.3739 | 66.9030876 | 0.44743692 | -1.1602438 | 1.70E-05 | 0.00148069 | 201.056346 | 237.096932 | 115.214866 | 176.127456 | 93.5635455 | 49.0270282 | 43.4308052 | 81.5909716 |
| Rad54l2 | ENSMUSG00000040661 | 1324.55388 | 1629.92137 | 1019.18639 | 0.64056246 | -0.6425889 | 1.74E-05 | 0.00150408 | 2029.44199 | 1445.45936 | 1468.53233 | 1576.25178 | 1186.42434 | 1049.99552 | 854.139169 | 986.186526 |
| Osbpl6 | ENSMUSG00000042359 | 1347.18609 | 1627.14184 | 1067.23034 | 0.66750304 | -0.5831537 | 1.75E-05 | 0.00150408 | 1936.93719 | 1365.38715 | 1517.91013 | 1688.33288 | 1022.44699 | 1190.94823 | 964.69031 | 1090.83582 |
| Pamr1 | ENSMUSG00000027188 | 273.404792 | 105.948501 | 440.861082 | 2.59485782 | 1.37565549 | 1.79E-05 | 0.00153362 | 52.8598843 | 174.703002 | 85.0395436 | 111.191576 | 654.944818 | 201.215095 | 737.007604 | 170.27681 |
| Cep170 | ENSMUSG00000057335 | 2766.34341 | 3193.81826 | 2338.86856 | 0.73750118 | -0.4392827 | 1.83E-05 | 0.00155776 | 3425.50929 | 2985.54952 | 3092.5133 | 3271.70092 | 2174.14672 | 2186.80974 | 2449.2342 | 2545.28357 |
| Kif5b | ENSMUSG00000006740 | 3529.88926 | 4150.85488 | 2908.92363 | 0.70825088 | -0.4976676 | 1.84E-05 | 0.00155776 | 4699.81007 | 4050.40591 | 3710.65019 | 4142.55334 | 2841.63098 | 3289.91787 | 2807.20932 | 2696.93635 |
| Zeb1 | ENSMUSG00000024238 | 9399.77227 | 12800.7267 | 5998.8178 | 0.51438901 | -0.9590683 | 1.93E-05 | 0.00162209 | 13553.8407 | 14521.1472 | 11710.7681 | 11417.151 | 4726.4059 | 9605.21195 | 3903.50813 | 5760.14522 |
| 5930430L01Rik | ENSMUSG00000106951 | 49.872126 | 26.2216677 | 73.5225843 | 2.2593313 | 1.17589584 | 1.94E-05 | 0.00162228 | 22.6542361 | 21.8378753 | 29.2609182 | 31.1336412 | 81.0241012 | 50.0484247 | 97.3902905 | 65.6275206 |
| Dlk2 | ENSMUSG00000047428 | 123.865097 | 71.9263746 | 175.80382 | 2.11157744 | 1.07832116 | 1.95E-05 | 0.0016223 | 58.5234434 | 55.1146376 | 87.7827547 | 86.2846627 | 130.217306 | 149.123878 | 168.458881 | 255.415215 |
| Grb14 | ENSMUSG00000026888 | 872.211032 | 460.17155 | 1284.25051 | 2.25936658 | 1.17591837 | 2.04E-05 | 0.00169541 | 422.879075 | 675.934235 | 294.43799 | 447.4349 | 1208.60951 | 800.774795 | 2372.90127 | 754.716487 |
| Wwp2 | ENSMUSG00000031930 | 4749.14394 | 1615.17614 | 7883.11174 | 2.65728327 | 1.40995203 | 2.09E-05 | 0.00171912 | 2037.93733 | 1688.79569 | 1356.97508 | 1376.99647 | 9235.78297 | 3737.28951 | 15236.3161 | 3323.05838 |
| Wif1 | ENSMUSG00000020218 | 76.3981094 | 23.2363754 | 129.559843 | 2.69588945 | 1.43076134 | 2.09E-05 | 0.00171912 | 16.9906771 | 20.7979765 | 32.918533 | 22.2383151 | 179.41051 | 48.0056318 | 252.688321 | 38.1349106 |
| Veph1 | ENSMUSG00000027831 | 26.2789655 | 1.67887837 | 50.8790526 | 2.65077655 | 1.40641506 | 2.13E-05 | 0.00174359 | 2.83177952 | 2.07979765 | 0.91440369 | 0.8895326 | 148.544186 | 22.4707213 | 28.9538701 | 3.54743355 |
| Tmsb10 | ENSMUSG00000079523 | 1410.11379 | 1806.43716 | 1013.79042 | 0.58648231 | -0.7698405 | 2.16E-05 | 0.00175463 | 1821.77816 | 1253.07808 | 2301.5541 | 1849.33829 | 1143.98314 | 1166.43471 | 835.713979 | 909.029846 |
| Peak1 | ENSMUSG00000074305 | 1820.37366 | 2483.04225 | 1157.70506 | 0.5133381 | -0.9620188 | 2.17E-05 | 0.00175463 | 3644.50024 | 2321.05417 | 1903.78849 | 2062.82611 | 1125.65626 | 1461.61828 | 677.783778 | 1365.76192 |
| Fam84b | ENSMUSG00000072568 | 388.435041 | 267.71905 | 509.151033 | 1.79306093 | 0.84242451 | 2.19E-05 | 0.00176433 | 200.112419 | 350.445903 | 226.772116 | 293.54576 | 649.157382 | 464.735372 | 457.997582 | 464.713795 |
| Ccnd2 | ENSMUSG00000000184 | 8184.95068 | 11953.2288 | 4416.6726 | 0.44965386 | -1.1531132 | 2.21E-05 | 0.0017706 | 11369.5948 | 13712.1059 | 11024.9653 | 11706.2491 | 6453.95549 | 5795.4033 | 1483.2278 | 3934.1038 |
| Lpin1 | ENSMUSG00000020593 | 1724.74705 | 2131.76313 | 1317.73097 | 0.6343708 | -0.6566017 | 2.24E-05 | 0.00178774 | 2727.00368 | 1813.58355 | 1816.00574 | 2170.45956 | 1439.14237 | 1389.09913 | 1330.56194 | 1112.12042 |
| Grm6 | ENSMUSG00000000617 | 19.8873708 | 34.4556641 | 5.31907742 | 0.36870346 | -1.4394671 | 2.26E-05 | 0.00179572 | 31.1495747 | 34.3166611 | 43.8913773 | 28.4650434 | 10.610299 | 4.08558569 | 6.58042503 | 0 |
| D16Ertd472e | ENSMUSG00000022864 | 356.638989 | 455.899393 | 257.378585 | 0.59014077 | -0.760869 | 2.28E-05 | 0.00179978 | 526.71099 | 358.765094 | 478.233132 | 459.888357 | 288.407217 | 285.990998 | 190.832326 | 264.283799 |
| Tirap | ENSMUSG00000032041 | 263.597869 | 344.158508 | 183.03723 | 0.56444305 | -0.8251001 | 2.59E-05 | 0.00203059 | 433.262266 | 299.490861 | 321.870101 | 322.010803 | 182.304228 | 160.359238 | 150.033691 | 239.451764 |
| Igfbp3 | ENSMUSG00000020427 | 9079.09554 | 11938.4395 | 6219.75163 | 0.55489373 | -0.8497166 | 2.60E-05 | 0.00203059 | 12408.8578 | 11403.5305 | 9044.36694 | 14897.0025 | 8400.46307 | 6649.29071 | 4460.21209 | 5369.04067 |
| Gng2 | ENSMUSG00000043004 | 1482.06288 | 1794.64469 | 1169.48108 | 0.66410503 | -0.5905167 | 2.65E-05 | 0.00206428 | 1780.24539 | 2011.16432 | 1825.14977 | 1562.01925 | 943.352036 | 1113.3221 | 1260.80944 | 1360.44077 |
| Tspan8 | ENSMUSG00000034127 | 92.8066386 | 18.9156213 | 166.697656 | 2.7000988 | 1.4330122 | 2.76E-05 | 0.0021294 | 10.3831916 | 19.7580776 | 38.4049552 | 7.11626084 | 370.395891 | 62.3051817 | 209.257516 | 24.8320348 |
| Tunar | ENSMUSG00000097929 | 91.8880081 | 50.6535117 | 133.122504 | 2.18681559 | 1.12883157 | 2.76E-05 | 0.0021294 | 46.2523988 | 69.6732211 | 47.5489921 | 39.1394346 | 81.9886738 | 137.888517 | 123.711991 | 188.900836 |
| D430041D05Rik | ENSMUSG00000068373 | 698.06793 | 360.786164 | 1035.3497 | 2.27459885 | 1.18561213 | 2.79E-05 | 0.00213384 | 179.346036 | 404.520642 | 359.360652 | 499.917324 | 935.635455 | 452.478615 | 1722.75527 | 1030.52945 |
| Sc5d | ENSMUSG00000032018 | 986.255098 | 1173.14938 | 799.36082 | 0.69063572 | -0.5340031 | 2.80E-05 | 0.00213384 | 1043.03879 | 1229.16041 | 1143.91902 | 1276.47929 | 734.039774 | 935.599122 | 804.127939 | 723.676444 |
| Plk5 | ENSMUSG00000035486 | 158.926674 | 55.2379946 | 262.615354 | 2.61408016 | 1.38630338 | 2.85E-05 | 0.00216917 | 67.0187819 | 55.1146376 | 35.6617441 | 63.156815 | 95.4926907 | 654.715106 | 118.447651 | 181.805969 |
| Dhh | ENSMUSG00000023000 | 92.8033838 | 138.755623 | 46.8511449 | 0.4336159 | -1.2055104 | 2.87E-05 | 0.00216972 | 141.588976 | 97.7504893 | 127.102114 | 188.580912 | 45.3349138 | 72.5191459 | 47.3790602 | 22.1714597 |
| Kif19a | ENSMUSG00000010021 | 1291.64547 | 249.289678 | 2334.00126 | 2.6906741 | 1.42796766 | 2.88E-05 | 0.00217135 | 135.925417 | 174.703002 | 189.281565 | 497.248726 | 1932.03898 | 887.59349 | 5489.39056 | 1026.98201 |
| Colgalt2 | ENSMUSG00000032649 | 379.102016 | 269.192699 | 489.011332 | 1.72946716 | 0.79032762 | 2.98E-05 | 0.00222637 | 349.252807 | 272.453491 | 247.803401 | 207.261097 | 610.574477 | 420.815326 | 442.204562 | 482.450962 |
| Col3a1 | ENSMUSG00000026043 | 2315.44905 | 3242.03746 | 1388.86064 | 0.48867132 | -1.0330637 | 2.99E-05 | 0.00222637 | 3092.30323 | 3942.25644 | 4397.36737 | 1536.22281 | 1786.38852 | 1666.91896 | 1042.33933 | 1059.79577 |
| Lamb1 | ENSMUSG00000002900 | 1463.08625 | 2005.257 | 920.9155 | 0.50973831 | -0.9721713 | 3.01E-05 | 0.00222637 | 2394.74155 | 1889.49616 | 2022.66097 | 1714.12933 | 1361.97656 | 993.818718 | 881.776955 | 446.089769 |
| Aplp1 | ENSMUSG00000006651 | 6508.28581 | 7626.9889 | 5389.58273 | 0.71396091 | -0.486083 | 3.01E-05 | 0.00222637 | 8083.7866 | 6685.50953 | 8018.406 | 7720.25348 | 5960.0943 | 5226.48549 | 5552.56264 | 4819.18847 |
| Tfg | ENSMUSG00000022757 | 1934.61546 | 2272.34734 | 1596.88359 | 0.71006539 | -0.4939762 | 3.07E-05 | 0.00226228 | 2303.18068 | 2220.18399 | 2258.57713 | 2307.44758 | 1504.73331 | 1797.6577 | 1747.76089 | 1337.38245 |
| Leng8 | ENSMUSG00000035545 | 7792.81068 | 6683.13739 | 8902.48396 | 1.32414769 | 0.40506404 | 3.21E-05 | 0.0023501 | 6167.61579 | 6970.44181 | 6630.34119 | 6964.15076 | 8401.42764 | 9433.61735 | 9119.15301 | 8655.73786 |
| Ttc3 | ENSMUSG00000040785 | 5494.29211 | 6553.09045 | 4435.49377 | 0.68700948 | -0.5415981 | 3.37E-05 | 0.00245101 | 6125.1391 | 6992.27968 | 7036.33643 | 6058.60657 | 4138.0166 | 5368.45959 | 3932.462 | 4303.03689 |
| Kif13a | ENSMUSG00000021375 | 3275.7666 | 4086.14304 | 2465.39016 | 0.62270811 | -0.683372 | 3.38E-05 | 0.00245101 | 3852.16407 | 4264.62507 | 3779.23047 | 4448.55256 | 2122.05979 | 3023.33341 | 1821.46165 | 2894.70577 |
| Col5a2 | ENSMUSG00000026042 | 873.880895 | 1237.99544 | 509.766354 | 0.47867845 | -1.0628712 | 3.54E-05 | 0.00255645 | 1728.32943 | 978.544792 | 1403.60967 | 841.497844 | 630.830502 | 454.521408 | 708.053734 | 245.659773 |
| Pcdh1 | ENSMUSG00000051375 | 2568.73777 | 3216.02026 | 1921.45528 | 0.61783589 | -0.6947044 | 3.59E-05 | 0.00257154 | 3861.60334 | 3225.76615 | 3142.8055 | 2633.90604 | 1964.83445 | 2459.52258 | 1475.33129 | 1786.13279 |
| Lasp1 | ENSMUSG00000038366 | 3658.14574 | 4534.36033 | 2781.93115 | 0.63138321 | -0.6634122 | 3.59E-05 | 0.00257154 | 4838.56727 | 5087.18504 | 4430.2859 | 3781.4031 | 2336.19492 | 3649.44941 | 2458.44679 | 2683.63348 |
| Cav2 | ENSMUSG00000000058 | 3117.78964 | 4208.65338 | 2026.9259 | 0.52669298 | -0.9249659 | 3.63E-05 | 0.00258735 | 5005.64226 | 4593.2331 | 3117.2022 | 4118.53596 | 1488.33557 | 3105.04512 | 1342.40671 | 2171.91619 |
| Npnt | ENSMUSG00000040998 | 209.340754 | 125.246305 | 293.435203 | 2.04632314 | 1.03303398 | 3.65E-05 | 0.00259342 | 101.944063 | 117.508567 | 114.300462 | 167.23213 | 438.880548 | 187.936942 | 230.314876 | 316.608444 |
| Pnpla7 | ENSMUSG00000036833 | 414.271384 | 308.456519 | 520.086249 | 1.62735149 | 0.70252589 | 3.71E-05 | 0.00262366 | 294.50507 | 340.046915 | 284.379549 | 314.894542 | 487.10918 | 393.237622 | 650.145993 | 549.8522 |
| Sult1a1 | ENSMUSG00000030711 | 76.6699026 | 35.5196278 | 117.820177 | 2.38264763 | 1.25256561 | 3.76E-05 | 0.00264995 | 40.5888398 | 38.4762564 | 27.4321108 | 35.5813042 | 54.9806401 | 136.867121 | 198.728836 | 80.7041132 |
| Sptb | ENSMUSG00000021061 | 2976.4836 | 2182.72764 | 3770.23957 | 1.66012141 | 0.73128876 | 3.79E-05 | 0.00265739 | 2005.84383 | 2832.68439 | 2150.67749 | 1741.70484 | 4568.21599 | 3041.71854 | 4261.48325 | 3209.5405 |
| Homer1 | ENSMUSG00000007617 | 861.8179 | 1008.27157 | 715.364233 | 0.71763564 | -0.4786765 | 3.91E-05 | 0.00272792 | 912.776931 | 1032.61953 | 1012.24489 | 1075.44492 | 736.933492 | 698.635152 | 633.036888 | 792.851398 |
| Tmx3 | ENSMUSG00000024614 | 2008.54926 | 2364.62083 | 1652.47769 | 0.70713522 | -0.499942 | 3.94E-05 | 0.00273948 | 2414.564 | 2389.68749 | 2231.14502 | 2423.08682 | 1489.30015 | 2010.10816 | 1534.55512 | 1575.94735 |
| Mical1 | ENSMUSG00000019823 | 1137.62825 | 1330.25988 | 944.996617 | 0.71819519 | -0.4775521 | 4.00E-05 | 0.00276834 | 1153.47819 | 1408.02301 | 1315.82692 | 1443.71142 | 981.934941 | 958.069844 | 844.926574 | 995.05511 |
| Fxyd1 | ENSMUSG00000036570 | 251.025357 | 53.6302444 | 448.42047 | 2.63928352 | 1.40014634 | 4.19E-05 | 0.00287491 | 56.6355904 | 39.5161553 | 67.6658734 | 50.7033585 | 614.432768 | 140.952706 | 923.891675 | 114.404732 |
| Fgf17 | ENSMUSG00000022101 | 14.7429034 | 3.27908483 | 26.206722 | 2.63905501 | 1.40002142 | 4.24E-05 | 0.00289523 | 2.83177952 | 2.07979765 | 7.31522956 | 0.8895326 | 18.32688 | 15.3209463 | 19.7412751 | 51.4377864 |
| Gtf3c2 | ENSMUSG00000106864 | 1547.0802 | 1176.24576 | 1917.91463 | 1.58372719 | 0.66332384 | 4.29E-05 | 0.00291725 | 1141.20715 | 1071.09579 | 1185.06719 | 1307.61293 | 2611.09812 | 1784.37955 | 1631.94541 | 1644.23545 |
| Rbms3 | ENSMUSG00000039607 | 345.919011 | 470.221047 | 221.616974 | 0.52013305 | -0.9430474 | 4.32E-05 | 0.00292516 | 448.36509 | 541.787287 | 632.767357 | 257.964455 | 179.41051 | 249.220727 | 226.366621 | 231.470039 |
| Mmp10 | ENSMUSG00000047562 | 203.392284 | 382.990704 | 23.793864 | 0.38738691 | -1.3681529 | 4.33E-05 | 0.00292516 | 478.570739 | 282.85248 | 596.191209 | 174.348391 | 54.9806401 | 17.3637392 | 21.0573601 | 1.77371677 |
| Arhgef12 | ENSMUSG00000059495 | 3277.75453 | 4113.3292 | 2442.17986 | 0.61505721 | -0.7012075 | 4.39E-05 | 0.00294037 | 5856.12004 | 3510.69842 | 3418.95541 | 3667.54293 | 2486.66825 | 2518.76358 | 2221.55149 | 2541.73614 |
| Cul2 | ENSMUSG00000024231 | 1146.41312 | 1339.10491 | 953.721325 | 0.7201311 | -0.4736685 | 4.44E-05 | 0.00296252 | 1451.75897 | 1379.94574 | 1250.90425 | 1273.81069 | 950.104044 | 1031.61039 | 818.604874 | 1014.56599 |
| En2 | ENSMUSG00000039095 | 302.565075 | 151.689136 | 453.441015 | 2.29048009 | 1.19565002 | 4.49E-05 | 0.00297053 | 166.131065 | 112.309073 | 166.421472 | 161.894934 | 913.450284 | 265.56307 | 227.682706 | 407.068 |
| Ift81 | ENSMUSG00000029469 | 712.648727 | 532.819363 | 892.478091 | 1.61850021 | 0.69465755 | 4.49E-05 | 0.00297053 | 543.701668 | 551.146376 | 560.529465 | 475.899944 | 1183.53062 | 738.469613 | 933.10427 | 714.80786 |
| Pde11a | ENSMUSG00000075270 | 26.3408695 | 44.2965314 | 8.38520757 | 0.38632235 | -1.3721229 | 4.56E-05 | 0.00297551 | 74.570194 | 20.7979765 | 32.0041293 | 49.8138259 | 16.3977348 | 6.12837853 | 6.58042503 | 4.43429193 |
| Pdgfd | ENSMUSG00000032006 | 129.45233 | 42.3930296 | 216.511631 | 2.58448875 | 1.36987892 | 4.56E-05 | 0.00297551 | 32.0935012 | 54.0747388 | 57.6074328 | 25.7964455 | 368.466746 | 100.096849 | 354.026867 | 43.456061 |
| Agt | ENSMUSG00000031980 | 770.020074 | 275.519433 | 1264.52071 | 2.552806 | 1.35208391 | 4.56E-05 | 0.00297551 | 313.3836 | 406.60044 | 83.2107362 | 298.882955 | 2060.32715 | 894.743265 | 1289.76331 | 813.249141 |
| Asap3 | ENSMUSG00000036995 | 908.365801 | 574.116252 | 1242.61535 | 1.9450149 | 0.9597812 | 4.57E-05 | 0.00297551 | 650.365363 | 563.625162 | 487.377169 | 595.097313 | 905.733703 | 917.213987 | 2172.85635 | 974.657367 |
| Tanc1 | ENSMUSG00000035168 | 3002.10926 | 3600.068 | 2404.15052 | 0.67915383 | -0.5581897 | 4.65E-05 | 0.00301548 | 4229.73467 | 3896.50089 | 3014.78898 | 3259.24746 | 2363.20295 | 2643.37394 | 2493.98109 | 2116.04411 |
| Lifr | ENSMUSG00000054263 | 1409.93131 | 1135.74867 | 1684.11395 | 1.46012419 | 0.54609108 | 4.69E-05 | 0.00302581 | 993.954611 | 1317.55181 | 1099.11324 | 1132.37501 | 1640.73805 | 1999.89419 | 1497.70474 | 1598.11881 |
| Rai14 | ENSMUSG00000022246 | 438.46923 | 567.685595 | 309.252864 | 0.57558903 | -0.796889 | 4.70E-05 | 0.00302581 | 672.075672 | 431.558011 | 655.627449 | 511.481248 | 401.262216 | 323.782666 | 264.533086 | 247.43349 |
| Abhd5 | ENSMUSG00000032540 | 513.817048 | 675.915752 | 351.718345 | 0.55688712 | -0.8445432 | 4.75E-05 | 0.00304224 | 776.851515 | 731.048872 | 466.345884 | 729.416736 | 403.191361 | 457.585597 | 265.849171 | 280.24725 |
| Zfp148 | ENSMUSG00000022811 | 1557.89906 | 1811.17524 | 1304.62287 | 0.72637228 | -0.4612189 | 4.78E-05 | 0.00304959 | 1834.99313 | 1790.70577 | 1747.42546 | 1871.5766 | 1190.28263 | 1359.47864 | 1504.28516 | 1164.44506 |
| Ggnbp1 | ENSMUSG00000048731 | 250.441953 | 148.449018 | 352.434887 | 2.05193094 | 1.03698218 | 4.82E-05 | 0.00306688 | 141.588976 | 195.500979 | 149.962206 | 106.743913 | 266.222047 | 367.702712 | 551.439618 | 224.375172 |
| Ncam2 | ENSMUSG00000022762 | 1113.22184 | 1518.09241 | 708.351277 | 0.51766939 | -0.9498971 | 4.87E-05 | 0.00308488 | 2006.78775 | 1526.57147 | 1187.8104 | 1351.20003 | 820.851311 | 949.898672 | 351.394697 | 711.260426 |
| LOC100505025 | ENSMUSG00000108530 | 16.8489211 | 6.66111956 | 27.0367227 | 2.48904134 | 1.31559019 | 4.89E-05 | 0.0030851 | 7.55141205 | 8.31919058 | 3.65761478 | 7.11626084 | 23.1497432 | 37.7916676 | 22.3734451 | 24.8320348 |
| Rnf165 | ENSMUSG00000025427 | 620.863771 | 781.101892 | 460.625649 | 0.61243011 | -0.7073829 | 4.91E-05 | 0.0030851 | 860.860974 | 692.572616 | 754.383048 | 816.590931 | 416.695378 | 611.816457 | 342.182102 | 471.808662 |
| Nos2 | ENSMUSG00000020826 | 122.096074 | 176.110098 | 68.0820497 | 0.46692051 | -1.0987511 | 5.00E-05 | 0.00313097 | 208.607758 | 166.383812 | 142.646976 | 186.801847 | 76.201238 | 108.268021 | 35.5342952 | 52.3246448 |
| Nfxl1 | ENSMUSG00000072889 | 1107.10476 | 764.668838 | 1449.54069 | 1.77840576 | 0.83058452 | 5.10E-05 | 0.00316774 | 847.646003 | 580.263543 | 728.779745 | 901.986061 | 2178.00501 | 1196.05521 | 1162.10306 | 1261.99948 |
| Cd200 | ENSMUSG00000022661 | 2702.6033 | 3540.23074 | 1864.97586 | 0.56187391 | -0.8316817 | 5.16E-05 | 0.00319093 | 4493.09017 | 3533.5762 | 3435.41468 | 2698.84192 | 1529.8122 | 2746.53498 | 1380.57317 | 1802.9831 |
| Adck5 | ENSMUSG00000022550 | 410.749409 | 321.942909 | 499.555909 | 1.51760132 | 0.60179284 | 5.28E-05 | 0.00325761 | 300.168629 | 321.328736 | 360.275056 | 305.999216 | 420.553668 | 464.735372 | 540.910938 | 572.023659 |
| Daam2 | ENSMUSG00000040260 | 773.246571 | 469.161942 | 1077.3312 | 2.01142156 | 1.00821548 | 5.50E-05 | 0.00336819 | 425.710854 | 423.238821 | 370.333496 | 657.364595 | 1720.79758 | 679.22862 | 1201.58561 | 707.712993 |
| Fam107a | ENSMUSG00000021750 | 230.650602 | 49.8761703 | 411.425033 | 2.59891053 | 1.37790697 | 5.50E-05 | 0.00336819 | 128.374005 | 17.67828 | 35.6617441 | 17.7906521 | 474.569736 | 284.969602 | 598.818678 | 287.342117 |
| Sfxn3 | ENSMUSG00000025212 | 1229.11377 | 1479.67074 | 978.556807 | 0.67353839 | -0.5701679 | 5.63E-05 | 0.0034339 | 1823.66601 | 1265.55687 | 1564.54472 | 1264.91536 | 953.962335 | 1034.67458 | 1006.80503 | 918.785289 |
| Hmgcs1 | ENSMUSG00000093930 | 3649.07048 | 4560.33198 | 2737.80897 | 0.62113438 | -0.6870227 | 5.67E-05 | 0.00344161 | 4996.203 | 4271.90436 | 5087.74216 | 3885.47842 | 2019.81509 | 3596.3368 | 2612.42874 | 2722.65525 |
| Rpl9 | ENSMUSG00000047215 | 285.970973 | 206.031954 | 365.909993 | 1.69141006 | 0.75822646 | 5.88E-05 | 0.00355641 | 194.44886 | 172.623205 | 190.195969 | 266.859781 | 346.281575 | 292.119377 | 409.302437 | 415.936583 |
| 0610040J01Rik | ENSMUSG00000060512 | 983.289903 | 601.244622 | 1365.33518 | 1.99691404 | 0.99777223 | 5.91E-05 | 0.00356093 | 500.281048 | 917.190761 | 429.769737 | 557.736943 | 1611.80087 | 952.962861 | 2004.39747 | 892.179537 |
| Atg3 | ENSMUSG00000022663 | 1139.11138 | 1404.53083 | 873.691921 | 0.63959695 | -0.644765 | 6.23E-05 | 0.00374346 | 1489.51603 | 1437.14017 | 1331.37178 | 1360.09535 | 861.363362 | 1151.11377 | 830.449639 | 651.840914 |
| Emilin3 | ENSMUSG00000050700 | 45.120906 | 15.1650481 | 75.0767638 | 2.53683638 | 1.34303047 | 6.35E-05 | 0.00379972 | 11.3271181 | 16.6383812 | 24.6888998 | 8.00579344 | 129.252733 | 34.7274783 | 105.286801 | 31.0400435 |
| Sytl2 | ENSMUSG00000030616 | 543.645408 | 329.777795 | 757.513021 | 2.00676719 | 1.00487325 | 6.57E-05 | 0.00391906 | 245.420892 | 274.533289 | 340.158174 | 458.998824 | 1054.27789 | 379.959469 | 912.04691 | 683.767816 |
| Lrig3 | ENSMUSG00000020105 | 257.954496 | 153.375106 | 362.533885 | 2.03584256 | 1.025626 | 6.94E-05 | 0.00412372 | 123.654372 | 151.825228 | 130.759728 | 207.261097 | 236.320295 | 250.242123 | 575.129148 | 388.443973 |
| Slco1a4 | ENSMUSG00000030237 | 269.200379 | 114.289076 | 424.111681 | 2.41572872 | 1.27245845 | 7.04E-05 | 0.00416924 | 75.5141205 | 225.658044 | 109.728443 | 46.2556955 | 223.780851 | 803.838984 | 275.061766 | 393.765124 |
| Pdxk | ENSMUSG00000032788 | 665.248296 | 865.900153 | 464.596438 | 0.57086705 | -0.8087733 | 7.18E-05 | 0.00422711 | 1242.20728 | 850.637237 | 620.880109 | 749.875986 | 406.085079 | 467.799561 | 402.722012 | 581.779102 |
| Tspan9 | ENSMUSG00000030352 | 1197.25197 | 1560.28545 | 834.218485 | 0.56936747 | -0.812568 | 7.19E-05 | 0.00422711 | 1766.08649 | 1432.98058 | 1238.1026 | 1803.97212 | 1172.92032 | 865.122769 | 531.698343 | 767.132505 |
| Rhoq | ENSMUSG00000024143 | 1967.09521 | 2495.65538 | 1438.53504 | 0.60213483 | -0.7318415 | 7.34E-05 | 0.00428427 | 2403.23688 | 2811.88642 | 1947.67987 | 2819.81836 | 1849.08574 | 1607.67797 | 1276.60246 | 1020.774 |
| Zfp740 | ENSMUSG00000046897 | 4228.0459 | 3648.41181 | 4807.68 | 1.31002459 | 0.38959389 | 7.35E-05 | 0.00428427 | 3581.25716 | 3572.05246 | 3575.31845 | 3865.01917 | 4581.72001 | 5381.73775 | 4722.113 | 4545.14923 |
| Tmem117 | ENSMUSG00000063296 | 1238.90263 | 991.645427 | 1486.15983 | 1.47195313 | 0.55773174 | 7.38E-05 | 0.00428427 | 983.571419 | 1114.77154 | 825.706536 | 1042.53221 | 1715.01014 | 1409.52706 | 1571.4055 | 1248.69661 |
| Rbfox1 | ENSMUSG00000008658 | 261.923564 | 344.348606 | 179.498522 | 0.55958806 | -0.8375629 | 7.40E-05 | 0.00428427 | 366.243484 | 367.084284 | 394.107992 | 249.958662 | 159.154485 | 248.19933 | 142.137181 | 168.503093 |
| Cilp2 | ENSMUSG00000044006 | 247.412499 | 142.434286 | 352.390713 | 2.08512541 | 1.06013416 | 7.48E-05 | 0.00429725 | 137.81327 | 226.697943 | 88.6971584 | 116.528771 | 416.695378 | 198.150906 | 443.520647 | 351.195921 |
| Notch4 | ENSMUSG00000015468 | 574.691539 | 767.578994 | 381.804085 | 0.54123972 | -0.8856604 | 7.49E-05 | 0.00429725 | 1105.33794 | 646.817068 | 682.145156 | 636.015813 | 410.907942 | 469.842354 | 415.882862 | 230.583181 |
| 4933407L21Rik | ENSMUSG00000026224 | 84.322736 | 44.1638933 | 124.481579 | 2.20947722 | 1.14370506 | 7.52E-05 | 0.00429725 | 21.7103096 | 65.5136258 | 50.2922032 | 39.1394346 | 107.067562 | 120.524778 | 178.987561 | 91.3464138 |
| Col4a1 | ENSMUSG00000031502 | 13979.3376 | 19556.446 | 8402.22929 | 0.49541803 | -1.0132817 | 7.52E-05 | 0.00429725 | 26045.7641 | 16251.5388 | 20449.7242 | 15478.7569 | 11767.7861 | 8430.60606 | 9583.73102 | 3826.79394 |
| Chst8 | ENSMUSG00000060402 | 198.76629 | 100.086081 | 297.446499 | 2.25685044 | 1.17431081 | 7.58E-05 | 0.00431416 | 107.607622 | 180.942395 | 54.8642217 | 56.9300867 | 254.647175 | 355.445955 | 398.773757 | 180.919111 |
| Prkd3 | ENSMUSG00000024070 | 2869.24756 | 3271.9368 | 2466.55833 | 0.75857014 | -0.3986455 | 7.71E-05 | 0.00437481 | 3461.3785 | 3413.98783 | 2841.05228 | 3371.32857 | 2366.09667 | 2479.95051 | 2536.09581 | 2484.09034 |
| Tmem176a | ENSMUSG00000023367 | 5338.7125 | 7370.20683 | 3307.21818 | 0.50827392 | -0.9763219 | 8.06E-05 | 0.00455705 | 11046.7719 | 6323.62474 | 5375.77932 | 6734.65135 | 4163.09549 | 4473.71633 | 1775.39867 | 2816.66224 |
| Cald1 | ENSMUSG00000029761 | 3054.06115 | 3716.70423 | 2391.41807 | 0.65851995 | -0.6027009 | 8.13E-05 | 0.00458031 | 4813.08125 | 3108.25758 | 3532.34147 | 3413.13661 | 2017.88595 | 2477.90772 | 2533.46364 | 2536.41499 |
| Shc3 | ENSMUSG00000021448 | 590.184042 | 795.242348 | 385.125736 | 0.53327585 | -0.9070461 | 8.38E-05 | 0.00470346 | 921.27227 | 916.150863 | 732.437359 | 611.1089 | 571.026999 | 328.889648 | 221.102281 | 419.484017 |
| Sdc3 | ENSMUSG00000025743 | 24641.3938 | 31812.7756 | 17470.0121 | 0.58093348 | -0.7835551 | 8.51E-05 | 0.00475973 | 38615.0894 | 36546.2042 | 25072.0349 | 27017.7738 | 18252.6079 | 21089.7933 | 13271.4012 | 17266.2459 |
| Gsk3b | ENSMUSG00000022812 | 3619.46666 | 4252.49963 | 2986.43368 | 0.71084145 | -0.4924003 | 8.54E-05 | 0.00476073 | 4475.15557 | 4020.24885 | 4120.30305 | 4394.29107 | 3204.31029 | 3288.89648 | 3070.42632 | 2382.10163 |
| Srpk3 | ENSMUSG00000002007 | 811.790174 | 582.101938 | 1041.47841 | 1.69840082 | 0.76417697 | 8.66E-05 | 0.00481409 | 506.888534 | 571.944352 | 580.646346 | 668.928519 | 624.078494 | 1260.40318 | 1151.57438 | 1129.85758 |
| Arhgap5 | ENSMUSG00000035133 | 1493.74751 | 1003.54454 | 1983.95048 | 1.82262775 | 0.86601994 | 9.02E-05 | 0.00497812 | 795.730045 | 1082.53467 | 822.963325 | 1312.95012 | 1428.53207 | 1531.07324 | 3037.5242 | 1938.67243 |
| Six1 | ENSMUSG00000051367 | 939.772999 | 163.30643 | 1716.23957 | 2.50500834 | 1.32481541 | 9.02E-05 | 0.00497812 | 212.383464 | 129.987353 | 211.227253 | 99.6276518 | 2994.99803 | 468.820958 | 3078.32283 | 322.816453 |
| Olfml2b | ENSMUSG00000038463 | 249.36317 | 352.426313 | 146.300027 | 0.48785721 | -1.0354692 | 9.28E-05 | 0.00506939 | 453.084723 | 245.416122 | 409.652855 | 301.551553 | 228.603714 | 84.775903 | 164.510626 | 107.309865 |
| Gfod1 | ENSMUSG00000051335 | 891.670738 | 1081.54294 | 701.798538 | 0.6639092 | -0.5909422 | 9.41E-05 | 0.00512051 | 1402.67479 | 982.704387 | 1007.67287 | 933.119703 | 710.890031 | 696.59236 | 634.352973 | 765.358788 |
| Iws1 | ENSMUSG00000024384 | 1525.1858 | 1790.29529 | 1260.0763 | 0.71300033 | -0.4880254 | 9.43E-05 | 0.00512051 | 1928.44185 | 1841.66081 | 1576.43197 | 1814.64651 | 1282.8816 | 1359.47864 | 1038.39107 | 1359.55391 |
| Bcl3 | ENSMUSG00000053175 | 334.35923 | 507.127264 | 161.591196 | 0.43767957 | -1.1920531 | 9.51E-05 | 0.00514521 | 979.795713 | 231.897437 | 433.427351 | 383.388553 | 272.009483 | 167.509013 | 103.970716 | 102.875573 |
| Amy1 | ENSMUSG00000074264 | 342.009119 | 179.545633 | 504.472605 | 2.19600932 | 1.13488418 | 9.58E-05 | 0.00515249 | 137.81327 | 184.062092 | 115.214866 | 281.092303 | 181.339655 | 684.335603 | 558.020043 | 594.195119 |
| Kcnj8 | ENSMUSG00000030247 | 389.816763 | 550.712445 | 228.92108 | 0.48826776 | -1.0342556 | 9.59E-05 | 0.00515249 | 673.019599 | 483.552952 | 497.43561 | 548.841617 | 274.903201 | 272.712845 | 285.590446 | 82.47783 |
| Aak1 | ENSMUSG00000057230 | 3870.50371 | 4773.73196 | 2967.27546 | 0.64030007 | -0.6431799 | 9.64E-05 | 0.00516169 | 5722.08248 | 4016.08925 | 4242.83314 | 5113.92295 | 3693.34861 | 2924.25796 | 2304.46485 | 2947.03042 |
| Snap29 | ENSMUSG00000022765 | 725.161625 | 855.477795 | 594.845456 | 0.70479277 | -0.504729 | 9.72E-05 | 0.00516733 | 864.63668 | 894.312987 | 865.940299 | 797.021214 | 481.321744 | 653.69371 | 606.715188 | 637.65118 |
| Bnip2 | ENSMUSG00000011958 | 1873.38916 | 2098.20869 | 1648.56963 | 0.78894577 | -0.342002 | 9.74E-05 | 0.00516733 | 2141.76924 | 1997.64564 | 2211.94254 | 2041.47733 | 1683.17925 | 1720.03157 | 1588.5146 | 1602.5531 |
| Cd248 | ENSMUSG00000056481 | 759.060428 | 1039.9146 | 478.206258 | 0.51661309 | -0.9528439 | 9.74E-05 | 0.00516733 | 1360.1981 | 780.964016 | 1248.16104 | 770.335236 | 582.601871 | 514.783797 | 577.761318 | 237.678048 |
| Obscn | ENSMUSG00000061462 | 102.655824 | 46.3932886 | 158.91836 | 2.34086918 | 1.22704431 | 9.82E-05 | 0.00518056 | 33.9813542 | 70.7131199 | 30.1753219 | 50.7033585 | 50.1577769 | 178.744374 | 232.947046 | 173.824244 |
| Syt6 | ENSMUSG00000027849 | 874.136857 | 1258.49647 | 489.777247 | 0.47354495 | -1.0784267 | 9.83E-05 | 0.00518056 | 1952.04001 | 1188.60435 | 1094.54122 | 798.800279 | 532.444094 | 689.442585 | 182.935816 | 554.286492 |
| Mapk1 | ENSMUSG00000063358 | 4594.0587 | 5279.22075 | 3908.89664 | 0.74615355 | -0.4224555 | 9.90E-05 | 0.0051987 | 5662.61511 | 5175.57644 | 5270.6229 | 5008.06857 | 3992.36613 | 4345.02038 | 3881.13468 | 3417.06536 |
| Kcnn2 | ENSMUSG00000054477 | 144.679516 | 88.5636792 | 200.795354 | 1.97855061 | 0.98444397 | 9.96E-05 | 0.00520068 | 103.831916 | 100.870186 | 69.4946808 | 80.0579344 | 184.233373 | 142.995499 | 314.544317 | 161.408226 |
| Cbr3 | ENSMUSG00000022947 | 405.604676 | 530.617841 | 280.591511 | 0.56682453 | -0.8190259 | 0.00010055 | 0.00521609 | 496.505342 | 686.333223 | 501.093225 | 438.539574 | 258.505466 | 375.873883 | 185.567986 | 302.41871 |
| Echdc2 | ENSMUSG00000028601 | 399.916891 | 235.149672 | 564.684111 | 2.04313254 | 1.0307828 | 0.00010057 | 0.00521609 | 269.962981 | 348.366106 | 205.740831 | 116.528771 | 404.155933 | 435.114876 | 817.288789 | 602.176845 |
| Tmcc2 | ENSMUSG00000042066 | 1311.33894 | 1032.70677 | 1589.9711 | 1.50465349 | 0.58943128 | 0.00010134 | 0.00523489 | 1101.56223 | 899.512481 | 1054.30746 | 1075.44492 | 1193.17635 | 1631.17009 | 1924.11628 | 1611.42169 |
| Gm3604 | ENSMUSG00000094942 | 28.1899254 | 8.89807466 | 47.4817762 | 2.49261368 | 1.31765931 | 0.00010199 | 0.00523489 | 2.83177952 | 9.3590894 | 10.0584406 | 13.3429891 | 25.0788885 | 27.5777034 | 100.022461 | 37.2480522 |
| Spidr | ENSMUSG00000041974 | 378.926803 | 501.141133 | 256.712472 | 0.5532031 | -0.8541189 | 0.00010222 | 0.00523489 | 723.047704 | 365.004487 | 403.252029 | 513.260313 | 272.974055 | 248.19933 | 313.228232 | 192.44827 |
| Slc27a1 | ENSMUSG00000031808 | 1363.96145 | 875.641721 | 1852.28118 | 1.90101979 | 0.92677355 | 0.0001039 | 0.00528429 | 713.608439 | 782.003915 | 954.637457 | 1052.31707 | 2635.21243 | 1086.76579 | 2462.39505 | 1224.75143 |
| Gramd2 | ENSMUSG00000074259 | 48.3409171 | 22.1510635 | 74.5307707 | 2.32012326 | 1.21420145 | 0.00010428 | 0.00528429 | 17.9346036 | 22.8777741 | 23.7744961 | 24.0173803 | 39.547478 | 78.6475245 | 132.924586 | 47.0034945 |
| Dok7 | ENSMUSG00000044716 | 783.723133 | 537.089598 | 1030.35667 | 1.78532585 | 0.83618742 | 0.00010434 | 0.00528429 | 513.496019 | 752.886748 | 441.656985 | 440.318639 | 1067.78191 | 1044.88854 | 655.410333 | 1353.3459 |
| Dbn1 | ENSMUSG00000034675 | 2707.28788 | 3422.16626 | 1992.4095 | 0.6078397 | -0.7182372 | 0.00010448 | 0.00528429 | 4742.28677 | 2500.95667 | 3723.45184 | 2721.96977 | 1793.14053 | 2201.10929 | 1885.94981 | 2089.43836 |
| Ifitm1 | ENSMUSG00000025491 | 127.022315 | 188.316311 | 65.7283186 | 0.45296194 | -1.1425383 | 0.00010529 | 0.00529263 | 301.112555 | 149.74543 | 227.68652 | 74.7207388 | 71.3783749 | 72.5191459 | 65.8042503 | 53.2115032 |
| Cpeb2 | ENSMUSG00000039782 | 1687.4808 | 1265.23988 | 2109.72172 | 1.60784314 | 0.68512666 | 0.00010617 | 0.00530471 | 1192.17918 | 1647.19973 | 963.781494 | 1257.7991 | 1825.936 | 2155.14645 | 1806.98471 | 2650.81972 |
| AI661453 | ENSMUSG00000034382 | 61.965749 | 89.0421292 | 34.8893688 | 0.47421802 | -1.0763776 | 0.0001065 | 0.00530471 | 112.327254 | 70.7131199 | 85.9539473 | 87.1741953 | 21.2205979 | 46.9842354 | 44.7468902 | 26.6057516 |
| Bach1 | ENSMUSG00000025612 | 1185.09333 | 1573.17197 | 797.014688 | 0.5498434 | -0.8629073 | 0.0001065 | 0.00530471 | 2443.82572 | 1155.32759 | 1145.74783 | 1547.78673 | 865.221652 | 961.134033 | 787.018834 | 574.684235 |
| Hk1 | ENSMUSG00000037012 | 4655.88046 | 5408.92259 | 3902.83833 | 0.72884543 | -0.4563152 | 0.00010714 | 0.00532004 | 5899.54066 | 4787.69418 | 5105.11583 | 5843.33968 | 4326.10826 | 4001.83118 | 3566.59037 | 3716.8235 |
| Nars | ENSMUSG00000024587 | 3637.20343 | 4299.5305 | 2974.87635 | 0.70192745 | -0.5106062 | 0.00010912 | 0.00540194 | 4382.65077 | 3853.86504 | 4104.75819 | 4856.84802 | 3174.40854 | 3441.08454 | 2537.41189 | 2746.60042 |
| Pde8a | ENSMUSG00000025584 | 930.34517 | 1255.70533 | 604.985005 | 0.53235713 | -0.9095337 | 0.00011022 | 0.00543983 | 1480.07676 | 1164.68668 | 928.11975 | 1449.93815 | 784.197551 | 689.442585 | 655.410333 | 290.889551 |
| Kctd11 | ENSMUSG00000046731 | 204.403735 | 273.46254 | 135.34493 | 0.54195033 | -0.8837675 | 0.00011081 | 0.00545264 | 386.065941 | 198.620675 | 307.239641 | 201.923901 | 147.579613 | 148.102481 | 119.763736 | 125.933891 |
| Mmp9 | ENSMUSG00000017737 | 1106.5245 | 2087.47567 | 125.57333 | 0.41232261 | -1.2781545 | 0.0001112 | 0.00545553 | 2182.35808 | 1096.05336 | 4294.03975 | 777.451497 | 298.052944 | 62.3051817 | 119.763736 | 22.1714597 |
| Rbpjl | ENSMUSG00000017007 | 926.924637 | 639.874666 | 1213.97461 | 1.76902405 | 0.82295366 | 0.0001121 | 0.00548326 | 712.664512 | 846.477642 | 575.159924 | 425.196585 | 983.864086 | 1059.18809 | 1709.59442 | 1103.25183 |
| Hdac9 | ENSMUSG00000004698 | 740.575796 | 904.401756 | 576.749837 | 0.65359497 | -0.6135312 | 0.00011293 | 0.00548856 | 1079.85192 | 841.278147 | 920.804521 | 775.672432 | 516.046359 | 496.398661 | 722.530669 | 572.023659 |
| Arhgap29 | ENSMUSG00000039831 | 678.258578 | 845.163535 | 511.353622 | 0.62617619 | -0.6753594 | 0.0001131 | 0.00548856 | 884.459136 | 871.435213 | 998.528835 | 626.230954 | 500.613197 | 537.254518 | 609.347358 | 398.199416 |
| Sema7a | ENSMUSG00000038264 | 1793.93697 | 2404.56818 | 1183.30577 | 0.53995164 | -0.8890979 | 0.00011334 | 0.00548856 | 3695.47227 | 2131.79259 | 2053.7507 | 1737.25718 | 1243.33412 | 1661.81198 | 797.547514 | 1030.52945 |
| Emp2 | ENSMUSG00000022505 | 345.251811 | 461.472178 | 229.031445 | 0.54328144 | -0.8802283 | 0.00011355 | 0.00548856 | 598.449405 | 477.31356 | 352.045422 | 418.080324 | 343.387858 | 221.643024 | 173.723221 | 177.371677 |
| Dok4 | ENSMUSG00000040631 | 344.128626 | 445.863323 | 242.39393 | 0.57797706 | -0.7909159 | 0.00011401 | 0.0054944 | 429.48656 | 462.754976 | 552.299832 | 338.911923 | 210.276834 | 338.082216 | 209.257516 | 211.959154 |
| Tvp23a | ENSMUSG00000050908 | 417.138459 | 586.857632 | 247.419287 | 0.49403923 | -1.0173025 | 0.00011482 | 0.00551709 | 638.094318 | 682.173628 | 416.053681 | 611.1089 | 418.624523 | 265.56307 | 126.344161 | 179.145394 |
| Gna11 | ENSMUSG00000034781 | 3098.71747 | 3494.4228 | 2703.01214 | 0.77752611 | -0.363037 | 0.00012169 | 0.00581281 | 3601.07962 | 3288.16008 | 3425.35624 | 3663.09527 | 2560.94034 | 2734.27822 | 2576.89444 | 2939.93555 |
| Aldh1l2 | ENSMUSG00000020256 | 1185.20696 | 1489.89986 | 880.514057 | 0.61584938 | -0.6993505 | 0.00012254 | 0.00583636 | 1896.34835 | 1462.09774 | 1183.23838 | 1417.91497 | 901.875412 | 837.545066 | 652.778163 | 1129.85758 |
| Nt5e | ENSMUSG00000032420 | 2747.95268 | 3515.22049 | 1980.68486 | 0.59356766 | -0.7525156 | 0.00012425 | 0.00590084 | 4606.36135 | 3080.18031 | 2647.1987 | 3727.14161 | 2610.13355 | 2077.52032 | 1454.27393 | 1780.81164 |
| Wfikkn2 | ENSMUSG00000044177 | 29.9506562 | 12.549468 | 47.3518443 | 2.37101967 | 1.24550763 | 0.00012624 | 0.00596055 | 7.55141205 | 22.8777741 | 7.31522956 | 12.4534565 | 62.6972212 | 27.5777034 | 59.2238253 | 39.9086274 |
| Atraid | ENSMUSG00000013622 | 2220.04782 | 1650.94183 | 2789.15381 | 1.62290432 | 0.69857795 | 0.00012625 | 0.00596055 | 1667.91814 | 1748.06992 | 1623.98096 | 1563.79832 | 4068.56737 | 2688.31538 | 2216.28715 | 2183.44535 |
| Col1a1 | ENSMUSG00000001506 | 2577.8941 | 3570.91271 | 1584.87549 | 0.50824501 | -0.976404 | 0.00012687 | 0.00596055 | 5223.68929 | 3207.04797 | 4112.98782 | 1739.92578 | 2417.21902 | 1435.06197 | 1271.33812 | 1215.88285 |
| Ecscr | ENSMUSG00000073599 | 231.311841 | 335.700998 | 126.922684 | 0.4697573 | -1.0900125 | 0.00012757 | 0.00597157 | 326.598571 | 298.450962 | 421.540103 | 296.214357 | 173.623074 | 178.744374 | 111.867226 | 43.456061 |
| Gm30731 | ENSMUSG00000107859 | 76.0200911 | 45.940002 | 106.10018 | 1.9961231 | 0.99720069 | 0.0001326 | 0.00618887 | 33.0374277 | 66.5535246 | 53.0354143 | 31.1336412 | 104.173844 | 119.503381 | 96.0742055 | 104.64929 |
| Scin | ENSMUSG00000002565 | 114.914968 | 31.0032416 | 198.826694 | 2.47289913 | 1.30620339 | 0.00013334 | 0.00618887 | 67.9627084 | 15.5984823 | 15.5448628 | 24.9069129 | 331.812986 | 67.4121638 | 360.607292 | 35.4743355 |
| Fmod | ENSMUSG00000041559 | 175.379361 | 8.17590059 | 342.582821 | 2.32966439 | 1.22012214 | 0.00013373 | 0.00618887 | 12.2710446 | 10.3989882 | 9.14403695 | 0.8895326 | 654.944818 | 114.396399 | 561.968298 | 39.021769 |
| Gsx1 | ENSMUSG00000053129 | 1228.68114 | 1668.4827 | 788.879568 | 0.52781594 | -0.9218932 | 0.00013445 | 0.00619173 | 1823.66601 | 1595.20479 | 2168.05116 | 1087.00884 | 853.646781 | 977.476376 | 381.664652 | 942.730465 |
| Rgma | ENSMUSG00000070509 | 2728.27096 | 2127.41306 | 3329.12885 | 1.52532298 | 0.60911476 | 0.00013455 | 0.00619173 | 2154.04029 | 2663.18088 | 1569.11674 | 2123.31433 | 3702.02977 | 3287.87508 | 2857.22055 | 3469.39001 |
| Mycl | ENSMUSG00000028654 | 1318.6713 | 775.350813 | 1861.99178 | 2.03274559 | 1.02342967 | 0.00013658 | 0.00626754 | 451.19687 | 1153.24779 | 551.385428 | 945.573159 | 893.194259 | 2349.21177 | 1759.60565 | 2445.95543 |
| Sox7 | ENSMUSG00000063060 | 106.600347 | 161.063137 | 52.1375572 | 0.44488862 | -1.1684839 | 0.00013782 | 0.00630704 | 293.561143 | 95.6706917 | 120.701288 | 134.319423 | 67.5200843 | 73.5405424 | 32.9021252 | 34.5874771 |
| Steap3 | ENSMUSG00000026389 | 331.84117 | 412.647896 | 251.034445 | 0.63072988 | -0.6649058 | 0.00013972 | 0.00637584 | 443.645458 | 348.366106 | 464.517077 | 394.062944 | 298.052944 | 279.86262 | 197.412751 | 228.809464 |
| Prpsap1 | ENSMUSG00000015869 | 1685.66233 | 1450.55911 | 1920.76555 | 1.31474757 | 0.39478583 | 0.00014035 | 0.00638456 | 1447.03933 | 1500.574 | 1475.84756 | 1378.77554 | 1782.53023 | 1943.71739 | 2168.90809 | 1787.90651 |
| Otoa | ENSMUSG00000034990 | 34.3735588 | 6.65719358 | 62.089924 | 2.4487536 | 1.29204762 | 0.00014069 | 0.00638456 | 1.88785301 | 2.07979765 | 15.5448628 | 7.11626084 | 79.0949559 | 26.556307 | 130.292416 | 12.4160174 |
| Armcx6 | ENSMUSG00000050394 | 148.40215 | 94.6411556 | 202.163144 | 1.90287051 | 0.92817739 | 0.00014475 | 0.00653267 | 95.3365771 | 93.590894 | 104.242021 | 85.3951301 | 271.04491 | 150.145274 | 255.320491 | 132.1419 |
| Fry | ENSMUSG00000056602 | 3806.97688 | 2759.78917 | 4854.1646 | 1.67222581 | 0.74176967 | 0.00014569 | 0.00655706 | 3928.62212 | 2627.82432 | 2008.03051 | 2474.67971 | 3872.75912 | 4981.35035 | 4554.97021 | 6007.57871 |
| Rgs17 | ENSMUSG00000019775 | 567.356869 | 747.301013 | 387.412725 | 0.56066721 | -0.8347834 | 0.0001471 | 0.00660221 | 844.814223 | 731.048872 | 682.145156 | 731.195801 | 404.155933 | 539.297311 | 202.677091 | 403.520566 |
| Lpar3 | ENSMUSG00000036832 | 47.6566747 | 76.1974491 | 19.1159004 | 0.42048577 | -1.2498711 | 0.0001489 | 0.00666471 | 132.149711 | 105.029781 | 32.918533 | 34.6917716 | 21.2205979 | 17.3637392 | 23.6895301 | 14.1897342 |
| Mpv17 | ENSMUSG00000107283 | 966.193493 | 743.600365 | 1188.78662 | 1.55247691 | 0.63457182 | 0.00014997 | 0.00669405 | 688.122423 | 958.786714 | 632.767357 | 694.724964 | 1461.32754 | 1215.46174 | 985.74767 | 1092.60953 |
| E330011O21Rik | ENSMUSG00000109841 | 14.2453551 | 22.8582827 | 5.63242746 | 0.42246821 | -1.2430853 | 0.0001529 | 0.00680008 | 22.6542361 | 29.117167 | 19.2024776 | 20.4592499 | 5.7874358 | 6.12837853 | 2.63217001 | 7.98172548 |
| Dbp | ENSMUSG00000059824 | 477.88767 | 322.208522 | 633.566819 | 1.80775684 | 0.85420064 | 0.00015318 | 0.00680008 | 187.841375 | 467.95447 | 348.387808 | 284.650434 | 653.980246 | 539.297311 | 775.174069 | 565.815651 |
| Rinl | ENSMUSG00000051735 | 98.3580768 | 68.5850708 | 128.131083 | 1.74181469 | 0.80059115 | 0.00015371 | 0.00680543 | 74.570194 | 65.5136258 | 63.0938549 | 71.1626084 | 108.996708 | 117.460588 | 159.246286 | 126.820749 |
| B230206H07Rik | ENSMUSG00000086844 | 34.0764412 | 11.6215072 | 56.5313752 | 2.42555825 | 1.27831683 | 0.00015533 | 0.00685834 | 4.71963253 | 2.07979765 | 20.1168813 | 19.5697173 | 48.2286317 | 51.0698211 | 81.5972704 | 45.2297777 |
| Padi2 | ENSMUSG00000028927 | 2530.32286 | 653.976891 | 4406.66884 | 2.44930182 | 1.29237057 | 0.00015669 | 0.0068852 | 1109.11364 | 397.24135 | 469.089095 | 640.463476 | 3589.17477 | 1394.20612 | 10537.8926 | 2105.40181 |
| Ptprn | ENSMUSG00000026204 | 4633.91883 | 7314.49923 | 1953.33843 | 0.42565729 | -1.2322358 | 0.00015678 | 0.0068852 | 10438.8832 | 5306.60369 | 6788.53303 | 6723.97696 | 914.414857 | 3724.01135 | 1522.71035 | 1652.21717 |
| Spag1 | ENSMUSG00000037617 | 308.096601 | 233.434988 | 382.758215 | 1.58426364 | 0.66381244 | 0.00016089 | 0.00704673 | 251.084451 | 271.413593 | 173.736702 | 237.505206 | 339.529567 | 361.574333 | 385.612907 | 444.316052 |
| Tcea2 | ENSMUSG00000059540 | 875.411749 | 683.018176 | 1067.80532 | 1.5229891 | 0.60690561 | 0.0001623 | 0.00708976 | 575.795169 | 743.527658 | 693.118001 | 719.631877 | 786.126696 | 1123.53606 | 1081.82188 | 1279.73665 |
| Lypla2 | ENSMUSG00000028670 | 4825.24158 | 6512.71165 | 3137.77152 | 0.53490581 | -0.9026432 | 0.00016346 | 0.00712129 | 10022.6116 | 3924.57816 | 5355.66244 | 6747.99434 | 4581.72001 | 2725.08565 | 2603.21614 | 2641.06428 |
| Glt1d1 | ENSMUSG00000049971 | 334.446221 | 228.606517 | 440.285925 | 1.78083397 | 0.83255302 | 0.00016417 | 0.00713323 | 134.98149 | 216.298955 | 264.262668 | 298.882955 | 345.317003 | 434.093479 | 494.847963 | 486.885254 |
| Dkk2 | ENSMUSG00000028031 | 110.699484 | 162.673057 | 58.7259104 | 0.46468469 | -1.105676 | 0.00016767 | 0.00724071 | 110.439401 | 215.259056 | 240.488172 | 84.5055975 | 52.0869222 | 81.7117137 | 40.7986352 | 60.3063703 |
| Tfcp2l1 | ENSMUSG00000026380 | 34.9029247 | 7.69087463 | 62.1149748 | 2.43162388 | 1.28192009 | 0.00016774 | 0.00724071 | 1.88785301 | 6.23939294 | 14.6304591 | 8.00579344 | 40.5120506 | 8.17117137 | 60.5399103 | 139.236767 |
| Tmie | ENSMUSG00000049555 | 141.816726 | 88.2123243 | 195.421128 | 1.94056651 | 0.95647788 | 0.00016798 | 0.00724071 | 44.3645458 | 107.109579 | 110.642847 | 90.7323257 | 198.701963 | 221.643024 | 210.573601 | 150.765926 |
| Bmp4 | ENSMUSG00000021835 | 460.465636 | 244.259169 | 676.672104 | 2.15316377 | 1.10645805 | 0.00017149 | 0.00737252 | 111.383328 | 160.144419 | 200.254409 | 505.25452 | 508.329778 | 532.147536 | 798.863599 | 867.347502 |
| Metrn | ENSMUSG00000002274 | 557.268299 | 714.573734 | 399.962864 | 0.59181157 | -0.7567902 | 0.00017294 | 0.00739619 | 606.944743 | 731.048872 | 788.215985 | 732.085334 | 522.798367 | 469.842354 | 355.342952 | 251.867782 |
| Adamts19 | ENSMUSG00000053441 | 77.1582151 | 24.8130208 | 129.503409 | 2.42453503 | 1.2777081 | 0.00017294 | 0.00739619 | 16.9906771 | 13.5186847 | 9.14403695 | 59.5986845 | 182.304228 | 40.8558569 | 207.941431 | 86.9121219 |
| Atp6ap1l | ENSMUSG00000078958 | 32.447549 | 16.4374542 | 48.4576437 | 2.19944556 | 1.13713989 | 0.00017351 | 0.00740102 | 11.3271181 | 17.67828 | 10.0584406 | 26.6859781 | 44.3703411 | 54.1340103 | 42.1147202 | 53.2115032 |
| Emcn | ENSMUSG00000054690 | 362.826628 | 523.884058 | 201.769197 | 0.47660976 | -1.0691196 | 0.00017494 | 0.00744278 | 592.785846 | 428.438315 | 579.731942 | 494.580128 | 258.505466 | 252.284916 | 239.527471 | 56.7589368 |
| Lpp | ENSMUSG00000033306 | 574.730116 | 703.780054 | 445.680177 | 0.65076656 | -0.619788 | 0.00017571 | 0.00745593 | 814.608575 | 612.500406 | 666.600293 | 721.410943 | 515.081786 | 437.157668 | 498.796218 | 331.685037 |
| Setd5 | ENSMUSG00000034269 | 4931.30153 | 5538.35126 | 4324.25179 | 0.78450645 | -0.3501428 | 0.00017622 | 0.00745847 | 5874.05465 | 5737.1218 | 5344.6896 | 5197.53901 | 4039.63019 | 4480.8661 | 4299.64972 | 4476.86114 |
| C1qtnf4 | ENSMUSG00000040794 | 206.15913 | 142.51122 | 269.807041 | 1.76000242 | 0.81557741 | 0.00017702 | 0.00747262 | 132.149711 | 154.944925 | 166.421472 | 116.528771 | 233.426577 | 188.958338 | 317.176487 | 339.666762 |
| 5830444B04Rik | ENSMUSG00000084803 | 36.1537381 | 6.08557562 | 66.2219006 | 2.39600317 | 1.26062981 | 0.00017766 | 0.00748064 | 0 | 13.5186847 | 5.48642217 | 5.33719563 | 50.1577769 | 162.402031 | 40.7986352 | 11.529159 |
| Tmem80 | ENSMUSG00000025505 | 1057.82446 | 902.135323 | 1213.51359 | 1.33396101 | 0.4157165 | 0.00017868 | 0.00750425 | 930.711535 | 921.350357 | 968.353513 | 788.125888 | 1128.54998 | 1223.63291 | 1310.82067 | 1191.05081 |
| Runx3 | ENSMUSG00000070691 | 354.691879 | 183.604239 | 525.779519 | 2.17662967 | 1.12209597 | 0.00018076 | 0.00755853 | 319.047159 | 165.343913 | 100.584406 | 149.441478 | 669.413408 | 196.108113 | 740.955859 | 496.640697 |
| Eml1 | ENSMUSG00000058070 | 901.791605 | 1078.93003 | 724.653183 | 0.68492767 | -0.5459765 | 0.0001809 | 0.00755853 | 1147.81463 | 905.751874 | 1245.41783 | 1016.73577 | 657.838536 | 789.539434 | 626.456463 | 824.7783 |
| 3830403N18Rik | ENSMUSG00000031125 | 42.8121622 | 9.31800757 | 76.3063168 | 2.417785 | 1.27368596 | 0.0001822 | 0.0075874 | 5.66355904 | 2.07979765 | 6.40082586 | 23.1278477 | 182.304228 | 42.8986497 | 63.1720803 | 16.8503093 |
| Ccdc134 | ENSMUSG00000068114 | 949.389634 | 714.429198 | 1184.35007 | 1.59504817 | 0.67359999 | 0.00018252 | 0.0075874 | 533.318476 | 732.088771 | 819.30571 | 773.003834 | 935.635455 | 1196.05521 | 1550.34814 | 1055.36148 |
| Pepd | ENSMUSG00000063931 | 772.573108 | 649.99371 | 895.152505 | 1.36387077 | 0.44770696 | 0.00018594 | 0.00769354 | 724.935557 | 691.532717 | 604.420842 | 579.085726 | 923.09601 | 952.962861 | 862.035679 | 842.515467 |
| Ap3s1 | ENSMUSG00000024480 | 303.597502 | 370.702763 | 236.492241 | 0.65611526 | -0.6079788 | 0.00018624 | 0.00769354 | 332.26213 | 375.403475 | 373.076707 | 402.068737 | 202.560253 | 288.033791 | 207.941431 | 247.43349 |
| Lrrc2 | ENSMUSG00000032495 | 55.2617562 | 31.9046254 | 78.6188871 | 2.04120529 | 1.02942128 | 0.00018649 | 0.00769354 | 32.0935012 | 33.2767623 | 32.0041293 | 30.2441086 | 63.6617938 | 52.0912175 | 117.131566 | 81.5909716 |
| Ildr2 | ENSMUSG00000040612 | 1872.76865 | 2190.71278 | 1554.82452 | 0.71893848 | -0.4760598 | 0.0001886 | 0.0077611 | 2236.16189 | 2531.11373 | 2052.83629 | 1942.73921 | 1757.45134 | 1599.5068 | 1355.56756 | 1506.7724 |
| Slc35f3 | ENSMUSG00000057060 | 41.1593622 | 15.8815939 | 66.4371304 | 2.36934265 | 1.24448685 | 0.00019054 | 0.00782106 | 7.55141205 | 27.0373694 | 17.3736702 | 11.5639239 | 27.9726064 | 43.9200461 | 75.0168454 | 118.839024 |
| Tekt2 | ENSMUSG00000028845 | 86.7506659 | 51.074208 | 122.427124 | 2.013272 | 1.0095421 | 0.00019139 | 0.00783642 | 50.0281048 | 48.8752447 | 48.4633958 | 56.9300867 | 81.9886738 | 91.925678 | 198.728836 | 117.065307 |
| Ret | ENSMUSG00000030110 | 281.327892 | 436.325781 | 126.330004 | 0.43731577 | -1.1932527 | 0.00020742 | 0.00847172 | 594.673699 | 235.017134 | 762.612681 | 152.999608 | 61.7326485 | 120.524778 | 223.734451 | 99.3281393 |
| Rgs9bp | ENSMUSG00000056043 | 27.1974549 | 11.5277853 | 42.8671245 | 2.31416983 | 1.21049475 | 0.00020805 | 0.00847619 | 4.71963253 | 12.4787859 | 16.4592665 | 12.4534565 | 22.1851706 | 37.7916676 | 64.4881653 | 47.0034945 |
| Synpo2l | ENSMUSG00000039376 | 23.5716567 | 10.0165881 | 37.1267253 | 2.30962851 | 1.20766082 | 0.00021333 | 0.00866958 | 14.1588976 | 5.19949411 | 9.14403695 | 11.5639239 | 40.5120506 | 52.0912175 | 38.1664652 | 17.7371677 |
| Pcdhgc3 | ENSMUSG00000102918 | 3508.0085 | 4581.8262 | 2434.1908 | 0.57149093 | -0.8071975 | 0.00021552 | 0.0087369 | 5373.7736 | 5431.39155 | 3377.80725 | 4144.33241 | 3501.39866 | 2459.52258 | 1475.33129 | 2300.51066 |
| March1 | ENSMUSG00000036469 | 696.4818 | 922.918649 | 470.044951 | 0.55606626 | -0.8466713 | 0.0002207 | 0.00892471 | 921.27227 | 1280.11545 | 829.364151 | 660.922726 | 328.919268 | 645.522539 | 346.130357 | 559.607642 |
| Nav3 | ENSMUSG00000020181 | 2418.30097 | 3071.93903 | 1764.66291 | 0.60422471 | -0.7268429 | 0.00022137 | 0.00892983 | 4258.05247 | 2725.57481 | 2479.86282 | 2824.26602 | 2308.22231 | 1854.8559 | 1604.30762 | 1291.26581 |
| Clmn | ENSMUSG00000021097 | 286.081314 | 199.096923 | 373.065706 | 1.7424743 | 0.80113738 | 0.00022836 | 0.00918899 | 202.944199 | 238.13683 | 210.31285 | 144.993815 | 339.529567 | 288.033791 | 533.014428 | 331.685037 |
| Gcnt2 | ENSMUSG00000021360 | 301.237519 | 405.252427 | 197.222612 | 0.53976803 | -0.8895886 | 0.00023274 | 0.0093423 | 488.010004 | 309.889849 | 534.011758 | 289.098097 | 219.92256 | 173.637392 | 265.849171 | 129.481324 |
| Ascl2 | ENSMUSG00000009248 | 37.3081217 | 18.3202183 | 56.2960251 | 2.20326142 | 1.13964068 | 0.00023445 | 0.00937061 | 12.2710446 | 11.438887 | 23.7744961 | 25.7964455 | 34.7246148 | 74.5619388 | 67.1203353 | 48.7772113 |
| Ociad1 | ENSMUSG00000029152 | 4554.2258 | 3379.59048 | 5728.86113 | 1.62179323 | 0.6975899 | 0.0002346 | 0.00937061 | 3432.11678 | 3041.70406 | 3413.46899 | 3631.07209 | 8459.302 | 4886.36048 | 4178.5699 | 5391.21213 |
| Ebf2 | ENSMUSG00000022053 | 43.6999474 | 20.1791003 | 67.2207946 | 2.25657273 | 1.17413328 | 0.00024078 | 0.00957074 | 18.8785301 | 30.1570659 | 20.1168813 | 11.5639239 | 54.0160675 | 42.8986497 | 48.6951453 | 123.273316 |
| Spg20 | ENSMUSG00000036580 | 1112.7286 | 948.615665 | 1276.84153 | 1.33371694 | 0.41545251 | 0.00024167 | 0.00958301 | 959.02933 | 963.986208 | 943.664613 | 927.782507 | 1225.00724 | 1157.24215 | 1497.70474 | 1227.41201 |
| Mylk | ENSMUSG00000022836 | 407.707008 | 523.623525 | 291.790492 | 0.59020841 | -0.7607036 | 0.00024515 | 0.00969719 | 510.66424 | 585.463037 | 502.007628 | 496.359194 | 245.966022 | 305.39753 | 422.463287 | 193.335128 |
| Mras | ENSMUSG00000032470 | 2629.23311 | 3506.81352 | 1751.65269 | 0.54967146 | -0.8633585 | 0.00024574 | 0.00969731 | 4679.98762 | 3065.62173 | 2444.20108 | 3837.44366 | 2484.7391 | 1852.81311 | 939.684695 | 1729.37385 |
| Arhgap1 | ENSMUSG00000027247 | 1932.80386 | 2270.84508 | 1594.76265 | 0.71232758 | -0.4893872 | 0.00024854 | 0.00976939 | 2739.27472 | 2279.45822 | 2106.78611 | 1957.86126 | 1410.20519 | 1690.41108 | 1546.39988 | 1732.03443 |
| Hes6 | ENSMUSG00000067071 | 846.368545 | 674.310464 | 1018.42663 | 1.47666633 | 0.56234387 | 0.00024876 | 0.00976939 | 580.514801 | 785.123611 | 685.802771 | 645.800671 | 998.332676 | 880.443715 | 1277.91854 | 917.011572 |
| Wnt4 | ENSMUSG00000036856 | 85.398601 | 35.2211458 | 135.576056 | 2.31302893 | 1.20978331 | 0.00025028 | 0.00979167 | 36.8131337 | 44.7156494 | 23.7744961 | 35.5813042 | 27.9726064 | 114.396399 | 250.056151 | 149.879067 |
| Nr0b1 | ENSMUSG00000025056 | 11.4392749 | 2.09528479 | 20.7832651 | 2.35551829 | 1.23604454 | 0.00025053 | 0.00979167 | 0 | 2.07979765 | 2.74321108 | 3.55813042 | 20.2560253 | 4.08558569 | 17.1091051 | 41.6823442 |
| Hivep1 | ENSMUSG00000021366 | 939.368044 | 1126.11094 | 752.625146 | 0.68222642 | -0.5516775 | 0.00025179 | 0.00981742 | 1375.30092 | 1201.08314 | 964.695898 | 963.363811 | 791.914132 | 820.181327 | 755.432794 | 642.97233 |
| Itsn1 | ENSMUSG00000022957 | 1646.03697 | 1962.83026 | 1329.24368 | 0.69016997 | -0.5349764 | 0.00025928 | 0.01005637 | 1941.65682 | 1948.77039 | 2249.43309 | 1711.46073 | 1137.23113 | 1662.83337 | 1191.05693 | 1325.85329 |
| Pafah1b2 | ENSMUSG00000003131 | 2805.42909 | 3096.85584 | 2514.00233 | 0.81433734 | -0.2963015 | 0.00025972 | 0.01005637 | 3148.93882 | 2961.63185 | 3151.03513 | 3125.81757 | 2537.7906 | 2605.58227 | 2393.95863 | 2518.67782 |
| Cfap69 | ENSMUSG00000040473 | 79.6267845 | 47.3533744 | 111.900195 | 1.9886122 | 0.99176196 | 0.00025977 | 0.01005637 | 39.6449133 | 34.3166611 | 58.5218365 | 56.9300867 | 65.5909391 | 114.396399 | 143.453266 | 124.160174 |
| Fkbp6 | ENSMUSG00000040013 | 13.3293958 | 0.98453196 | 25.6742596 | 2.26244995 | 1.17788588 | 0.00026098 | 0.01007967 | 0.94392651 | 2.07979765 | 0.91440369 | 0 | 77.1658107 | 12.2567571 | 2.63217001 | 10.6423006 |
| Adgrl4 | ENSMUSG00000039167 | 1116.06596 | 1485.48193 | 746.649989 | 0.55238164 | -0.8562627 | 0.00026854 | 0.01033126 | 1964.31106 | 1413.2225 | 1368.86233 | 1195.53182 | 993.509813 | 1021.39642 | 521.169663 | 450.52406 |
| Kpna1 | ENSMUSG00000022905 | 1606.7403 | 1901.31496 | 1312.16564 | 0.70138343 | -0.5117247 | 0.00026876 | 0.01033126 | 1846.32025 | 1750.14972 | 1884.58601 | 2124.20386 | 1493.15844 | 1461.61828 | 1271.33812 | 1022.54772 |
| Nlk | ENSMUSG00000017376 | 1522.53376 | 1714.84338 | 1330.22414 | 0.78018974 | -0.3581031 | 0.00027033 | 0.01035307 | 1740.60048 | 1657.59872 | 1831.5506 | 1629.62373 | 1342.68511 | 1438.12616 | 1221.32689 | 1318.75842 |
| Meox2 | ENSMUSG00000036144 | 32.1931113 | 10.0593295 | 54.326893 | 2.36745189 | 1.24333511 | 0.00027111 | 0.01035307 | 7.55141205 | 9.3590894 | 7.31522956 | 16.0115869 | 29.9017516 | 18.3851356 | 34.2182102 | 134.802475 |
| Zfp428 | ENSMUSG00000064264 | 1249.58937 | 1498.11732 | 1001.06143 | 0.68281691 | -0.5504293 | 0.00027158 | 0.01035307 | 1483.85247 | 1332.11039 | 1732.795 | 1443.71142 | 1056.20703 | 995.861511 | 762.013219 | 1190.16396 |
| C130036L24Rik | ENSMUSG00000073627 | 27.5666947 | 15.3002875 | 39.8331019 | 2.07478108 | 1.05295912 | 0.00027187 | 0.01035307 | 16.9906771 | 13.5186847 | 16.4592665 | 14.2325217 | 49.1932043 | 31.6632891 | 42.1147202 | 36.3611939 |
| Rgs4 | ENSMUSG00000038530 | 640.952685 | 909.994334 | 371.911037 | 0.49400324 | -1.0174076 | 0.00027519 | 0.01045539 | 1262.02974 | 605.221115 | 1042.42021 | 730.306269 | 447.561702 | 321.739873 | 576.445233 | 141.897342 |
| Tex101 | ENSMUSG00000062773 | 12.4502356 | 1.84473173 | 23.0557395 | 2.31648977 | 1.21194031 | 0.00027678 | 0.01049113 | 2.83177952 | 0 | 3.65761478 | 0.8895326 | 56.9097854 | 19.406532 | 5.26434003 | 10.6423006 |
| Gabpa | ENSMUSG00000008976 | 1493.09277 | 1799.43391 | 1186.75163 | 0.67520893 | -0.5665941 | 0.00028029 | 0.01057135 | 2092.68506 | 1757.42901 | 1613.92252 | 1733.69905 | 1243.33412 | 1433.01918 | 877.8287 | 1192.82453 |
| 1810013L24Rik | ENSMUSG00000022507 | 1064.39062 | 1275.87963 | 852.901607 | 0.68292929 | -0.5501919 | 0.00028083 | 0.01057135 | 1194.06703 | 1410.1028 | 1168.60792 | 1330.74078 | 721.50033 | 1074.50904 | 722.530669 | 893.066396 |
| Dock11 | ENSMUSG00000031093 | 623.979339 | 739.272751 | 508.685927 | 0.70086913 | -0.512783 | 0.00028441 | 0.0106814 | 789.122559 | 716.490289 | 743.410204 | 708.067954 | 489.038325 | 560.746636 | 401.405927 | 583.552819 |
| Doc2b | ENSMUSG00000020848 | 75.0264571 | 27.6444074 | 122.408507 | 2.33715275 | 1.22475203 | 0.00028517 | 0.01068541 | 6.60748554 | 55.1146376 | 30.1753219 | 18.6801847 | 129.252733 | 42.8986497 | 206.625346 | 110.857298 |
| Slc38a6 | ENSMUSG00000044712 | 323.578957 | 246.625658 | 400.532256 | 1.56801427 | 0.64893869 | 0.0002863 | 0.01070333 | 266.187275 | 230.857539 | 236.830557 | 252.62726 | 360.750165 | 325.825459 | 381.664652 | 533.888749 |
| Ubash3b | ENSMUSG00000032020 | 789.931108 | 1095.86592 | 483.996292 | 0.51379045 | -0.960748 | 0.00028882 | 0.01075003 | 1442.3197 | 732.088771 | 1255.47627 | 953.578953 | 787.091269 | 522.954968 | 222.418366 | 403.520566 |
| Syde1 | ENSMUSG00000032714 | 1114.37914 | 1297.13596 | 931.622315 | 0.72719901 | -0.4595779 | 0.00028887 | 0.01075003 | 1515.00204 | 1203.16294 | 1248.16104 | 1222.2178 | 875.831951 | 982.583358 | 830.449639 | 1037.62431 |
| Slc38a9 | ENSMUSG00000047789 | 692.540786 | 539.066176 | 846.015396 | 1.52383887 | 0.60771037 | 0.00029048 | 0.01078544 | 551.25308 | 520.98931 | 576.988731 | 507.033585 | 584.531016 | 843.673444 | 996.27635 | 959.580775 |
| Fam49a | ENSMUSG00000020589 | 1922.74787 | 2466.35076 | 1379.14497 | 0.59390711 | -0.7516908 | 0.00029677 | 0.01099394 | 2454.20892 | 2951.23286 | 2054.6651 | 2405.29616 | 1190.28263 | 1928.39644 | 837.030064 | 1560.87076 |
| Aif1l | ENSMUSG00000001864 | 1418.04438 | 981.727065 | 1854.36169 | 1.74887688 | 0.80642873 | 0.00029804 | 0.01101595 | 538.982035 | 1044.05842 | 1110.08609 | 1233.78172 | 2233.95022 | 1248.14643 | 2062.30521 | 1873.04491 |
| Pgam1 | ENSMUSG00000011752 | 233.866203 | 296.47086 | 171.261546 | 0.60741276 | -0.7192509 | 0.00030086 | 0.01109511 | 245.420892 | 241.256527 | 328.270926 | 370.935096 | 194.843672 | 183.851356 | 169.774966 | 136.576192 |
| Itgav | ENSMUSG00000027087 | 11850.5453 | 15985.0407 | 7716.0498 | 0.53969095 | -0.8897946 | 0.00030156 | 0.01109592 | 21245.8978 | 17213.4452 | 10667.4335 | 14813.3865 | 8792.07955 | 11276.2165 | 4503.64289 | 6292.26025 |
| Gpx3 | ENSMUSG00000018339 | 2000.34442 | 2292.34607 | 1708.34277 | 0.75154642 | -0.4120659 | 0.00030236 | 0.01110037 | 2113.45145 | 2131.79259 | 2702.97732 | 2221.16291 | 1690.89583 | 1821.14982 | 1639.84192 | 1681.4835 |
| Fbxo2 | ENSMUSG00000041556 | 937.62814 | 637.365584 | 1237.8907 | 1.77977384 | 0.83169392 | 0.00031168 | 0.01141676 | 420.991222 | 868.315517 | 609.907264 | 650.248334 | 1143.98314 | 1016.28944 | 1899.11066 | 892.179537 |
| Efhd1 | ENSMUSG00000026255 | 63.2198483 | 24.7069439 | 101.732753 | 2.30737618 | 1.20625323 | 0.00031697 | 0.01157944 | 9.43926506 | 30.1570659 | 19.2024776 | 40.0289672 | 97.421836 | 36.7702712 | 202.677091 | 70.0618126 |
| Adora2a | ENSMUSG00000020178 | 140.419913 | 198.772027 | 82.0677989 | 0.49762815 | -1.00686 | 0.00031791 | 0.01157944 | 228.430214 | 183.022193 | 139.903765 | 243.731934 | 95.4926907 | 108.268021 | 90.8098655 | 33.7006187 |
| Dusp16 | ENSMUSG00000030203 | 819.207823 | 962.030628 | 676.385018 | 0.71329712 | -0.4874249 | 0.00031824 | 0.01157944 | 880.68343 | 1096.05336 | 875.99874 | 995.386985 | 786.126696 | 659.822088 | 675.151608 | 584.439677 |
| Fmn2 | ENSMUSG00000028354 | 1566.94503 | 1995.58177 | 1138.30829 | 0.60281266 | -0.7302184 | 0.00032076 | 0.01164486 | 2765.70466 | 2129.71279 | 1447.50105 | 1639.40859 | 1298.31476 | 1302.28044 | 801.495769 | 1151.14219 |
| Ppp1r18 | ENSMUSG00000034595 | 3692.12725 | 4536.75958 | 2847.49492 | 0.6483335 | -0.625192 | 0.00032238 | 0.01167797 | 5337.90439 | 4896.88356 | 4082.8125 | 3829.43786 | 2464.48308 | 3890.49897 | 2437.38943 | 2597.60821 |
| Dtna | ENSMUSG00000024302 | 1943.21876 | 2267.14858 | 1619.28894 | 0.72349165 | -0.4669517 | 0.00032695 | 0.0117787 | 2220.11514 | 2307.53549 | 2252.1763 | 2288.76739 | 1541.38707 | 2026.4505 | 1398.99836 | 1510.31983 |
| Dll4 | ENSMUSG00000027314 | 330.73072 | 451.775413 | 209.686026 | 0.52817456 | -0.9209133 | 0.00032733 | 0.0117787 | 599.393331 | 404.520642 | 357.531845 | 445.655835 | 268.151192 | 256.370502 | 221.102281 | 93.1201306 |
| Lrrtm3 | ENSMUSG00000042846 | 2267.88424 | 2946.66136 | 1589.10713 | 0.57958862 | -0.7868988 | 0.00032941 | 0.01181338 | 4158.94019 | 2832.68439 | 2207.37052 | 2587.65035 | 1541.38707 | 2087.73429 | 966.006395 | 1761.30076 |
| 9630001P10Rik | ENSMUSG00000097825 | 161.065118 | 84.623928 | 237.506308 | 2.12326218 | 1.08628252 | 0.00032974 | 0.01181338 | 112.327254 | 82.152007 | 62.1794512 | 81.8369997 | 209.312261 | 167.509013 | 465.894092 | 107.309865 |
| Triobp | ENSMUSG00000033088 | 774.92477 | 929.913848 | 619.935691 | 0.68084685 | -0.5545978 | 0.00033491 | 0.01197257 | 1082.6837 | 822.559969 | 907.088465 | 907.323257 | 717.642039 | 597.516907 | 685.680289 | 478.903529 |
| Micall2 | ENSMUSG00000036718 | 437.082748 | 596.525343 | 277.640154 | 0.52964327 | -0.9169071 | 0.00033643 | 0.01199732 | 773.075809 | 370.203981 | 397.765607 | 845.055975 | 355.927302 | 327.868251 | 188.200156 | 238.564906 |
| Tie1 | ENSMUSG00000033191 | 694.152147 | 911.643464 | 476.660831 | 0.56775127 | -0.8166691 | 0.00033707 | 0.01199732 | 1031.71167 | 785.123611 | 947.322228 | 882.416344 | 587.424734 | 659.822088 | 409.302437 | 250.094065 |
| Usf3 | ENSMUSG00000068284 | 752.150287 | 886.616822 | 617.683753 | 0.7072945 | -0.499617 | 0.00034058 | 0.01209461 | 995.842464 | 839.19835 | 783.643966 | 927.782507 | 670.37798 | 664.929071 | 634.352973 | 501.074989 |
| Vps26b | ENSMUSG00000031988 | 2176.30525 | 2480.24349 | 1872.367 | 0.76101162 | -0.3940096 | 0.00034129 | 0.01209461 | 2428.7229 | 2635.10362 | 2529.24062 | 2327.90683 | 1785.42394 | 1987.63744 | 1641.158 | 2075.24863 |
| Sipa1 | ENSMUSG00000056917 | 1170.17353 | 1467.58003 | 872.76703 | 0.62198199 | -0.6850553 | 0.00034384 | 0.01213693 | 1490.45995 | 1401.78361 | 1370.69114 | 1607.38542 | 982.899514 | 1216.48314 | 618.559953 | 673.125516 |
| Asl | ENSMUSG00000025533 | 411.230858 | 317.609045 | 504.85267 | 1.54114827 | 0.62400567 | 0.00034397 | 0.01213693 | 304.888261 | 389.962058 | 329.18533 | 246.400532 | 596.105888 | 449.414426 | 440.888477 | 533.00189 |
| Il18bp | ENSMUSG00000070427 | 491.660647 | 309.386452 | 673.934841 | 1.90322268 | 0.92844437 | 0.00034497 | 0.01214206 | 216.15917 | 380.602969 | 273.406705 | 367.376966 | 633.72422 | 637.351367 | 335.601677 | 1089.0621 |
| Ccdc62 | ENSMUSG00000061882 | 147.299496 | 91.2158255 | 203.383166 | 1.92412184 | 0.94420016 | 0.0003456 | 0.01214206 | 56.6355904 | 128.947454 | 83.2107362 | 96.0695213 | 165.906493 | 223.685816 | 285.590446 | 138.349908 |
| Igfbp7 | ENSMUSG00000036256 | 2375.62178 | 3069.39549 | 1681.84807 | 0.58600889 | -0.7710056 | 0.00034762 | 0.0121818 | 3749.27608 | 2648.6223 | 3331.17266 | 2548.51091 | 2172.21757 | 1877.32662 | 1768.81825 | 909.029846 |
| Stac2 | ENSMUSG00000017400 | 315.342082 | 447.229602 | 183.454563 | 0.49729268 | -1.0078329 | 0.0003486 | 0.0121818 | 509.720313 | 239.176729 | 332.842945 | 707.178421 | 235.355723 | 161.380635 | 239.527471 | 97.5544225 |
| Tubb6 | ENSMUSG00000001473 | 2493.8148 | 3814.22316 | 1173.40644 | 0.45231985 | -1.1445848 | 0.00034946 | 0.0121818 | 4421.35175 | 2675.65967 | 4193.45534 | 3966.42589 | 2177.04043 | 1238.95386 | 523.801833 | 753.829629 |
| Gab2 | ENSMUSG00000004508 | 1719.63516 | 2094.24691 | 1345.02341 | 0.66097542 | -0.5973315 | 0.00034971 | 0.0121818 | 2091.74114 | 2170.26884 | 2093.07006 | 2021.90761 | 1778.67194 | 1430.97639 | 958.109885 | 1212.33541 |
| Slc2a12 | ENSMUSG00000037490 | 234.236362 | 157.036471 | 311.436253 | 1.80118232 | 0.84894422 | 0.00035291 | 0.01222049 | 147.252535 | 222.538348 | 112.471654 | 145.883347 | 331.812986 | 205.300681 | 407.986352 | 300.644993 |
| Cxxc5 | ENSMUSG00000046668 | 3214.48445 | 3894.29283 | 2534.67607 | 0.66806741 | -0.5819344 | 0.00035307 | 0.01222049 | 4435.51065 | 4072.24379 | 3935.5935 | 3133.82337 | 2510.78257 | 3280.72531 | 2071.5178 | 2275.67862 |
| Col4a2 | ENSMUSG00000031503 | 8440.41213 | 11514.4954 | 5366.32884 | 0.53026817 | -0.9152059 | 0.00035507 | 0.01226391 | 15383.1703 | 9497.39595 | 11817.7534 | 9359.66207 | 7392.48466 | 5437.91455 | 6367.21926 | 2267.6969 |
| Ptprk | ENSMUSG00000019889 | 989.179678 | 1270.23195 | 708.127407 | 0.59330668 | -0.7531501 | 0.00035883 | 0.01234663 | 1272.41293 | 1445.45936 | 1324.97095 | 1038.08455 | 458.172001 | 1079.61602 | 665.939013 | 628.782596 |
| Postn | ENSMUSG00000027750 | 1287.16753 | 1741.78122 | 832.553845 | 0.53780612 | -0.8948419 | 0.00035898 | 0.01234663 | 1918.05866 | 1169.88618 | 1828.80739 | 2050.37265 | 1202.82207 | 725.191459 | 1019.96588 | 382.235965 |
| Diaph1 | ENSMUSG00000024456 | 1254.78071 | 1474.70796 | 1034.85347 | 0.71256988 | -0.4888966 | 0.00036113 | 0.01239452 | 1762.31079 | 1450.65886 | 1354.23187 | 1331.63031 | 971.324642 | 1197.07661 | 906.78257 | 1064.23006 |
| Pnpla2 | ENSMUSG00000025509 | 843.10948 | 702.082581 | 984.136379 | 1.38305964 | 0.46786336 | 0.00036235 | 0.0124003 | 811.776795 | 646.817068 | 665.68589 | 684.050573 | 1098.64823 | 946.834483 | 1042.33933 | 848.723476 |
| Shisa2 | ENSMUSG00000044461 | 73.773701 | 31.0372776 | 116.510124 | 2.26795267 | 1.18139053 | 0.00036282 | 0.0124003 | 7.55141205 | 55.1146376 | 36.5761478 | 24.9069129 | 202.560253 | 86.8186958 | 65.8042503 | 110.857298 |
| Lrrtm2 | ENSMUSG00000071862 | 933.233727 | 1216.22078 | 650.246678 | 0.57708479 | -0.7931448 | 0.00036418 | 0.01242091 | 1434.76829 | 1359.14776 | 1182.32398 | 888.643072 | 716.677467 | 749.704974 | 340.866017 | 793.738256 |
| Zdhhc3 | ENSMUSG00000025786 | 2113.50385 | 2539.77426 | 1687.23344 | 0.67966522 | -0.5571038 | 0.0003664 | 0.01247042 | 2454.20892 | 2516.55515 | 2572.21759 | 2616.11539 | 2005.34651 | 2038.70726 | 1359.51581 | 1345.36417 |
| Dusp3 | ENSMUSG00000003518 | 1360.70484 | 1638.87025 | 1082.53943 | 0.67645585 | -0.5639323 | 0.000368 | 0.01248051 | 1759.47901 | 1908.21434 | 1223.47214 | 1664.3155 | 982.899514 | 1308.40882 | 989.695925 | 1049.15347 |
| Angpt1 | ENSMUSG00000022309 | 240.589654 | 152.419898 | 328.75941 | 1.89060951 | 0.91885142 | 0.00036822 | 0.01248051 | 160.467506 | 210.059562 | 93.2691769 | 145.883347 | 339.529567 | 179.76577 | 389.561162 | 406.181141 |
| Add3 | ENSMUSG00000025026 | 4088.92265 | 5571.56951 | 2606.27578 | 0.53188481 | -0.9108143 | 0.00037656 | 0.01272597 | 8758.69405 | 4867.76639 | 4161.45121 | 4498.36638 | 2112.41407 | 4369.53389 | 1626.68107 | 2316.47411 |
| Rbak | ENSMUSG00000061898 | 651.477567 | 521.625212 | 781.329923 | 1.46509999 | 0.55099913 | 0.00037702 | 0.01272597 | 502.168901 | 631.218585 | 440.742581 | 512.37078 | 640.476229 | 770.132902 | 906.78257 | 807.92799 |
| Ndst1 | ENSMUSG00000054008 | 3097.68083 | 3811.14645 | 2384.21521 | 0.64716336 | -0.6277982 | 0.00038187 | 0.01285359 | 4788.53917 | 3469.10247 | 2996.50091 | 3990.44327 | 3076.02213 | 2417.64533 | 1930.6967 | 2112.49668 |
| Sec63 | ENSMUSG00000019802 | 2808.62361 | 3171.87405 | 2445.37316 | 0.77540447 | -0.3669791 | 0.00038237 | 0.01285359 | 3112.12569 | 3165.45202 | 2908.71815 | 3501.20033 | 2289.89543 | 2534.08452 | 2632.17001 | 2325.34269 |
| Sfrp1 | ENSMUSG00000031548 | 685.043125 | 251.959447 | 1118.1268 | 2.30203475 | 1.20290961 | 0.00038367 | 0.01287081 | 229.374141 | 265.1742 | 164.592665 | 348.696781 | 2564.79863 | 455.542804 | 827.817469 | 624.348304 |
| Gtf2e1 | ENSMUSG00000022828 | 652.688283 | 765.599724 | 539.776842 | 0.71488901 | -0.4842088 | 0.00038671 | 0.0129461 | 904.281593 | 694.652413 | 758.955067 | 704.509823 | 470.711445 | 587.302942 | 569.864808 | 531.228174 |
| Gja1 | ENSMUSG00000050953 | 300.715758 | 381.788435 | 219.643081 | 0.60572269 | -0.7232706 | 0.00038865 | 0.01295652 | 413.43981 | 360.844891 | 437.084966 | 315.784075 | 189.056236 | 229.814195 | 300.067382 | 159.63451 |
| Zcchc8 | ENSMUSG00000029427 | 1690.18236 | 1363.76626 | 2016.59846 | 1.44980259 | 0.53585647 | 0.00038909 | 0.01295652 | 1408.33835 | 1334.19019 | 1288.39481 | 1424.1417 | 2595.66496 | 1827.2782 | 1580.61809 | 2062.83261 |
| Pecam1 | ENSMUSG00000020717 | 849.720746 | 1167.10253 | 532.338966 | 0.52505983 | -0.9294463 | 0.00039047 | 0.01296406 | 1451.75897 | 1022.22054 | 1273.76435 | 920.666246 | 677.129989 | 840.609255 | 357.975122 | 253.641499 |
| Zfp142 | ENSMUSG00000026135 | 926.910256 | 1084.56243 | 769.258084 | 0.71907191 | -0.475792 | 0.00039197 | 0.01296406 | 1306.39428 | 1063.8165 | 962.867091 | 1005.17184 | 842.071909 | 685.356999 | 797.547514 | 752.055912 |
| Exog | ENSMUSG00000042787 | 466.783048 | 559.669236 | 373.896859 | 0.68309566 | -0.5498405 | 0.00039337 | 0.01296406 | 572.019463 | 491.872143 | 610.821668 | 563.963672 | 374.254182 | 466.778165 | 323.756912 | 330.798178 |
| Sdf2l1 | ENSMUSG00000022769 | 452.792965 | 534.411913 | 371.174017 | 0.70694747 | -0.5003251 | 0.00039438 | 0.01296406 | 574.851242 | 517.869614 | 545.899006 | 499.027791 | 443.703411 | 364.638523 | 317.176487 | 359.177647 |
| Arhgdib | ENSMUSG00000030220 | 473.858442 | 595.832985 | 351.883899 | 0.61895933 | -0.6920835 | 0.00039493 | 0.01296406 | 728.711263 | 474.193863 | 654.713045 | 525.71377 | 442.738839 | 379.959469 | 332.969507 | 251.867782 |
| Arl13b | ENSMUSG00000022911 | 349.351229 | 415.656216 | 283.046242 | 0.69472498 | -0.5254861 | 0.00039497 | 0.01296406 | 447.421164 | 380.602969 | 400.508818 | 434.091911 | 312.521533 | 317.654287 | 242.159641 | 259.849507 |
| Prss23 | ENSMUSG00000039405 | 849.66832 | 310.351943 | 1388.9847 | 2.29939667 | 1.20125537 | 0.00039598 | 0.01296406 | 320.935012 | 255.81511 | 300.838816 | 363.818835 | 1844.26288 | 598.538303 | 2687.44558 | 425.692026 |
| Spred2 | ENSMUSG00000045671 | 1750.91563 | 2145.15688 | 1356.67438 | 0.65302752 | -0.6147843 | 0.00040109 | 0.01310525 | 2737.38687 | 2074.59815 | 1754.74069 | 2013.90182 | 1730.4433 | 1415.65544 | 1105.51141 | 1175.08736 |
| Slc27a3 | ENSMUSG00000027932 | 399.384829 | 305.244585 | 493.525074 | 1.55861178 | 0.64026163 | 0.00040278 | 0.01313429 | 235.0377 | 271.413593 | 335.586156 | 378.94089 | 467.817727 | 420.815326 | 497.480133 | 587.98711 |
| Ctsl | ENSMUSG00000021477 | 3257.0241 | 3708.18321 | 2805.86498 | 0.76251502 | -0.3911623 | 0.00040482 | 0.01316404 | 4036.22974 | 3399.42925 | 3587.20569 | 3809.86815 | 3114.60503 | 2879.31651 | 2490.03283 | 2739.50556 |
| Pcdhb19 | ENSMUSG00000043313 | 182.885907 | 230.489486 | 135.282328 | 0.61615265 | -0.6986403 | 0.00040531 | 0.01316404 | 300.168629 | 189.261586 | 236.830557 | 195.697173 | 130.217306 | 151.16667 | 132.924586 | 126.820749 |
| Lama4 | ENSMUSG00000019846 | 2278.40162 | 2898.13333 | 1658.66991 | 0.60509183 | -0.724774 | 0.00040746 | 0.0132063 | 3213.12583 | 2505.11626 | 2740.46787 | 3133.82337 | 2524.28658 | 1351.30747 | 1637.20975 | 1121.87586 |
| Fam19a2 | ENSMUSG00000044071 | 951.141924 | 1224.54153 | 677.742321 | 0.59088003 | -0.7590629 | 0.00040916 | 0.0132063 | 1459.31038 | 1166.76648 | 1120.14453 | 1151.94472 | 350.139866 | 744.597991 | 787.018834 | 829.212592 |
| B3galt1 | ENSMUSG00000034780 | 395.749108 | 471.110305 | 320.387912 | 0.69460149 | -0.5257426 | 0.00041024 | 0.0132063 | 483.290371 | 553.226174 | 432.512948 | 415.411727 | 348.210721 | 302.333341 | 286.906531 | 344.101054 |
| Cyp4f13 | ENSMUSG00000024055 | 381.437386 | 256.541974 | 506.332798 | 1.7908229 | 0.84062267 | 0.0004105 | 0.0132063 | 244.476965 | 270.373694 | 190.195969 | 321.12127 | 355.927302 | 479.034922 | 802.811854 | 387.557115 |
| Bace1 | ENSMUSG00000032086 | 1523.64696 | 1793.74317 | 1253.55075 | 0.71045477 | -0.4931853 | 0.00041065 | 0.0132063 | 1961.47928 | 1927.97242 | 1718.16454 | 1567.35645 | 1320.49994 | 1229.76129 | 1004.17286 | 1459.7689 |
| Ammecr1l | ENSMUSG00000041915 | 1065.8478 | 1237.60413 | 894.091464 | 0.73165776 | -0.4507591 | 0.00041346 | 0.01325082 | 1378.1327 | 1190.68415 | 1254.56187 | 1127.03781 | 853.646781 | 958.069844 | 758.064964 | 1006.58427 |
| Pcyt1b | ENSMUSG00000035246 | 2042.1526 | 2803.8357 | 1280.46951 | 0.52597878 | -0.9269235 | 0.00041366 | 0.01325082 | 3299.02314 | 3252.80352 | 2474.3764 | 2189.13974 | 1428.53207 | 1889.58338 | 461.945837 | 1341.81674 |
| Gphn | ENSMUSG00000047454 | 1708.50591 | 1965.66948 | 1451.34233 | 0.74595427 | -0.4228409 | 0.0004147 | 0.0132583 | 1820.83423 | 2156.75016 | 2013.51694 | 1871.5766 | 1380.30344 | 1607.67797 | 1247.64859 | 1569.73934 |
| Lias | ENSMUSG00000029199 | 950.347644 | 726.736353 | 1173.95893 | 1.55792606 | 0.63962676 | 0.00041573 | 0.01326508 | 728.711263 | 681.133729 | 654.713045 | 842.387377 | 1676.42724 | 1019.35363 | 968.638565 | 1031.4163 |
| Ppm1f | ENSMUSG00000026181 | 1432.99906 | 1695.53152 | 1170.46659 | 0.70277488 | -0.5088655 | 0.00042059 | 0.01339403 | 1699.06771 | 1905.09464 | 1590.14803 | 1587.8157 | 1382.23258 | 1310.45161 | 948.89729 | 1040.28489 |
| Diras2 | ENSMUSG00000047842 | 370.412564 | 489.454863 | 251.370264 | 0.56332743 | -0.8279544 | 0.00042387 | 0.01347231 | 447.421164 | 747.687253 | 341.072578 | 421.638455 | 202.560253 | 314.590098 | 194.780581 | 293.550126 |
| Ttc9 | ENSMUSG00000042734 | 1086.10508 | 1376.41775 | 795.792401 | 0.61022402 | -0.7125891 | 0.00042796 | 0.01354687 | 1355.47846 | 1596.24469 | 1540.77023 | 1013.17764 | 719.571185 | 933.556329 | 514.589238 | 1015.45285 |
| Scx | ENSMUSG00000034161 | 86.1469571 | 37.8116917 | 134.482222 | 2.23104746 | 1.1577212 | 0.0004281 | 0.01354687 | 14.1588976 | 31.1969647 | 66.7514697 | 39.1394346 | 263.328329 | 74.5619388 | 118.447651 | 81.5909716 |
| Irf2bpl | ENSMUSG00000034168 | 1861.44426 | 2255.60522 | 1467.28329 | 0.66842836 | -0.5811552 | 0.0004287 | 0.01354687 | 2831.77952 | 2273.21883 | 1926.64858 | 1990.77397 | 1734.3016 | 1520.85927 | 1113.40792 | 1500.56439 |
| Snhg20 | ENSMUSG00000086859 | 732.64843 | 542.194193 | 923.102666 | 1.61917821 | 0.69526178 | 0.00044069 | 0.01388974 | 518.215652 | 463.794875 | 593.447998 | 593.318248 | 655.909391 | 890.65768 | 1347.67105 | 798.172548 |
| Necap1 | ENSMUSG00000030327 | 1190.23486 | 1357.59908 | 1022.87065 | 0.76011538 | -0.3957097 | 0.00044125 | 0.01388974 | 1333.76815 | 1371.62655 | 1354.23187 | 1370.76974 | 958.785198 | 1042.84575 | 906.78257 | 1183.06909 |
| Inpp5k | ENSMUSG00000006127 | 780.808583 | 662.871384 | 898.745781 | 1.34154631 | 0.42389686 | 0.00044621 | 0.01401471 | 680.571011 | 690.492818 | 635.510568 | 644.911139 | 810.241012 | 977.476376 | 1000.22461 | 807.041132 |
| Tcf24 | ENSMUSG00000099032 | 101.329622 | 41.5391534 | 161.120091 | 2.25552783 | 1.17346509 | 0.00044694 | 0.01401471 | 31.1495747 | 69.6732211 | 14.6304591 | 50.7033585 | 280.690636 | 106.225228 | 47.3790602 | 210.185438 |
| Zswim5 | ENSMUSG00000033948 | 766.473868 | 911.559858 | 621.387879 | 0.6958064 | -0.5232421 | 0.00045845 | 0.01434814 | 873.132018 | 1099.17306 | 876.913143 | 797.021214 | 614.432768 | 706.806324 | 505.376643 | 658.935781 |
| Ccnd1 | ENSMUSG00000070348 | 26103.0094 | 36013.88 | 16192.1388 | 0.52267056 | -0.9360262 | 0.00046096 | 0.01437519 | 45071.5467 | 29678.7124 | 36694.1059 | 32611.1548 | 27667.8014 | 15087.0465 | 13632.0085 | 8381.69861 |
| Jak3 | ENSMUSG00000031805 | 360.363863 | 491.244107 | 229.483618 | 0.53326052 | -0.9070876 | 0.00046108 | 0.01437519 | 757.029058 | 295.331266 | 587.047172 | 325.568933 | 260.434611 | 291.09798 | 193.464496 | 172.937385 |
| Map7 | ENSMUSG00000019996 | 577.152689 | 433.779849 | 720.525529 | 1.5899808 | 0.66900935 | 0.00046614 | 0.0145054 | 342.645322 | 486.672649 | 336.50056 | 569.300867 | 602.857896 | 651.650917 | 843.610489 | 783.982814 |
| Rara | ENSMUSG00000037992 | 1107.90029 | 1461.28296 | 754.517615 | 0.56530295 | -0.8229039 | 0.00046947 | 0.01458136 | 2075.69439 | 858.956427 | 1142.09021 | 1768.39082 | 962.643488 | 817.117137 | 715.950244 | 522.35959 |
| N4bp2 | ENSMUSG00000037795 | 1181.31168 | 959.004181 | 1403.61917 | 1.43659158 | 0.52264997 | 0.0004743 | 0.01470315 | 1040.20701 | 1084.61447 | 873.255528 | 837.939714 | 1678.35638 | 1151.11377 | 1289.76331 | 1495.24324 |
| Cdc42ep1 | ENSMUSG00000049521 | 2084.75298 | 2675.27785 | 1494.22811 | 0.59568236 | -0.7473849 | 0.00047968 | 0.01483834 | 2962.9853 | 3444.1449 | 2427.74181 | 1866.23941 | 1555.85566 | 2111.2264 | 1026.54631 | 1283.28409 |
| Eng | ENSMUSG00000026814 | 1180.36488 | 1568.54402 | 792.185729 | 0.55784279 | -0.8420695 | 0.00048047 | 0.01483834 | 2236.16189 | 1249.95838 | 1612.09371 | 1175.9621 | 1155.55801 | 826.309705 | 756.748879 | 430.126318 |
| Kif3b | ENSMUSG00000027475 | 1336.10643 | 1508.88572 | 1163.32713 | 0.77600654 | -0.3658593 | 0.00048165 | 0.01484675 | 1633.93678 | 1599.36439 | 1348.74545 | 1453.49628 | 1134.33742 | 1236.91107 | 1134.46528 | 1147.59475 |
| Rgs20 | ENSMUSG00000002459 | 412.597229 | 574.297867 | 250.896591 | 0.51632559 | -0.953647 | 0.00048626 | 0.01494784 | 745.70194 | 496.031738 | 573.331117 | 482.126672 | 230.532859 | 378.938072 | 90.8098655 | 303.305568 |
| Atp2c1 | ENSMUSG00000032570 | 2198.20296 | 2626.34227 | 1770.06366 | 0.6886964 | -0.53806 | 0.00048676 | 0.01494784 | 2935.61143 | 2686.05866 | 2328.07181 | 2555.62717 | 1474.83156 | 2003.97978 | 1446.37742 | 2155.06588 |
| Il1r1 | ENSMUSG00000026072 | 341.583686 | 467.144952 | 216.02242 | 0.53093482 | -0.9133933 | 0.00049468 | 0.01512408 | 786.29078 | 337.967117 | 449.886618 | 294.435292 | 269.115765 | 245.135141 | 207.941431 | 141.897342 |
| Cfl2 | ENSMUSG00000062929 | 3207.75528 | 3826.48709 | 2589.02347 | 0.69083927 | -0.533578 | 0.00049481 | 0.01512408 | 4878.21218 | 3013.62679 | 3788.37451 | 3625.7349 | 2828.12696 | 2666.86606 | 2238.6606 | 2622.44025 |
| Wrb | ENSMUSG00000023147 | 870.548564 | 1039.86359 | 701.233535 | 0.68902879 | -0.5373638 | 0.00049528 | 0.01512408 | 1064.7491 | 955.667018 | 1166.77911 | 972.259137 | 584.531016 | 892.700473 | 609.347358 | 718.355293 |
| Dact2 | ENSMUSG00000048826 | 70.2501147 | 24.5881575 | 115.912072 | 2.2741572 | 1.18533198 | 0.00050429 | 0.01534794 | 42.4766928 | 39.5161553 | 12.8016517 | 3.55813042 | 164.94192 | 41.8772533 | 156.614116 | 100.214998 |
| Runx1 | ENSMUSG00000022952 | 256.563978 | 348.162866 | 164.96509 | 0.53817577 | -0.8938507 | 0.00050449 | 0.01534794 | 546.533447 | 213.179259 | 344.730193 | 288.208564 | 226.674569 | 166.487617 | 146.085436 | 120.612741 |
| Firre | ENSMUSG00000085396 | 2317.9635 | 1814.75922 | 2821.16778 | 1.50918389 | 0.5937686 | 0.00050711 | 0.01539901 | 1592.40402 | 2135.95218 | 1966.88235 | 1563.79832 | 2048.75227 | 2846.63183 | 3541.58475 | 2847.70228 |
| Usp14 | ENSMUSG00000047879 | 1544.29321 | 1806.58199 | 1282.00443 | 0.72014798 | -0.4736347 | 0.00052013 | 0.0157333 | 1850.09595 | 1608.72348 | 1909.27491 | 1858.23361 | 1580.93455 | 1203.20498 | 1076.55754 | 1267.32063 |
| Hic1 | ENSMUSG00000043099 | 308.539448 | 395.759157 | 221.319738 | 0.59682922 | -0.7446099 | 0.00052084 | 0.0157333 | 351.14066 | 462.754976 | 512.066069 | 257.074923 | 206.418544 | 271.691448 | 196.096666 | 211.072296 |
| Pxdc1 | ENSMUSG00000021411 | 4064.51972 | 5135.43005 | 2993.60939 | 0.61439763 | -0.7027555 | 0.00052173 | 0.0157333 | 6139.298 | 4779.37499 | 5497.39501 | 4125.65222 | 2089.26432 | 4004.89537 | 2304.46485 | 3575.81302 |
| Ptgs2 | ENSMUSG00000032487 | 113.676866 | 159.827866 | 67.5258662 | 0.50994769 | -0.9715788 | 0.00052271 | 0.0157333 | 175.57033 | 178.862597 | 171.907895 | 112.970641 | 50.1577769 | 48.0056318 | 51.3273153 | 120.612741 |
| Sh3pxd2a | ENSMUSG00000053617 | 5132.92002 | 6805.57036 | 3460.26968 | 0.56079693 | -0.8344497 | 0.00052391 | 0.0157333 | 9611.05969 | 4849.04821 | 6281.03898 | 6481.13456 | 5507.70974 | 3480.91901 | 2465.02722 | 2387.42278 |
| Cbx7 | ENSMUSG00000053411 | 634.966809 | 429.959461 | 839.974158 | 1.77498893 | 0.82781002 | 0.00052511 | 0.0157333 | 416.271589 | 472.114065 | 480.976343 | 350.475846 | 464.924009 | 753.790559 | 1368.72841 | 772.453655 |
| Garnl3 | ENSMUSG00000038860 | 268.876076 | 169.632143 | 368.120009 | 1.88250149 | 0.91265101 | 0.0005259 | 0.0157333 | 180.289963 | 117.508567 | 196.596794 | 184.133249 | 283.584354 | 216.536041 | 623.824293 | 348.535346 |
| Slc25a33 | ENSMUSG00000028982 | 200.722472 | 148.859721 | 252.585223 | 1.61229855 | 0.68911892 | 0.00052663 | 0.0157333 | 117.046887 | 155.984823 | 145.390187 | 177.016988 | 274.903201 | 215.514645 | 307.963892 | 211.959154 |
| Qpct | ENSMUSG00000024084 | 1439.6023 | 1793.78328 | 1085.42132 | 0.6317927 | -0.6624768 | 0.00052707 | 0.0157333 | 1553.70303 | 2342.89205 | 1494.13564 | 1784.40241 | 845.9302 | 1484.089 | 919.94342 | 1091.72267 |
| Cpm | ENSMUSG00000020183 | 267.873454 | 176.035964 | 359.710945 | 1.8214552 | 0.86509151 | 0.00052775 | 0.0157333 | 243.533039 | 135.186847 | 158.191839 | 167.23213 | 345.317003 | 327.868251 | 547.491363 | 218.167163 |
| Zcchc4 | ENSMUSG00000029179 | 484.692839 | 398.823245 | 570.562434 | 1.40633139 | 0.4919366 | 0.00053086 | 0.01579194 | 411.551957 | 315.089343 | 409.652855 | 458.998824 | 591.283024 | 563.810825 | 579.077403 | 548.078483 |
| Tmem176b | ENSMUSG00000029810 | 8868.83913 | 11821.3872 | 5916.29107 | 0.55561571 | -0.8478407 | 0.00053165 | 0.01579194 | 18684.0813 | 10004.8666 | 9256.5086 | 9340.09235 | 6619.86198 | 7894.37294 | 3462.61965 | 5688.30969 |
| Pdlim5 | ENSMUSG00000028273 | 4182.68121 | 5154.874 | 3210.48842 | 0.64600233 | -0.6303887 | 0.00053429 | 0.01581689 | 6085.49419 | 4816.81135 | 4805.19142 | 4911.99904 | 4124.51258 | 3363.45842 | 2093.89125 | 3260.09143 |
| Mtmr10 | ENSMUSG00000030522 | 1796.14465 | 1471.40015 | 2120.88916 | 1.41685918 | 0.50269637 | 0.00053519 | 0.01581689 | 1319.60926 | 1840.62092 | 1204.26967 | 1521.10075 | 1872.23548 | 2200.08789 | 2218.91932 | 2192.31393 |
| Inafm1 | ENSMUSG00000091811 | 441.803956 | 352.508573 | 531.099338 | 1.46923037 | 0.55506062 | 0.00053539 | 0.01581689 | 287.897584 | 360.844891 | 419.711296 | 341.58052 | 492.896616 | 494.355868 | 639.617313 | 497.527555 |
| Cotl1 | ENSMUSG00000031827 | 3741.72656 | 4754.67528 | 2728.77784 | 0.60775174 | -0.718446 | 0.00054208 | 0.0159856 | 5063.22178 | 4215.74983 | 5666.5597 | 4073.1698 | 3486.93007 | 3348.13747 | 1597.7272 | 2482.31662 |
| Col7a1 | ENSMUSG00000025650 | 465.292511 | 613.292096 | 317.292925 | 0.56782586 | -0.8164795 | 0.00055849 | 0.01643972 | 763.636543 | 679.053931 | 522.12451 | 488.3534 | 304.804952 | 457.585597 | 169.774966 | 337.006187 |
| Hnrnpa1 | ENSMUSG00000046434 | 2091.94003 | 1740.67223 | 2443.20783 | 1.38403293 | 0.46887827 | 0.0005607 | 0.01645993 | 1613.1704 | 1468.33714 | 1967.79675 | 1913.38463 | 2219.48163 | 2272.60704 | 2429.49292 | 2851.24971 |
| Gm5803 | ENSMUSG00000046434 | 2091.94003 | 1740.67223 | 2443.20783 | 1.38403293 | 0.46887827 | 0.0005607 | 0.01645993 | 1613.1704 | 1468.33714 | 1967.79675 | 1913.38463 | 2219.48163 | 2272.60704 | 2429.49292 | 2851.24971 |
| Gnaz | ENSMUSG00000040009 | 575.722599 | 711.347294 | 440.097904 | 0.64300776 | -0.6370919 | 0.00056145 | 0.01645993 | 706.057027 | 772.644825 | 667.514697 | 699.172628 | 314.450679 | 602.623889 | 377.716397 | 465.600653 |
| Nab2 | ENSMUSG00000025402 | 1335.85571 | 1620.37767 | 1051.33375 | 0.66783105 | -0.5824449 | 0.00056288 | 0.01645993 | 1502.731 | 1802.14466 | 1901.04528 | 1275.58976 | 1157.48716 | 1206.26917 | 779.122324 | 1062.45635 |
| Kcna3 | ENSMUSG00000047959 | 33.1551666 | 50.9096322 | 15.4007009 | 0.46148197 | -1.1156538 | 0.00056711 | 0.01654433 | 67.9627084 | 55.1146376 | 51.2066069 | 29.354576 | 15.4331621 | 14.2995499 | 5.26434003 | 26.6057516 |
| Lars | ENSMUSG00000024493 | 2500.08165 | 2828.76624 | 2171.39706 | 0.77279752 | -0.3718376 | 0.00056903 | 0.01657072 | 2723.22797 | 2678.77937 | 2726.75182 | 3186.30579 | 2343.9115 | 2275.67123 | 2076.78214 | 1989.22336 |
| Fam89a | ENSMUSG00000043068 | 1199.39124 | 802.440773 | 1596.34171 | 1.79319838 | 0.8425351 | 0.00057078 | 0.0165923 | 974.132154 | 894.312987 | 846.737821 | 494.580128 | 1992.80706 | 982.583358 | 2300.51659 | 1109.45984 |
| Dnm2 | ENSMUSG00000033335 | 2576.3841 | 3014.67205 | 2138.09615 | 0.71990787 | -0.4741158 | 0.00057423 | 0.01666297 | 3483.08881 | 3006.3475 | 2579.53282 | 2989.71909 | 2131.70552 | 2325.71965 | 1735.91612 | 2359.04331 |
| Oxr1 | ENSMUSG00000022307 | 3916.26333 | 5299.21162 | 2533.31503 | 0.54197959 | -0.8836896 | 0.00057784 | 0.01668502 | 8024.31923 | 3464.94288 | 4419.31306 | 5288.27134 | 4199.74925 | 2475.86493 | 1663.53145 | 1794.11452 |
| Begain | ENSMUSG00000040867 | 1006.16152 | 690.99716 | 1321.32587 | 1.74960275 | 0.80702739 | 0.00057834 | 0.01668502 | 660.748554 | 670.734741 | 765.355892 | 667.149454 | 826.638747 | 948.877276 | 2255.7697 | 1254.01776 |
| Chst3 | ENSMUSG00000057337 | 1255.90332 | 1599.18795 | 912.618685 | 0.60536867 | -0.7241141 | 0.00057894 | 0.01668502 | 2095.51684 | 1786.54618 | 1143.91902 | 1370.76974 | 713.783749 | 1159.28494 | 1096.29881 | 681.107241 |
| Tsc22d2 | ENSMUSG00000027806 | 1794.60222 | 2187.7035 | 1401.50094 | 0.66079821 | -0.5977183 | 0.00058066 | 0.01668502 | 2914.84505 | 1814.62345 | 2182.68162 | 1838.66389 | 1744.91189 | 1372.75679 | 1250.28076 | 1238.05431 |
| Ddc | ENSMUSG00000020182 | 149.980727 | 21.1734946 | 278.78796 | 2.18036381 | 1.12456888 | 0.00058111 | 0.01668502 | 3.77570602 | 32.2368635 | 23.7744961 | 24.9069129 | 190.020809 | 97.0326601 | 760.697134 | 67.4012374 |
| Chad | ENSMUSG00000039084 | 28.2808924 | 7.04561644 | 49.5161684 | 2.24815807 | 1.16874348 | 0.00058112 | 0.01668502 | 12.2710446 | 11.438887 | 0.91440369 | 3.55813042 | 119.607007 | 29.6204962 | 35.5342952 | 13.3028758 |
| Nol4 | ENSMUSG00000041923 | 572.495348 | 708.851746 | 436.13895 | 0.64004399 | -0.643757 | 0.00058345 | 0.01672248 | 807.057163 | 735.208468 | 743.410204 | 549.73115 | 299.017516 | 538.275914 | 451.417157 | 455.845211 |
| Car5b | ENSMUSG00000031373 | 83.6285235 | 43.0799916 | 124.177056 | 2.10333298 | 1.07267726 | 0.00058522 | 0.01674394 | 20.7663831 | 75.912614 | 33.8329367 | 41.8080324 | 162.048202 | 80.6903173 | 175.039306 | 78.9303964 |
| Tagln2 | ENSMUSG00000026547 | 6577.75195 | 8879.35368 | 4276.15021 | 0.54428477 | -0.8775664 | 0.00058787 | 0.01679036 | 10441.715 | 6648.07317 | 10860.3727 | 7567.25387 | 6364.25024 | 4056.98659 | 4807.65853 | 1875.70549 |
| Ebf4 | ENSMUSG00000053552 | 1123.71447 | 881.509013 | 1365.91994 | 1.50352745 | 0.58835121 | 0.00058903 | 0.01679407 | 917.496564 | 936.948839 | 758.040663 | 913.549985 | 1159.41631 | 1344.15769 | 1870.15679 | 1089.94896 |
| Mdfi | ENSMUSG00000032717 | 318.747933 | 194.669786 | 442.826081 | 1.92420629 | 0.94426347 | 0.00059087 | 0.01681726 | 250.140524 | 135.186847 | 170.079087 | 223.272684 | 732.110629 | 249.220727 | 513.273153 | 276.699817 |
| Tnfrsf1a | ENSMUSG00000030341 | 3463.20454 | 4413.03302 | 2513.37606 | 0.60492113 | -0.725181 | 0.0005963 | 0.01694211 | 6274.27949 | 3425.42672 | 3793.86093 | 4158.56493 | 3352.85447 | 2723.04286 | 1721.43919 | 2256.16774 |
| Acacb | ENSMUSG00000042010 | 810.379454 | 323.301257 | 1297.45765 | 2.22831911 | 1.15595585 | 0.00060605 | 0.01718939 | 140.645049 | 284.932277 | 208.484042 | 659.14366 | 1236.58212 | 661.864881 | 2154.43116 | 1136.95245 |
| S1pr2 | ENSMUSG00000043895 | 2161.29945 | 2725.53142 | 1597.06747 | 0.61757524 | -0.6953132 | 0.00061156 | 0.01731545 | 3342.44376 | 3103.05809 | 2324.41419 | 2132.20965 | 1606.01343 | 2084.6701 | 1008.12112 | 1689.46523 |
| Stt3b | ENSMUSG00000032437 | 3276.84058 | 3764.05592 | 2789.62525 | 0.74856997 | -0.4177909 | 0.00061364 | 0.01734431 | 3926.73427 | 3493.02014 | 3656.70038 | 3979.76887 | 3357.67734 | 2578.00457 | 2537.41189 | 2685.4072 |
| Clec16a | ENSMUSG00000068663 | 1409.62906 | 1726.72385 | 1092.53428 | 0.65473039 | -0.6110271 | 0.00061536 | 0.01736284 | 1758.53508 | 1830.22193 | 1445.67224 | 1872.46613 | 944.316608 | 1422.80522 | 776.490154 | 1226.52515 |
| Strn3 | ENSMUSG00000020954 | 1350.36124 | 1527.72926 | 1172.99322 | 0.77346616 | -0.3705899 | 0.00061808 | 0.01740947 | 1641.48819 | 1588.9654 | 1444.75784 | 1435.70562 | 1078.3922 | 1212.39755 | 1108.14358 | 1293.03953 |
| Cacna1h | ENSMUSG00000024112 | 1788.43912 | 2122.07252 | 1454.80573 | 0.69891289 | -0.5168154 | 0.00062622 | 0.01760858 | 2252.20864 | 1494.33461 | 2341.78786 | 2399.95897 | 1435.28408 | 1511.6667 | 1421.37181 | 1450.90032 |
| Rcan1 | ENSMUSG00000022951 | 1136.32035 | 1389.72662 | 882.914089 | 0.65701085 | -0.6060109 | 0.00062746 | 0.01761317 | 1495.17959 | 1502.6538 | 1312.1693 | 1248.90378 | 846.894772 | 1182.77706 | 608.031273 | 893.953254 |
| Vps8 | ENSMUSG00000033653 | 1275.99523 | 1486.49283 | 1065.49764 | 0.72652487 | -0.4609159 | 0.00063261 | 0.01772722 | 1518.77775 | 1733.51134 | 1281.99398 | 1411.68824 | 958.785198 | 1239.97526 | 1072.60928 | 990.620818 |
| Adamts4 | ENSMUSG00000006403 | 541.372726 | 864.378545 | 218.366907 | 0.45068861 | -1.1497971 | 0.00064093 | 0.01792953 | 1826.49779 | 398.281249 | 648.31222 | 584.422921 | 363.643883 | 211.429059 | 126.344161 | 172.050527 |
| Lama5 | ENSMUSG00000015647 | 344.75735 | 216.210385 | 473.304316 | 1.88477774 | 0.9143944 | 0.00064397 | 0.01793464 | 250.140524 | 174.703002 | 282.550742 | 157.447271 | 581.637298 | 295.183566 | 734.375434 | 282.020967 |
| Cap2 | ENSMUSG00000021373 | 724.641522 | 907.243367 | 542.039676 | 0.62689089 | -0.6737137 | 0.0006444 | 0.01793464 | 999.61817 | 1096.05336 | 808.332866 | 724.969073 | 430.199395 | 735.405424 | 414.566777 | 587.98711 |
| Slc6a20a | ENSMUSG00000036814 | 140.827283 | 62.1889714 | 219.465594 | 2.18561965 | 1.12804236 | 0.00065448 | 0.01817993 | 33.9813542 | 90.4711976 | 89.6115621 | 34.6917716 | 58.8389306 | 152.188067 | 418.515032 | 248.320348 |
| Eps15l1 | ENSMUSG00000006276 | 1710.76209 | 1445.76351 | 1975.76067 | 1.3507108 | 0.43371881 | 0.00065663 | 0.01817993 | 1400.78694 | 1498.4942 | 1435.6138 | 1448.15908 | 1968.69275 | 1646.49103 | 2392.64254 | 1895.21637 |
| Pim3 | ENSMUSG00000035828 | 1463.09369 | 1170.96852 | 1755.21886 | 1.4628864 | 0.54881774 | 0.00065732 | 0.01817993 | 1317.7214 | 1106.45235 | 926.290943 | 1333.40937 | 1456.50468 | 1818.08563 | 2159.6955 | 1586.58965 |
| Filip1l | ENSMUSG00000043336 | 267.329725 | 344.119496 | 190.539954 | 0.59315874 | -0.7535099 | 0.00065927 | 0.01817993 | 410.60803 | 273.49339 | 273.406705 | 418.969857 | 243.072304 | 161.380635 | 218.470111 | 139.236767 |
| Fam168a | ENSMUSG00000029461 | 5025.21535 | 5822.12371 | 4228.30699 | 0.73523865 | -0.4437155 | 0.00065936 | 0.01817993 | 5786.26948 | 6726.06558 | 5115.17427 | 5660.9855 | 4213.25326 | 4319.48547 | 3604.75683 | 4775.73241 |
| Tacr1 | ENSMUSG00000030043 | 25.4384341 | 43.8420816 | 7.03478652 | 0.44680109 | -1.1622954 | 0.00065989 | 0.01817993 | 38.7009868 | 33.2767623 | 40.2337626 | 63.156815 | 17.3623074 | 1.02139642 | 0 | 9.75544225 |
| Rnf168 | ENSMUSG00000014074 | 919.936891 | 1050.53112 | 789.342659 | 0.75710557 | -0.4014336 | 0.0006663 | 0.0183256 | 1007.16958 | 1038.85892 | 1074.42434 | 1081.67165 | 726.323193 | 864.101373 | 875.196529 | 691.749542 |
| Cacng3 | ENSMUSG00000066189 | 14.1244591 | 24.5867886 | 3.66212962 | 0.44853631 | -1.1567033 | 0.00067291 | 0.01847628 | 25.4860157 | 18.7181788 | 28.3465145 | 25.7964455 | 6.75200843 | 0 | 7.89651004 | 0 |
| Txnl1 | ENSMUSG00000024583 | 1993.83464 | 2286.91257 | 1700.75672 | 0.75102033 | -0.4130761 | 0.00067845 | 0.01855456 | 2205.95624 | 2075.63805 | 2464.31796 | 2401.73803 | 1838.47544 | 1917.16108 | 1506.91733 | 1540.47302 |
| Pde4b | ENSMUSG00000028525 | 2623.45159 | 3375.32175 | 1871.58143 | 0.59467087 | -0.7498367 | 0.00067904 | 0.01855456 | 4081.53821 | 3719.71809 | 2678.28842 | 3021.74226 | 1642.66719 | 2510.5924 | 1044.9715 | 2288.09464 |
| C2cd4d | ENSMUSG00000091648 | 59.0251618 | 32.7895599 | 85.2607637 | 2.02733399 | 1.01958378 | 0.00067917 | 0.01855456 | 24.5420892 | 57.1944352 | 18.2880739 | 31.1336412 | 108.032135 | 73.5405424 | 97.3902905 | 62.0800871 |
| Bex1 | ENSMUSG00000050071 | 569.639741 | 464.59544 | 674.684041 | 1.42364069 | 0.50958507 | 0.00068368 | 0.01864641 | 417.215516 | 414.91963 | 545.899006 | 480.347607 | 629.86593 | 574.024789 | 776.490154 | 718.355293 |
| Ptprn2 | ENSMUSG00000056553 | 992.493291 | 1285.72192 | 699.264667 | 0.58705319 | -0.7684369 | 0.00068841 | 0.01874418 | 1292.23539 | 1588.9654 | 1260.9627 | 1000.72418 | 353.998157 | 767.068713 | 755.432794 | 920.559005 |
| Chac2 | ENSMUSG00000020309 | 145.486984 | 180.311576 | 110.662391 | 0.63978327 | -0.6443448 | 0.00069379 | 0.01885927 | 151.972167 | 163.264115 | 210.31285 | 195.697173 | 102.244699 | 118.481985 | 103.970716 | 117.952165 |
| Fam53a | ENSMUSG00000037339 | 1214.03788 | 985.53944 | 1442.53632 | 1.43440484 | 0.52045226 | 0.00070032 | 0.0190051 | 914.664784 | 983.744286 | 966.524705 | 1077.22398 | 1893.45608 | 1372.75679 | 1325.2976 | 1178.6348 |
| Csad | ENSMUSG00000023044 | 1084.15469 | 909.520189 | 1258.78918 | 1.36573696 | 0.44967965 | 0.00070264 | 0.01903641 | 779.683294 | 968.145804 | 948.236631 | 942.015029 | 1339.79139 | 1219.54733 | 1424.00398 | 1051.81405 |
| Cst3 | ENSMUSG00000027447 | 7819.74294 | 6079.07209 | 9560.41379 | 1.52099606 | 0.60501641 | 0.00071117 | 0.01923574 | 6687.7193 | 7041.15493 | 4324.21507 | 6263.19907 | 10430.8885 | 7999.57677 | 11536.8012 | 8274.38875 |
| Fer | ENSMUSG00000000127 | 379.873895 | 456.586821 | 303.16097 | 0.68013811 | -0.5561004 | 0.00071437 | 0.01926667 | 420.991222 | 445.076696 | 442.571388 | 517.707976 | 352.069011 | 235.942573 | 343.498187 | 281.134109 |
| Skil | ENSMUSG00000027660 | 1390.74993 | 1671.93101 | 1109.56884 | 0.68067148 | -0.5549694 | 0.00071467 | 0.01926667 | 1923.72222 | 1797.98506 | 1221.64334 | 1744.37344 | 1120.8334 | 1350.28607 | 976.535075 | 990.620818 |
| Atrnl1 | ENSMUSG00000054843 | 1053.56415 | 1339.95963 | 767.168678 | 0.60812626 | -0.7175572 | 0.00071659 | 0.01928655 | 1728.32943 | 1159.48719 | 1177.75196 | 1294.26994 | 1138.19571 | 780.346866 | 627.772548 | 522.35959 |
| Slc9a2 | ENSMUSG00000026062 | 43.0555088 | 20.1893049 | 65.9217127 | 2.1474343 | 1.10261399 | 0.00072083 | 0.01936801 | 11.3271181 | 33.2767623 | 21.031285 | 15.1220543 | 64.6263664 | 24.5135141 | 97.3902905 | 77.1566797 |
| Tbc1d4 | ENSMUSG00000033083 | 585.092388 | 455.959414 | 714.225362 | 1.5171986 | 0.60140994 | 0.00072246 | 0.01936801 | 343.589248 | 527.228703 | 470.003499 | 483.016205 | 709.925458 | 599.5597 | 630.404718 | 917.011572 |
| Adgrf5 | ENSMUSG00000056492 | 690.762236 | 872.950353 | 508.574118 | 0.61543924 | -0.7003117 | 0.00072446 | 0.01936801 | 1079.85192 | 761.205938 | 940.006998 | 710.736551 | 534.373239 | 676.164431 | 505.376643 | 318.382161 |
| Taldo1 | ENSMUSG00000025503 | 4150.93595 | 3567.03277 | 4734.83913 | 1.31583932 | 0.39598333 | 0.00072596 | 0.01936801 | 3378.31297 | 3216.40706 | 3804.83377 | 3868.5773 | 5238.59397 | 4430.81768 | 5007.70345 | 4262.24141 |
| Ppp2r5b | ENSMUSG00000024777 | 1796.51861 | 2177.58703 | 1415.45019 | 0.66927199 | -0.5793355 | 0.0007266 | 0.01936801 | 2462.70425 | 2130.75269 | 2294.23887 | 1822.65231 | 1851.01488 | 1524.94486 | 1195.00519 | 1090.83582 |
| Tagln3 | ENSMUSG00000022658 | 1492.69906 | 1880.67956 | 1104.71857 | 0.61945352 | -0.6909321 | 0.00072785 | 0.01936801 | 1871.80626 | 1950.85019 | 2242.11786 | 1457.94394 | 1095.75451 | 1625.04171 | 763.329304 | 934.74874 |
| Pcdh12 | ENSMUSG00000024440 | 193.12178 | 289.056078 | 97.1874819 | 0.47503786 | -1.0738856 | 0.00072792 | 0.01936801 | 319.991086 | 261.014604 | 348.387808 | 226.830814 | 131.181878 | 120.524778 | 121.079821 | 15.963451 |
| Nubp1 | ENSMUSG00000022503 | 351.192981 | 417.734391 | 284.65157 | 0.69563048 | -0.5236069 | 0.00073163 | 0.01943362 | 413.43981 | 370.203981 | 473.661114 | 413.632661 | 340.49414 | 277.819827 | 273.745681 | 246.546632 |
| Mfsd2a | ENSMUSG00000028655 | 1785.47175 | 1206.32594 | 2364.61757 | 1.77182938 | 0.82523968 | 0.00073276 | 0.01943362 | 861.8049 | 1974.76786 | 742.4958 | 1246.23518 | 2087.33518 | 3348.13747 | 1953.07015 | 2069.92747 |
| Tub | ENSMUSG00000031028 | 10496.2739 | 12597.8411 | 8394.70672 | 0.68296242 | -0.5501219 | 0.00073985 | 0.01958978 | 12000.1377 | 14296.529 | 11738.2002 | 12356.4974 | 9352.49625 | 10357.9811 | 6688.344 | 7180.0055 |
| Cdh15 | ENSMUSG00000031962 | 355.678268 | 47.2891093 | 664.067426 | 2.13317479 | 1.09300218 | 0.00074141 | 0.0195995 | 52.8598843 | 35.35656 | 15.5448628 | 85.3951301 | 461.065719 | 137.888517 | 1908.32326 | 148.992209 |
| Syt4 | ENSMUSG00000024261 | 972.999621 | 1313.82632 | 632.172922 | 0.54616159 | -0.8726002 | 0.00074387 | 0.01962631 | 2207.8441 | 810.081183 | 1021.38893 | 1215.99107 | 891.265113 | 682.29281 | 576.445233 | 378.688531 |
| Mocs1 | ENSMUSG00000064120 | 373.47289 | 307.50621 | 439.43957 | 1.40589435 | 0.49148819 | 0.00074483 | 0.01962631 | 327.542498 | 332.767623 | 244.145786 | 325.568933 | 465.888582 | 433.072083 | 422.463287 | 436.334326 |
| Cldn14 | ENSMUSG00000047109 | 27.7130331 | 4.33330787 | 51.0927583 | 2.1513811 | 1.10526311 | 0.00076088 | 0.01998463 | 1.88785301 | 0 | 11.887248 | 3.55813042 | 98.3864086 | 32.6846855 | 69.7525054 | 3.54743355 |
| Mapre2 | ENSMUSG00000024277 | 3331.42745 | 3788.64087 | 2874.21403 | 0.76479047 | -0.3868636 | 0.00077118 | 0.02022278 | 4411.91249 | 3651.08477 | 3572.57524 | 3518.99099 | 2687.29936 | 3174.50008 | 2797.99672 | 2837.05998 |
| 2810403A07Rik | ENSMUSG00000028060 | 2482.28641 | 2164.14007 | 2800.43275 | 1.28599234 | 0.36288205 | 0.00077452 | 0.02027788 | 2116.28323 | 2120.3537 | 2283.26603 | 2136.65732 | 2827.16239 | 2700.57214 | 2476.87198 | 3197.12448 |
| Cldnd1 | ENSMUSG00000022744 | 1998.13236 | 2555.75748 | 1440.50724 | 0.60209251 | -0.7319429 | 0.00077688 | 0.0202869 | 2794.02246 | 2702.69704 | 2101.29969 | 2625.01072 | 1403.45318 | 2174.55298 | 809.392279 | 1374.6305 |
| Rnf128 | ENSMUSG00000031438 | 422.199571 | 341.757284 | 502.641859 | 1.43841387 | 0.52447884 | 0.00077735 | 0.0202869 | 304.888261 | 391.001957 | 342.901386 | 328.237531 | 430.199395 | 467.799561 | 615.927783 | 496.640697 |
| Tctn1 | ENSMUSG00000038593 | 370.426067 | 289.051163 | 451.800971 | 1.51221033 | 0.59665882 | 0.00080339 | 0.02089976 | 312.439674 | 289.091873 | 279.807531 | 274.865575 | 585.495589 | 376.89528 | 485.635367 | 359.177647 |
| Pcdhga4 | ENSMUSG00000103677 | 85.9025751 | 126.688655 | 45.1164952 | 0.48475203 | -1.0446811 | 0.00081088 | 0.02106085 | 126.486152 | 178.862597 | 144.475784 | 56.9300867 | 26.0434611 | 28.5990998 | 51.3273153 | 74.4961045 |
| Sox11 | ENSMUSG00000063632 | 2945.92125 | 3604.19477 | 2287.64773 | 0.65710212 | -0.6058105 | 0.00081816 | 0.02121629 | 4326.01518 | 2508.23596 | 3930.10708 | 3652.42088 | 2253.24167 | 2165.36041 | 1834.6225 | 2897.36635 |
| Ugdh | ENSMUSG00000029201 | 7518.7583 | 5429.53878 | 9607.97782 | 1.65742878 | 0.72894688 | 0.00082194 | 0.02128051 | 5935.40987 | 4080.56298 | 5083.17014 | 6619.01211 | 14873.71 | 7256.00018 | 8508.48957 | 7793.7115 |
| Slc2a3 | ENSMUSG00000003153 | 1740.34016 | 2125.26858 | 1355.41173 | 0.65956682 | -0.6004093 | 0.00082604 | 0.02133734 | 2154.04029 | 1464.17754 | 2035.46262 | 2847.39387 | 1498.94587 | 1387.05634 | 1139.72962 | 1395.9151 |
| Ralb | ENSMUSG00000004451 | 1171.5383 | 1413.10168 | 929.974916 | 0.67666933 | -0.5634771 | 0.00082675 | 0.02133734 | 1302.61858 | 1509.93309 | 1521.56775 | 1318.28732 | 1018.5887 | 1191.96962 | 665.939013 | 843.402326 |
| Golm1 | ENSMUSG00000021556 | 631.558328 | 783.029657 | 480.086999 | 0.64018127 | -0.6434476 | 0.00083165 | 0.02136268 | 870.300239 | 740.407962 | 632.767357 | 888.643072 | 622.149349 | 336.039423 | 418.515032 | 543.644191 |
| Itga8 | ENSMUSG00000026768 | 888.386879 | 1408.21209 | 368.561672 | 0.45858902 | -1.1247263 | 0.00083355 | 0.02137799 | 1675.46955 | 734.168569 | 1193.29682 | 2029.9134 | 596.105888 | 508.655418 | 113.183311 | 256.302074 |
| Sh3glb1 | ENSMUSG00000037062 | 5808.89437 | 6907.67219 | 4710.11656 | 0.69656762 | -0.5216647 | 0.00083551 | 0.02139458 | 7247.46771 | 7173.22208 | 6393.51063 | 6816.48835 | 4939.57646 | 5458.34248 | 3319.16639 | 5123.3809 |
| Ngrn | ENSMUSG00000047084 | 639.972119 | 533.982442 | 745.961797 | 1.37650408 | 0.46100888 | 0.00083818 | 0.0214293 | 493.673563 | 535.547894 | 593.447998 | 513.260313 | 812.170157 | 798.732002 | 772.541899 | 600.403128 |
| Sorbs3 | ENSMUSG00000022091 | 1407.68712 | 1071.93119 | 1743.44305 | 1.55933346 | 0.64092948 | 0.00084423 | 0.02155017 | 1043.98272 | 977.504893 | 1003.10085 | 1263.1363 | 2477.9871 | 1430.97639 | 1824.09382 | 1240.71488 |
| Map7d1 | ENSMUSG00000028849 | 4004.46479 | 4645.15657 | 3363.77301 | 0.73370918 | -0.4467198 | 0.00084864 | 0.021629 | 4605.41742 | 4183.51296 | 5124.31831 | 4667.37758 | 3331.63388 | 3784.27374 | 2678.23299 | 3660.95142 |
| Ptpra | ENSMUSG00000027303 | 3381.77554 | 3780.0443 | 2983.50677 | 0.79365074 | -0.3334238 | 0.0008523 | 0.02168175 | 3982.42593 | 3873.62311 | 3670.41643 | 3593.71172 | 3117.49875 | 3149.98656 | 2633.4861 | 3033.05568 |
| Snx3 | ENSMUSG00000019804 | 5054.8844 | 5927.82779 | 4181.941 | 0.71711427 | -0.4797251 | 0.00085336 | 0.02168175 | 6149.68119 | 5589.45617 | 5885.10218 | 6087.07162 | 3756.04583 | 4977.26476 | 3299.42511 | 4695.0283 |
| Edil3 | ENSMUSG00000034488 | 2172.04789 | 1392.55062 | 2951.54517 | 1.84520462 | 0.88378081 | 0.00085807 | 0.02176752 | 1851.98381 | 1929.01232 | 896.115621 | 893.090735 | 1488.33557 | 2703.63633 | 4265.43151 | 3348.77727 |
| Abcb10 | ENSMUSG00000031974 | 368.871969 | 267.38388 | 470.360057 | 1.64890581 | 0.72150899 | 0.00086468 | 0.02186357 | 341.701395 | 295.331266 | 235.916153 | 196.586706 | 413.80166 | 366.681315 | 664.622928 | 436.334326 |
| Dcxr | ENSMUSG00000039450 | 90.9078415 | 61.3034169 | 120.512266 | 1.76975466 | 0.82354938 | 0.00086551 | 0.02186357 | 44.3645458 | 86.3116023 | 57.6074328 | 56.9300867 | 108.996708 | 101.118246 | 153.981946 | 117.952165 |
| F2rl3 | ENSMUSG00000050147 | 21.5251718 | 31.9664608 | 11.0838829 | 0.48147019 | -1.0544816 | 0.00086587 | 0.02186357 | 23.5981627 | 30.1570659 | 42.9769737 | 31.1336412 | 9.64572634 | 15.3209463 | 13.1608501 | 6.20800871 |
| Fam111a | ENSMUSG00000024691 | 753.483484 | 965.432796 | 541.534171 | 0.60076195 | -0.7351347 | 0.00087119 | 0.02196401 | 911.833005 | 613.540305 | 1259.13389 | 1077.22398 | 774.551825 | 513.7624 | 426.411542 | 451.410919 |
| Kcnj2 | ENSMUSG00000041695 | 320.06921 | 381.460767 | 258.677653 | 0.69374437 | -0.5275239 | 0.00087763 | 0.02209232 | 325.654645 | 399.321148 | 370.333496 | 430.533781 | 307.69867 | 233.899781 | 244.791811 | 248.320348 |
| Cds1 | ENSMUSG00000029330 | 97.9418018 | 55.7708328 | 140.112771 | 1.98504276 | 0.98917008 | 0.0008884 | 0.02232891 | 42.4766928 | 43.6757505 | 96.0123879 | 40.9184998 | 224.745424 | 99.0754529 | 138.188926 | 98.4412809 |
| Entpd1 | ENSMUSG00000048120 | 1116.72009 | 1442.62428 | 790.815904 | 0.59208154 | -0.7561322 | 0.00089859 | 0.02249626 | 2017.17094 | 1337.30989 | 1375.26316 | 1040.75315 | 937.5646 | 928.449347 | 431.675882 | 865.573786 |
| Agap1 | ENSMUSG00000055013 | 7604.61254 | 9327.94101 | 5881.28406 | 0.65396737 | -0.6127094 | 0.00089919 | 0.02249626 | 9497.7885 | 9792.72721 | 8150.99453 | 9870.25378 | 6253.32438 | 8036.34705 | 4175.93773 | 5059.5271 |
| Atp13a5 | ENSMUSG00000048939 | 106.268517 | 48.5348037 | 164.00223 | 2.13711624 | 1.09566538 | 0.00091226 | 0.0227884 | 64.1870024 | 60.3141317 | 42.06257 | 27.5755108 | 32.7954695 | 138.909913 | 280.326106 | 203.977429 |
| Gstm1 | ENSMUSG00000058135 | 468.907412 | 348.050958 | 589.763867 | 1.60517167 | 0.6827276 | 0.00091674 | 0.0228654 | 300.168629 | 437.797404 | 342.901386 | 311.336412 | 556.55841 | 530.104743 | 844.926574 | 427.465742 |
| Dmp1 | ENSMUSG00000029307 | 62.7236347 | 101.693857 | 23.7534125 | 0.45747108 | -1.1282476 | 0.00092046 | 0.02292312 | 50.9720313 | 118.548466 | 121.615691 | 115.639239 | 16.3977348 | 34.7274783 | 42.1147202 | 1.77371677 |
| Enpp3 | ENSMUSG00000019989 | 121.005451 | 158.662582 | 83.3483208 | 0.57514264 | -0.7980083 | 0.00092398 | 0.02297564 | 167.074992 | 96.7105905 | 160.93505 | 209.929695 | 78.1303833 | 77.626128 | 98.7063755 | 78.9303964 |
| Slc25a34 | ENSMUSG00000040740 | 13.4621016 | 5.09778254 | 21.8264206 | 2.18091359 | 1.12493261 | 0.00092993 | 0.02308844 | 4.71963253 | 2.07979765 | 9.14403695 | 4.44766302 | 17.3623074 | 31.6632891 | 27.6377851 | 10.6423006 |
| S1pr3 | ENSMUSG00000067586 | 570.936775 | 756.101476 | 385.772074 | 0.56615689 | -0.8207262 | 0.00093466 | 0.02317086 | 993.010684 | 528.268602 | 778.157544 | 724.969073 | 511.223496 | 359.53154 | 484.319282 | 188.013978 |
| Nqo2 | ENSMUSG00000046949 | 348.68717 | 409.63024 | 287.7441 | 0.71582122 | -0.4823288 | 0.00094711 | 0.02339725 | 369.075264 | 428.438315 | 439.828177 | 401.179205 | 278.761491 | 276.79843 | 256.636576 | 338.779904 |
| Dennd6b | ENSMUSG00000015377 | 1136.48866 | 890.100341 | 1382.87697 | 1.50458016 | 0.58936097 | 0.0009478 | 0.02339725 | 765.524396 | 1026.38014 | 1017.73131 | 750.765519 | 1009.90755 | 1709.81761 | 1273.97029 | 1537.81244 |
| Rarb | ENSMUSG00000017491 | 242.324064 | 338.971016 | 145.677111 | 0.52033551 | -0.9424859 | 0.00094809 | 0.02339725 | 353.97244 | 400.361047 | 302.667623 | 298.882955 | 225.709996 | 207.343474 | 61.8559953 | 87.7989803 |
| Cdh22 | ENSMUSG00000053166 | 749.204842 | 1041.60501 | 456.804674 | 0.52481987 | -0.9301058 | 0.00096381 | 0.02374933 | 1795.34821 | 836.078653 | 870.512317 | 664.480856 | 304.804952 | 700.677945 | 240.843556 | 580.892243 |
| St3gal6 | ENSMUSG00000022747 | 436.829532 | 548.66888 | 324.990184 | 0.62479409 | -0.6785473 | 0.00097929 | 0.02409449 | 594.673699 | 499.151435 | 443.485792 | 657.364595 | 329.883841 | 459.62839 | 240.843556 | 269.60495 |
| Plaur | ENSMUSG00000046223 | 193.763699 | 260.063709 | 127.463689 | 0.55485752 | -0.8498107 | 0.00098159 | 0.0240984 | 380.402382 | 183.022193 | 230.429731 | 246.400532 | 173.623074 | 152.188067 | 80.2811854 | 103.762431 |
| Scg3 | ENSMUSG00000032181 | 3120.10882 | 4103.27405 | 2136.9436 | 0.57379088 | -0.8014031 | 0.0009824 | 0.0240984 | 4509.13692 | 4411.25081 | 4061.78121 | 3430.92726 | 864.25708 | 2694.44376 | 2337.36697 | 2651.70658 |
| Abcb8 | ENSMUSG00000028973 | 1142.55996 | 948.290957 | 1336.82896 | 1.38721407 | 0.47219044 | 0.00098528 | 0.02410581 | 1045.87057 | 955.667018 | 886.971584 | 904.654659 | 1541.38707 | 1063.27367 | 1516.12993 | 1226.52515 |
| Rnf14 | ENSMUSG00000060450 | 2778.04826 | 3181.6866 | 2374.40992 | 0.7537578 | -0.4078271 | 0.00098566 | 0.02410581 | 3265.04178 | 3394.22976 | 2778.87283 | 3288.60204 | 2220.4462 | 2824.16111 | 2249.18928 | 2203.84309 |
| Grik3 | ENSMUSG00000001985 | 5337.30553 | 6301.73624 | 4372.87482 | 0.70727967 | -0.4996473 | 0.00099531 | 0.02428631 | 8003.55285 | 5434.51125 | 5409.61226 | 6359.26859 | 4702.29159 | 4043.70843 | 3927.19766 | 4818.30162 |
| Tmem178 | ENSMUSG00000024245 | 488.257824 | 586.693502 | 389.822146 | 0.68216578 | -0.5518057 | 0.00099601 | 0.02428631 | 523.879211 | 514.749917 | 771.756718 | 536.388161 | 433.093112 | 415.708344 | 359.291207 | 351.195921 |
| Inca1 | ENSMUSG00000057054 | 45.8113747 | 27.3644215 | 64.2583279 | 1.92187245 | 0.94251259 | 0.00100324 | 0.02442608 | 30.2056482 | 27.0373694 | 22.8600924 | 29.354576 | 61.7326485 | 50.0484247 | 100.022461 | 45.2297777 |
| Syn1 | ENSMUSG00000037217 | 1561.07955 | 2008.512 | 1113.64709 | 0.59715569 | -0.743821 | 0.00102052 | 0.0248098 | 2500.46131 | 1915.49363 | 2335.38704 | 1282.70602 | 727.287766 | 1495.32436 | 885.72521 | 1346.25103 |
| Tnfrsf8 | ENSMUSG00000028602 | 28.0780229 | 43.9289138 | 12.2271321 | 0.46717863 | -1.0979538 | 0.00102397 | 0.02483023 | 70.794488 | 36.3964588 | 33.8329367 | 34.6917716 | 26.0434611 | 6.12837853 | 10.5286801 | 6.20800871 |
| Klf10 | ENSMUSG00000037465 | 2712.44568 | 3511.01225 | 1913.8791 | 0.59061287 | -0.7597153 | 0.0010244 | 0.02483023 | 3892.75291 | 4303.10133 | 2860.25476 | 2987.94002 | 2138.45753 | 2963.07102 | 1206.84995 | 1347.13789 |
| Gadd45g | ENSMUSG00000021453 | 715.824744 | 992.746558 | 438.90293 | 0.52750909 | -0.9227322 | 0.00103605 | 0.02506452 | 1805.73141 | 395.161553 | 683.973964 | 1086.11931 | 546.912683 | 385.066451 | 352.710782 | 470.921803 |
| Pak3 | ENSMUSG00000031284 | 1642.67262 | 1974.20914 | 1311.13609 | 0.6820861 | -0.5519742 | 0.00103713 | 0.02506452 | 2674.14379 | 1496.41441 | 1963.22473 | 1763.05362 | 1387.05545 | 1344.15769 | 1152.89047 | 1360.44077 |
| Kcnq1 | ENSMUSG00000009545 | 93.6371218 | 141.834934 | 45.4393092 | 0.47730554 | -1.067015 | 0.00105148 | 0.02537367 | 196.336713 | 109.189376 | 108.81404 | 152.999608 | 71.3783749 | 51.0698211 | 51.3273153 | 7.98172548 |
| Dnmt3a | ENSMUSG00000020661 | 3443.98442 | 3871.32259 | 3016.64626 | 0.78432527 | -0.350476 | 0.00106406 | 0.02563947 | 3661.49092 | 3959.93472 | 3775.57286 | 4088.29185 | 2775.07547 | 3223.52711 | 2744.03724 | 3323.94523 |
| Abcb4 | ENSMUSG00000042476 | 489.553162 | 342.035847 | 637.070477 | 1.70979904 | 0.77382677 | 0.00106825 | 0.02570242 | 314.327527 | 550.106477 | 204.826428 | 298.882955 | 774.551825 | 515.805193 | 583.025658 | 674.899232 |
| Apoc1 | ENSMUSG00000040564 | 69.1025645 | 30.2526358 | 107.952493 | 2.13648717 | 1.09524065 | 0.00107154 | 0.02574365 | 30.2056482 | 34.3166611 | 16.4592665 | 40.0289672 | 64.6263664 | 97.0326601 | 31.5860402 | 238.564906 |
| Mfi2 | ENSMUSG00000022780 | 34.8101197 | 11.8728969 | 57.7473425 | 2.16988631 | 1.11761945 | 0.00108104 | 0.0259339 | 4.71963253 | 23.9176729 | 6.40082586 | 12.4534565 | 111.890425 | 34.7274783 | 71.0685904 | 13.3028758 |
| Rock1 | ENSMUSG00000024290 | 1682.3051 | 1937.60966 | 1427.00053 | 0.74505895 | -0.4245735 | 0.00108516 | 0.02599441 | 2077.58224 | 2002.84513 | 1660.55711 | 2009.45415 | 1259.73186 | 1697.56085 | 1345.03888 | 1405.67054 |
| Rapgef2 | ENSMUSG00000062232 | 1398.14111 | 1613.62711 | 1182.65511 | 0.74218832 | -0.4301428 | 0.00108789 | 0.02602176 | 1791.57251 | 1797.98506 | 1332.28618 | 1532.66468 | 1172.92032 | 1274.70273 | 1047.60367 | 1235.39373 |
| Plekhg2 | ENSMUSG00000037552 | 1499.45503 | 1819.99059 | 1178.91947 | 0.66855316 | -0.5808858 | 0.00109202 | 0.02608246 | 2022.8345 | 1549.44925 | 1998.88648 | 1708.79213 | 1563.57224 | 1198.098 | 1126.56877 | 827.438875 |
| Bfsp2 | ENSMUSG00000032556 | 394.687295 | 268.208101 | 521.166489 | 1.7522522 | 0.80921043 | 0.00109712 | 0.02609225 | 217.103096 | 253.735313 | 155.448628 | 446.545368 | 448.526275 | 560.746636 | 444.836732 | 630.556313 |
| Frmd8 | ENSMUSG00000024816 | 2342.76636 | 2970.50986 | 1715.02285 | 0.61411917 | -0.7034094 | 0.00109804 | 0.02609225 | 3381.14475 | 2941.87377 | 2432.31383 | 3126.70711 | 2276.39142 | 2085.69149 | 929.156015 | 1568.85249 |
| Btrc | ENSMUSG00000025217 | 1069.90124 | 1228.3159 | 911.486573 | 0.75062898 | -0.4138281 | 0.00109882 | 0.02609225 | 1208.22593 | 1307.15282 | 1264.62031 | 1133.26454 | 973.253787 | 1042.84575 | 758.064964 | 871.781794 |
| Nkx6-2 | ENSMUSG00000041309 | 272.312578 | 84.0232242 | 460.601932 | 2.16451788 | 1.11404572 | 0.00110128 | 0.02611271 | 58.5234434 | 124.787859 | 24.6888998 | 128.092695 | 211.241407 | 235.942573 | 1050.23584 | 344.987912 |
| Osbpl10 | ENSMUSG00000040875 | 497.605074 | 605.506718 | 389.70343 | 0.66624563 | -0.5858739 | 0.00110345 | 0.02612626 | 551.25308 | 680.09383 | 508.408454 | 682.271508 | 365.573028 | 437.157668 | 284.274361 | 471.808662 |
| Slc39a8 | ENSMUSG00000053897 | 281.799664 | 373.54992 | 190.049409 | 0.56731079 | -0.8177888 | 0.0011106 | 0.0262574 | 364.355631 | 360.844891 | 408.738452 | 360.260705 | 265.257474 | 260.456088 | 131.608501 | 102.875573 |
| Dalir | ENSMUSG00000099784 | 15.279173 | 5.35153947 | 25.2068066 | 2.16452666 | 1.11405157 | 0.00111262 | 0.02626715 | 4.71963253 | 3.11969647 | 8.22963325 | 5.33719563 | 5.7874358 | 37.7916676 | 36.8503802 | 20.3977429 |
| Chst11 | ENSMUSG00000034612 | 11372.2744 | 13476.3422 | 9268.20655 | 0.7022017 | -0.5100426 | 0.00112184 | 0.02644656 | 15874.0121 | 13384.5377 | 10583.3084 | 14063.5105 | 9793.30595 | 10469.3133 | 7866.24009 | 8943.96683 |
| Ascc3 | ENSMUSG00000038774 | 646.94856 | 774.68627 | 519.21085 | 0.68697198 | -0.5416768 | 0.00112698 | 0.02652957 | 808.001089 | 662.41555 | 639.168183 | 989.160257 | 463.959437 | 468.820958 | 573.813063 | 570.249943 |
| Pdlim7 | ENSMUSG00000021493 | 969.568337 | 1276.16054 | 662.976139 | 0.57427007 | -0.8001987 | 0.00113758 | 0.02674049 | 2003.95597 | 750.80695 | 1429.21297 | 920.666246 | 913.450284 | 642.458349 | 508.008813 | 587.98711 |
| Rap2c | ENSMUSG00000050029 | 1400.18857 | 1595.92027 | 1204.45687 | 0.76213434 | -0.3918828 | 0.00114191 | 0.02680362 | 1493.29173 | 1627.44166 | 1640.44023 | 1622.50747 | 1278.05874 | 1342.1149 | 984.431585 | 1213.22227 |
| Mtg1 | ENSMUSG00000039018 | 462.160035 | 382.596946 | 541.723125 | 1.39246095 | 0.47763687 | 0.00115505 | 0.02703442 | 433.262266 | 320.288837 | 435.256159 | 341.58052 | 544.018965 | 526.019157 | 510.640983 | 586.213394 |
| Mcam | ENSMUSG00000032135 | 5221.00138 | 6975.04894 | 3466.95382 | 0.56019381 | -0.8360021 | 0.00116559 | 0.02721401 | 8914.44192 | 4782.49468 | 5824.75154 | 8378.50761 | 5268.49572 | 3768.9528 | 3237.56912 | 1592.79766 |
| Fkbp1a | ENSMUSG00000032966 | 7191.81237 | 8171.70666 | 6211.91808 | 0.76659988 | -0.3834543 | 0.001176 | 0.0274069 | 8925.76904 | 7337.52609 | 9029.73649 | 7393.79501 | 5913.79482 | 6710.57449 | 6188.2317 | 6035.07132 |
| Nkain3 | ENSMUSG00000055761 | 89.1220421 | 117.24798 | 60.9961037 | 0.57382711 | -0.801312 | 0.00119222 | 0.02774533 | 140.645049 | 131.027252 | 92.3547732 | 104.964847 | 70.4138022 | 44.9414426 | 78.9651004 | 49.6640697 |
| Zfp286 | ENSMUSG00000047342 | 363.161451 | 304.724418 | 421.598485 | 1.36506988 | 0.4489748 | 0.00119492 | 0.02776869 | 271.850834 | 346.286308 | 306.325238 | 294.435292 | 410.907942 | 384.045055 | 438.256307 | 453.184636 |
| Gfra1 | ENSMUSG00000025089 | 3911.3347 | 5075.94656 | 2746.72284 | 0.58907514 | -0.7634764 | 0.00120448 | 0.02795119 | 6457.40123 | 5698.64555 | 4139.50553 | 4008.23392 | 3435.80772 | 3610.63635 | 1384.52143 | 2555.92587 |
| Dennd2a | ENSMUSG00000038456 | 3016.84819 | 3806.1961 | 2227.50028 | 0.62048357 | -0.6885351 | 0.001217 | 0.0281882 | 4157.05233 | 3551.25448 | 3496.67973 | 4019.79784 | 2136.52838 | 3108.10931 | 1193.6891 | 2471.67432 |
| Stard4 | ENSMUSG00000024378 | 407.347331 | 521.951071 | 292.743592 | 0.60238923 | -0.7312321 | 0.00122407 | 0.02828511 | 494.617489 | 420.119124 | 678.487541 | 494.580128 | 398.368498 | 276.79843 | 323.756912 | 172.050527 |
| Ivd | ENSMUSG00000027332 | 1176.3218 | 952.545416 | 1400.09818 | 1.43635645 | 0.52241382 | 0.00122879 | 0.02835409 | 925.047976 | 1054.45741 | 1079.91076 | 750.765519 | 1098.64823 | 1619.93472 | 1564.82507 | 1316.9847 |
| Pcdhga9 | ENSMUSG00000102440 | 40.6759365 | 66.3539142 | 14.9979588 | 0.46550878 | -1.1031197 | 0.00123754 | 0.0285158 | 39.6449133 | 58.2343341 | 43.8913773 | 123.645032 | 3.85829053 | 30.6418927 | 21.0573601 | 4.43429193 |
| Ehd2 | ENSMUSG00000074364 | 942.25702 | 1201.87232 | 682.641716 | 0.60773607 | -0.7184832 | 0.00126039 | 0.0289606 | 1728.32943 | 854.796832 | 1327.71416 | 896.648866 | 704.138022 | 693.52817 | 867.300019 | 465.600653 |
| Smad2 | ENSMUSG00000024563 | 541.950803 | 630.017903 | 453.883703 | 0.73033398 | -0.4533717 | 0.00126792 | 0.02909265 | 609.776523 | 563.625162 | 651.055431 | 695.614497 | 524.727513 | 396.301812 | 483.003197 | 411.502291 |
| Slc29a4 | ENSMUSG00000050822 | 1389.32545 | 1609.62766 | 1169.02323 | 0.73636203 | -0.4415129 | 0.00128111 | 0.02935409 | 1662.25458 | 1532.81086 | 1741.02463 | 1502.42057 | 958.785198 | 1226.6971 | 1080.50579 | 1410.10484 |
| Gimap4 | ENSMUSG00000054435 | 104.86042 | 156.764185 | 52.9566553 | 0.48660927 | -1.0391643 | 0.00128602 | 0.02942541 | 141.588976 | 159.10452 | 160.020647 | 166.342597 | 84.8823918 | 40.8558569 | 76.3329304 | 9.75544225 |
| Prkag2 | ENSMUSG00000028944 | 1028.01819 | 825.832774 | 1230.2036 | 1.45194717 | 0.53798896 | 0.0012887 | 0.02944539 | 853.309562 | 847.51754 | 899.773236 | 702.730758 | 1544.28079 | 957.048447 | 1384.52143 | 1034.96374 |
| Cyp4f17 | ENSMUSG00000091586 | 177.959606 | 127.904822 | 228.01439 | 1.65961706 | 0.73085039 | 0.00129334 | 0.02951016 | 146.308608 | 168.463609 | 74.981103 | 121.865967 | 249.824312 | 217.557438 | 213.205771 | 231.470039 |
| Crebrf | ENSMUSG00000048249 | 1323.18829 | 1051.97648 | 1594.4001 | 1.47208272 | 0.55785875 | 0.00129946 | 0.02960847 | 1142.15107 | 1168.84628 | 865.940299 | 1030.96829 | 1250.08613 | 1396.24891 | 2154.43116 | 1576.83421 |
| 1110004E09Rik | ENSMUSG00000022972 | 104.440233 | 133.865605 | 75.0148613 | 0.60315123 | -0.7294083 | 0.00130566 | 0.02965183 | 122.710446 | 124.787859 | 154.534224 | 133.429891 | 52.0869222 | 93.9684708 | 69.7525054 | 84.2515467 |
| Wdr7 | ENSMUSG00000040560 | 1636.01037 | 1925.59858 | 1346.42217 | 0.71272287 | -0.4885869 | 0.00130748 | 0.02965183 | 2065.3112 | 2231.62287 | 1581.91839 | 1823.54184 | 1368.72857 | 1620.95612 | 1094.98273 | 1301.02125 |
| Cyyr1 | ENSMUSG00000041134 | 101.647595 | 134.494953 | 68.800237 | 0.56890574 | -0.8137385 | 0.00130761 | 0.02965183 | 145.364682 | 99.830287 | 168.25028 | 124.534565 | 49.1932043 | 89.8828851 | 82.9133554 | 53.2115032 |
| St3gal4 | ENSMUSG00000032038 | 1274.82757 | 1581.45833 | 968.196821 | 0.64132258 | -0.6408779 | 0.00130862 | 0.02965183 | 2272.0311 | 1514.09269 | 1507.85169 | 1031.85782 | 1055.24246 | 973.39079 | 787.018834 | 1057.1352 |
| Gprasp2 | ENSMUSG00000072966 | 1945.77438 | 1617.80485 | 2273.74391 | 1.38308822 | 0.46789318 | 0.00131397 | 0.02973183 | 1641.48819 | 1813.58355 | 1756.5695 | 1259.57817 | 1902.13723 | 2331.84803 | 2289.98791 | 2571.00246 |
| Ppif | ENSMUSG00000021868 | 334.110181 | 263.724962 | 404.4954 | 1.48559457 | 0.57104045 | 0.00132613 | 0.02996539 | 208.607758 | 259.974706 | 233.172942 | 353.144444 | 409.943369 | 386.087847 | 426.411542 | 395.538841 |
| Cers5 | ENSMUSG00000023021 | 1712.6261 | 1511.38576 | 1913.86643 | 1.26001437 | 0.33344019 | 0.00134339 | 0.03031359 | 1552.7591 | 1531.77097 | 1430.12738 | 1530.88561 | 1963.86988 | 2001.93699 | 1654.31885 | 2035.34 |
| Cndp2 | ENSMUSG00000024644 | 1607.19264 | 1799.12089 | 1415.2644 | 0.79151295 | -0.3373151 | 0.00134703 | 0.03034503 | 1830.2735 | 1702.31437 | 1730.05179 | 1933.84388 | 1443.96523 | 1558.65094 | 1266.07378 | 1392.36767 |
| Bhlhe40 | ENSMUSG00000030103 | 1895.71382 | 2397.16843 | 1394.25921 | 0.61832951 | -0.6935522 | 0.00134872 | 0.03034503 | 2409.84437 | 2150.51076 | 1889.15803 | 3139.16056 | 2091.19347 | 1364.58562 | 1201.58561 | 919.672147 |
| Fam212b | ENSMUSG00000048458 | 5817.59524 | 9026.59795 | 2608.59252 | 0.475633 | -1.0720793 | 0.00135217 | 0.03034503 | 7722.26275 | 13987.6791 | 7858.38535 | 6538.06465 | 1831.72343 | 5538.0114 | 1059.44843 | 2005.18681 |
| Ube2l3 | ENSMUSG00000038965 | 3268.34784 | 3882.96359 | 2653.73209 | 0.69903349 | -0.5165665 | 0.00135222 | 0.03034503 | 4282.59456 | 4107.60035 | 3615.55221 | 3526.10725 | 2634.24786 | 3541.18139 | 2229.448 | 2210.0511 |
| Prkar2a | ENSMUSG00000032601 | 2003.77409 | 2291.95828 | 1715.58991 | 0.75643725 | -0.4027077 | 0.00135647 | 0.03037024 | 2382.4705 | 2040.28149 | 2333.55823 | 2411.52289 | 1646.52549 | 1599.5068 | 1546.39988 | 2069.92747 |
| Arpp19 | ENSMUSG00000007656 | 2713.43418 | 3031.01745 | 2395.85091 | 0.79515051 | -0.3307001 | 0.00135706 | 0.03037024 | 2999.79844 | 3028.18537 | 3045.87871 | 3050.2073 | 2467.3768 | 2597.4111 | 2047.82827 | 2470.78747 |
| Tmem44 | ENSMUSG00000022537 | 638.615534 | 811.405954 | 465.825114 | 0.61314614 | -0.7056971 | 0.0013646 | 0.03049712 | 786.29078 | 1031.57963 | 721.464515 | 706.288888 | 272.974055 | 637.351367 | 406.670267 | 546.304766 |
| St8sia2 | ENSMUSG00000025789 | 31.9574435 | 8.58565204 | 55.329235 | 2.11801175 | 1.08271059 | 0.0013798 | 0.03079487 | 6.60748554 | 4.15959529 | 16.4592665 | 7.11626084 | 154.331621 | 29.6204962 | 30.2699552 | 7.09486709 |
| Naa60 | ENSMUSG00000005982 | 1307.51203 | 1568.16389 | 1046.86016 | 0.68591164 | -0.5439053 | 0.00138704 | 0.03088355 | 1555.59088 | 1805.26436 | 1615.75133 | 1296.04901 | 963.608061 | 1421.78382 | 867.300019 | 934.74874 |
| Tigd2 | ENSMUSG00000049232 | 572.771172 | 706.50024 | 439.042105 | 0.64919922 | -0.6232668 | 0.00138756 | 0.03088355 | 866.524533 | 709.210997 | 573.331117 | 676.934312 | 541.125247 | 505.591229 | 290.854786 | 418.597159 |
| Fbln7 | ENSMUSG00000027386 | 107.605409 | 76.8958344 | 138.314984 | 1.66224815 | 0.73313577 | 0.00139731 | 0.03105829 | 76.458047 | 63.4338282 | 82.2963325 | 85.3951301 | 133.111023 | 97.0326601 | 177.671476 | 145.444775 |
| Grk4 | ENSMUSG00000052783 | 127.115358 | 88.2000932 | 166.030622 | 1.71470511 | 0.77796048 | 0.00140274 | 0.03113653 | 51.9159578 | 101.910085 | 87.7827547 | 111.191576 | 163.977348 | 202.236491 | 130.292416 | 167.616235 |
| Socs5 | ENSMUSG00000037104 | 1597.77711 | 1931.39163 | 1264.16259 | 0.67531508 | -0.5663673 | 0.00141723 | 0.03137595 | 2370.19946 | 2079.79765 | 1613.92252 | 1661.64691 | 1593.47399 | 1356.41445 | 942.316865 | 1164.44506 |
| Tm4sf1 | ENSMUSG00000027800 | 421.123371 | 553.14579 | 289.100951 | 0.5777426 | -0.7915012 | 0.00141762 | 0.03137595 | 679.627084 | 410.760035 | 705.005249 | 417.190792 | 383.899908 | 258.413295 | 355.342952 | 158.747651 |
| Xk | ENSMUSG00000015342 | 220.360379 | 278.114169 | 162.60659 | 0.62237417 | -0.6841459 | 0.00141928 | 0.03137595 | 329.430351 | 280.772682 | 216.713676 | 285.539966 | 196.772817 | 144.016895 | 111.867226 | 197.76942 |
| Zswim6 | ENSMUSG00000032846 | 888.123166 | 1067.42104 | 708.825289 | 0.68319645 | -0.5496276 | 0.00142543 | 0.03143151 | 1316.77748 | 877.674606 | 848.566629 | 1226.66546 | 803.489004 | 710.891909 | 580.393488 | 740.526753 |
| Fgfr2 | ENSMUSG00000030849 | 403.991225 | 143.870595 | 664.111854 | 2.12951002 | 1.09052152 | 0.00142564 | 0.03143151 | 223.710582 | 93.590894 | 106.070829 | 152.110075 | 790.949559 | 173.637392 | 1380.57317 | 311.287294 |
| Pde10a | ENSMUSG00000023868 | 1005.58919 | 1170.74461 | 840.43376 | 0.7295885 | -0.4548451 | 0.00144701 | 0.03181675 | 1296.01109 | 1324.8311 | 988.470394 | 1073.66585 | 865.221652 | 878.400923 | 694.892884 | 923.219581 |
| Nrp2 | ENSMUSG00000025969 | 637.673267 | 752.452663 | 522.893871 | 0.70828548 | -0.4975971 | 0.00145221 | 0.03184729 | 873.132018 | 668.654943 | 828.449747 | 639.573943 | 562.345845 | 462.692579 | 608.031273 | 458.505786 |
| Gpr179 | ENSMUSG00000070337 | 243.517231 | 131.154975 | 355.879486 | 2.00022591 | 1.00016295 | 0.0014523 | 0.03184729 | 137.81327 | 142.466139 | 87.7827547 | 156.557738 | 173.623074 | 344.210594 | 762.013219 | 143.671059 |
| Entpd2 | ENSMUSG00000015085 | 74.702339 | 44.0597537 | 105.344924 | 1.92216437 | 0.94273171 | 0.00145779 | 0.03192486 | 39.6449133 | 70.7131199 | 34.7473404 | 31.1336412 | 153.367049 | 75.5833352 | 125.028076 | 67.4012374 |
| Fbxo17 | ENSMUSG00000030598 | 218.009603 | 149.714517 | 286.304689 | 1.72601004 | 0.78744086 | 0.00146517 | 0.03200414 | 171.794624 | 152.865127 | 138.989362 | 135.208956 | 280.690636 | 184.872752 | 463.261922 | 216.393446 |
| Tnfaip1 | ENSMUSG00000017615 | 2447.52701 | 2697.57538 | 2197.47863 | 0.81756682 | -0.2905914 | 0.00146533 | 0.03200414 | 2820.4524 | 2715.17583 | 2689.26127 | 2565.41203 | 2353.55723 | 2175.57438 | 2230.76409 | 2030.01885 |
| Btnl9 | ENSMUSG00000040283 | 42.978416 | 72.5704951 | 13.3863368 | 0.47194665 | -1.0833043 | 0.001486 | 0.03241229 | 55.6916639 | 57.1944352 | 46.6345884 | 130.761293 | 8.6811537 | 13.2781535 | 31.5860402 | 0 |
| Pde7b | ENSMUSG00000019990 | 1133.02933 | 1392.48858 | 873.570081 | 0.65385274 | -0.6129623 | 0.0015091 | 0.03286869 | 1425.32902 | 1886.37646 | 1101.85645 | 1156.39239 | 938.529172 | 1088.80859 | 650.145993 | 816.796574 |
| Il10rb | ENSMUSG00000022969 | 388.087513 | 465.916939 | 310.258087 | 0.68572556 | -0.5442968 | 0.00151095 | 0.03286869 | 471.019327 | 476.273661 | 463.602673 | 452.772096 | 281.655209 | 394.259019 | 228.998791 | 336.119329 |
| Crim1 | ENSMUSG00000024074 | 547.500708 | 399.106955 | 695.894461 | 1.63094971 | 0.7057123 | 0.00151722 | 0.03296112 | 445.533311 | 523.069108 | 352.959826 | 274.865575 | 938.529172 | 545.425689 | 787.018834 | 512.604148 |
| Nsmaf | ENSMUSG00000028245 | 1632.73708 | 1410.27948 | 1855.19468 | 1.30352138 | 0.38241425 | 0.00151963 | 0.03296977 | 1456.4786 | 1482.89572 | 1316.74132 | 1385.00227 | 1543.31621 | 1851.79171 | 2116.26469 | 1909.40611 |
| Ist1 | ENSMUSG00000031729 | 3450.26338 | 2993.58278 | 3906.94397 | 1.29440447 | 0.3722885 | 0.00153144 | 0.03318197 | 3058.32188 | 3103.05809 | 3232.41706 | 2580.53409 | 3789.80588 | 3534.03162 | 4411.51694 | 3892.42146 |
| Plpp2 | ENSMUSG00000052151 | 253.183225 | 179.530129 | 326.83632 | 1.675588 | 0.74466746 | 0.00154107 | 0.03334623 | 144.420755 | 159.10452 | 133.502939 | 281.092303 | 295.159226 | 258.413295 | 375.084227 | 378.688531 |
| Plxdc2 | ENSMUSG00000026748 | 820.851896 | 975.494682 | 666.20911 | 0.69932192 | -0.5159714 | 0.0015521 | 0.03354048 | 870.300239 | 1175.08567 | 920.804521 | 935.7883 | 517.010932 | 772.175695 | 604.083018 | 771.566797 |
| Zwint | ENSMUSG00000019923 | 4562.86956 | 5246.72767 | 3879.01145 | 0.74810395 | -0.4186893 | 0.00155415 | 0.03354048 | 4949.9506 | 4347.81698 | 5683.01896 | 6006.12415 | 4035.7719 | 4206.11046 | 3437.61404 | 3836.54938 |
| Car8 | ENSMUSG00000041261 | 2255.39917 | 1589.71479 | 2921.08355 | 1.68559789 | 0.75326041 | 0.00156778 | 0.03378314 | 1023.21633 | 1678.3967 | 1714.50693 | 1942.73921 | 2496.31398 | 1993.76582 | 4843.19282 | 2351.06158 |
| Ttc13 | ENSMUSG00000037300 | 1263.40846 | 1089.93766 | 1436.87925 | 1.30644222 | 0.38564332 | 0.00156953 | 0.03378314 | 1051.53413 | 1225.00081 | 1142.09021 | 941.125496 | 1558.74938 | 1310.45161 | 1518.7621 | 1359.55391 |
| 1110008P14Rik | ENSMUSG00000039195 | 458.078532 | 562.768671 | 353.388393 | 0.65293425 | -0.6149904 | 0.00157645 | 0.03388738 | 586.17836 | 490.832244 | 584.303961 | 589.760117 | 248.859739 | 349.317576 | 501.428388 | 313.947869 |
| Irx6 | ENSMUSG00000031738 | 302.115245 | 170.379528 | 433.850961 | 1.9584331 | 0.96969984 | 0.00157903 | 0.0338983 | 184.065669 | 300.53076 | 77.7243141 | 119.197369 | 204.489398 | 529.083346 | 686.996374 | 314.834727 |
| Tomm7 | ENSMUSG00000028998 | 978.736265 | 816.421687 | 1141.05084 | 1.37593254 | 0.46040974 | 0.00159015 | 0.03409232 | 849.533856 | 723.76958 | 966.524705 | 725.858606 | 1358.11827 | 1101.06534 | 980.48333 | 1124.53643 |
| Ss18 | ENSMUSG00000037013 | 2230.6623 | 2547.32613 | 1913.99846 | 0.75909394 | -0.3976497 | 0.00160546 | 0.03435741 | 2688.30269 | 2406.32588 | 2660.91475 | 2433.76121 | 1674.49809 | 2300.18474 | 1729.3357 | 1951.97531 |
| Lrrc1 | ENSMUSG00000032352 | 533.435862 | 427.876908 | 638.994816 | 1.4541771 | 0.54020298 | 0.00160672 | 0.03435741 | 460.636135 | 524.109007 | 360.275056 | 366.487433 | 721.50033 | 501.505643 | 696.208969 | 636.764322 |
| Csnk1g3 | ENSMUSG00000073563 | 1245.13927 | 1420.20742 | 1070.07112 | 0.76060404 | -0.3947825 | 0.00161013 | 0.03438516 | 1402.67479 | 1299.87353 | 1410.9249 | 1567.35645 | 1121.79797 | 1204.22638 | 1062.0806 | 892.179537 |
| Tmem106c | ENSMUSG00000052369 | 1189.01478 | 1034.28298 | 1343.74658 | 1.2890812 | 0.36634314 | 0.00161388 | 0.03442039 | 1026.99204 | 1027.42004 | 993.042412 | 1089.67744 | 1575.14711 | 1270.61715 | 1323.98152 | 1205.24055 |
| Rock2 | ENSMUSG00000020580 | 2222.73792 | 2507.77628 | 1937.69957 | 0.77880535 | -0.3606653 | 0.00161656 | 0.03442846 | 2666.59238 | 2439.60264 | 2306.12612 | 2618.78399 | 2083.47689 | 2055.0496 | 1634.57758 | 1977.6942 |
| Cav1 | ENSMUSG00000007655 | 1650.94305 | 2115.4964 | 1186.38971 | 0.6047748 | -0.7255301 | 0.0016198 | 0.03442846 | 2934.66751 | 2264.89964 | 1163.1215 | 2099.29695 | 829.532465 | 1507.58112 | 1051.55192 | 1356.89333 |
| Car10 | ENSMUSG00000056158 | 257.064507 | 362.778087 | 151.350927 | 0.5219994 | -0.9378799 | 0.00162058 | 0.03442846 | 569.187683 | 412.839833 | 305.410834 | 163.673999 | 109.96128 | 185.894149 | 78.9651004 | 230.583181 |
| Psmd2 | ENSMUSG00000006998 | 4093.39169 | 4848.05183 | 3338.73155 | 0.70391564 | -0.5065256 | 0.0016238 | 0.03445187 | 5571.99817 | 4287.50285 | 4497.95177 | 5034.75454 | 4109.07942 | 3680.09131 | 3015.15075 | 2550.60472 |
| Zfp770 | ENSMUSG00000040321 | 1063.45146 | 1254.70497 | 872.197955 | 0.70984276 | -0.4944286 | 0.00162683 | 0.03447151 | 1418.72154 | 1389.30483 | 1025.04654 | 1185.74696 | 771.658107 | 1047.95273 | 729.111094 | 940.06989 |
| Pfkp | ENSMUSG00000021196 | 2249.97646 | 2603.58139 | 1896.37153 | 0.73818959 | -0.4379367 | 0.00163002 | 0.03449438 | 2727.9476 | 2144.27137 | 2497.23649 | 3044.87011 | 1605.04886 | 1867.11266 | 2088.62691 | 2024.6977 |
| Naalad2 | ENSMUSG00000043943 | 90.2937532 | 117.505086 | 63.0824203 | 0.58605306 | -0.7708968 | 0.00164322 | 0.03472861 | 117.990813 | 120.628263 | 135.331747 | 96.0695213 | 54.9806401 | 58.219596 | 92.1259505 | 47.0034945 |
| Taf13 | ENSMUSG00000048100 | 559.342632 | 470.939249 | 647.746016 | 1.355694 | 0.43903157 | 0.00165209 | 0.03482906 | 488.95393 | 454.435785 | 462.68827 | 477.679009 | 725.35862 | 694.549567 | 659.358588 | 511.717289 |
| Stmn4 | ENSMUSG00000022044 | 956.458577 | 1185.4692 | 727.447956 | 0.64349069 | -0.6360088 | 0.00165436 | 0.03482906 | 1491.40388 | 1048.21801 | 1365.20472 | 837.050181 | 622.149349 | 997.904304 | 593.554338 | 696.183834 |
| Coq8a | ENSMUSG00000026489 | 347.404123 | 262.602339 | 432.205907 | 1.56546297 | 0.64658939 | 0.00166124 | 0.03492881 | 295.448996 | 281.812581 | 225.857713 | 247.290064 | 266.222047 | 454.521408 | 477.738857 | 530.341315 |
| Mrpl24 | ENSMUSG00000019710 | 762.956213 | 664.717392 | 861.195034 | 1.28487193 | 0.36162457 | 0.0016797 | 0.03527148 | 722.103777 | 578.183745 | 663.857082 | 694.724964 | 891.265113 | 809.967362 | 905.466485 | 838.081176 |
| Flt4 | ENSMUSG00000020357 | 281.846981 | 373.248231 | 190.445731 | 0.57179141 | -0.8064391 | 0.00168604 | 0.0353592 | 497.449269 | 300.53076 | 370.333496 | 324.679401 | 277.796918 | 202.236491 | 189.516241 | 92.2332722 |
| Cdh9 | ENSMUSG00000025370 | 25.7151901 | 41.4735442 | 9.95683591 | 0.47619268 | -1.0703826 | 0.00169586 | 0.03551949 | 53.8038109 | 63.4338282 | 22.8600924 | 25.7964455 | 7.71658107 | 2.04279284 | 7.89651004 | 22.1714597 |
| Vopp1 | ENSMUSG00000037788 | 1271.80957 | 1470.02687 | 1073.59227 | 0.74082661 | -0.4327922 | 0.00171842 | 0.03594609 | 1583.90868 | 1364.34726 | 1404.52408 | 1527.32748 | 1089.96708 | 1126.60025 | 831.765724 | 1246.03603 |
| Atp6v1a | ENSMUSG00000052459 | 3344.02731 | 3894.06453 | 2793.9901 | 0.7289618 | -0.4560849 | 0.00172651 | 0.03606905 | 3818.18272 | 4372.77455 | 3383.29367 | 4002.00719 | 2716.23654 | 3523.81765 | 2538.72798 | 2397.17822 |
| Tsc22d3 | ENSMUSG00000031431 | 667.220694 | 491.47168 | 842.969709 | 1.60981408 | 0.68689408 | 0.00173096 | 0.03611575 | 533.318476 | 474.193863 | 568.759098 | 389.615281 | 605.751614 | 590.367132 | 1246.3325 | 929.427589 |
| Sema6a | ENSMUSG00000019647 | 3295.16353 | 4129.18842 | 2461.13863 | 0.6305112 | -0.6654061 | 0.00173325 | 0.03611738 | 3887.08935 | 4108.64025 | 4291.29654 | 4229.72754 | 2820.41038 | 3286.85369 | 1267.38986 | 2469.90061 |
| Zcchc7 | ENSMUSG00000035649 | 1257.88664 | 999.122505 | 1516.65078 | 1.47182721 | 0.55760831 | 0.00173802 | 0.03617072 | 964.692889 | 942.148333 | 1051.56425 | 1038.08455 | 949.139471 | 1658.74779 | 1810.93297 | 1647.78288 |
| Pea15a | ENSMUSG00000013698 | 12443.6957 | 16273.736 | 8613.65541 | 0.58436728 | -0.7750527 | 0.00175904 | 0.03651524 | 18374.4734 | 19500.1827 | 16177.6302 | 11042.6578 | 8526.82208 | 13140.265 | 4568.13106 | 8219.40353 |
| Stx12 | ENSMUSG00000028879 | 4047.94366 | 4497.76146 | 3598.12587 | 0.8040173 | -0.3147015 | 0.00176596 | 0.03660291 | 4341.118 | 4673.30531 | 4624.13948 | 4352.48304 | 3593.99763 | 4047.79402 | 3374.44196 | 3376.26988 |
| Tst | ENSMUSG00000044986 | 192.44725 | 121.433769 | 263.46073 | 1.83764977 | 0.87786184 | 0.00176784 | 0.03660291 | 84.009459 | 150.785329 | 101.49881 | 149.441478 | 474.569736 | 203.257888 | 206.625346 | 169.389952 |
| Spata2l | ENSMUSG00000033594 | 86.2144152 | 58.9981041 | 113.430726 | 1.72934712 | 0.79022748 | 0.00176999 | 0.03660291 | 68.9066349 | 69.6732211 | 49.3777995 | 48.0347607 | 121.536152 | 71.4977495 | 127.660246 | 133.028758 |
| Pcsk6 | ENSMUSG00000030513 | 1998.07447 | 1722.46379 | 2273.68515 | 1.30738117 | 0.38667983 | 0.00177376 | 0.03663462 | 1705.6752 | 1732.47144 | 1679.75959 | 1771.94895 | 2661.2559 | 2082.6273 | 2430.80901 | 1920.04841 |
| Gldc | ENSMUSG00000024827 | 1044.06717 | 1300.11368 | 788.020664 | 0.63847608 | -0.6472955 | 0.00180397 | 0.03721147 | 1469.69357 | 1121.01093 | 1172.26554 | 1437.48469 | 1104.43567 | 828.352498 | 464.578007 | 754.716487 |
| Zbtb33 | ENSMUSG00000048047 | 1006.45667 | 1187.13971 | 825.773637 | 0.71057875 | -0.4929336 | 0.0018076 | 0.03723666 | 1467.80572 | 1217.72152 | 1021.38893 | 1041.64268 | 974.21836 | 753.790559 | 696.208969 | 878.876661 |
| Ifnar1 | ENSMUSG00000022967 | 1711.93106 | 1993.13866 | 1430.72347 | 0.72956309 | -0.4548954 | 0.00180975 | 0.03723666 | 2070.97475 | 2133.87238 | 1916.59014 | 1851.11735 | 1650.38378 | 1684.2827 | 1158.15481 | 1230.07258 |
| Dcun1d4 | ENSMUSG00000051674 | 1515.48492 | 1065.42004 | 1965.5498 | 1.68677473 | 0.75426731 | 0.00181278 | 0.03723697 | 1006.22566 | 1096.05336 | 901.602043 | 1257.7991 | 3525.51298 | 1492.26017 | 1289.76331 | 1554.66275 |
| Kif21b | ENSMUSG00000041642 | 5431.93933 | 6677.45065 | 4186.42802 | 0.65414497 | -0.6123177 | 0.00181621 | 0.03723697 | 6647.13046 | 7571.50333 | 6455.69008 | 6035.47872 | 3689.49032 | 5876.09361 | 2700.60643 | 4479.52171 |
| Nfe2 | ENSMUSG00000058794 | 20.4117738 | 3.55080704 | 37.2727406 | 2.01891871 | 1.01358282 | 0.0018166 | 0.03723697 | 7.55141205 | 2.07979765 | 4.57201847 | 0 | 109.96128 | 21.4493249 | 5.26434003 | 12.4160174 |
| Hif3a | ENSMUSG00000004328 | 90.2261286 | 35.4225412 | 145.029716 | 2.08871912 | 1.0626185 | 0.00184325 | 0.03771774 | 86.8412386 | 20.7979765 | 9.14403695 | 24.9069129 | 105.138417 | 195.086717 | 238.211386 | 41.6823442 |
| Eno3 | ENSMUSG00000060600 | 329.686112 | 191.161232 | 468.210993 | 1.92258217 | 0.94304526 | 0.00184468 | 0.03771774 | 131.205784 | 158.064621 | 242.316979 | 233.057543 | 335.671276 | 309.483116 | 1001.54069 | 226.148889 |
| Reep3 | ENSMUSG00000019873 | 2815.73154 | 3253.77549 | 2377.68759 | 0.74083691 | -0.4327721 | 0.00184769 | 0.03773217 | 3825.73413 | 3286.08028 | 2531.06943 | 3372.21811 | 2325.58462 | 2582.09015 | 2151.79899 | 2451.27658 |
| Shroom1 | ENSMUSG00000018387 | 508.115602 | 369.355796 | 646.875409 | 1.63214032 | 0.70676509 | 0.00186036 | 0.03789609 | 354.916366 | 537.627691 | 343.815789 | 241.063336 | 444.667984 | 603.645285 | 776.490154 | 762.698213 |
| Tmod2 | ENSMUSG00000032186 | 3422.76269 | 4071.64839 | 2773.877 | 0.69822253 | -0.5182412 | 0.0018724 | 0.03802835 | 4673.38013 | 4353.01647 | 3656.70038 | 3603.49658 | 2263.85197 | 2924.25796 | 2297.88442 | 3609.51363 |
| Epdr1 | ENSMUSG00000002808 | 2370.37118 | 2811.5429 | 1929.19946 | 0.70233275 | -0.5097734 | 0.00187256 | 0.03802835 | 3592.58428 | 2964.75154 | 2293.32447 | 2395.51131 | 2335.23035 | 1809.91446 | 1700.38183 | 1871.2712 |
| Kcnv1 | ENSMUSG00000022342 | 115.606368 | 175.342247 | 55.8704882 | 0.49063839 | -1.027268 | 0.00187383 | 0.03802835 | 161.411433 | 181.982294 | 210.31285 | 147.662412 | 41.4766232 | 92.9470744 | 6.58042503 | 82.47783 |
| Vps37b | ENSMUSG00000066278 | 845.789824 | 630.993219 | 1060.58643 | 1.58762074 | 0.66686631 | 0.00188071 | 0.03812049 | 577.683022 | 686.333223 | 602.592035 | 657.364595 | 1678.35638 | 736.42682 | 804.127939 | 1023.43458 |
| Fam227a | ENSMUSG00000042564 | 109.904331 | 81.5049087 | 138.303753 | 1.59658601 | 0.67499028 | 0.00188423 | 0.03814464 | 84.009459 | 68.6333223 | 95.0979843 | 78.2788692 | 107.067562 | 169.551806 | 132.924586 | 143.671059 |
| Idh1 | ENSMUSG00000025950 | 1951.85257 | 2268.45222 | 1635.25292 | 0.73222031 | -0.4496503 | 0.00189546 | 0.03823955 | 2829.89167 | 2308.57539 | 2094.89886 | 1840.44296 | 1759.38048 | 1741.4809 | 1479.27955 | 1560.87076 |
| Luzp1 | ENSMUSG00000001089 | 794.801276 | 887.196102 | 702.40645 | 0.79641747 | -0.3284032 | 0.0018982 | 0.03823955 | 873.132018 | 890.153392 | 890.629199 | 894.869801 | 780.339261 | 637.351367 | 697.525054 | 694.410117 |
| Trmt1 | ENSMUSG00000001909 | 1827.45704 | 1495.57318 | 2159.34089 | 1.41233439 | 0.49808171 | 0.00190061 | 0.03823955 | 1441.37577 | 1321.7114 | 1602.03527 | 1617.17028 | 1827.86514 | 1927.37505 | 2920.39263 | 1961.73075 |
| Mlc1 | ENSMUSG00000035805 | 278.900626 | 180.39045 | 377.410803 | 1.80438682 | 0.85150865 | 0.00190063 | 0.03823955 | 129.317931 | 298.450962 | 139.903765 | 153.889141 | 596.105888 | 224.707213 | 352.710782 | 336.119329 |
| Tmem204 | ENSMUSG00000024168 | 271.332819 | 347.576086 | 195.089553 | 0.60626092 | -0.7219893 | 0.00190807 | 0.0383421 | 248.252671 | 430.518113 | 454.458636 | 257.074923 | 181.339655 | 257.391898 | 188.200156 | 153.426501 |
| Kcne4 | ENSMUSG00000047330 | 128.574623 | 173.31884 | 83.8304066 | 0.55796823 | -0.8417451 | 0.00191785 | 0.03849139 | 240.701259 | 159.10452 | 128.016517 | 165.453065 | 136.969314 | 61.2837853 | 77.6490154 | 59.4195119 |
| Myh14 | ENSMUSG00000030739 | 3444.70197 | 2927.22821 | 3962.17572 | 1.33714453 | 0.41915542 | 0.00192222 | 0.03853171 | 3093.24716 | 3537.73579 | 2699.31971 | 2378.61019 | 3707.8172 | 4150.95506 | 4006.16276 | 3983.76787 |
| Arl6ip4 | ENSMUSG00000029404 | 1020.32371 | 832.227705 | 1208.41972 | 1.41950366 | 0.50538657 | 0.00193118 | 0.0386639 | 744.758013 | 743.527658 | 889.714795 | 950.910355 | 1591.54485 | 1081.65881 | 1142.36179 | 1018.11343 |
| Lmo1 | ENSMUSG00000036111 | 584.001757 | 719.129659 | 448.873854 | 0.65225171 | -0.6164993 | 0.00194359 | 0.03886471 | 590.897993 | 702.971604 | 823.877729 | 758.771312 | 282.619782 | 580.153168 | 434.308052 | 498.414413 |
| Fbxo28 | ENSMUSG00000047539 | 1571.35774 | 1743.04419 | 1399.67128 | 0.80744462 | -0.3085648 | 0.00194827 | 0.03887155 | 1842.54454 | 1726.23205 | 1669.70115 | 1733.69905 | 1465.18583 | 1400.33449 | 1245.01642 | 1488.14837 |
| Tnfaip3 | ENSMUSG00000019850 | 220.047942 | 295.117068 | 144.978816 | 0.56278893 | -0.8293341 | 0.0019531 | 0.03891185 | 301.112555 | 254.775212 | 168.25028 | 456.330226 | 198.701963 | 151.16667 | 90.8098655 | 139.236767 |
| Rhoa | ENSMUSG00000007815 | 2873.67696 | 3274.81252 | 2472.5414 | 0.76282829 | -0.3905697 | 0.00197123 | 0.03919602 | 3603.9114 | 3314.15755 | 3165.66559 | 3015.51553 | 2394.06928 | 2812.92575 | 2043.88002 | 2639.29056 |
| Tcf12 | ENSMUSG00000032228 | 5925.4489 | 6738.02054 | 5112.87725 | 0.76605959 | -0.3844715 | 0.00197216 | 0.03919602 | 7612.76727 | 6147.88184 | 6201.48586 | 6989.94721 | 4887.48953 | 4766.8571 | 4880.04321 | 5917.11916 |
| Cbl | ENSMUSG00000034342 | 2597.49629 | 3076.83506 | 2118.15751 | 0.70447003 | -0.5053898 | 0.00198321 | 0.03936059 | 3720.95829 | 2787.96874 | 2763.32797 | 3035.08525 | 1996.66535 | 2418.66673 | 1568.77333 | 2488.52463 |
| Cxadr | ENSMUSG00000022865 | 1866.19644 | 2401.66209 | 1330.73078 | 0.60185906 | -0.7325024 | 0.00198526 | 0.03936059 | 2075.69439 | 2835.80409 | 2787.10246 | 1908.04744 | 1208.60951 | 2001.93699 | 668.571183 | 1443.80545 |
| Kcnmb4 | ENSMUSG00000054934 | 155.332329 | 112.698041 | 197.966616 | 1.63479104 | 0.70910624 | 0.00200268 | 0.03962674 | 81.1776795 | 155.984823 | 103.327618 | 110.302043 | 180.375082 | 257.391898 | 182.935816 | 171.163669 |
| Plekhg4 | ENSMUSG00000014782 | 92.1900328 | 62.3435237 | 122.036542 | 1.74337986 | 0.80188695 | 0.00200491 | 0.03962674 | 47.1963253 | 71.7530188 | 53.0354143 | 77.3893366 | 84.8823918 | 124.610363 | 106.602886 | 172.050527 |
| F420014N23Rik | ENSMUSG00000097331 | 40.2332479 | 24.8615443 | 55.6049514 | 1.85493519 | 0.89136878 | 0.00200636 | 0.03962674 | 16.0467506 | 37.4363576 | 21.9456887 | 24.0173803 | 49.1932043 | 44.9414426 | 69.7525054 | 58.5326535 |
| Igdcc4 | ENSMUSG00000032816 | 1260.28528 | 958.691515 | 1561.87905 | 1.55129686 | 0.63347479 | 0.00200838 | 0.03962674 | 766.468323 | 856.87663 | 1080.82517 | 1130.59594 | 1700.54155 | 908.021419 | 1874.10505 | 1764.84819 |
| Acad12 | ENSMUSG00000042647 | 74.344738 | 52.2271897 | 96.4622864 | 1.68477495 | 0.75255589 | 0.00201223 | 0.03965481 | 50.9720313 | 53.0348399 | 63.0938549 | 41.8080324 | 94.5281181 | 69.4549567 | 109.235056 | 112.631015 |
| Acad10 | ENSMUSG00000042647 | 74.344738 | 52.2271897 | 96.4622864 | 1.68477495 | 0.75255589 | 0.00201223 | 0.03965481 | 50.9720313 | 53.0348399 | 63.0938549 | 41.8080324 | 94.5281181 | 69.4549567 | 109.235056 | 112.631015 |
| Dlg4 | ENSMUSG00000020886 | 3882.64309 | 4399.72411 | 3365.56208 | 0.77155559 | -0.374158 | 0.00201593 | 0.03967985 | 4339.23015 | 4216.78973 | 4672.60288 | 4370.27369 | 3020.07692 | 4042.68704 | 3336.27549 | 3063.20887 |
| Tnks1bp1 | ENSMUSG00000033955 | 1693.34941 | 1900.32935 | 1486.36947 | 0.78707466 | -0.3454276 | 0.00204885 | 0.04023085 | 1715.11446 | 2055.87997 | 1929.3918 | 1900.93118 | 1358.11827 | 1556.60815 | 1651.68668 | 1379.06479 |
| Slc26a2 | ENSMUSG00000034320 | 847.887466 | 437.936222 | 1257.83871 | 1.99869598 | 0.99905904 | 0.00205865 | 0.04037468 | 507.83246 | 471.074167 | 320.955697 | 451.882563 | 1673.53352 | 583.217357 | 2171.54026 | 603.063703 |
| Gjc3 | ENSMUSG00000056966 | 12319.8963 | 9158.02736 | 15481.7652 | 1.59191651 | 0.67076468 | 0.0020642 | 0.04043508 | 8363.18884 | 12590.055 | 5100.54381 | 10578.3217 | 14386.6008 | 15866.372 | 16277.3394 | 15396.7485 |
| Fryl | ENSMUSG00000070733 | 3369.8493 | 2514.32375 | 4225.37484 | 1.58552469 | 0.66496035 | 0.00207394 | 0.040524 | 2751.54577 | 2803.56723 | 1873.61317 | 2628.56885 | 6628.54314 | 3324.64535 | 3020.41509 | 3927.8958 |
| Arhgef6 | ENSMUSG00000031133 | 457.957864 | 349.043461 | 566.872267 | 1.54788365 | 0.63029703 | 0.00208675 | 0.04068169 | 374.738823 | 403.480743 | 284.379549 | 333.574727 | 804.453576 | 530.104743 | 538.278768 | 394.651982 |
| Cyp3a13 | ENSMUSG00000029727 | 365.016315 | 213.467466 | 516.565164 | 1.90705111 | 0.93134351 | 0.00210934 | 0.04106763 | 120.822593 | 192.381282 | 157.277435 | 383.388553 | 187.127091 | 782.389659 | 561.968298 | 534.775607 |
| Ifit2 | ENSMUSG00000045932 | 1681.3502 | 2087.14873 | 1275.55167 | 0.64302707 | -0.6370486 | 0.00211168 | 0.04106763 | 2147.4328 | 2311.69508 | 1454.81628 | 2434.65074 | 1500.87502 | 1498.38855 | 762.013219 | 1340.92988 |
| Trim9 | ENSMUSG00000021071 | 3803.5063 | 4992.61206 | 2614.40055 | 0.58261788 | -0.7793781 | 0.00211465 | 0.04106763 | 7214.43029 | 4519.40028 | 4692.71976 | 3543.8979 | 2378.63611 | 3577.95167 | 1213.43038 | 3287.58404 |
| Fgf12 | ENSMUSG00000022523 | 890.445463 | 1159.06788 | 621.823048 | 0.59083234 | -0.7591793 | 0.0021166 | 0.04106763 | 842.92637 | 1699.19468 | 1217.07132 | 877.079149 | 456.242856 | 1004.03268 | 448.784987 | 578.231668 |
| Hacd3 | ENSMUSG00000033629 | 3674.6929 | 4147.59207 | 3201.79372 | 0.77829112 | -0.3616182 | 0.00212249 | 0.04108419 | 4237.28609 | 4216.78973 | 4241.91874 | 3894.37374 | 2858.02871 | 3497.26135 | 2836.16319 | 3615.72164 |
| Pax6 | ENSMUSG00000027168 | 190.952979 | 143.410436 | 238.495522 | 1.57326136 | 0.65375836 | 0.00212679 | 0.04111885 | 168.018918 | 142.466139 | 158.191839 | 104.964847 | 302.875807 | 197.129509 | 255.320491 | 198.656279 |
| Mrpl39 | ENSMUSG00000022889 | 575.321279 | 681.364996 | 469.277561 | 0.70523189 | -0.5038304 | 0.00213196 | 0.04113142 | 716.440218 | 587.542835 | 719.635708 | 701.841225 | 545.948111 | 554.618257 | 360.607292 | 415.936583 |
| Ptpn9 | ENSMUSG00000032290 | 1757.59782 | 2088.08047 | 1427.11517 | 0.70073068 | -0.513068 | 0.00213248 | 0.04113142 | 2298.46104 | 2213.94459 | 1824.23537 | 2015.68088 | 1604.08429 | 1459.57549 | 968.638565 | 1676.16235 |
| Dmrta2 | ENSMUSG00000047143 | 95.0220989 | 42.8390916 | 147.205106 | 2.04761608 | 1.03394524 | 0.00214006 | 0.04122901 | 64.1870024 | 33.2767623 | 67.6658734 | 6.22672823 | 121.536152 | 67.4121638 | 173.723221 | 226.148889 |
| Bcl2l13 | ENSMUSG00000009112 | 2056.48244 | 2391.79005 | 1721.17483 | 0.73159952 | -0.450874 | 0.00214561 | 0.0412414 | 2697.74195 | 2373.04911 | 2117.75896 | 2378.61019 | 1938.79099 | 1821.14982 | 1281.8668 | 1842.89173 |
| Acap2 | ENSMUSG00000049076 | 1602.50098 | 1843.19921 | 1361.80275 | 0.74834748 | -0.4182198 | 0.00214666 | 0.0412414 | 1767.97435 | 2140.11178 | 1569.11674 | 1895.59398 | 1330.14566 | 1614.82774 | 1235.80382 | 1266.43378 |
| Eif1a | ENSMUSG00000057561 | 1003.87111 | 1181.46682 | 826.275403 | 0.71330151 | -0.4874161 | 0.00214828 | 0.0412414 | 1254.47833 | 884.953898 | 1230.78737 | 1355.64769 | 920.202292 | 792.603623 | 909.41474 | 682.880958 |
| Iffo2 | ENSMUSG00000041025 | 1040.09536 | 1309.74241 | 770.448304 | 0.62614718 | -0.6754263 | 0.00216345 | 0.04148386 | 1583.90868 | 974.385197 | 1228.95857 | 1451.71721 | 1204.75122 | 677.185828 | 663.306843 | 536.549324 |
| Rnd3 | ENSMUSG00000017144 | 531.023558 | 637.615611 | 424.431505 | 0.68497397 | -0.5458789 | 0.0021685 | 0.04150613 | 682.458864 | 626.019091 | 694.032404 | 547.952085 | 501.577769 | 323.782666 | 521.169663 | 351.195921 |
| Kcnmb4os2 | ENSMUSG00000085837 | 64.7089606 | 40.5086994 | 88.9092218 | 1.83800098 | 0.87813754 | 0.00216969 | 0.04150613 | 34.9252807 | 60.3141317 | 35.6617441 | 31.1336412 | 124.42987 | 53.1126139 | 97.3902905 | 80.7041132 |
| Pde5a | ENSMUSG00000053965 | 363.103977 | 462.639041 | 263.568912 | 0.61218785 | -0.7079537 | 0.00217827 | 0.04158587 | 407.776251 | 484.592851 | 627.280935 | 330.906129 | 217.993415 | 247.177934 | 389.561162 | 199.543137 |
| Rap1b | ENSMUSG00000052681 | 2870.94336 | 3220.53275 | 2521.35397 | 0.7882664 | -0.3432448 | 0.00217895 | 0.04158587 | 3210.29405 | 3159.21262 | 2989.18568 | 3523.43865 | 2375.7424 | 2909.95841 | 2338.68306 | 2461.03202 |
| Lrrfip1 | ENSMUSG00000026305 | 2979.21522 | 3547.53072 | 2410.89972 | 0.69727944 | -0.5201912 | 0.00218295 | 0.04161369 | 3737.00504 | 3142.57424 | 3316.5422 | 3994.0014 | 3127.14448 | 2618.86043 | 1780.66301 | 2116.93097 |
| Rab10 | ENSMUSG00000020671 | 5191.4461 | 6043.51816 | 4339.37404 | 0.73002424 | -0.4539837 | 0.00218787 | 0.04165876 | 6476.27976 | 6157.24093 | 5321.8295 | 6218.72244 | 4261.48189 | 4878.18931 | 3269.15516 | 4948.6698 |
| Rab11fip4 | ENSMUSG00000017639 | 1453.30783 | 1009.2268 | 1897.38886 | 1.7000317 | 0.76556164 | 0.00219394 | 0.04166566 | 1056.25376 | 1034.69933 | 1132.03177 | 813.922334 | 1413.09891 | 1003.01129 | 3332.32724 | 1841.11801 |
| Trp53bp2 | ENSMUSG00000026510 | 818.122309 | 699.290159 | 936.954458 | 1.32421477 | 0.40513713 | 0.00219926 | 0.04166566 | 728.711263 | 771.604926 | 585.218365 | 711.626084 | 830.497037 | 904.95723 | 1060.76452 | 951.599049 |
| Hyal2 | ENSMUSG00000010047 | 731.455732 | 861.205423 | 601.706042 | 0.71344931 | -0.4871172 | 0.0021995 | 0.04166566 | 1100.61831 | 676.974133 | 826.62094 | 840.608312 | 653.980246 | 553.596861 | 555.387873 | 643.859189 |
| Nell2 | ENSMUSG00000022454 | 6612.67771 | 8062.13485 | 5163.22058 | 0.6655362 | -0.5874109 | 0.00220041 | 0.04166566 | 10579.5283 | 6240.43283 | 8129.96325 | 7298.61502 | 4316.46254 | 5978.23326 | 4015.37536 | 6342.81118 |
| Nptn | ENSMUSG00000032336 | 4090.3212 | 4791.37513 | 3389.26728 | 0.72083735 | -0.4722543 | 0.00220098 | 0.04166566 | 4972.60483 | 5094.46433 | 4720.15187 | 4378.27948 | 3766.65613 | 4031.45168 | 2451.86637 | 3307.09492 |
| Prickle1 | ENSMUSG00000036158 | 341.117971 | 278.915254 | 403.320687 | 1.41252997 | 0.49828148 | 0.00220585 | 0.04170946 | 236.925553 | 240.216628 | 320.955697 | 317.56314 | 443.703411 | 376.89528 | 426.411542 | 366.272514 |
| Cdc14b | ENSMUSG00000033102 | 328.218919 | 255.395276 | 401.042561 | 1.50949777 | 0.59406862 | 0.00221548 | 0.04184313 | 200.112419 | 292.211569 | 195.682391 | 333.574727 | 434.057685 | 360.552937 | 382.980737 | 426.578884 |
| Slc44a1 | ENSMUSG00000028412 | 5255.94573 | 4281.41399 | 6230.47747 | 1.42120594 | 0.50711562 | 0.00222669 | 0.041961 | 4339.23015 | 4578.67452 | 3402.49615 | 4805.25513 | 5918.61768 | 5380.71635 | 8178.15223 | 5444.42364 |
| Mamdc2 | ENSMUSG00000033207 | 109.412851 | 54.0707498 | 164.754953 | 2.01156173 | 1.00831601 | 0.00222686 | 0.041961 | 87.7851651 | 37.4363576 | 44.805781 | 46.2556955 | 217.993415 | 61.2837853 | 313.228232 | 66.514379 |
| Stat3 | ENSMUSG00000004040 | 6747.81034 | 8143.04934 | 5352.57134 | 0.6790576 | -0.5583941 | 0.00223274 | 0.04202328 | 9995.23777 | 6973.5615 | 7722.1392 | 7881.25888 | 7128.19176 | 4900.66003 | 3921.93332 | 5459.50023 |
| Slc15a4 | ENSMUSG00000029416 | 608.378991 | 527.562997 | 689.194985 | 1.29508807 | 0.3730502 | 0.00223629 | 0.0420418 | 551.25308 | 502.271131 | 489.205977 | 567.521802 | 721.50033 | 604.666682 | 688.312459 | 742.30047 |
| Cblb | ENSMUSG00000022637 | 481.181362 | 574.50195 | 387.860775 | 0.69321652 | -0.5286221 | 0.00224382 | 0.04213479 | 617.327935 | 624.979192 | 549.556621 | 506.144052 | 282.619782 | 483.120507 | 415.882862 | 369.819947 |
| Matn2 | ENSMUSG00000022324 | 542.393876 | 360.573832 | 724.21392 | 1.76037853 | 0.81588568 | 0.00225049 | 0.04221146 | 544.645594 | 189.261586 | 273.406705 | 434.981444 | 742.720928 | 486.184697 | 1089.71839 | 578.231668 |
| Pithd1 | ENSMUSG00000028669 | 1604.22458 | 1891.74375 | 1316.7054 | 0.71077334 | -0.4925385 | 0.0022564 | 0.04227378 | 2107.78789 | 1454.81845 | 1754.74069 | 2249.62796 | 1467.11498 | 1270.61715 | 1459.53827 | 1069.55121 |
| Eif4g1 | ENSMUSG00000045983 | 8992.14033 | 10538.8861 | 7445.39459 | 0.71994508 | -0.4740412 | 0.00226291 | 0.04234726 | 11829.287 | 9103.27429 | 10582.394 | 10640.589 | 9131.60912 | 7038.44274 | 7722.78682 | 5888.73969 |
| Apold1 | ENSMUSG00000090698 | 354.785219 | 465.062204 | 244.508235 | 0.58466871 | -0.7743087 | 0.00229313 | 0.04286376 | 461.580062 | 402.440844 | 423.368911 | 572.858998 | 402.226788 | 234.921177 | 215.837941 | 125.047033 |
| Wac | ENSMUSG00000024283 | 1739.81045 | 1943.46599 | 1536.15491 | 0.79557218 | -0.3299353 | 0.00230202 | 0.04287123 | 1967.14284 | 2034.0421 | 1805.03289 | 1967.64612 | 1435.28408 | 1727.18135 | 1388.46968 | 1593.68452 |
| Dynll2 | ENSMUSG00000020483 | 7052.75421 | 5293.43096 | 8812.07745 | 1.57347585 | 0.65395504 | 0.00230252 | 0.04287123 | 3631.28527 | 7482.07203 | 5583.34896 | 4477.0176 | 6158.79627 | 9347.82005 | 8854.61993 | 10887.0736 |
| Rhob | ENSMUSG00000054364 | 5431.35654 | 6566.40088 | 4296.3122 | 0.67674212 | -0.5633219 | 0.00230321 | 0.04287123 | 8310.32896 | 7163.86299 | 5708.62227 | 5082.7893 | 3680.80917 | 5504.30532 | 3512.63088 | 4487.50344 |
| Stab2 | ENSMUSG00000035459 | 41.4177889 | 15.3620476 | 67.4735303 | 2.05941875 | 1.04223721 | 0.00230403 | 0.04287123 | 2.83177952 | 24.9575717 | 27.4321108 | 6.22672823 | 24.1143158 | 52.0912175 | 131.608501 | 62.0800871 |
| 5330434G04Rik | ENSMUSG00000087620 | 76.1584999 | 102.213347 | 50.1036525 | 0.56366022 | -0.8271024 | 0.00231084 | 0.04294901 | 72.682341 | 136.226746 | 123.444499 | 76.499804 | 32.7954695 | 55.1554068 | 56.5916553 | 55.8720784 |
| Clic1 | ENSMUSG00000007041 | 2193.57141 | 2789.90316 | 1597.23967 | 0.61545964 | -0.7002639 | 0.00232572 | 0.04317653 | 3116.84532 | 2036.12189 | 3224.18743 | 2782.45799 | 2201.15475 | 1682.23991 | 1708.27834 | 797.28569 |
| Ackr2 | ENSMUSG00000044534 | 92.7645645 | 150.213581 | 35.3155482 | 0.4861749 | -1.0404527 | 0.00233963 | 0.04333057 | 172.738551 | 123.74796 | 136.246151 | 168.121662 | 84.8823918 | 38.813064 | 15.7930201 | 1.77371677 |
| Zfp521 | ENSMUSG00000024420 | 1414.05223 | 1730.12141 | 1097.98306 | 0.66156196 | -0.5960518 | 0.00234068 | 0.04333057 | 2130.44212 | 1994.52594 | 1330.45738 | 1465.0602 | 1206.68036 | 1340.07211 | 733.059349 | 1112.12042 |
| Agbl5 | ENSMUSG00000029165 | 593.715694 | 415.439024 | 771.992364 | 1.68769305 | 0.75505253 | 0.00234198 | 0.04333057 | 388.897721 | 569.864555 | 369.419093 | 333.574727 | 1295.42105 | 695.570963 | 622.508208 | 474.469237 |
| Tmem243 | ENSMUSG00000079659 | 351.069798 | 277.339643 | 424.799953 | 1.47884362 | 0.56446951 | 0.00236358 | 0.04368076 | 222.766655 | 305.730254 | 261.519457 | 319.342205 | 489.038325 | 348.29618 | 508.008813 | 353.856496 |
| Irgq | ENSMUSG00000041037 | 1462.72981 | 1640.88829 | 1284.57134 | 0.78826914 | -0.3432398 | 0.00236961 | 0.04371869 | 1707.56305 | 1539.05026 | 1794.06005 | 1522.87982 | 1353.2954 | 1119.45048 | 1306.87241 | 1358.66705 |
| Ankhd1 | ENSMUSG00000024483 | 1084.42338 | 1212.76307 | 956.083691 | 0.79277904 | -0.3350093 | 0.00237151 | 0.04371869 | 1360.1981 | 1156.36749 | 1157.63508 | 1176.85164 | 963.608061 | 898.828851 | 1052.86801 | 909.029846 |
| Lzts1 | ENSMUSG00000036306 | 54.8179146 | 22.9858796 | 86.6499497 | 2.04765166 | 1.03397031 | 0.00237533 | 0.04371869 | 11.3271181 | 29.117167 | 29.2609182 | 22.2383151 | 152.402476 | 21.4493249 | 140.821096 | 31.9269019 |
| Cggbp1 | ENSMUSG00000054604 | 2024.76862 | 2392.92745 | 1656.60978 | 0.708283 | -0.4976022 | 0.00237688 | 0.04371869 | 2164.42348 | 2383.4481 | 2542.04227 | 2481.79597 | 1803.75082 | 2098.96965 | 1175.26391 | 1548.45474 |
| Iglon5 | ENSMUSG00000013367 | 2474.29589 | 2915.63655 | 2032.95524 | 0.71240161 | -0.4892373 | 0.0023797 | 0.04371869 | 3365.09799 | 2530.07384 | 3159.26477 | 2608.1096 | 2588.91295 | 2061.17798 | 1609.57196 | 1872.15805 |
| Zfp719 | ENSMUSG00000030469 | 486.444929 | 554.739123 | 418.150735 | 0.76260142 | -0.3909989 | 0.00238174 | 0.04371869 | 526.71099 | 572.984251 | 597.105613 | 522.155639 | 478.428026 | 401.408794 | 377.716397 | 415.049725 |
| Chm | ENSMUSG00000025531 | 1013.83723 | 1165.76945 | 861.905013 | 0.74927538 | -0.416432 | 0.0023919 | 0.04371869 | 1217.66519 | 1340.42958 | 993.956816 | 1111.02622 | 835.319901 | 1005.05408 | 756.748879 | 850.497193 |
| Myd88 | ENSMUSG00000032508 | 292.454763 | 359.639303 | 225.270224 | 0.65548965 | -0.6093551 | 0.00239332 | 0.04371869 | 497.449269 | 305.730254 | 303.582027 | 331.795662 | 235.355723 | 268.627259 | 184.251901 | 212.846013 |
| Zfp24 | ENSMUSG00000051469 | 3422.28661 | 3933.00097 | 2911.57224 | 0.74974273 | -0.4155325 | 0.00239486 | 0.04371869 | 3720.01436 | 4578.67452 | 3546.05753 | 3887.25748 | 2824.26867 | 3545.26698 | 2634.80218 | 2641.95113 |
| Nox4 | ENSMUSG00000030562 | 124.462269 | 169.610028 | 79.3145106 | 0.5512583 | -0.8591996 | 0.00239505 | 0.04371869 | 228.430214 | 134.146948 | 166.421472 | 149.441478 | 107.067562 | 72.5191459 | 103.970716 | 33.7006187 |
| Tha1 | ENSMUSG00000017713 | 139.278401 | 97.1834789 | 181.373323 | 1.68742569 | 0.75482397 | 0.00239507 | 0.04371869 | 101.000136 | 71.7530188 | 124.358902 | 91.6218583 | 155.296194 | 125.63176 | 264.533086 | 180.032253 |
| Cbarp | ENSMUSG00000035640 | 2879.78753 | 3302.66569 | 2456.90938 | 0.75291179 | -0.4094472 | 0.00239885 | 0.04372854 | 3031.89194 | 3683.32163 | 3535.08468 | 2960.36451 | 1994.73621 | 2779.21966 | 2472.92373 | 2580.75791 |
| Sft2d2 | ENSMUSG00000040848 | 739.656816 | 529.201467 | 950.112166 | 1.65219105 | 0.72438052 | 0.00240096 | 0.04372854 | 527.654917 | 622.899395 | 400.508818 | 565.742737 | 1474.83156 | 557.682446 | 1069.97711 | 697.95755 |
| Zfp407 | ENSMUSG00000048410 | 463.878291 | 546.190503 | 381.566078 | 0.71411887 | -0.4857638 | 0.00241897 | 0.04400744 | 636.206465 | 479.393357 | 521.210106 | 547.952085 | 448.526275 | 318.675684 | 336.917762 | 422.144592 |
| Ch25h | ENSMUSG00000050370 | 40.4822726 | 60.1331922 | 20.831353 | 0.50386533 | -0.9888899 | 0.0024232 | 0.04403544 | 75.5141205 | 73.8328164 | 49.3777995 | 41.8080324 | 28.937179 | 30.6418927 | 18.4251901 | 5.32115032 |
| Mrps17 | ENSMUSG00000034211 | 1316.4588 | 1160.67493 | 1472.24266 | 1.26010682 | 0.33354604 | 0.00245692 | 0.04459864 | 1110.05757 | 1029.49983 | 1242.67462 | 1260.4677 | 1584.79284 | 1374.79958 | 1471.38304 | 1457.99519 |
| Snn | ENSMUSG00000037972 | 1931.25758 | 2296.40736 | 1566.1078 | 0.69968483 | -0.5152229 | 0.00248755 | 0.04510446 | 2018.11487 | 2649.6622 | 2318.92777 | 2198.9246 | 1495.08758 | 2157.18924 | 1281.8668 | 1330.28758 |
| Klhl33 | ENSMUSG00000090799 | 133.404065 | 77.7549924 | 189.053139 | 1.89960387 | 0.9256986 | 0.00249361 | 0.04516421 | 38.7009868 | 137.266645 | 92.3547732 | 42.697565 | 108.996708 | 179.76577 | 239.527471 | 227.922605 |
| Hilpda | ENSMUSG00000043421 | 219.079979 | 275.853291 | 162.306667 | 0.62769783 | -0.6718579 | 0.00250126 | 0.0452526 | 350.196734 | 184.062092 | 288.951568 | 280.202771 | 135.040169 | 158.316445 | 142.137181 | 213.732871 |
| Boc | ENSMUSG00000022687 | 356.771841 | 260.744936 | 452.798747 | 1.61699857 | 0.6933184 | 0.00251181 | 0.04532692 | 226.542361 | 366.044386 | 239.573768 | 210.819227 | 593.21217 | 405.494379 | 522.485748 | 290.002692 |
| Slc7a11 | ENSMUSG00000027737 | 266.618457 | 376.081885 | 157.155028 | 0.52969643 | -0.9167623 | 0.00251369 | 0.04532692 | 448.36509 | 218.378753 | 640.99699 | 196.586706 | 235.355723 | 150.145274 | 71.0685904 | 172.050527 |
| Csgalnact1 | ENSMUSG00000036356 | 404.231852 | 321.725619 | 486.738086 | 1.46486324 | 0.55076598 | 0.00252384 | 0.04545974 | 305.832188 | 373.323677 | 268.834686 | 338.911923 | 350.139866 | 474.949336 | 576.445233 | 545.417908 |
| Dab2ip | ENSMUSG00000026883 | 1839.03478 | 2077.06593 | 1601.00363 | 0.77704067 | -0.363938 | 0.00254609 | 0.04578587 | 2321.11528 | 2205.6254 | 1933.96381 | 1847.55922 | 1431.42579 | 1679.17572 | 1760.92174 | 1532.49129 |
| Cttnbp2nl | ENSMUSG00000062127 | 3645.3566 | 4323.00271 | 2967.71049 | 0.70349585 | -0.5073862 | 0.00254755 | 0.04578587 | 5536.12896 | 4685.78409 | 3175.72403 | 3894.37374 | 3203.34572 | 3184.71404 | 2526.88321 | 2955.899 |
| Nkd1 | ENSMUSG00000031661 | 624.882913 | 767.383024 | 482.382802 | 0.65695395 | -0.6061358 | 0.00255671 | 0.04586169 | 621.103641 | 883.913999 | 974.754339 | 589.760117 | 344.35243 | 593.431321 | 511.957068 | 479.790387 |
| Ccdc30 | ENSMUSG00000028637 | 35.2317477 | 22.5856252 | 47.8778702 | 1.79542991 | 0.84432933 | 0.00255739 | 0.04586169 | 25.4860157 | 19.7580776 | 22.8600924 | 22.2383151 | 51.1223496 | 40.8558569 | 63.1720803 | 36.3611939 |
| Ypel2 | ENSMUSG00000018427 | 333.619273 | 258.194666 | 409.04388 | 1.51677863 | 0.60101055 | 0.00257068 | 0.04604957 | 295.448996 | 256.855009 | 299.010008 | 181.464651 | 367.502173 | 319.69708 | 489.583623 | 459.392644 |
| Mast4 | ENSMUSG00000034751 | 661.068279 | 807.549349 | 514.587209 | 0.66431798 | -0.5900541 | 0.00258039 | 0.04617294 | 858.029194 | 969.185703 | 791.8736 | 611.1089 | 393.545634 | 560.746636 | 418.515032 | 685.541533 |
| Evi2a | ENSMUSG00000078771 | 169.216352 | 118.991024 | 219.441681 | 1.67751196 | 0.74632305 | 0.00258583 | 0.04621955 | 115.159034 | 124.787859 | 108.81404 | 127.203163 | 115.748716 | 234.921177 | 218.470111 | 308.626719 |
| Sbsn | ENSMUSG00000046056 | 109.783005 | 79.3313282 | 140.234681 | 1.63242691 | 0.7070184 | 0.00259744 | 0.0463051 | 63.2430759 | 90.4711976 | 63.0938549 | 100.517184 | 110.925853 | 131.760138 | 176.355391 | 141.897342 |
| 4833439L19Rik | ENSMUSG00000025871 | 4235.7753 | 4777.90701 | 3693.64359 | 0.77953501 | -0.3593143 | 0.00259792 | 0.0463051 | 5316.19408 | 4714.90126 | 4389.13773 | 4691.39496 | 3724.21494 | 4043.70843 | 3120.43755 | 3886.21345 |
| Mkl2 | ENSMUSG00000009569 | 694.608332 | 848.914859 | 540.301806 | 0.66285511 | -0.5932345 | 0.00259912 | 0.0463051 | 893.898401 | 934.869041 | 865.940299 | 700.951693 | 472.64059 | 735.405424 | 594.870423 | 358.290788 |
| Ahcyl2 | ENSMUSG00000029772 | 1649.04619 | 1918.52569 | 1379.56669 | 0.73157557 | -0.4509212 | 0.00261252 | 0.04644326 | 1839.71276 | 1709.59366 | 1865.38354 | 2259.41282 | 1437.21322 | 1437.10477 | 1047.60367 | 1596.3451 |
| Usp7 | ENSMUSG00000022710 | 3190.62244 | 3584.87162 | 2796.37325 | 0.78560281 | -0.348128 | 0.00261265 | 0.04644326 | 3461.3785 | 3555.41407 | 3697.84854 | 3624.84537 | 2747.10286 | 3192.88521 | 2900.65135 | 2344.85357 |
| 6030419C18Rik | ENSMUSG00000066607 | 1342.4234 | 1737.28013 | 947.566674 | 0.59863469 | -0.7402522 | 0.0026196 | 0.04644326 | 2634.49888 | 1534.89066 | 1763.88473 | 1015.84623 | 863.292507 | 1029.56759 | 577.761318 | 1319.64528 |
| Plch2 | ENSMUSG00000029055 | 48.0752252 | 18.2487335 | 77.901717 | 2.04035491 | 1.02882012 | 0.00262305 | 0.04644326 | 9.43926506 | 24.9575717 | 12.8016517 | 25.7964455 | 26.0434611 | 15.3209463 | 105.286801 | 164.95566 |
| Gatsl3 | ENSMUSG00000020424 | 39.2330588 | 24.0640725 | 54.4020451 | 1.84260318 | 0.88174541 | 0.00262393 | 0.04644326 | 25.4860157 | 23.9176729 | 21.9456887 | 24.9069129 | 39.547478 | 62.3051817 | 80.2811854 | 35.4743355 |
| Hps4 | ENSMUSG00000042328 | 536.611745 | 438.921033 | 634.302457 | 1.41091903 | 0.4966352 | 0.00263433 | 0.0465448 | 395.505206 | 393.081755 | 431.598544 | 535.498628 | 679.059134 | 500.484247 | 750.168454 | 607.497995 |
| Zdhhc1 | ENSMUSG00000039199 | 691.409749 | 535.617305 | 847.202193 | 1.51423275 | 0.59858698 | 0.00263562 | 0.0465448 | 553.140933 | 556.34587 | 630.024146 | 402.95827 | 612.503622 | 824.266912 | 1185.79259 | 766.245646 |
| Cnppd1 | ENSMUSG00000033159 | 1255.84627 | 1116.73502 | 1394.95752 | 1.24244248 | 0.31317906 | 0.00263857 | 0.0465448 | 1228.04838 | 1010.78166 | 1138.4326 | 1089.67744 | 1306.99592 | 1468.76805 | 1372.67666 | 1431.38944 |
| Ctdp1 | ENSMUSG00000033323 | 739.770677 | 841.217416 | 638.323937 | 0.76662196 | -0.3834128 | 0.00264106 | 0.0465448 | 895.786254 | 829.83926 | 746.153415 | 893.090735 | 681.952852 | 714.977495 | 584.341743 | 572.023659 |
| Jam2 | ENSMUSG00000053062 | 3285.07556 | 4235.44496 | 2334.70616 | 0.60240844 | -0.7311861 | 0.0026489 | 0.04658221 | 3865.37904 | 5528.10214 | 3750.88396 | 3797.41469 | 2326.54919 | 3373.67238 | 1043.65541 | 2594.94764 |
| Adamtsl1 | ENSMUSG00000066113 | 70.416012 | 36.2973819 | 104.534642 | 1.97591914 | 0.98252391 | 0.002651 | 0.04658221 | 19.8224566 | 41.5959529 | 38.4049552 | 45.3661629 | 218.957988 | 40.8558569 | 80.2811854 | 78.043538 |
| 1810041L15Rik | ENSMUSG00000062760 | 3235.62562 | 2515.85206 | 3955.39919 | 1.50859765 | 0.59320808 | 0.00265421 | 0.04658221 | 2139.88139 | 3573.09235 | 2246.68988 | 2103.74461 | 3474.39063 | 3663.74896 | 3307.32162 | 5376.13554 |
| Rab8b | ENSMUSG00000036943 | 2627.90185 | 3341.12623 | 1914.67747 | 0.61710441 | -0.6964135 | 0.00265476 | 0.04658221 | 3223.50902 | 4183.51296 | 2855.68274 | 3101.80019 | 1878.02292 | 2478.92912 | 905.466485 | 2396.29136 |
| Klf6 | ENSMUSG00000000078 | 1513.33139 | 1782.41256 | 1244.25023 | 0.71344352 | -0.4871289 | 0.00265773 | 0.04658221 | 2363.59197 | 1664.87801 | 1383.49279 | 1717.68746 | 1338.82682 | 1299.21625 | 1080.50579 | 1258.45205 |
| Sbf2 | ENSMUSG00000038371 | 4739.67817 | 5431.64387 | 4047.71248 | 0.75435639 | -0.4066818 | 0.00266029 | 0.04658221 | 5526.68969 | 5606.09455 | 4754.89921 | 5838.89202 | 4778.49283 | 3577.95167 | 3502.1022 | 4332.30322 |
| Ephb1 | ENSMUSG00000032537 | 427.159722 | 273.466479 | 580.852966 | 1.80190745 | 0.84952491 | 0.00266743 | 0.04665722 | 123.654372 | 531.388298 | 206.655235 | 232.16801 | 613.468195 | 506.612625 | 464.578007 | 738.753036 |
| Akt3 | ENSMUSG00000019699 | 2035.03439 | 2344.02376 | 1726.04502 | 0.74601263 | -0.422728 | 0.00267244 | 0.04669474 | 2572.19973 | 2169.22894 | 2394.82328 | 2239.8431 | 1851.01488 | 1460.59688 | 2104.41993 | 1488.14837 |
| Cebpd | ENSMUSG00000071637 | 606.056337 | 805.554646 | 406.558027 | 0.57372138 | -0.8015778 | 0.00268664 | 0.04689276 | 1538.60021 | 525.148905 | 566.930291 | 591.539182 | 472.64059 | 387.109244 | 471.158432 | 295.323843 |
| Dzip3 | ENSMUSG00000064061 | 1406.43579 | 1610.26803 | 1202.60355 | 0.75525488 | -0.4049645 | 0.00272557 | 0.04752149 | 1728.32943 | 1541.13005 | 1585.57601 | 1586.03663 | 1069.71105 | 1408.50567 | 1337.14237 | 995.05511 |
| Adprm | ENSMUSG00000020910 | 333.740689 | 277.156627 | 390.324751 | 1.38074119 | 0.46544292 | 0.00273621 | 0.04764868 | 264.299422 | 297.411063 | 288.951568 | 257.964455 | 323.131832 | 368.724108 | 463.261922 | 406.181141 |
| Stxbp3 | ENSMUSG00000027882 | 582.639365 | 487.52943 | 677.749301 | 1.36693875 | 0.45094859 | 0.0027387 | 0.04764868 | 416.271589 | 558.425668 | 410.567259 | 564.853204 | 645.299092 | 744.597991 | 686.996374 | 634.103747 |
| Tarbp1 | ENSMUSG00000090290 | 355.459511 | 270.442419 | 440.476604 | 1.5467788 | 0.62926689 | 0.00275888 | 0.04794868 | 388.897721 | 262.054503 | 206.655235 | 224.162216 | 451.419992 | 335.018026 | 500.112303 | 475.356095 |
| St5 | ENSMUSG00000031024 | 830.065841 | 675.920201 | 984.211482 | 1.42090642 | 0.50681154 | 0.00276803 | 0.04805669 | 613.552229 | 767.445331 | 684.888367 | 637.794878 | 1279.98788 | 727.234252 | 1002.85678 | 926.767014 |
| Capg | ENSMUSG00000056737 | 339.294847 | 435.982254 | 242.607441 | 0.60583703 | -0.7229983 | 0.00278478 | 0.0482961 | 451.19687 | 426.358517 | 522.12451 | 344.249118 | 321.202687 | 261.477484 | 269.797426 | 117.952165 |
| Junb | ENSMUSG00000052837 | 970.776273 | 1304.40885 | 637.143698 | 0.5651864 | -0.8232013 | 0.0027937 | 0.04839946 | 2146.48887 | 680.09383 | 915.318098 | 1475.73459 | 960.714343 | 575.046185 | 655.410333 | 357.40393 |
| Clip1 | ENSMUSG00000049550 | 1427.32532 | 1053.56111 | 1801.08952 | 1.59847013 | 0.67669178 | 0.00281473 | 0.04862136 | 796.673971 | 1160.52709 | 992.128009 | 1264.91536 | 2838.73726 | 1105.15093 | 1567.45724 | 1693.01266 |
| Ubn1 | ENSMUSG00000039473 | 1623.65195 | 1837.33566 | 1409.96824 | 0.77386523 | -0.3698458 | 0.00281544 | 0.04862136 | 1805.73141 | 1829.18203 | 1887.32923 | 1827.09997 | 1334.00395 | 1608.69936 | 1539.81946 | 1157.35019 |
| Sgsh | ENSMUSG00000005043 | 324.633651 | 264.386834 | 384.880468 | 1.42178282 | 0.50770111 | 0.00282153 | 0.04867508 | 226.542361 | 347.326207 | 253.289823 | 230.388945 | 382.935336 | 414.686947 | 365.871632 | 376.027956 |
| Zyx | ENSMUSG00000029860 | 2429.0603 | 2838.67007 | 2019.45054 | 0.72489953 | -0.464147 | 0.00283871 | 0.04891976 | 3021.50875 | 2599.74706 | 3023.01861 | 2710.40585 | 2621.70842 | 2105.09803 | 1684.58881 | 1666.40691 |
| Acot11 | ENSMUSG00000034853 | 144.646688 | 92.1853488 | 197.108026 | 1.80131339 | 0.8490492 | 0.00285113 | 0.04908215 | 58.5234434 | 122.708061 | 91.4403695 | 96.0695213 | 177.481365 | 203.257888 | 311.912147 | 95.7807058 |
| Pms2 | ENSMUSG00000075569 | 52.4554472 | 31.2487826 | 73.6621118 | 1.87073649 | 0.90360636 | 0.00286376 | 0.04921951 | 38.7009868 | 39.5161553 | 19.2024776 | 27.5755108 | 39.547478 | 59.2409925 | 94.7581205 | 101.101856 |
| Rlbp1 | ENSMUSG00000039194 | 6432.03887 | 4405.8788 | 8458.19894 | 1.7119433 | 0.77563492 | 0.0028664 | 0.04921951 | 7287.11263 | 3136.33485 | 4847.25399 | 2352.81374 | 9543.48164 | 5617.68032 | 10791.8971 | 7879.73677 |
| Ift88 | ENSMUSG00000040040 | 276.247394 | 213.893914 | 338.600874 | 1.51249698 | 0.59693226 | 0.00286815 | 0.04921951 | 224.654508 | 195.500979 | 245.06019 | 190.359977 | 350.139866 | 272.712845 | 461.945837 | 269.60495 |
| Selo | ENSMUSG00000035757 | 281.269372 | 233.262365 | 329.276379 | 1.38595005 | 0.47087526 | 0.00288267 | 0.04936677 | 265.243348 | 246.456021 | 185.62395 | 235.72614 | 346.281575 | 325.825459 | 305.331722 | 339.666762 |
| Parva | ENSMUSG00000030770 | 1124.49132 | 1302.96954 | 946.013103 | 0.73736326 | -0.4395526 | 0.00288278 | 0.04936677 | 1532.93665 | 1097.09326 | 1421.89775 | 1159.95052 | 1093.82537 | 941.727501 | 939.684695 | 808.814849 |
| Foxc2 | ENSMUSG00000046714 | 84.6693895 | 46.4694108 | 122.869368 | 1.93141473 | 0.94965799 | 0.00289493 | 0.04952295 | 55.6916639 | 51.9949411 | 62.1794512 | 16.0115869 | 114.784143 | 66.3907674 | 214.521856 | 95.7807058 |
| Kcna6 | ENSMUSG00000038077 | 2197.8197 | 2627.0975 | 1768.54191 | 0.69308562 | -0.5288945 | 0.00290989 | 0.04972685 | 3173.48091 | 2347.05164 | 2658.17154 | 2329.68589 | 1479.65442 | 2192.93812 | 1312.13675 | 2089.43836 |
| Tars2 | ENSMUSG00000028107 | 842.280009 | 741.228317 | 943.331702 | 1.26410391 | 0.33811505 | 0.0029276 | 0.04992802 | 715.496292 | 695.692312 | 741.581396 | 812.143268 | 976.147505 | 843.673444 | 926.523845 | 1026.98201 |
| Hars | ENSMUSG00000001380 | 1849.75462 | 2085.08878 | 1614.42047 | 0.78071681 | -0.3571288 | 0.00292777 | 0.04992802 | 2067.19905 | 1848.94011 | 2244.86107 | 2179.35488 | 1775.77822 | 1764.97302 | 1433.21657 | 1483.71408 |
