# Supplementary material for: Effects of combination therapy of a CDK4/6 and MEK inhibitor in diffuse midline glioma preclinical models
Source: PLoS One. 2025 Dec 22;20(12):e0323235. doi: 10.1371/journal.pone.0323235 (PMC12721541; doi:10.1371/journal.pone.0323235)
Supplement: S7 Table — (DOCX) [file pone.0323235.s014.docx]

**Supplemental table 7. Gene set enrichment analysis comparing tumors treated with ribociclib and those treated with vehicle**

| **Positively enriched in Ribociclib vs Vehicle** | | | | | | |
| --- | --- | --- | --- | --- | --- | --- |
| **NAME** | **SIZE** | **ES** | **NES** | **NOM p-val** | **FDR q-val** | **FWER p-val** |
| WP_DNA_MISMATCH_REPAIR | 23 | 0.62036663 | 1.6918778 | 0.008976661 | 0.17586079 | 0.985 |

| **Negatively enriched in Ribociclib vs Vehicle** | | | | | | |
| --- | --- | --- | --- | --- | --- | --- |
| **NAME** | **SIZE** | **ES** | **NES** | **NOM p-val** | **FDR q-val** | **FWER p-val** |
| HALLMARK_EPITHELIAL_MESENCHYMAL_TRANSITION | 192 | -0.5302794 | -2.1437438 | 0 | 0 | 0 |
| HALLMARK_ANGIOGENESIS | 35 | -0.6041411 | -1.8599153 | 0.002173913 | 7.75E-04 | 0.001 |
| HALLMARK_KRAS_SIGNALING_UP | 189 | -0.45524448 | -1.8367298 | 0 | 5.17E-04 | 0.001 |
| HALLMARK_PROTEIN_SECRETION | 94 | -0.49951094 | -1.8172841 | 0 | 3.88E-04 | 0.001 |
| HALLMARK_HYPOXIA | 189 | -0.4051564 | -1.6214639 | 0 | 0.007690735 | 0.029 |
| HALLMARK_GLYCOLYSIS | 191 | -0.33875483 | -1.3577577 | 0.007194245 | 0.117687054 | 0.401 |
| HALLMARK_TGF_BETA_SIGNALING | 54 | -0.40364763 | -1.3361213 | 0.059322033 | 0.12711564 | 0.474 |
| HALLMARK_COAGULATION | 132 | -0.3493548 | -1.3298134 | 0.02097902 | 0.11639156 | 0.489 |
| HALLMARK_TNFA_SIGNALING_VIA_NFKB | 196 | -0.32741818 | -1.323807 | 0.01909308 | 0.10954534 | 0.512 |
| HALLMARK_UV_RESPONSE_DN | 139 | -0.332705 | -1.2891166 | 0.04137931 | 0.13653195 | 0.618 |
| HALLMARK_COMPLEMENT | 184 | -0.31093037 | -1.248481 | 0.028037382 | 0.17912167 | 0.767 |
| HALLMARK_PI3K_AKT_MTOR_SIGNALING | 104 | -0.32710975 | -1.2166412 | 0.105032824 | 0.21511096 | 0.841 |
| HALLMARK_ANDROGEN_RESPONSE | 93 | -0.32838815 | -1.1957147 | 0.11062907 | 0.23879686 | 0.887 |
