# Supplementary material for: Effects of combination therapy of a CDK4/6 and MEK inhibitor in diffuse midline glioma preclinical models
Source: PLoS One. 2025 Dec 22;20(12):e0323235. doi: 10.1371/journal.pone.0323235 (PMC12721541; doi:10.1371/journal.pone.0323235)
Supplement: S8 Table — (DOCX) [file pone.0323235.s015.docx]

**Supplemental table 8. Gene set enrichment analysis comparing tumors treated with trametinib and those treated with vehicle**

| **Positively enriched in Trametinib vs Vehicle** | | | | | | |
| --- | --- | --- | --- | --- | --- | --- |
| **NAME** | **SIZE** | **ES** | **NES** | **NOM p-val** | **FDR q-val** | **FWER p-val** |
| HALLMARK_E2F_TARGETS | 190 | 0.6733044 | 2.6960552 | 0 | 0 | 0 |
| HALLMARK_MYC_TARGETS_V2 | 58 | 0.7001151 | 2.330352 | 0 | 0 | 0 |
| HALLMARK_G2M_CHECKPOINT | 188 | 0.5622073 | 2.2207732 | 0 | 0 | 0 |
| HALLMARK_MYC_TARGETS_V1 | 192 | 0.53736377 | 2.1551607 | 0 | 0 | 0 |
| HALLMARK_INTERFERON_ALPHA_RESPONSE | 89 | 0.50392574 | 1.8107297 | 0 | 3.76E-04 | 0.002 |
| HALLMARK_MTORC1_SIGNALING | 188 | 0.3732327 | 1.4990188 | 0.001736111 | 0.027006382 | 0.148 |
| HALLMARK_OXIDATIVE_PHOSPHORYLATION | 178 | 0.37154385 | 1.4672222 | 0.003305785 | 0.03383788 | 0.206 |
| HALLMARK_UNFOLDED_PROTEIN_RESPONSE | 107 | 0.38718227 | 1.4385302 | 0.008488964 | 0.039069954 | 0.267 |
| HALLMARK_DNA_REPAIR | 141 | 0.3692948 | 1.4148266 | 0.006666667 | 0.046249412 | 0.344 |
| HALLMARK_INTERFERON_GAMMA_RESPONSE | 185 | 0.34196514 | 1.3569248 | 0.02020202 | 0.07813505 | 0.551 |
| HALLMARK_MITOTIC_SPINDLE | 197 | 0.33413643 | 1.3328631 | 0.027732464 | 0.09165281 | 0.644 |
| HALLMARK_ANDROGEN_RESPONSE | 93 | 0.3418842 | 1.2558193 | 0.077319585 | 0.16940975 | 0.886 |
| HALLMARK_XENOBIOTIC_METABOLISM | 185 | 0.31574768 | 1.2528038 | 0.049342107 | 0.1600557 | 0.891 |
| REACTOME_HDR_THROUGH_HOMOLOGOUS_RECOMBINATION_HRR | 65 | 0.6563015 | 2.2326074 | 0 | 0 | 0 |
| WP_DNA_MISMATCH_REPAIR | 23 | 0.75184345 | 2.0856464 | 0 | 4.57E-04 | 0.013 |
| GOBP_CELL_CYCLE_DNA_REPLICATION | 61 | 0.69321936 | 2.3576686 | 0 | 0 | 0 |

| **Negatively enriched in Trametinib vs Vehicle** | | | | | | |
| --- | --- | --- | --- | --- | --- | --- |
| **NAME** | **SIZE** | **ES** | **NES** | **NOM p-val** | **FDR q-val** | **FWER p-val** |
| HALLMARK_KRAS_SIGNALING_UP | 189 | -0.44424483 | -1.8301736 | 0 | 0.004256945 | 0.004 |
| HALLMARK_COMPLEMENT | 184 | -0.34994173 | -1.4470874 | 0.00243309 | 0.10617872 | 0.188 |
| HALLMARK_HEDGEHOG_SIGNALING | 36 | -0.45220286 | -1.4035985 | 0.05263158 | 0.11017557 | 0.275 |
| HALLMARK_ANGIOGENESIS | 35 | -0.43769482 | -1.3475701 | 0.082959644 | 0.14103438 | 0.426 |
| HALLMARK_TGF_BETA_SIGNALING | 54 | -0.374488 | -1.2700175 | 0.09677419 | 0.23175421 | 0.684 |
| HALLMARK_PROTEIN_SECRETION | 94 | -0.33262324 | -1.2530521 | 0.06413302 | 0.21974987 | 0.729 |
