# Supplementary material for: Effects of combination therapy of a CDK4/6 and MEK inhibitor in diffuse midline glioma preclinical models
Source: PLoS One. 2025 Dec 22;20(12):e0323235. doi: 10.1371/journal.pone.0323235 (PMC12721541; doi:10.1371/journal.pone.0323235)
Supplement: S9 Table — (DOCX) [file pone.0323235.s016.docx]

**Supplemental table 9. Gene set enrichment analysis comparing tumors treated with combination therapy and those treated with vehicle**

| **Positively enriched in Combination vs Vehicle** | | | | | | |
| --- | --- | --- | --- | --- | --- | --- |
| **NAME** | **SIZE** | **ES** | **NES** | **NOM p-val** | **FDR q-val** | **FWER p-val** |
| KEGG_DRUG_METABOLISM_CYTOCHROME_P450 | 42 | 0.60616106 | 1.8974358 | 0 | 0.14854956 | 0.248 |

| **Negatively enriched in Combination vs Vehicle** | | | | | | |
| --- | --- | --- | --- | --- | --- | --- |
| **NAME** | **SIZE** | **ES** | **NES** | **NOM p-val** | **FDR q-val** | **FWER p-val** |
| HALLMARK_TNFA_SIGNALING_VIA_NFKB | 196 | -0.53652775 | -2.1848779 | 0 | 0 | 0 |
| HALLMARK_KRAS_SIGNALING_UP | 189 | -0.50003284 | -2.0557435 | 0 | 8.48E-04 | 0.001 |
| HALLMARK_APOPTOSIS | 157 | -0.49309665 | -1.9537182 | 0 | 5.65E-04 | 0.001 |
| HALLMARK_EPITHELIAL_MESENCHYMAL_TRANSITION | 192 | -0.46552268 | -1.9077985 | 0 | 4.24E-04 | 0.001 |
| HALLMARK_HYPOXIA | 189 | -0.46741965 | -1.8837991 | 0 | 3.39E-04 | 0.001 |
| HALLMARK_ANGIOGENESIS | 35 | -0.6034829 | -1.8814076 | 0.00209205 | 7.82E-04 | 0.003 |
| HALLMARK_UV_RESPONSE_DN | 139 | -0.47987595 | -1.8647304 | 0 | 6.70E-04 | 0.003 |
| HALLMARK_MTORC1_SIGNALING | 188 | -0.438272 | -1.7898682 | 0 | 0.001006056 | 0.005 |
| HALLMARK_PI3K_AKT_MTOR_SIGNALING | 104 | -0.4775691 | -1.775411 | 0 | 0.001067605 | 0.006 |
| HALLMARK_PROTEIN_SECRETION | 94 | -0.47155416 | -1.7329471 | 0 | 0.001721845 | 0.011 |
| HALLMARK_TGF_BETA_SIGNALING | 54 | -0.523304 | -1.7289543 | 0.00204499 | 0.001565313 | 0.011 |
| HALLMARK_IL6_JAK_STAT3_SIGNALING | 85 | -0.47820365 | -1.725335 | 0 | 0.00143487 | 0.011 |
| HALLMARK_COMPLEMENT | 184 | -0.41815898 | -1.6928765 | 0 | 0.001580859 | 0.013 |
| HALLMARK_CHOLESTEROL_HOMEOSTASIS | 68 | -0.4664935 | -1.638773 | 0 | 0.002932911 | 0.024 |
| HALLMARK_INFLAMMATORY_RESPONSE | 195 | -0.39035058 | -1.589938 | 0 | 0.004757988 | 0.039 |
| HALLMARK_INTERFERON_GAMMA_RESPONSE | 185 | -0.39414242 | -1.5871997 | 0 | 0.004659676 | 0.041 |
| HALLMARK_MITOTIC_SPINDLE | 197 | -0.38639808 | -1.5795599 | 0 | 0.004717051 | 0.044 |
| HALLMARK_ALLOGRAFT_REJECTION | 178 | -0.38051024 | -1.5358496 | 0 | 0.007462494 | 0.075 |
| HALLMARK_P53_PATHWAY | 190 | -0.3697726 | -1.5136662 | 0 | 0.009138124 | 0.096 |
| HALLMARK_REACTIVE_OXYGEN_SPECIES_PATHWAY | 45 | -0.46074617 | -1.4928274 | 0.017429193 | 0.010178386 | 0.114 |
| HALLMARK_IL2_STAT5_SIGNALING | 193 | -0.3663158 | -1.4776064 | 0 | 0.011106659 | 0.131 |
| HALLMARK_INTERFERON_ALPHA_RESPONSE | 89 | -0.39753136 | -1.4646987 | 0.002183406 | 0.012177988 | 0.151 |
| HALLMARK_GLYCOLYSIS | 191 | -0.35374537 | -1.4502105 | 0.002232143 | 0.014191438 | 0.179 |
| HALLMARK_APICAL_JUNCTION | 194 | -0.34845832 | -1.4335222 | 0.004474273 | 0.016321559 | 0.215 |
| HALLMARK_COAGULATION | 132 | -0.3333329 | -1.3119173 | 0.03218884 | 0.057943206 | 0.57 |
| HALLMARK_MYOGENESIS | 197 | -0.29805762 | -1.2283503 | 0.05676856 | 0.12093044 | 0.839 |
| HALLMARK_HEDGEHOG_SIGNALING | 36 | -0.38313377 | -1.1924944 | 0.17038539 | 0.15749669 | 0.924 |
| HALLMARK_UNFOLDED_PROTEIN_RESPONSE | 107 | -0.3184756 | -1.1835332 | 0.12206573 | 0.16525199 | 0.942 |
| HALLMARK_ANDROGEN_RESPONSE | 93 | -0.31411967 | -1.1562278 | 0.1961207 | 0.20183782 | 0.971 |
| HALLMARK_G2M_CHECKPOINT | 188 | -0.28194013 | -1.1473372 | 0.12962963 | 0.2082313 | 0.979 |
| HALLMARK_APICAL_SURFACE | 43 | -0.3495739 | -1.1293205 | 0.2345679 | 0.23039542 | 0.987 |
