# Supplementary material for: Effects of combination therapy of a CDK4/6 and MEK inhibitor in diffuse midline glioma preclinical models
Source: PLoS One. 2025 Dec 22;20(12):e0323235. doi: 10.1371/journal.pone.0323235 (PMC12721541; doi:10.1371/journal.pone.0323235)
Supplement: S10 Table — (DOCX) [file pone.0323235.s017.docx]

**Supplemental table 10. Gene set enrichment analysis comparing tumors treated with ribociclib therapy and those treated with trametinib**

| **Negatively enriched in Ribociclib vs Trametinib** | | | | | | |
| --- | --- | --- | --- | --- | --- | --- |
| **NAME** | **SIZE** | **ES** | **NES** | **NOM p-val** | **FDR q-val** | **FWER p-val** |
| HALLMARK_E2F_TARGETS | 190 | -0.60799 | -2.43243 | 0 | 0 | 0 |
| HALLMARK_MYC_TARGETS_V2 | 58 | -0.681 | -2.34934 | 0 | 0 | 0 |
| HALLMARK_MYC_TARGETS_V1 | 192 | -0.5389 | -2.20817 | 0 | 0 | 0 |
| HALLMARK_G2M_CHECKPOINT | 188 | -0.50252 | -2.03951 | 0 | 0 | 0 |
| HALLMARK_MTORC1_SIGNALING | 188 | -0.43507 | -1.77983 | 0 | 0.001828 | 0.006 |
| HALLMARK_INTERFERON_ALPHA_RESPONSE | 89 | -0.48365 | -1.75862 | 0 | 0.001972 | 0.008 |
| HALLMARK_UNFOLDED_PROTEIN_RESPONSE | 107 | -0.45877 | -1.72488 | 0 | 0.001862 | 0.009 |
| HALLMARK_EPITHELIAL_MESENCHYMAL_TRANSITION | 192 | -0.39634 | -1.61634 | 0 | 0.007424 | 0.043 |
| HALLMARK_INTERFERON_GAMMA_RESPONSE | 185 | -0.38134 | -1.54702 | 0 | 0.01465 | 0.093 |
| HALLMARK_GLYCOLYSIS | 191 | -0.36455 | -1.47672 | 0 | 0.027355 | 0.186 |
| HALLMARK_OXIDATIVE_PHOSPHORYLATION | 178 | -0.33952 | -1.37064 | 0.005714 | 0.069879 | 0.436 |
| HALLMARK_ANDROGEN_RESPONSE | 93 | -0.37281 | -1.36374 | 0.025048 | 0.068545 | 0.463 |
| HALLMARK_ESTROGEN_RESPONSE_EARLY | 193 | -0.31872 | -1.28713 | 0.035382 | 0.130794 | 0.721 |
| HALLMARK_ESTROGEN_RESPONSE_LATE | 190 | -0.29942 | -1.22131 | 0.066667 | 0.227977 | 0.911 |
