# Supplementary material for: Effects of combination therapy of a CDK4/6 and MEK inhibitor in diffuse midline glioma preclinical models
Source: PLoS One. 2025 Dec 22;20(12):e0323235. doi: 10.1371/journal.pone.0323235 (PMC12721541; doi:10.1371/journal.pone.0323235)
Supplement: S11 Table — (DOCX) [file pone.0323235.s018.docx]

**Supplemental table 11. Gene set enrichment analysis comparing tumors treated with combination therapy and those treated with ribociclib**

| **Negatively enriched in Combination vs Ribociclib** | | | | | | |
| --- | --- | --- | --- | --- | --- | --- |
| **NAME** | **SIZE** | **ES** | **NES** | **NOM p-val** | **FDR q-val** | **FWER p-val** |
| HALLMARK_TNFA_SIGNALING_VIA_NFKB | 196 | -0.54745 | -2.21813 | 0 | 0 | 0 |
| HALLMARK_APOPTOSIS | 157 | -0.47769 | -1.92034 | 0 | 0 | 0 |
| HALLMARK_MITOTIC_SPINDLE | 197 | -0.45657 | -1.8806 | 0 | 5.47E-04 | 0.001 |
| HALLMARK_MTORC1_SIGNALING | 188 | -0.43908 | -1.81069 | 0 | 8.76E-04 | 0.002 |
| HALLMARK_PI3K_AKT_MTOR_SIGNALING | 104 | -0.47863 | -1.80746 | 0 | 7.01E-04 | 0.002 |
| HALLMARK_IL6_JAK_STAT3_SIGNALING | 85 | -0.49611 | -1.7993 | 0 | 5.84E-04 | 0.002 |
| HALLMARK_KRAS_SIGNALING_UP | 189 | -0.43307 | -1.7784 | 0 | 9.80E-04 | 0.004 |
| HALLMARK_ALLOGRAFT_REJECTION | 178 | -0.43663 | -1.76923 | 0 | 0.001102 | 0.005 |
| HALLMARK_CHOLESTEROL_HOMEOSTASIS | 68 | -0.50622 | -1.75903 | 0 | 9.80E-04 | 0.005 |
| HALLMARK_INTERFERON_GAMMA_RESPONSE | 185 | -0.43153 | -1.75697 | 0 | 8.82E-04 | 0.005 |
| HALLMARK_INTERFERON_ALPHA_RESPONSE | 89 | -0.4741 | -1.72356 | 0 | 0.001538 | 0.009 |
| HALLMARK_G2M_CHECKPOINT | 188 | -0.41182 | -1.6669 | 0 | 0.00252 | 0.016 |
| HALLMARK_P53_PATHWAY | 190 | -0.40754 | -1.65403 | 0 | 0.003358 | 0.023 |
| HALLMARK_TGF_BETA_SIGNALING | 54 | -0.46008 | -1.56217 | 0.012766 | 0.010002 | 0.07 |
| HALLMARK_COMPLEMENT | 184 | -0.38346 | -1.54262 | 0 | 0.010618 | 0.078 |
| HALLMARK_INFLAMMATORY_RESPONSE | 195 | -0.37149 | -1.52065 | 0 | 0.011758 | 0.092 |
| HALLMARK_REACTIVE_OXYGEN_SPECIES_PATHWAY | 45 | -0.46803 | -1.51652 | 0.008753 | 0.012019 | 0.1 |
| HALLMARK_UV_RESPONSE_DN | 139 | -0.36976 | -1.45108 | 0.006834 | 0.022375 | 0.192 |
| HALLMARK_HYPOXIA | 189 | -0.35371 | -1.44866 | 0 | 0.021706 | 0.197 |
| HALLMARK_WNT_BETA_CATENIN_SIGNALING | 41 | -0.44757 | -1.41287 | 0.045752 | 0.028943 | 0.266 |
| HALLMARK_MYOGENESIS | 197 | -0.33348 | -1.35645 | 0.007481 | 0.047938 | 0.402 |
| HALLMARK_IL2_STAT5_SIGNALING | 193 | -0.33129 | -1.35277 | 0.005 | 0.046883 | 0.408 |
| HALLMARK_HEDGEHOG_SIGNALING | 36 | -0.41986 | -1.32848 | 0.069351 | 0.057072 | 0.491 |
| HALLMARK_ANGIOGENESIS | 35 | -0.42597 | -1.31205 | 0.099783 | 0.064102 | 0.545 |
| HALLMARK_E2F_TARGETS | 190 | -0.32021 | -1.30923 | 0.012853 | 0.062603 | 0.553 |
| HALLMARK_APICAL_JUNCTION | 194 | -0.3096 | -1.28526 | 0.020202 | 0.073654 | 0.621 |
| HALLMARK_NOTCH_SIGNALING | 31 | -0.42629 | -1.28479 | 0.108 | 0.071132 | 0.622 |
