# Supplementary material for: Effects of combination therapy of a CDK4/6 and MEK inhibitor in diffuse midline glioma preclinical models
Source: PLoS One. 2025 Dec 22;20(12):e0323235. doi: 10.1371/journal.pone.0323235 (PMC12721541; doi:10.1371/journal.pone.0323235)
Supplement: S12 Table — (DOCX) [file pone.0323235.s019.docx]

**Supplemental table 12. Gene set enrichment analysis comparing tumors treated with combination therapy and those treated with trametinib**

| **Negatively enriched in Combination vs Trametinib** | | | | | | |
| --- | --- | --- | --- | --- | --- | --- |
| **NAME** | **SIZE** | **ES** | **NES** | **NOM p-val** | **FDR q-val** | **FWER p-val** |
| HALLMARK_E2F_TARGETS | 190 | -0.69176 | -2.80513 | 0 | 0 | 0 |
| HALLMARK_G2M_CHECKPOINT | 188 | -0.67382 | -2.72369 | 0 | 0 | 0 |
| HALLMARK_MTORC1_SIGNALING | 188 | -0.60864 | -2.45453 | 0 | 0 | 0 |
| HALLMARK_MYC_TARGETS_V2 | 58 | -0.68461 | -2.34437 | 0 | 0 | 0 |
| HALLMARK_INTERFERON_ALPHA_RESPONSE | 89 | -0.63366 | -2.26096 | 0 | 0 | 0 |
| HALLMARK_INTERFERON_GAMMA_RESPONSE | 185 | -0.5521 | -2.21544 | 0 | 0 | 0 |
| HALLMARK_MYC_TARGETS_V1 | 192 | -0.55091 | -2.1922 | 0 | 0 | 0 |
| HALLMARK_MITOTIC_SPINDLE | 197 | -0.51215 | -2.10035 | 0 | 0 | 0 |
| HALLMARK_TNFA_SIGNALING_VIA_NFKB | 196 | -0.52237 | -2.09333 | 0 | 0 | 0 |
| HALLMARK_IL6_JAK_STAT3_SIGNALING | 85 | -0.54824 | -1.99058 | 0 | 0 | 0 |
| HALLMARK_UNFOLDED_PROTEIN_RESPONSE | 107 | -0.4933 | -1.83697 | 0 | 1.56E-04 | 0.001 |
| HALLMARK_CHOLESTEROL_HOMEOSTASIS | 68 | -0.51525 | -1.81121 | 0 | 4.09E-04 | 0.003 |
| HALLMARK_GLYCOLYSIS | 191 | -0.4254 | -1.71777 | 0 | 0.001927 | 0.015 |
| HALLMARK_APOPTOSIS | 157 | -0.43654 | -1.71316 | 0 | 0.001789 | 0.015 |
| HALLMARK_PI3K_AKT_MTOR_SIGNALING | 104 | -0.45577 | -1.70142 | 0 | 0.00167 | 0.015 |
| HALLMARK_ALLOGRAFT_REJECTION | 178 | -0.42506 | -1.69949 | 0 | 0.001805 | 0.017 |
| HALLMARK_HYPOXIA | 189 | -0.40537 | -1.64694 | 0 | 0.002464 | 0.024 |
| HALLMARK_REACTIVE_OXYGEN_SPECIES_PATHWAY | 45 | -0.50639 | -1.63457 | 0.005917 | 0.002957 | 0.03 |
| HALLMARK_IL2_STAT5_SIGNALING | 193 | -0.40003 | -1.61752 | 0 | 0.003538 | 0.038 |
| HALLMARK_OXIDATIVE_PHOSPHORYLATION | 178 | -0.39861 | -1.5897 | 0 | 0.005098 | 0.056 |
| HALLMARK_UV_RESPONSE_DN | 139 | -0.40741 | -1.58036 | 0 | 0.00532 | 0.062 |
| HALLMARK_P53_PATHWAY | 190 | -0.3859 | -1.55638 | 0.001961 | 0.007069 | 0.088 |
| HALLMARK_EPITHELIAL_MESENCHYMAL_TRANSITION | 192 | -0.37248 | -1.52406 | 0 | 0.009348 | 0.12 |
| HALLMARK_KRAS_SIGNALING_UP | 189 | -0.3686 | -1.49425 | 0.00198 | 0.012669 | 0.162 |
| HALLMARK_INFLAMMATORY_RESPONSE | 195 | -0.35216 | -1.43557 | 0.004057 | 0.021504 | 0.261 |
| HALLMARK_APICAL_JUNCTION | 194 | -0.34489 | -1.39401 | 0.014344 | 0.030316 | 0.366 |
| HALLMARK_TGF_BETA_SIGNALING | 54 | -0.40931 | -1.3607 | 0.048583 | 0.041437 | 0.484 |
| HALLMARK_DNA_REPAIR | 141 | -0.32846 | -1.28061 | 0.022449 | 0.082701 | 0.743 |
| HALLMARK_SPERMATOGENESIS | 131 | -0.32589 | -1.25759 | 0.045545 | 0.100309 | 0.83 |
| HALLMARK_COAGULATION | 132 | -0.32515 | -1.25066 | 0.065263 | 0.103036 | 0.849 |
| HALLMARK_ANGIOGENESIS | 35 | -0.39953 | -1.24978 | 0.149312 | 0.100455 | 0.854 |
| HALLMARK_ANDROGEN_RESPONSE | 93 | -0.33934 | -1.24168 | 0.076023 | 0.103381 | 0.873 |
| HALLMARK_UV_RESPONSE_UP | 147 | -0.30924 | -1.20329 | 0.088063 | 0.137543 | 0.939 |
