# Supplementary material for: Effects of combination therapy of a CDK4/6 and MEK inhibitor in diffuse midline glioma preclinical models
Source: PLoS One. 2025 Dec 22;20(12):e0323235. doi: 10.1371/journal.pone.0323235 (PMC12721541; doi:10.1371/journal.pone.0323235)
Supplement: S13 Table — (PDF) [file pone.0323235.s020.pdf]

Supplemental table 13. Cell lines used in the study

LIST of the CELL LINES	
**NAME**	**GENETICAL BACKGROUND**
14-1214-1 (murine)	PDGFB; H3.3K27M; p53 loss
14-1206-1 (murine)	PDGFB; H3.3K27M; p53 loss
14-1206-5 (murine)	PDGFB; H3.3K27M; p53 loss
23-0509-2 (murine)	PDGFB; H3.3K27M; p53 loss
23-0104-3 (murine)	PDGFB; H3.3K27M; p53 loss
4738 (murine)	PDGFB; H3.3K27M; p53 loss
BT-245 (human)	H3.3K27M; H424Y and P443L mutations in PDGFRA; TP53.R249S; MYC amplification, CDKN2A/B loss
SF8628 (human)	H3.3K27M
SF7761 (human)	H3.3K27M
HSJD-DIPG-007 (human)	H3.3K27M; ACVR1 R206H; PPM1D Pro428Ginfs*3
SU-DIPG17 (human)	H3.3K27M
